# Circulating inflammatory proteins and osteomyelitis: A bidirectional Mendelian randomization and colocalization analysis

**DOI:** 10.1097/MD.0000000000044916

**Published:** 2025-10-17

**Authors:** Tianxuan Feng, Peisheng Chen, Fengfei Lin

**Affiliations:** a Fujian University of Traditional Chinese Medicine, Fuzhou, Fujian, China; b Department of Orthopedic, Fuzhou Second General Hospital, School of Clinical Medicine of Fujian Medical University, Fujian Provincial Clinical Medical Research Center for First Aid and Rehabilitation in Orthopedic Trauma, Fuzhou, Fujian, China.

**Keywords:** colocalization, inflammatory protein, Mendelian randomization, osteomyelitis

## Abstract

Circulating inflammatory proteins (CIPs) have been implicated in the progression of osteomyelitis (OM); however, whether these proteins play a causal role or are merely a consequence remains unclear. This study aimed to assess the causal relationships between CIPs and OM using a bidirectional 2-sample Mendelian randomization (MR) approach. MR analyses were performed using genome-wide association study summary statistics for 91 inflammation-related proteins (n = 14,824) and OM (1881 cases and 3,91,037 controls). The inverse variance weighted method was used as the primary analytical approach, supplemented by MR-Egger, weighted median, simple mode, and weighted mode methods. Sensitivity analyses were conducted to evaluate heterogeneity, horizontal pleiotropy, and robustness. Colocalization analysis was applied to identify shared causal variants, and pathway enrichment analysis was used to explore underlying biological mechanisms. Forward MR analysis revealed that elevated levels of tumor necrosis factor-beta (TNF-β) were significantly associated with increased OM risk (odds ratio [OR] = 1.132; 95% confidence interval [CI]: 1.052–1.217; false discovery rate [FDR] = 0.027). Conversely, decreased levels of osteoprotegerin (OR = 0.772; 95% CI: 0.671–0.889; FDR = 0.015) and adenosine deaminase (OR = 0.811; 95% CI: 0.736–0.894; FDR < 0.001) were associated with increased OM risk. Reverse MR analysis identified increased levels of interleukin-15 receptor alpha, C-X-C motif chemokine ligand 1, fms-related tyrosine kinase 3 ligand, interleukin-20, interleukin-10 (IL10), C-C motif chemokine ligand 19, and CXCL6 as being significantly associated with OM susceptibility (all FDR < 0.05). Colocalization analysis provided strong evidence for a shared causal variant between TNF-β and OM (posterior probability for hypothesis 4 = 0.999). Enrichment analyses indicated involvement of implicated proteins in Toll-like receptor signaling and T-helper 17 cell differentiation pathways. This study identified several CIPs – including TNF-β, osteoprotegerin, and adenosine deaminase – as potentially causal in OM development. These findings highlight promising targets for future immunomodulatory therapies aimed at preventing or mitigating osteomyelitis.

## 1. Introduction

Osteomyelitis (OM) is a severe bone infection characterized by localized bone pain, erythema, fever, and systemic weakness.^[1,2]^ If untreated, OM can lead to skeletal deformities, arthritis, and chronic pain.^[3]^ The global burden of OM has risen markedly in recent years, with hospitalization rates increasing from 2.9 to 5.4 per 1,00,000 individuals and case numbers climbing from 8021 to 16,917. This trend highlights both the growing prevalence and the considerable economic burden of the disease, now estimated in the billions of dollars.^[4–6]^

The principal causative organism is *Staphylococcus aureus (S aureus*), which can invade bone tissue through hematogenous spread or direct inoculation.^[7]^ Standard treatment involves surgical debridement in combination with prolonged antibiotic therapy.^[8,9]^ Despite these measures, effective pharmacological strategies to prevent or halt disease progression remain elusive.^[10]^ High-risk groups include individuals with diabetes, peripheral vascular disease, or recent trauma, as well as children and the elderly.^[11,12]^ Moreover, genetic predisposition contributes to susceptibility, with several genetic markers identified as risk factors for OM.^[13–15]^

Accumulating evidence underscores the pivotal role of inflammation in OM pathogenesis.^[16,17]^ Genomic analyses of infected bone tissue have revealed activation of multiple inflammatory pathways, reflecting complex interactions between the immune system and the infectious process.^[18]^ Inflammation serves a dual function: while essential for pathogen clearance and tissue repair, sustained or excessive activation undermines bone integrity and aggravates disease severity.^[19,20]^

Several key inflammatory markers – such as interleukin-1 (IL-1), tumor necrosis factor-alpha (TNF-α), and C-reactive protein (CRP) – are consistently elevated in patients with OM. These cytokines not only drive disease pathogenesis but also influence disease progression through diverse cellular and molecular mechanisms.^[21,22]^

Beyond soluble mediators, recent Mendelian randomization (MR) studies have demonstrated causal roles for specific immune cell subsets – including CD8^⁺^ T cells, memory B cells, plasmacytoid dendritic cells, and regulatory T cells – in OM susceptibility.^[13,14]^ Dysregulation of the immune-inflammatory axis, such as increased expression of human leukocyte antigen – DR isotype (HLA-DR) on dendritic and B-cell subsets and elevated plasma levels of CD6 and interleukin-12B (IL-12B), has also been linked to heightened OM risk in European populations.^[23]^ These findings highlight the multifaceted contributions of immune cells, cytokines, chemokines, and other inflammatory mediators to OM pathogenesis, suggesting potential targets for precision immunomodulatory therapies.

To clarify the causal role of inflammation in OM, we employed a bidirectional MR framework – a robust analytical approach that leverages genetic variants as instrumental variables to infer causality.^[24]^ Because genetic variants are fixed at conception and not influenced by environmental exposures, MR analysis helps reduce confounding and reverse causation bias.^[25]^ While previous investigations have primarily examined individual inflammatory markers, the broader landscape of circulating inflammatory proteins (CIPs) remains insufficiently explored, potentially overlooking critical contributors.

In this study, we performed a comprehensive bidirectional 2-sample MR analysis integrating genome-wide association study (GWAS) data on CIPs and OM. Furthermore, colocalization analyses were applied to identify shared causal variants, and pathway enrichment analyses were conducted to explore relevant biological processes (BP). This integrative strategy aims to delineate causal proteins and pathways underlying OM, thereby providing insights that may inform the development of targeted therapeutic interventions.

## 2. Materials and methods

### 2.1. Study design

**Figure 1.**
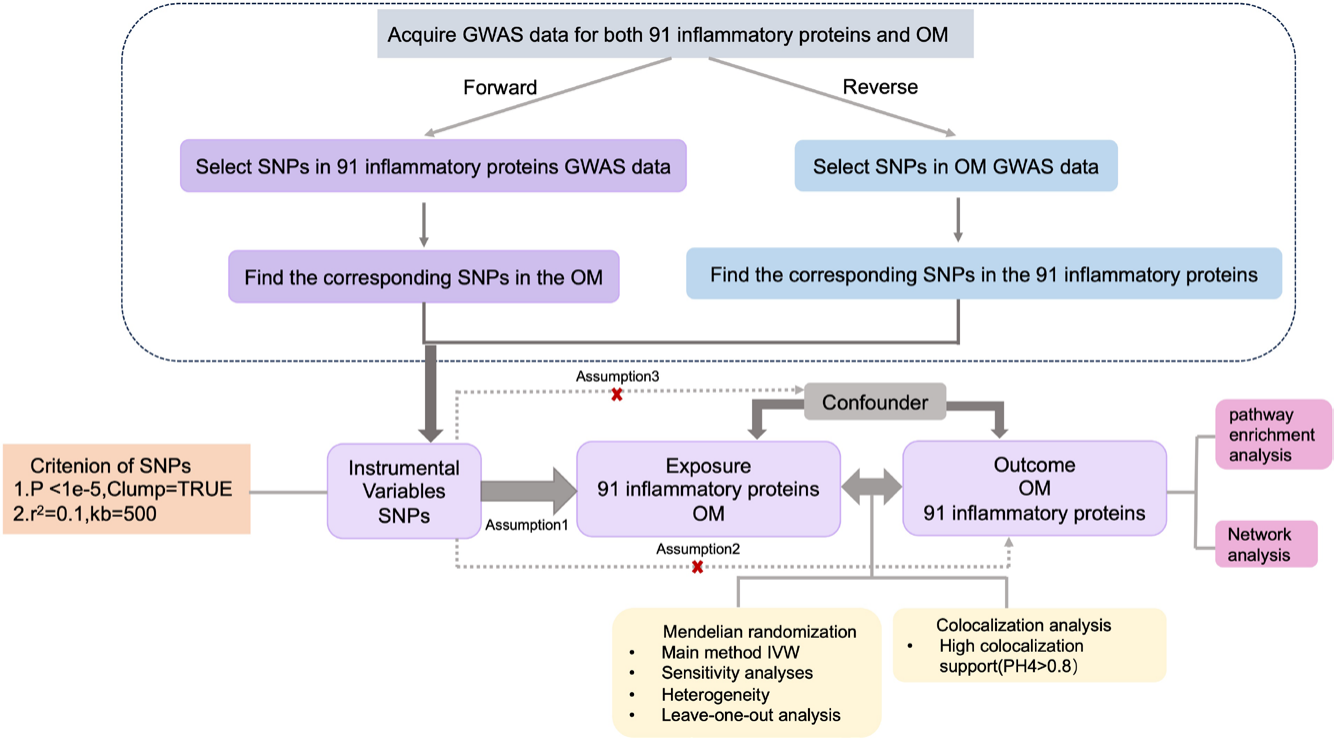
Flowchart overview of the assumptions of the Mendelian randomization design and the study design.

The overall study design is illustrated in Figure 1. This study utilized publicly available GWAS summary statistics for CIPs from the Cambridge Proteomics dataset (https://www.phpc.cam.ac.uk/ceu/proteins/)^[26]^ and OM data from the FinnGen study (https://www.finngen.fi/en).^[27]^ All analyses were based on summary-level data from populations of European ancestry, as detailed in Table S1, Supplemental Digital Content, https://links.lww.com/MD/Q245. Ethical approval had been obtained for each contributing GWAS dataset.

### 2.2. Plasma inflammatory protein data sources

Data on 91 CIPs were derived from a study including 14,824 individuals of European ancestry across 11 cohorts.^[26]^ Plasma protein levels were quantified using the Olink targeted inflammation panel, with all assays performed at Olink laboratories in Uppsala, Sweden.^[27]^ The original study aimed to identify genetic determinants of immune-mediated disease risk and potential therapeutic targets within the CIP profile.

### 2.3. Osteomyelitis data sources

OM data were obtained from the R10 release of the FinnGen project, including 1881 OM cases and 3,91,037 controls.^[28]^ FinnGen is a large-scale biobank and genomics initiative that integrates genetic and clinical data from over 5,00,000 Finnish individuals to elucidate genetic determinants of disease.

### 2.4. Mendelian randomization analysis

MR analysis was conducted in accordance with the 3 core instrumental variable assumptions^[29]^ and followed the strengthening the reporting of observational studies in epidemiology using Mendelian randomization reporting guidelines.^[30]^ Single nucleotide polymorphisms (SNPs) significantly associated with each protein (*P* < 1 × 10^−^⁵) were extracted from the CIP GWAS dataset. Linkage disequilibrium clumping was performed using a threshold of *r*² = 0.001 within a 10,000 kb window. SNPs with potential pleiotropic effects or weak instrument strength (*F*-statistic < 10) were excluded.^[31]^

### 2.5. Statistical analysis

All statistical analyses adhered to the strengthening the reporting of observational studies in epidemiology using Mendelian randomization reporting guidelines.^[30]^ The primary MR method was the inverse variance weighted (IVW) approach, which provides efficient estimates under the assumption that all genetic instruments are valid. To address potential violations of MR assumptions, sensitivity analyses were performed using MR-Egger regression, the weighted median estimator, simple mode, and weighted mode methods.

Heterogeneity across instrumental variables was assessed using Cochran’s *Q* test. Horizontal pleiotropy was evaluated with both the MR-Egger intercept test and the MR-PRESSO (MR Pleiotropy RESidual Sum and Outlier) global test. A leave-one-out analysis was further conducted to examine the robustness of causal estimates by iteratively excluding each SNP.

To verify the directionality of associations, reverse MR analyses were carried out to assess potential causal effects of OM on CIPs. For multiple testing correction, the false discovery rate (FDR) method was applied, with statistical significance defined as FDR < 0.05.

All analyses were conducted using R software (version 4.3.3) with the “TwoSampleMR” and “MendelianRandomization” packages.

### 2.6. Colocalization analysis

Colocalization analysis was performed to investigate whether CIPs and OM share a common causal variant within the same genomic locus.^[32]^ The “coloc” package in R (version 4.3.3) was used to analyze a 1 Mb region surrounding each gene of interest. All SNPs within this region were extracted and tested under a Bayesian framework considering 5 hypotheses: H0 (no association with either trait), H1 (association with CIPs only), H2 (association with OM only), H3 (association with both traits but with distinct causal variants), and H4 (shared causal variant). Posterior probabilities were calculated, and probability for hypothesis 4 ≥ 0.8 was regarded as strong evidence of colocalization.^[33]^

### 2.7. Pathway enrichment analysis

To explore functional pathways involving CIPs associated with OM, gene ontology and Kyoto Encyclopedia of Genes and Genomes (KEGG) enrichment analyses were conducted using the “clusterProfiler” package in R.^[34]^ The results were visualized with circular and bubble plots. In addition, a protein–protein interaction network was constructed using GeneMANIA (https://genemania.org/) to identify potential regulatory interactions.

## 3. Results

### 3.1. Exploring the causal links between CIPs and OM through MR

Given the limited number of genetic variants and the modest effect sizes of protein quantitative trait locus, a relaxed genome-wide significance threshold (*P* < 1 × 10^−^⁵) was applied to select instrumental SNPs for forward MR analysis, ensuring sufficient SNP coverage (Table S2, Supplemental Digital Content, https://links.lww.com/MD/Q245). Summary statistics for the 91 CIPs are presented in Tables S3 and S6, Supplemental Digital Content, https://links.lww.com/MD/Q245.

**Figure 2.**
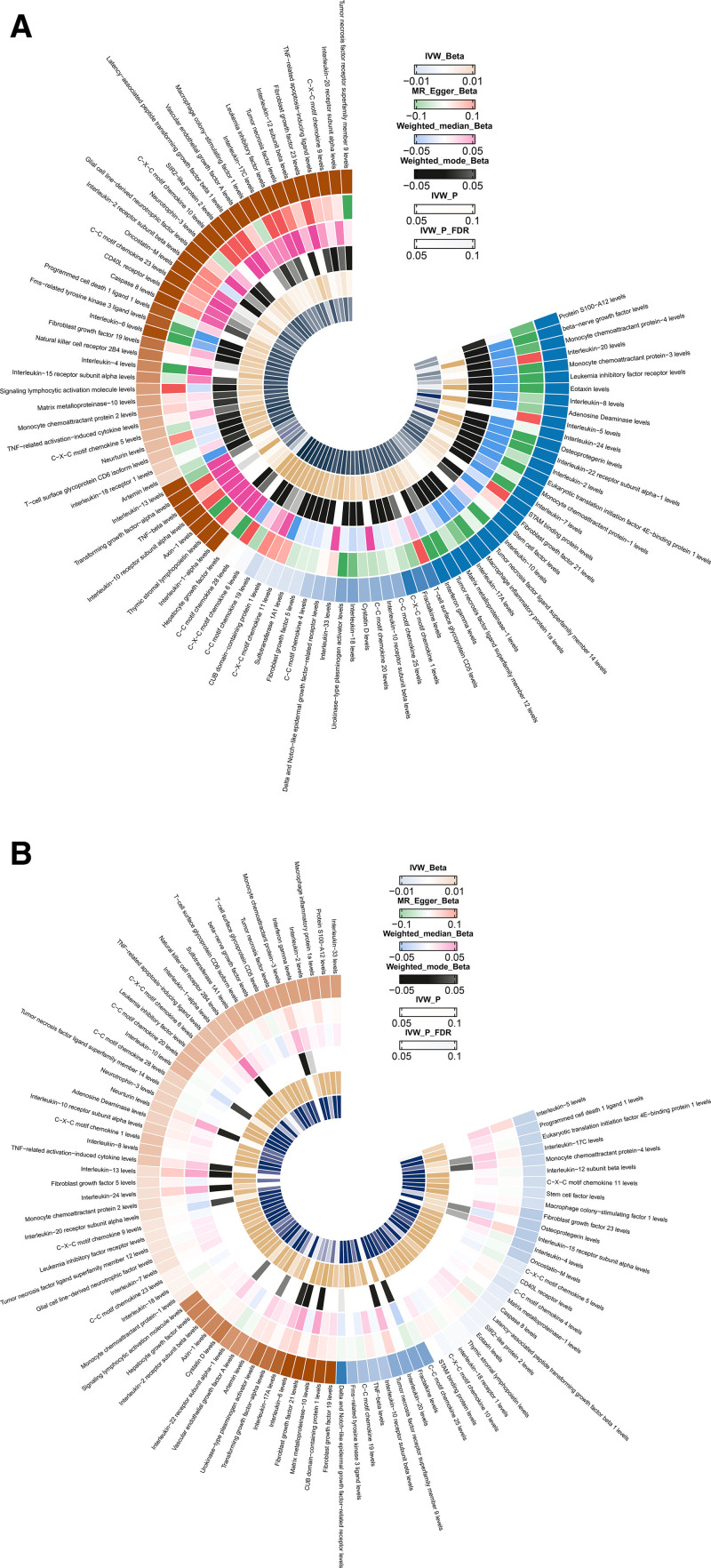
(A) Ring heat map of MR analysis of the causal association of CIPs on OM. (B) Ring heat map of MR analysis of the causal association of OM on CIPs. CIPs = circulating inflammatory proteins, MR = Mendelian randomization, OM = osteomyelitis.

**Figure 3.**
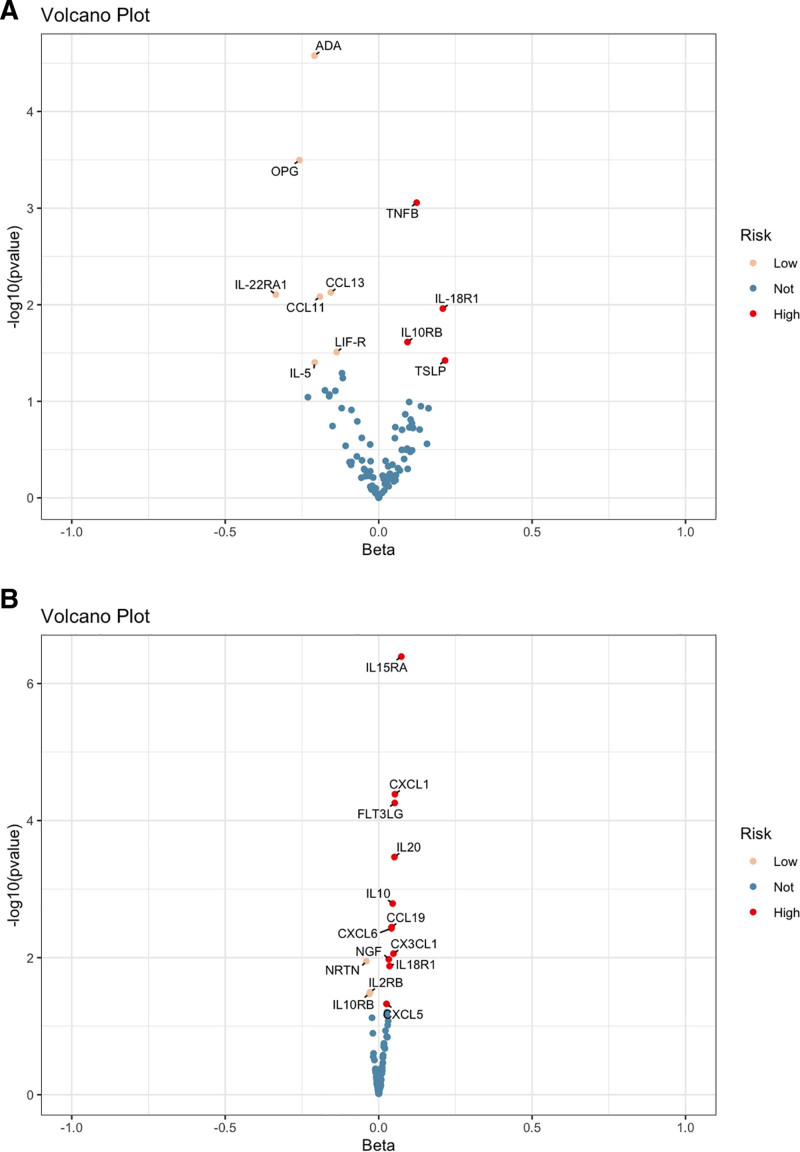
Volcano plot summary results of 91 CIPs and OM using the IVW method in MR analysis. Volcano plots show the MR results of 91 CIPs on the risk of OM in forward Mendelian randomization (A) and reverse Mendelian randomization (B), respectively. CIPs = circulating inflammatory proteins, IVW = inverse variance weighted, MR = Mendelian randomization, OM = osteomyelitis.

Two-sample MR analysis was conducted to assess the causal effects of CIPs on OM. The IVW method served as the primary analytical approach, and FDR correction was applied to account for multiple testing. Key results are illustrated in Figure 2A (ring heat map) and Figure 3A (volcano plot), with detailed statistics provided in Table S4, Supplemental Digital Content, https://links.lww.com/MD/Q245.

The IVW method identified several proteins positively associated with OM risk. Notably, genetically predicted elevated levels of lymphotoxin-α (LTA; OR = 1.132, 95% confidence interval [CI]: 1.052–1.217, *P* = .001), interleukin-18 receptor 1 (OR = 1.233, 95% CI: 1.049–1.448, *P* = .011), interleukin-10 receptor subunit β (OR = 1.098, 95% CI: 1.012–1.192, *P* = .024), and thymic stromal lymphopoietin (OR = 1.241, 95% CI: 1.012–1.522, *P* = .038) were significantly associated with increased OM susceptibility.

Conversely, genetically predicted lower levels of several proteins were significantly associated with reduced OM risk, including adenosine deaminase (ADA; OR = 0.811, 95% CI: 0.736–0.894, *P* < .001), tumor necrosis factor receptor superfamily member 11B (TNFRSF11B; OR = 0.772, 95% CI: 0.671–0.889, *P* < .001), C-C motif chemokine ligand (CCL13; OR = 0.856, 95% CI: 0.763–0.959, *P* = .007), interleukin-22 receptor subunit α1 (OR = 0.715, 95% CI: 0.558–0.916, *P* = .008), CCL11 (OR = 0.826, 95% CI: 0.716–0.952, *P* = .008), leukemia inhibitory factor receptor (OR = 0.872, 95% CI: 0.770–0.987, *P* = .031), and interleukin-5 (OR = 0.812, 95% CI: 0.665–0.990, *P* = .040).

After FDR correction, 3 proteins remained statistically significant: TNFRSF11B (FDR = 0.015), LTA (FDR = 0.027), and ADA (FDR < 0.001), supporting their potential causal role in OM pathogenesis. Full results of heterogeneity and pleiotropy sensitivity analyses are available in Table S4, Supplemental Digital Content, https://links.lww.com/MD/Q245. Visual diagnostics – including scatter plots, funnel plots, forest plots, and leave-one-out analyses – are presented in Figures S1 to S11, Supplemental Digital Content, https://links.lww.com/MD/Q246 and Tables S1 to S11, Supplemental Digital Content, https://links.lww.com/MD/Q247.

### 3.2. Reverse Mendelian randomization: Exploring the causal links between OM and CIPs

To evaluate whether OM causally influences circulating levels of CIPs, a 2-sample reverse MR analysis was conducted using the IVW method as the primary approach. Statistical significance was determined after FDR correction. The main results are visualized in Figure 2B (ring heat map) and Figure 3B (volcano plot), with complete summary statistics provided in Tables S7 and S8, Supplemental Digital Content, https://links.lww.com/MD/Q245.

The reverse MR analysis revealed that genetically predicted OM susceptibility was significantly associated with elevated levels of several CIPs. Notably, OM was linked to increased levels of interleukin-15 receptor α (IL15RA; odds ratio [OR] = 1.077, 95% CI: 1.046–1.108, *P* < .001), C-X-C motif chemokine ligand 1 (CXCL1; OR = 1.054, 95% CI: 1.028–1.081, *P* < .001), fms-related tyrosine kinase 3 ligand (FLT3LG; OR = 1.054, 95% CI: 1.027–1.081, *P* < .001), interleukin-20 (IL20; OR = 1.053, 95% CI: 1.023–1.082, *P* < .001), and IL10 (OR = 1.047, 95% CI: 1.017–1.077, *P* = .002). Significant associations were also observed for CCL19 (OR = 1.043, 95% CI: 1.014–1.072, *P* = .004), CXCL6 (OR = 1.042, 95% CI: 1.014–1.072, *P* = .004), and C-X3-C motif chemokine ligand 1 (OR = 1.049, 95% CI: 1.012–1.088, *P* = .009).

In contrast, OM was associated with reduced levels of neurturin (NRTN; OR = 0.961, 95% CI: 0.931–0.991, *P* = .011), interleukin-2 receptor β (OR = 0.973, 95% CI: 0.948–0.998, *P* = .032), and interleukin-10 receptor subunit β (OR = 0.970, 95% CI: 0.943–0.998, *P* = .034). A modest positive association was also observed between OM and CXCL5 (OR = 1.026, 95% CI: 1.000–1.052, *P* = .047).

FDR-adjusted analysis confirmed statistically significant associations for IL15RA (FDR = 3.68 × 10^−^⁵), CXCL1 (FDR = 0.001), FLT3LG (FDR = 0.001), IL20 (FDR = 0.007), IL10 (FDR = 0.030), CCL19 (FDR = 0.049), and CXCL6 (FDR = 0.049), supporting the robustness of these findings. These results suggest that OM may causally influence systemic inflammatory profiles by modulating specific CIPs, potentially contributing to sustained immune activation.

Heterogeneity and horizontal pleiotropy test results for the reverse MR analysis are provided in Table S8, Supplemental Digital Content, https://links.lww.com/MD/Q245. Scatter plots, funnel plots, forest plots, and leave-one-out sensitivity analyses are available in Figures S12 to S25, Supplemental Digital Content, https://links.lww.com/MD/Q248 and Tables S12 to S25, Supplemental Digital Content, https://links.lww.com/MD/Q249.

### 3.3. Colocalization analysis

**Figure 4.**
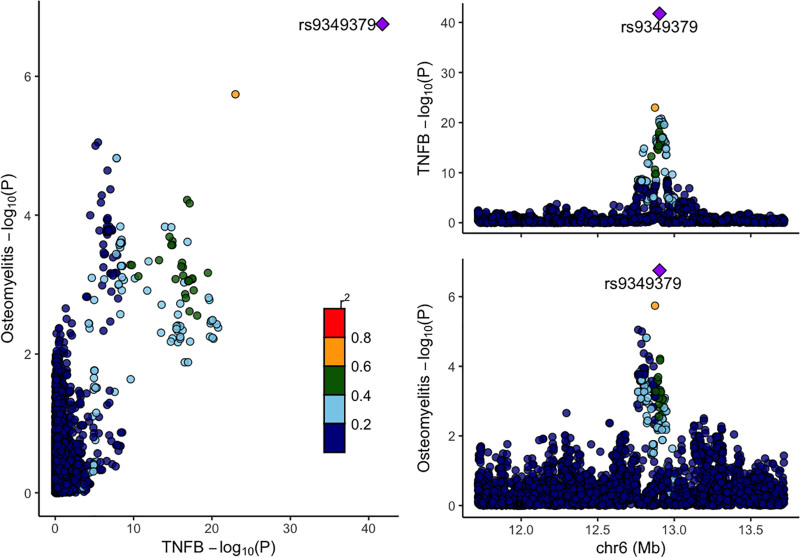
Colocalization Plot Colocalization analysis for the relationship between the CIPs and OM. CIPs = circulating inflammatory proteins, OM = osteomyelitis.

To assess shared genetic architecture between CIPs and OM, colocalization analysis was performed for proteins meeting the FDR significance threshold. Among these, only TNF-β (PH4 = 0.999) showed strong evidence of colocalization (Fig. 4), suggesting a shared causal variant between TNF-β and OM. Full colocalization results are provided in Table S5, Supplemental Digital Content, https://links.lww.com/MD/Q245.

### 3.4. Pathway and network analysis

**Figure 5.**
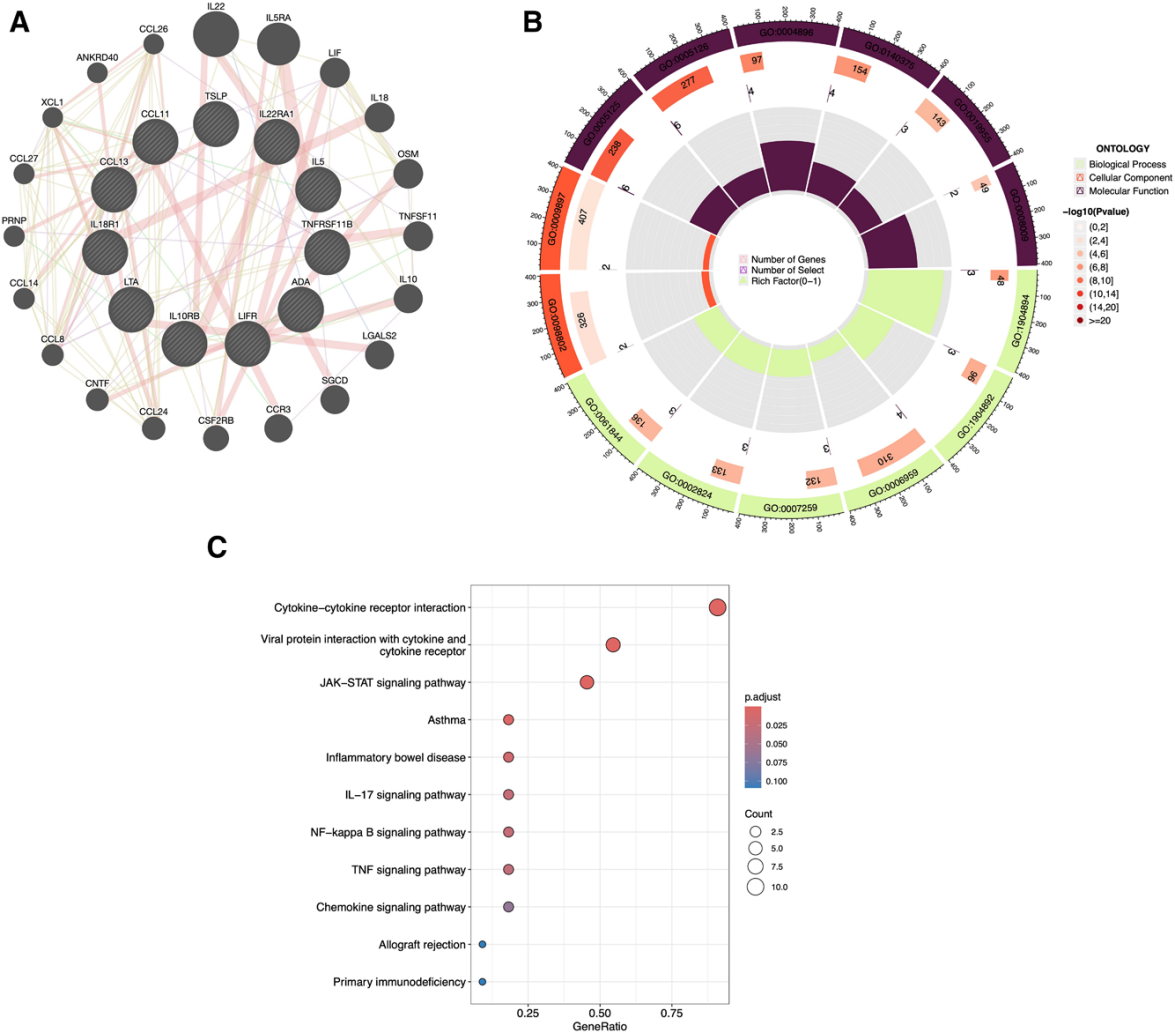
(A) Co-expression network and functional hypothesis analysis based on the GeneMANIA platform, with the 91 inflammatory proteins as exposures and OM as the outcome. (B) Circular chart of the GO analysis results. (C) Bubble chart of the KEGG pathway enrichment analysis. GO = gene ontology, KEGG = Kyoto Encyclopedia of Genes and Genomes, OM = osteomyelitis.

Proteins with evidence of causal association with OM from the forward MR analysis were further subjected to functional enrichment and network analysis. GeneMANIA was used to construct a co-expression network by integrating expression quantitative trait loci data from human liver tissue and innate immune response datasets (Fig. 5A). The resulting network included 2,91,300 interactions, suggesting extensive regulatory roles in immune signaling.

Gene ontology enrichment analysis revealed significant involvement of these proteins in 265 terms across BP, cellular components, and molecular functions. Enriched BP terms included regulation of cytokine production and Toll-like receptor signaling; cellular components terms were enriched for phagocytic vesicle membranes and endocytic compartments. Key molecular functions terms involved glycosyl hydrolase activity and NAD⁺ nucleotidase activity (Table S9, Supplemental Digital Content, https://links.lww.com/MD/Q245).

KEGG pathway enrichment identified 80 significantly overrepresented pathways, including the Toll-like receptor signaling pathway, the cytosolic DNA-sensing pathway, and T-helper 17 cell cell differentiation (Fig. 5B, C). These findings underscore the involvement of inflammatory proteins in OM-related immune regulation and highlight potential therapeutic targets.

## 4. Discussion

This study aimed to clarify the causal relationship between CIPs and OM. Using bidirectional MR, we examined whether specific CIPs influence OM risk and whether OM alters systemic inflammatory profiles. Colocalization and enrichment analyses further identified shared causal variants and biological pathways involved in these associations.

Forward MR analysis revealed that higher genetically predicted levels of ADA and osteoprotegerin (also known as tumor necrosis factor receptor superfamily member 11B; OPG) were associated with reduced OM risk, suggesting protective roles, whereas increased levels of LTA were linked to higher susceptibility. Colocalization analysis provided strong evidence for a shared causal variant between TNF-β and OM.

These protein-level findings align with recent MR studies demonstrating that specific immune cell subsets contribute causally to OM susceptibility. For instance, previous research has shown that CD8^⁺^ T cells, memory B cells, dendritic cells, and regulatory T cells all participate in OM-related immune dysregulation.^[13,14,23]^ Collectively, these data support the hypothesis that immune cells not only secrete but also regulate CIPs, shaping the immune-inflammatory landscape underlying OM pathogenesis.

ADA regulates immune responses by degrading adenosine, an immunosuppressive metabolite that accumulates during chronic infection.^[35–38]^ By limiting adenosine-mediated suppression of T cells and macrophages, elevated ADA activity may restore immune activation, promote bacterial clearance, and mitigate infection-induced bone destruction. Thus, ADA could serve as a potential therapeutic adjunct in OM management.

OPG, a decoy receptor for receptor activator of nuclear factor kappa-Β ligand, inhibits osteoclast-mediated bone resorption, thereby preserving bone architecture in OM.^[39–41]^ In addition to skeletal protection, OPG has been shown to modulate inflammatory cytokine production, suggesting a dual role in immune regulation and bone homeostasis.

TNF-β is a pro-inflammatory cytokine that promotes immune cell recruitment and osteoclast activation, contributing to both pathogen clearance and tissue damage.^[42–45]^ It can upregulate matrix metalloproteinases, which degrade extracellular matrix components, thereby exacerbating bone degradation. These findings underscore TNF-β as a central mediator of OM pathology and a potential biomarker or therapeutic target.

Functional pathway analyses (gene ontology and KEGG) confirmed enrichment in immune-related signaling cascades, including TNF and nuclear factor kappa B pathways. These pathways regulate cytokine release, leukocyte recruitment, and inflammation resolution. Disruption of these processes may lead to persistent inflammation and bone loss in OM. GeneMANIA further highlighted ADA, TNFRSF11B, and LTA as network hubs in immune regulation and host-pathogen interaction.

Reverse MR analysis indicated that OM itself may influence circulating inflammatory protein profiles. Genetically predicted OM risk was associated with increased levels of IL15RA, CXCL1, FLT3LG, IL20, IL10, CCL19, and CXCL6. These mediators are involved in immune cell proliferation, migration, and cytokine signaling.^[46–53]^ Notably, concurrent elevation of IL10 and IL20 suggests the presence of both pro-inflammatory and anti-inflammatory feedback mechanisms.^[54–57]^

A major strength of this study lies in the integration of bidirectional MR, colocalization, and enrichment analyses, which collectively strengthen the causal inference and biological interpretation. The use of large-scale GWAS data from European populations also enhances the generalizability of our findings within this ancestry group.

However, several limitations should be noted. First, due to limited instrument strength, we applied a lenient SNP selection threshold (*P* < 1 × 10^−^⁵), which may increase the risk of false positives. Second, the analysis was restricted to individuals of European descent, limiting its trans-ethnic applicability. Finally, although sensitivity tests addressed pleiotropy, residual horizontal pleiotropy cannot be entirely ruled out.

## 5. Conclusion

This study employed bidirectional MR, colocalization, and pathway enrichment analyses to investigate the causal links between CIPs and OM. TNF-β, OPG, and ADA were identified as potential causal factors contributing to OM development and progression. Reverse MR analysis further suggested that OM may reshape systemic inflammation by elevating specific CIPs. These findings enhance our understanding of OM pathogenesis and highlight novel therapeutic targets for future clinical and translational research.

## Acknowledgments

We would like to thank the participants and investigators of the FinnGen study (https://finngen.gitbook.io/documentation/) and UK Biobank (https://www.ukbiobank.ac.uk/) for sharing the genetic data included in the present work. Additionally, we used GeneMANIA (https://genemania.org/) as a tool for data analysis and visualization.

## Author contributions

**Data curation:** Peisheng Chen.

**Supervision:** Fengfei Lin.

**Writing – original draft:** Tianxuan Feng.

## Supplementary Material










