# Supplementary material for: Circulating inflammatory proteins and osteomyelitis: A bidirectional Mendelian randomization and colocalization analysis
Source: Medicine (Baltimore). 2025 Oct 17;104(42):e44916. doi: 10.1097/MD.0000000000044916 (PMC12537171; doi:10.1097/MD.0000000000044916)
Supplement: Supplementary file 2 [file medi-104-e44916-s002.pdf]

Supplementary Figures S1–S11. Diagnostic and sensitivity plots for forward Mendelian randomization (MR) analyses, including scatter plots, forest plots, funnel plots, and leave-one-out analyses for each significant protein–osteomyelitis association.

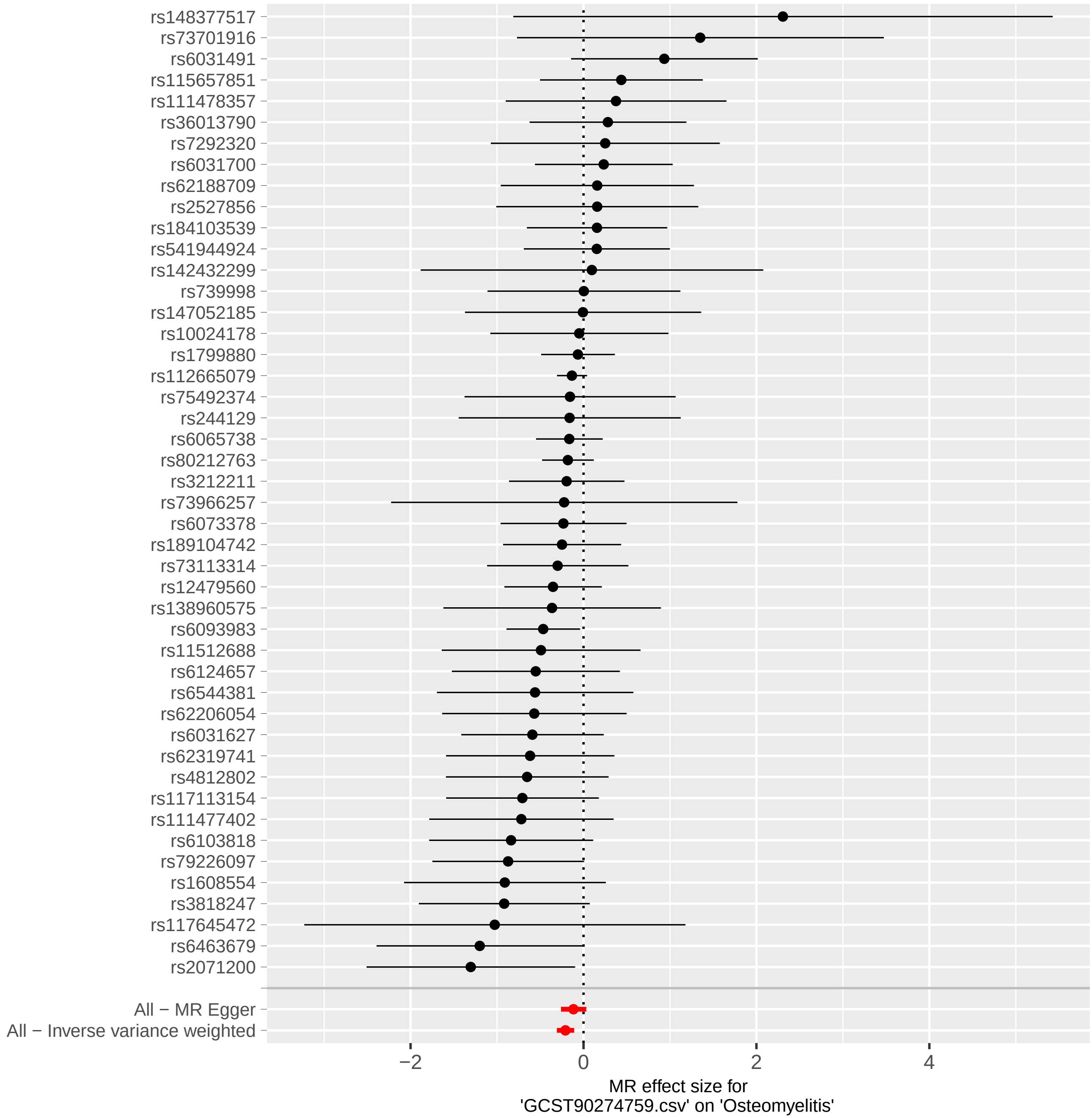
rs148377517
rs73701916
rs6031491
rs115657851
rs111478357
rs36013790
rs7292320
rs6031700
rs62188709
rs2527856
rs184103539
rs541944924
rs142432299
rs739998
rs147052185
rs10024178
rs1799880
rs112665079
rs75492374
rs244129
rs6065738
rs80212763
rs3212211
rs73966257
rs6073378
rs189104742
rs73113314
rs12479560
rs138960575
rs6093983
rs11512688
rs6124657
rs6544381
rs62206054
rs6031627
rs62319741
rs4812802
rs117113154
rs111477402
rs6103818
rs79226097
rs1608554
rs3818247
rs117645472
rs6463679
rs2071200
All − MR Egger
All − Inverse variance weighted
−2
0
2
4
MR effect size for
'GCST90274759.csv' on 'Osteomyelitis'

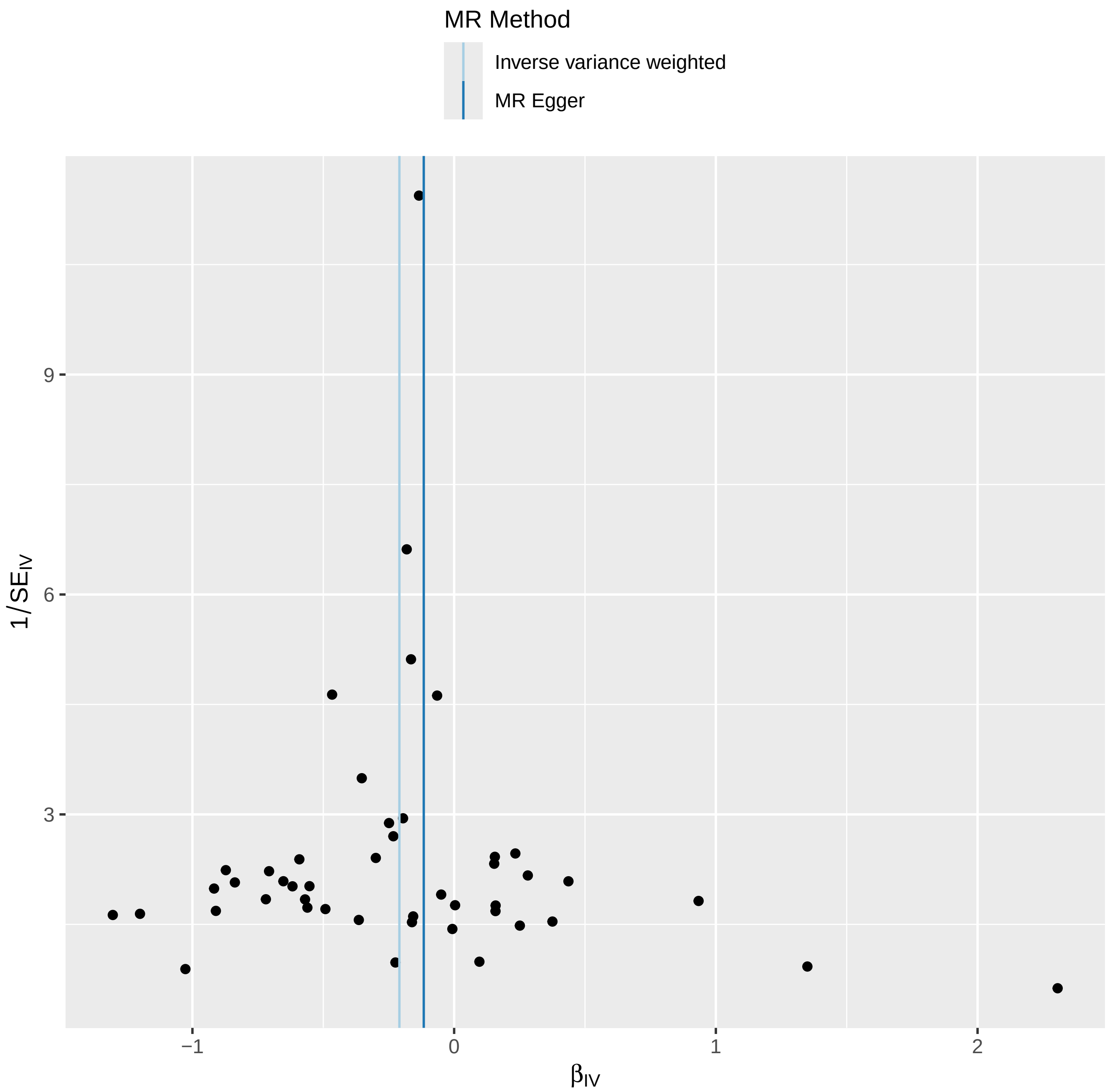
MR Method
Inverse variance weighted
MR Egger
1/SE_IV
β_IV
9
6
3
−1
0
1
2

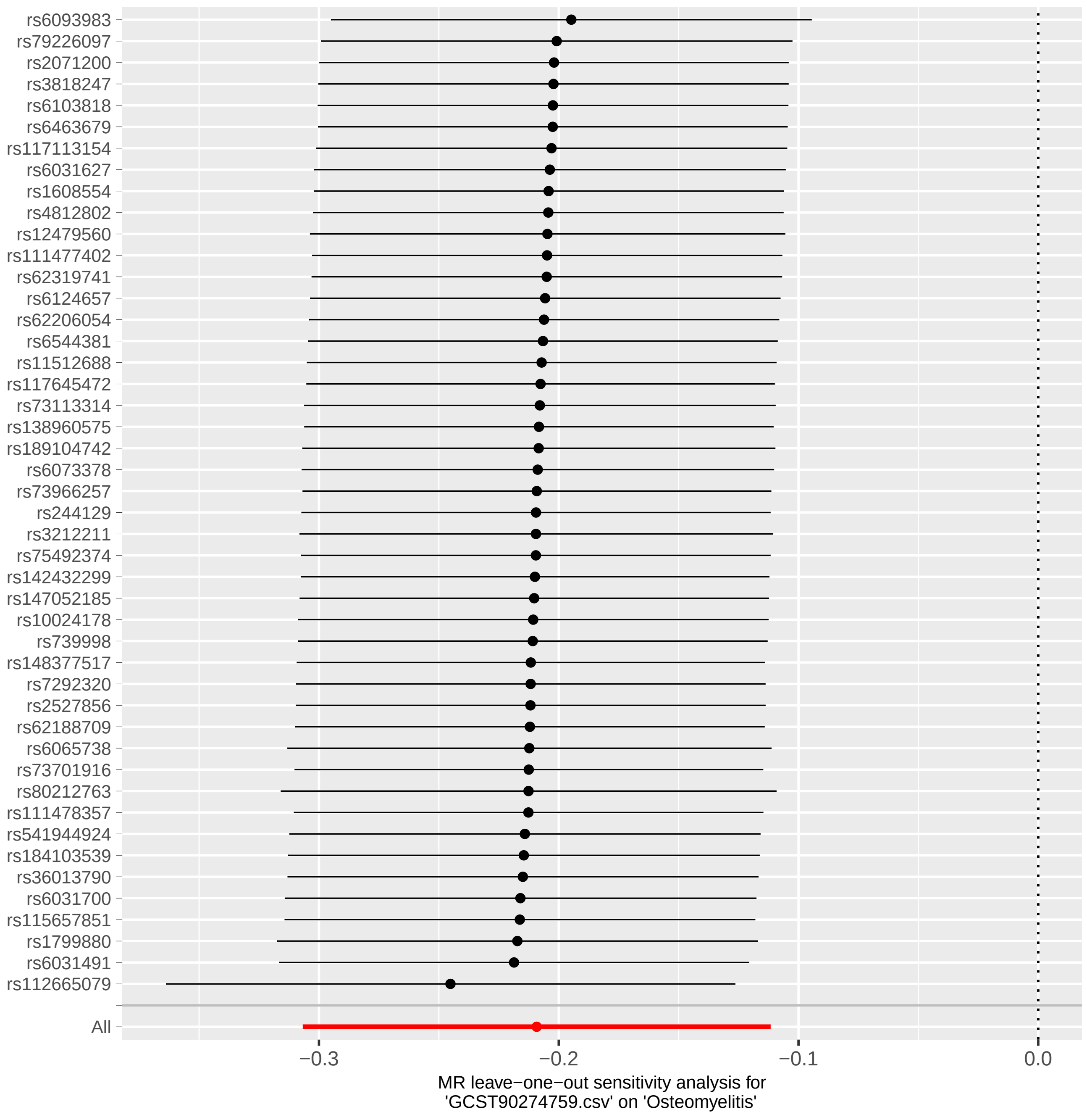
rs6093983
rs79226097
rs2071200
rs3818247
rs6103818
rs6463679
rs117113154
rs6031627
rs1608554
rs4812802
rs12479560
rs111477402
rs62319741
rs6124657
rs62206054
rs6544381
rs11512688
rs117645472
rs73113314
rs138960575
rs189104742
rs6073378
rs73966257
rs244129
rs3212211
rs75492374
rs142432299
rs147052185
rs10024178
rs739998
rs148377517
rs7292320
rs2527856
rs62188709
rs6065738
rs73701916
rs80212763
rs111478357
rs541944924
rs184103539
rs36013790
rs6031700
rs115657851
rs1799880
rs6031491
rs112665079
All
−0.3
−0.2
−0.1
0.0
MR leave−one−out sensitivity analysis for
'GCST90274759.csv' on 'Osteomyelitis'

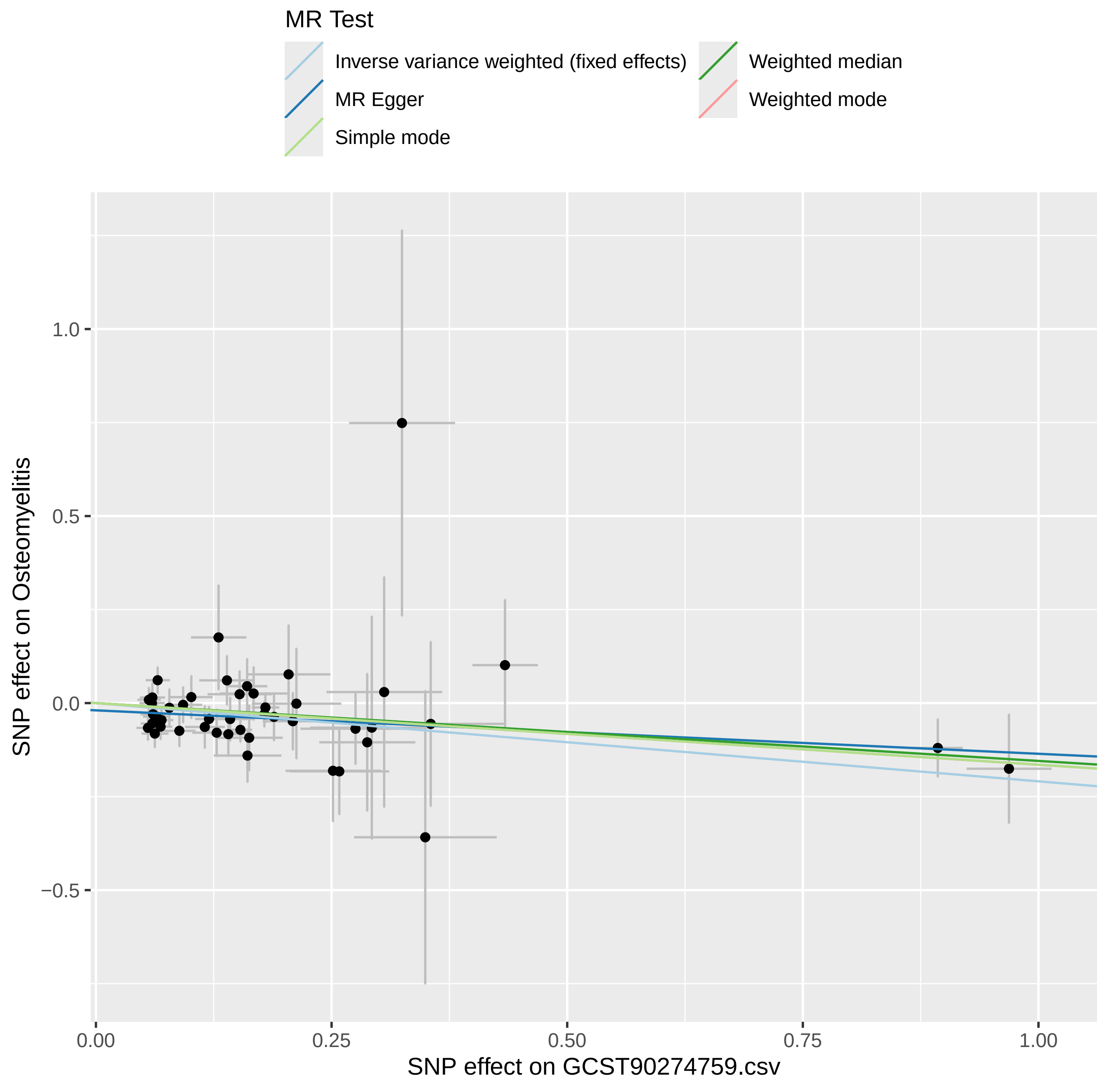
MR Test
Inverse variance weighted (fixed effects)
MR Egger
Simple mode
Weighted median
Weighted mode
SNP effect on Osteomyelitis
1.0
0.5
0.0
−0.5
0.00
0.25
0.50
0.75
1.00
SNP effect on GCST90274759.csv

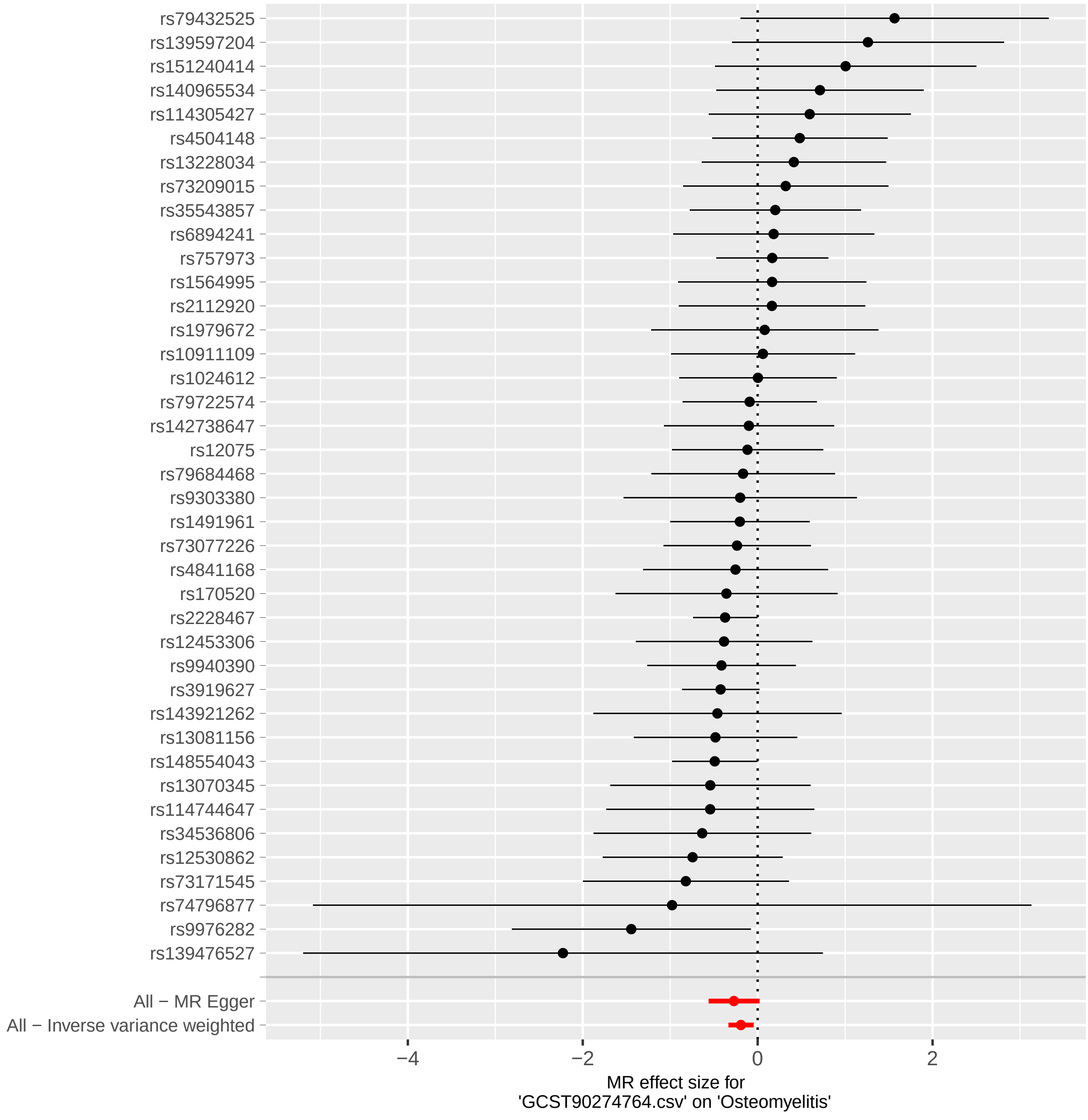
rs79432525
rs139597204
rs151240414
rs140965534
rs114305427
rs4504148
rs13228034
rs73209015
rs35543857
rs6894241
rs757973
rs1564995
rs2112920
rs1979672
rs10911109
rs1024612
rs79722574
rs142738647
rs12075
rs79684468
rs9303380
rs1491961
rs73077226
rs4841168
rs170520
rs2228467
rs12453306
rs9940390
rs3919627
rs143921262
rs13081156
rs148554043
rs13070345
rs114744647
rs34536806
rs12530862
rs73171545
rs74796877
rs9976282
rs139476527
All − MR Egger
All − Inverse variance weighted
−4
−2
0
2
MR effect size for
'GCST90274764.csv' on 'Osteomyelitis'

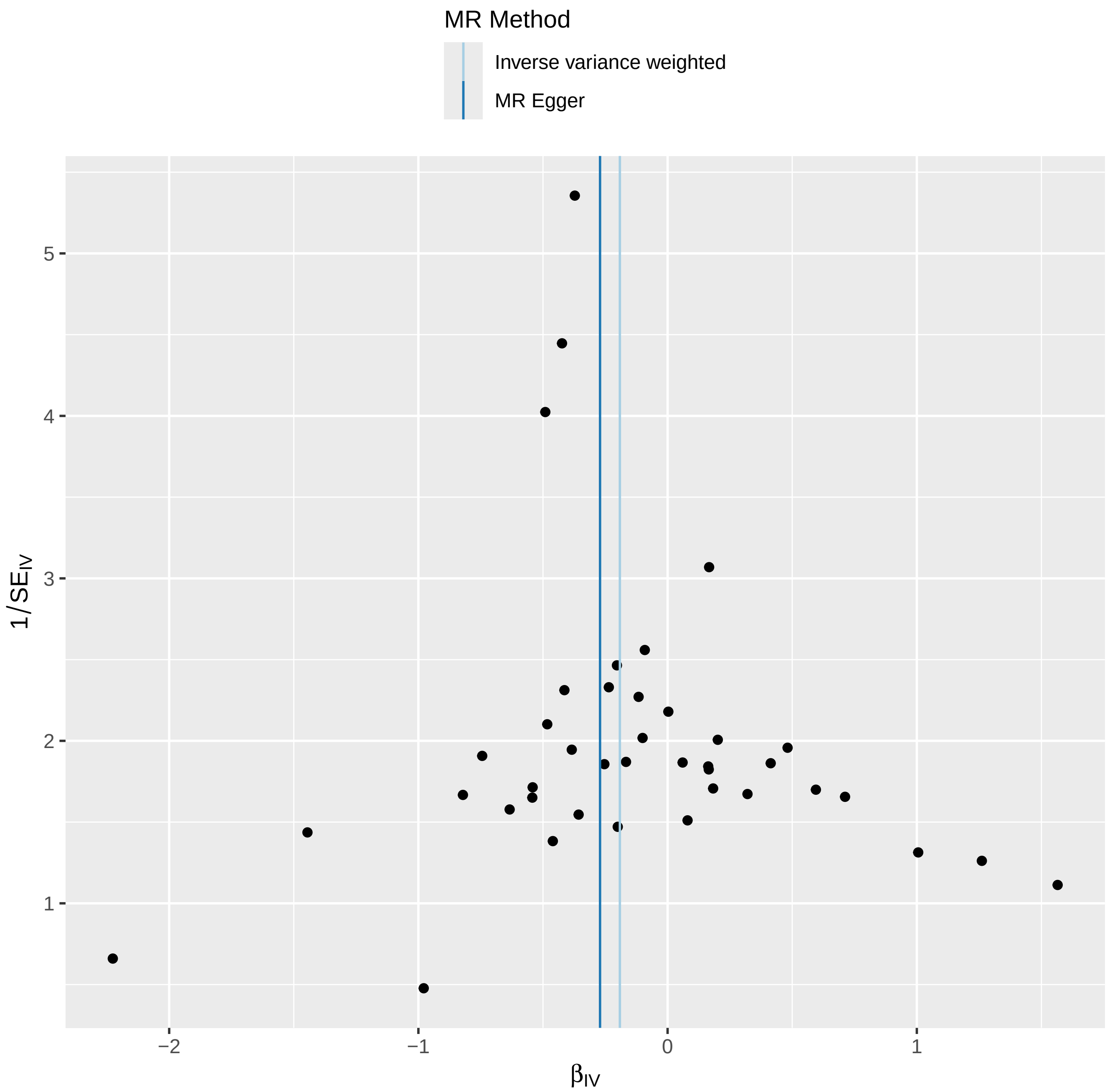
MR Method
Inverse variance weighted
MR Egger
1/SE_IV
β_IV
5
4
3
2
1
−2
−1
0
1

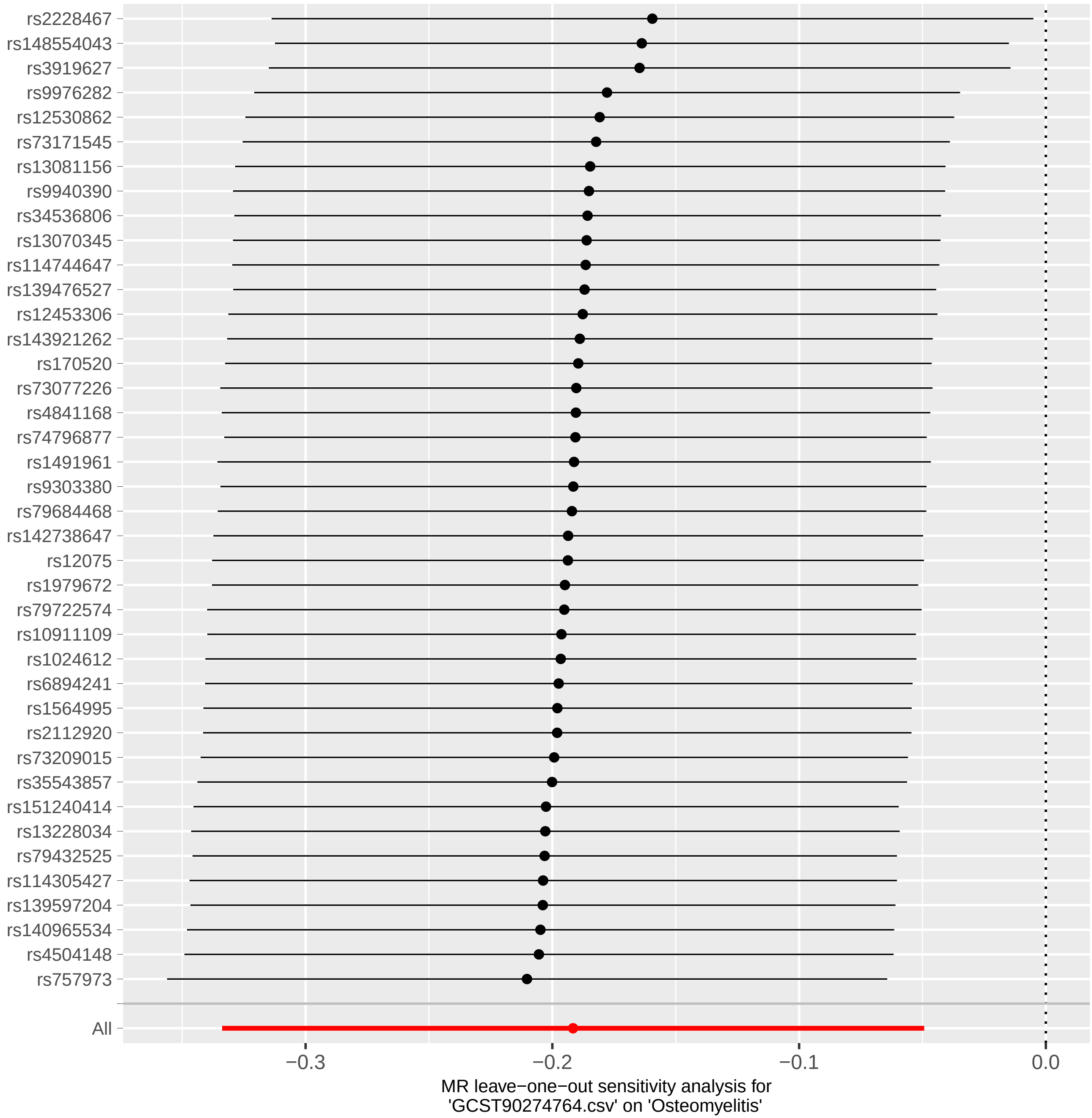

rs2228467
rs148554043
rs3919627
rs9976282
rs12530862
rs73171545
rs13081156
rs9940390
rs34536806
rs13070345
rs114744647
rs139476527
rs12453306
rs143921262
rs170520
rs73077226
rs4841168
rs74796877
rs1491961
rs9303380
rs79684468
rs142738647
rs12075
rs1979672
rs79722574
rs10911109
rs1024612
rs6894241
rs1564995
rs2112920
rs73209015
rs35543857
rs151240414
rs13228034
rs79432525
rs114305427
rs139597204
rs140965534
rs4504148
rs757973
All
−0.3
−0.2
−0.1
0.0
MR leave−one−out sensitivity analysis for
'GCST90274764.csv' on 'Osteomyelitis'

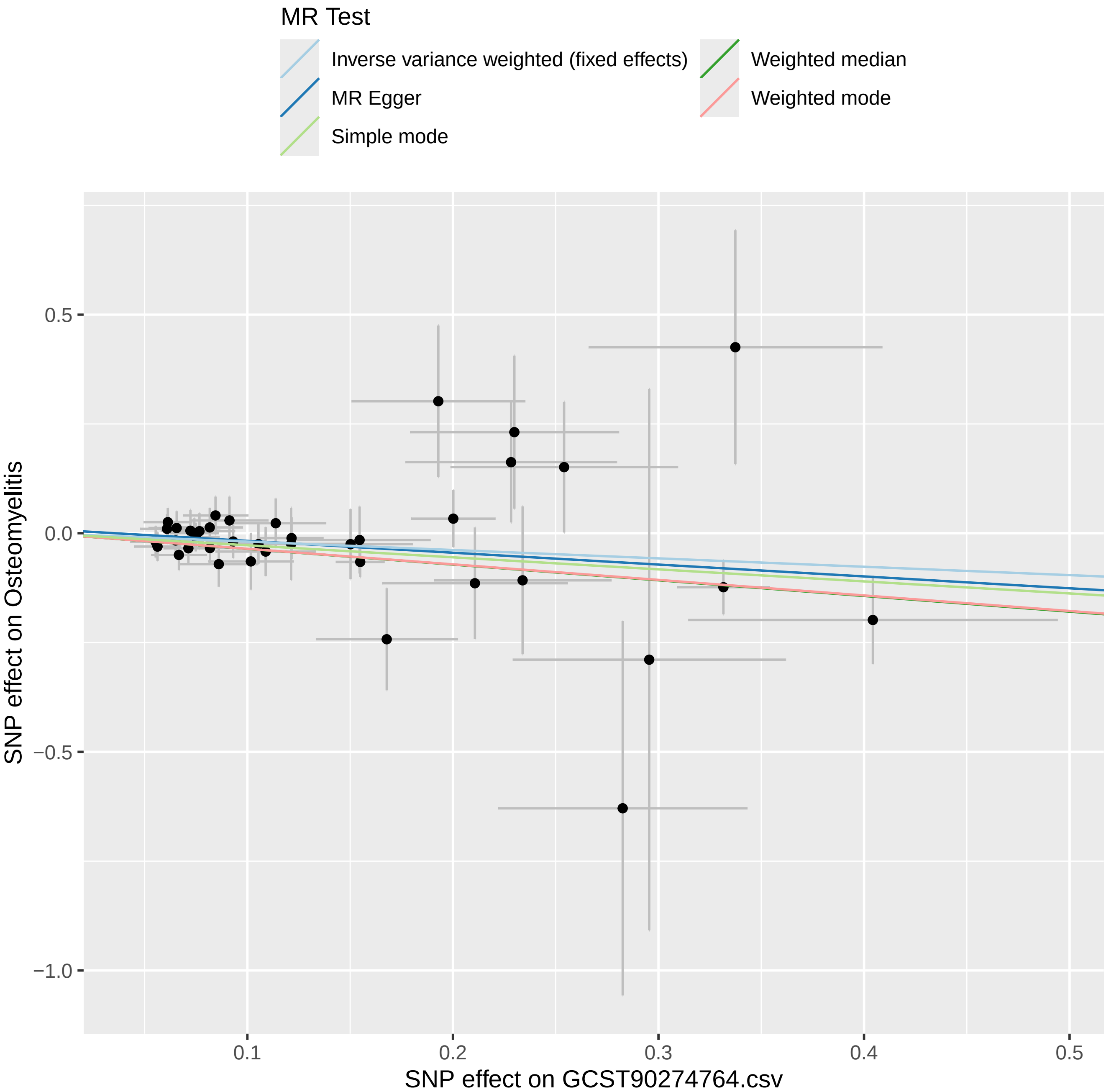
MR Test
Inverse variance weighted (fixed effects)
MR Egger
Simple mode
Weighted median
Weighted mode
0.5
0.0
−0.5
−1.0
0.1
0.2
0.3
0.4
0.5
SNP effect on Osteomyelitis
SNP effect on GCST90274764.csv

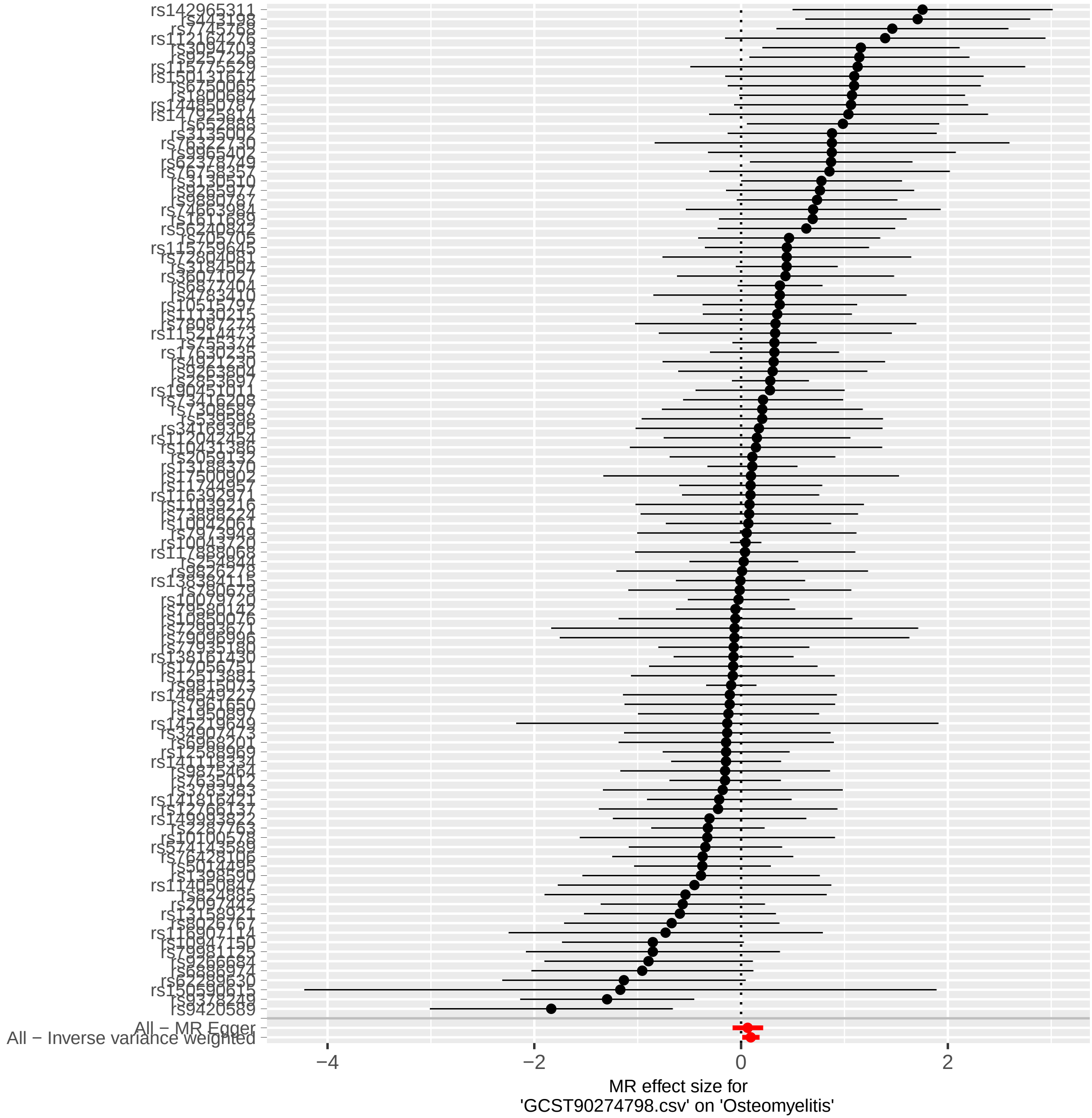
rs142965311
rs9420589
All − MR Egger
All − Inverse variance weighted
−4
−2
0
2
MR effect size for
'GCST90274798.csv' on 'Osteomyelitis'

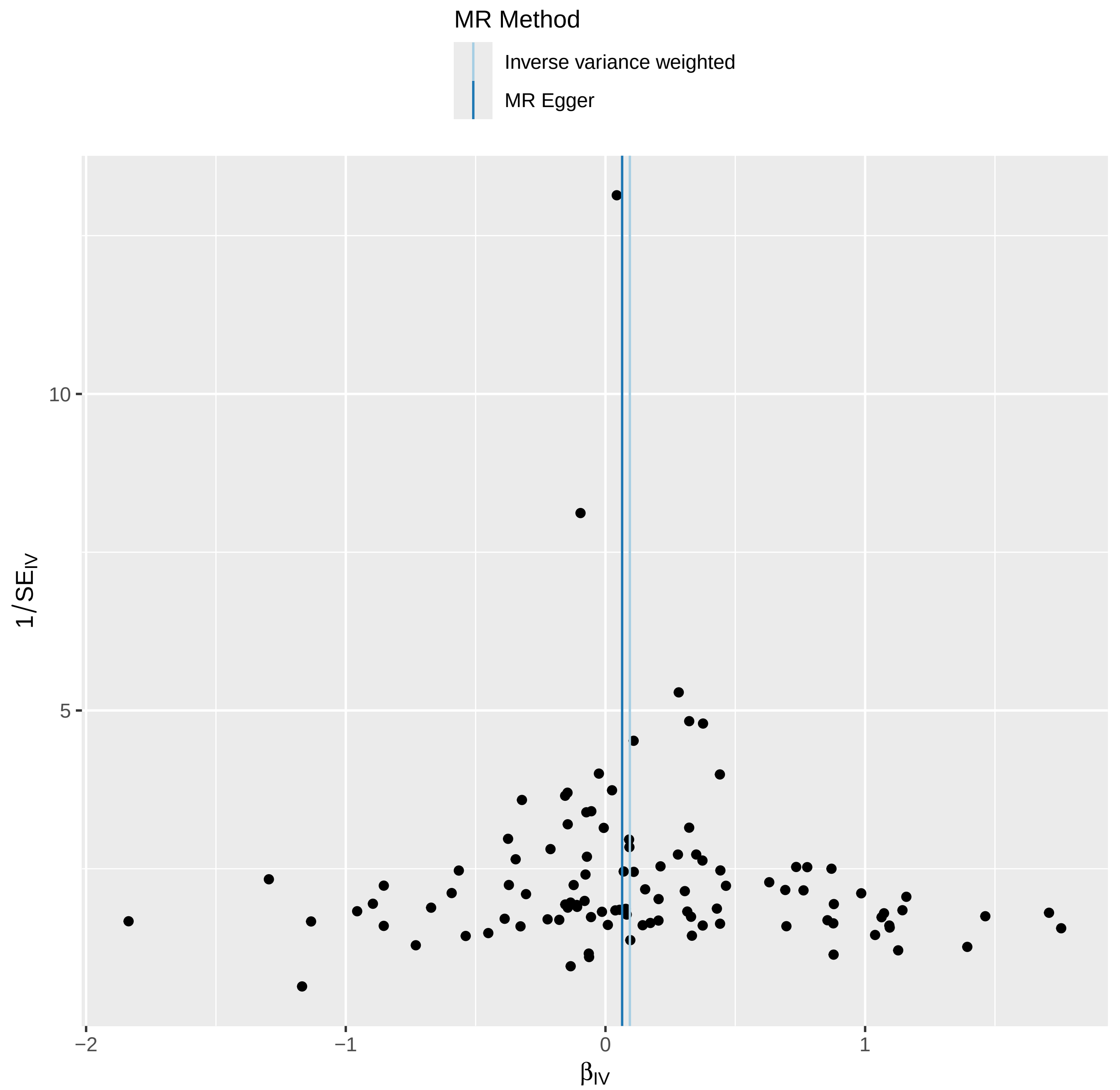

MR Method
Inverse variance weighted
MR Egger
1/SE$_{IV}$
10
5
−2
−1
0
1
β$_{IV}$

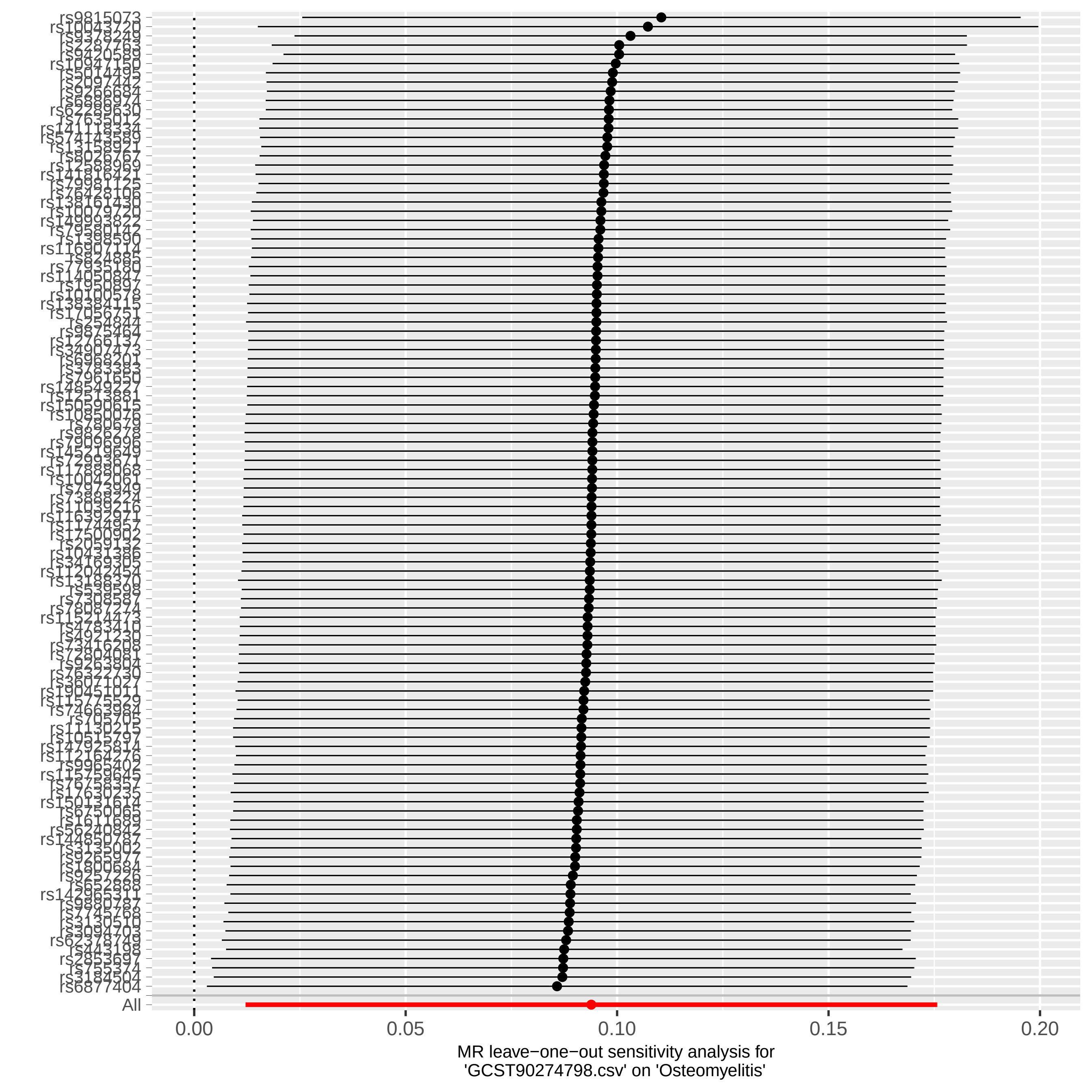
rs9815073
rs6877404
All
0.00
0.05
0.10
0.15
0.20
MR leave−one−out sensitivity analysis for
'GCST90274798.csv' on 'Osteomyelitis'

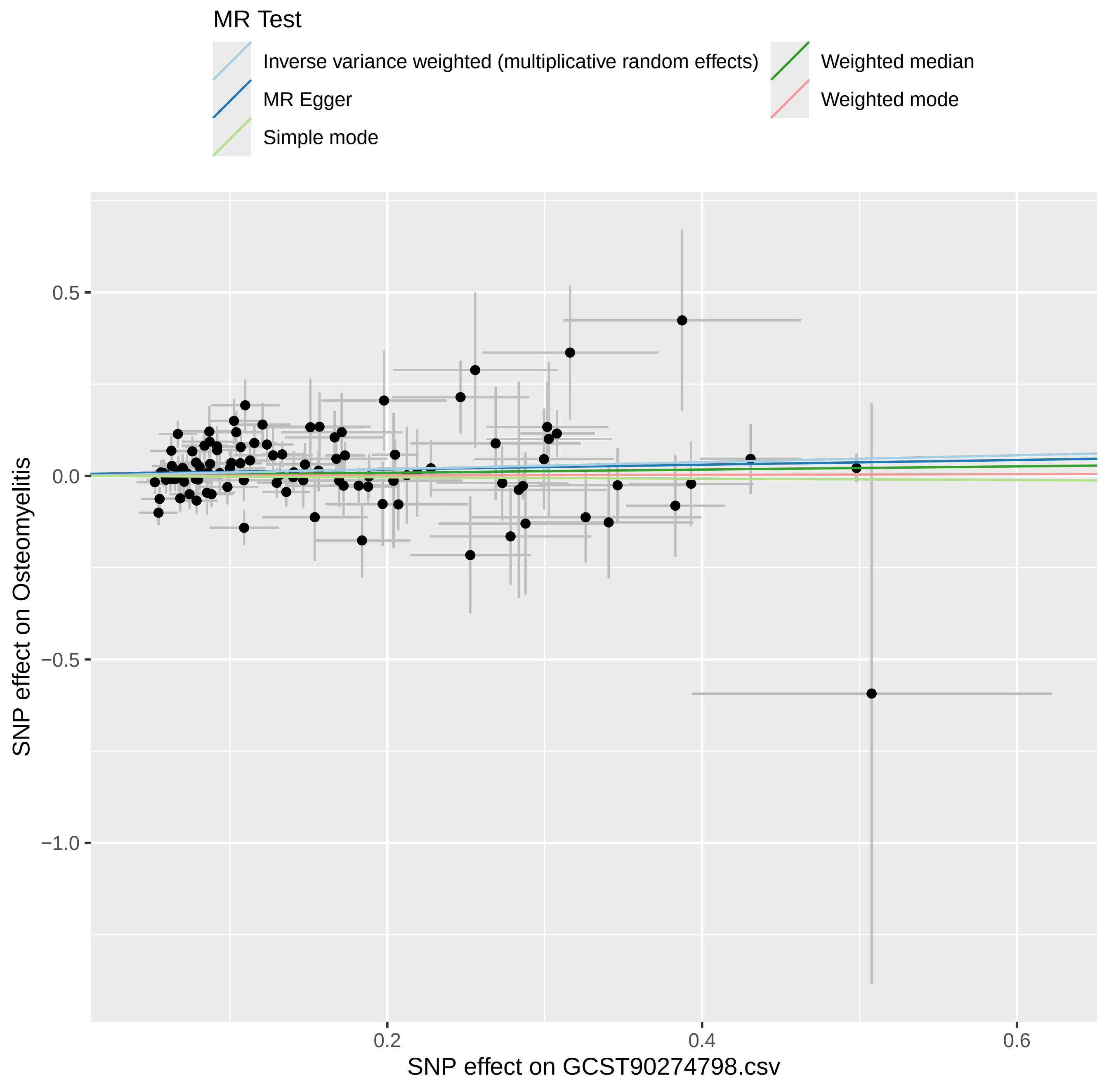

MR Test
Inverse variance weighted (multiplicative random effects)
MR Egger
Simple mode
Weighted median
Weighted mode
0.5
0.0
−0.5
−1.0
0.2
0.4
0.6
SNP effect on Osteomyelitis
SNP effect on GCST90274798.csv

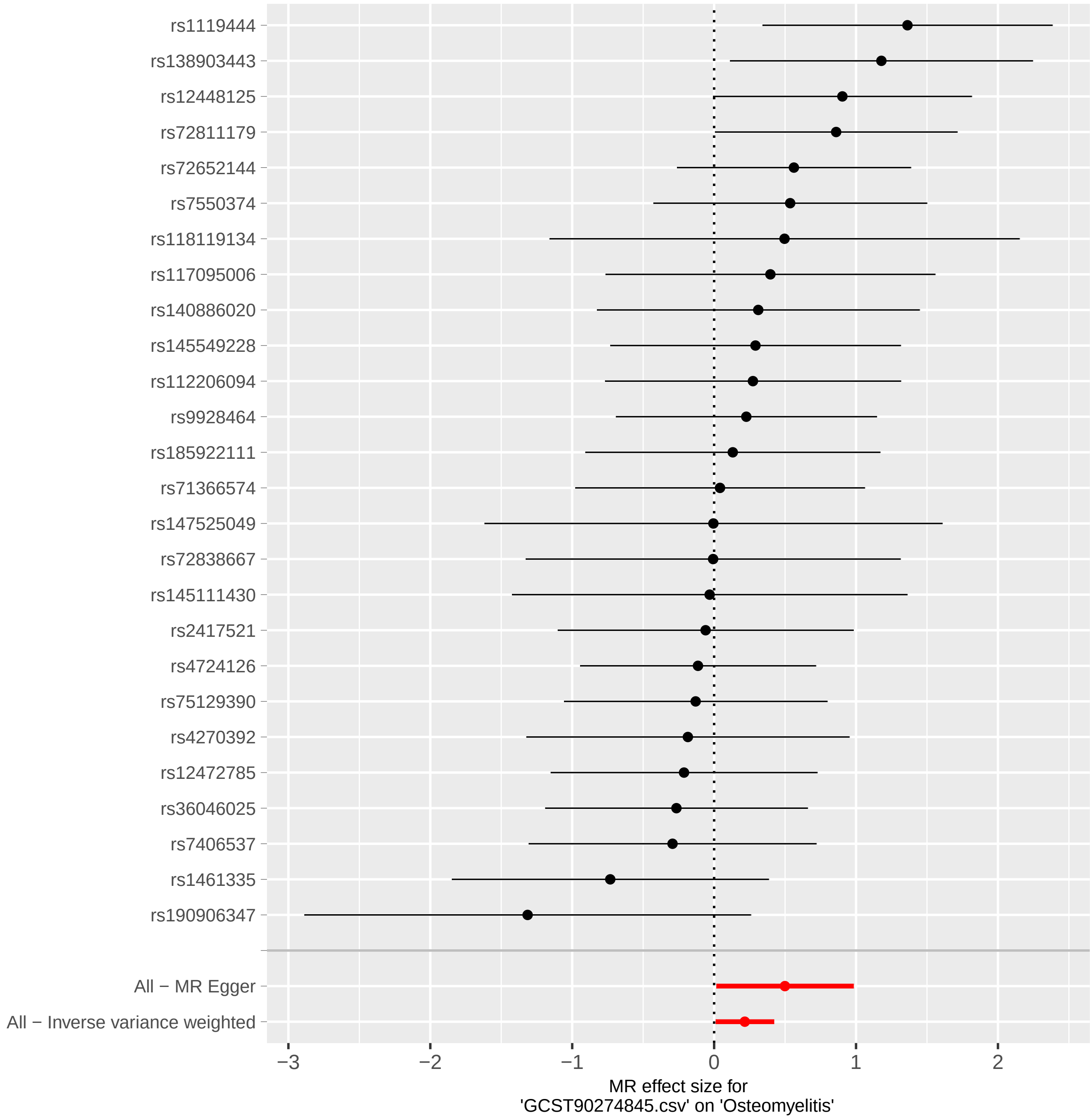
rs1119444
rs138903443
rs12448125
rs72811179
rs72652144
rs7550374
rs118119134
rs117095006
rs140886020
rs145549228
rs112206094
rs9928464
rs185922111
rs71366574
rs147525049
rs72838667
rs145111430
rs2417521
rs4724126
rs75129390
rs4270392
rs12472785
rs36046025
rs7406537
rs1461335
rs190906347
All − MR Egger
All − Inverse variance weighted
−3
−2
−1
0
1
2
MR effect size for
'GCST90274845.csv' on 'Osteomyelitis'

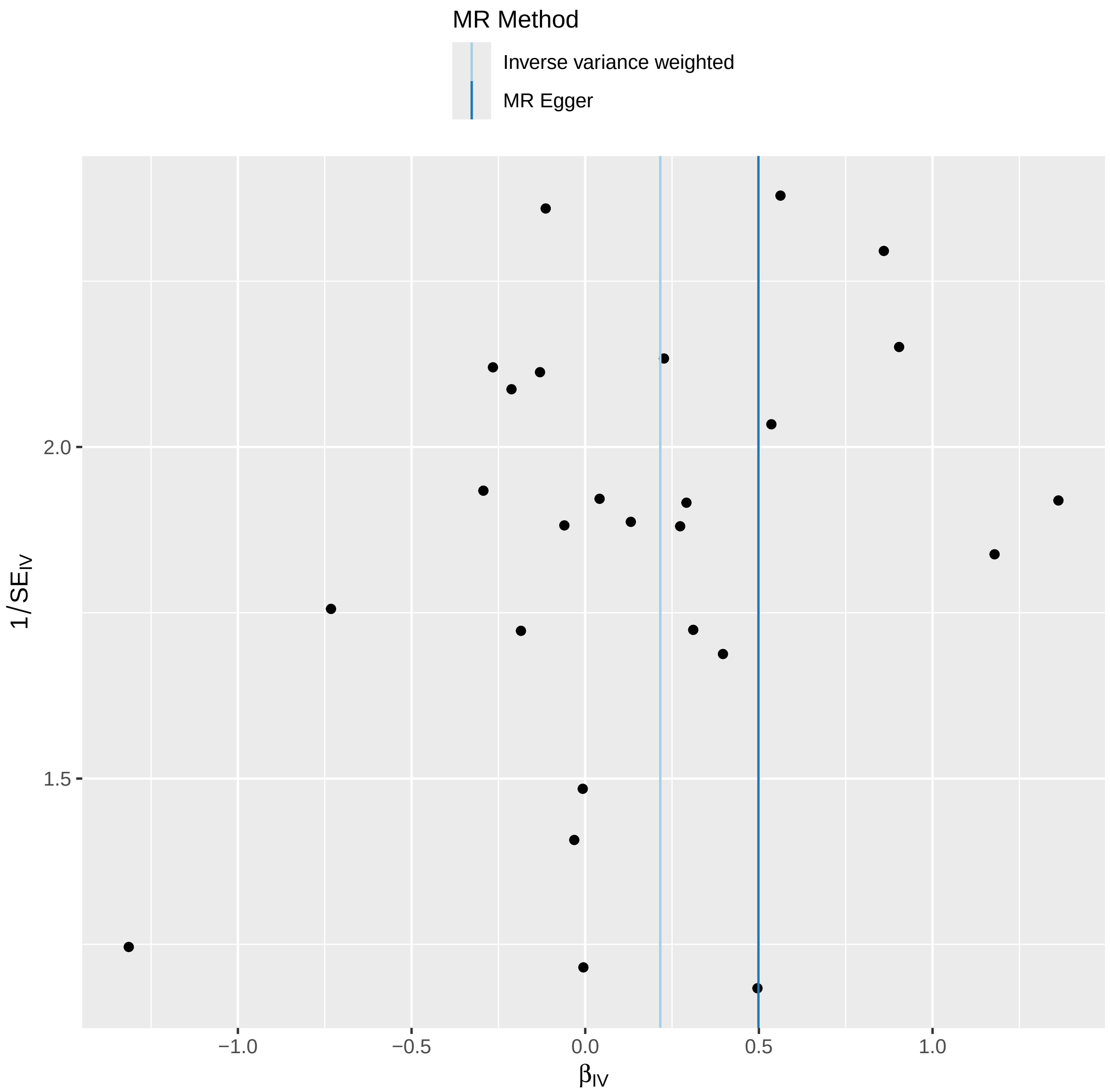
MR Method
Inverse variance weighted
MR Egger
2.0
1.5
−1.0
−0.5
0.0
0.5
1.0
β_{IV}
$1/SE_{IV}$

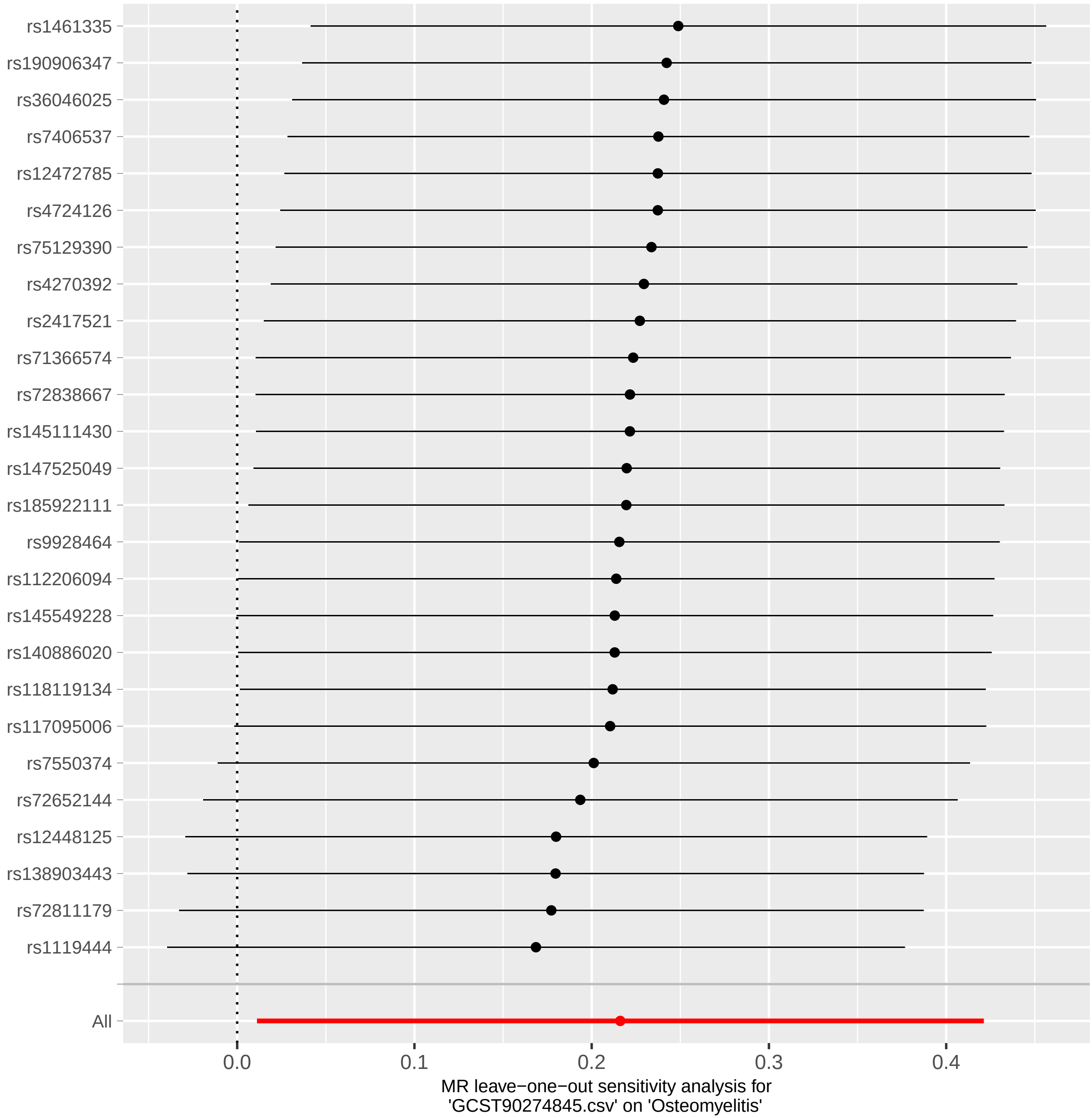
rs1461335
rs190906347
rs36046025
rs7406537
rs12472785
rs4724126
rs75129390
rs4270392
rs2417521
rs71366574
rs72838667
rs145111430
rs147525049
rs185922111
rs9928464
rs112206094
rs145549228
rs140886020
rs118119134
rs117095006
rs7550374
rs72652144
rs12448125
rs138903443
rs72811179
rs1119444
All
0.0
0.1
0.2
0.3
0.4
MR leave−one−out sensitivity analysis for
'GCST90274845.csv' on 'Osteomyelitis'

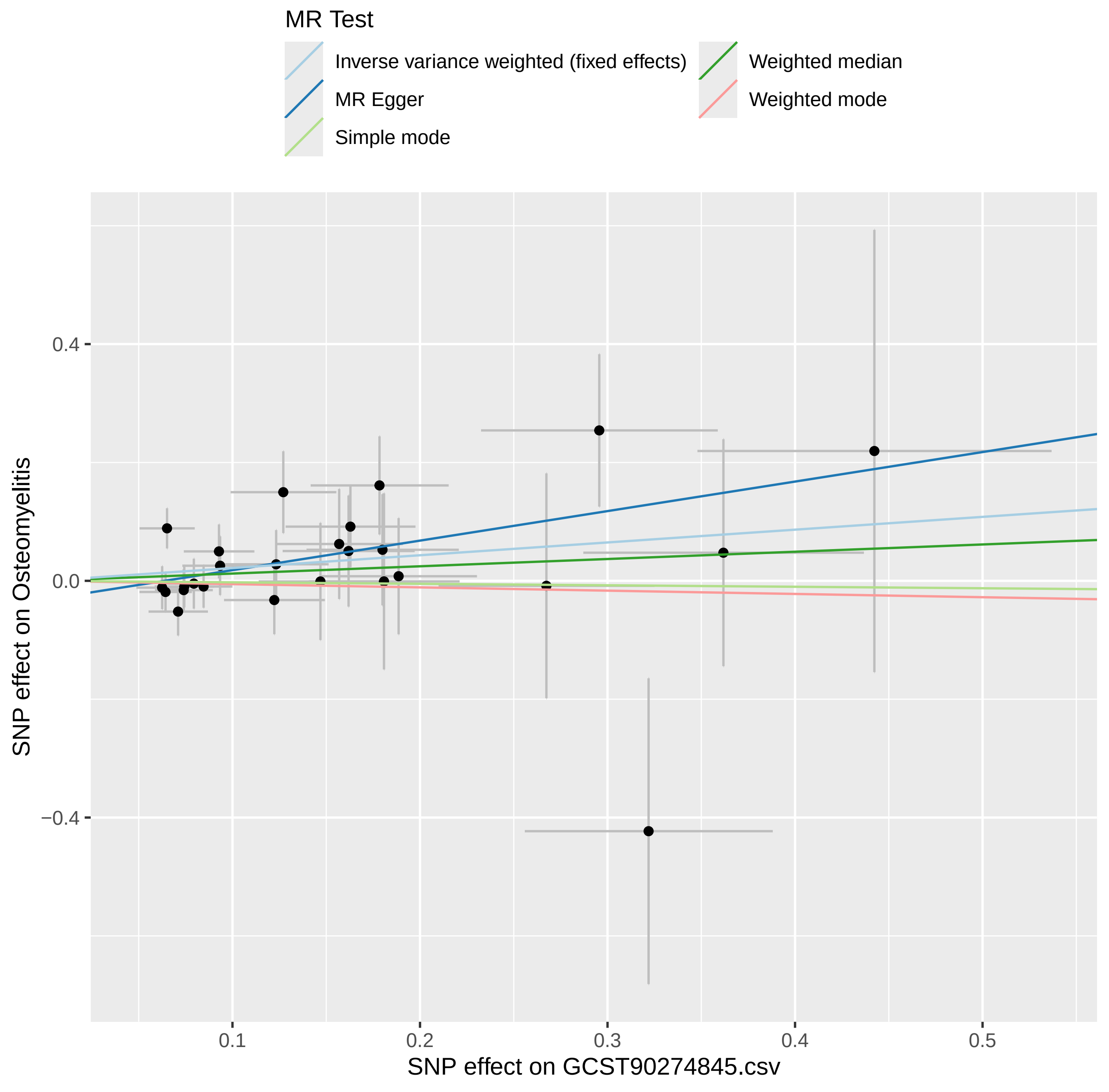
MR Test
Inverse variance weighted (fixed effects)
MR Egger
Simple mode
Weighted median
Weighted mode
SNP effect on Osteomyelitis
0.4
0.0
−0.4
0.1
0.2
0.3
0.4
0.5
SNP effect on GCST90274845.csv

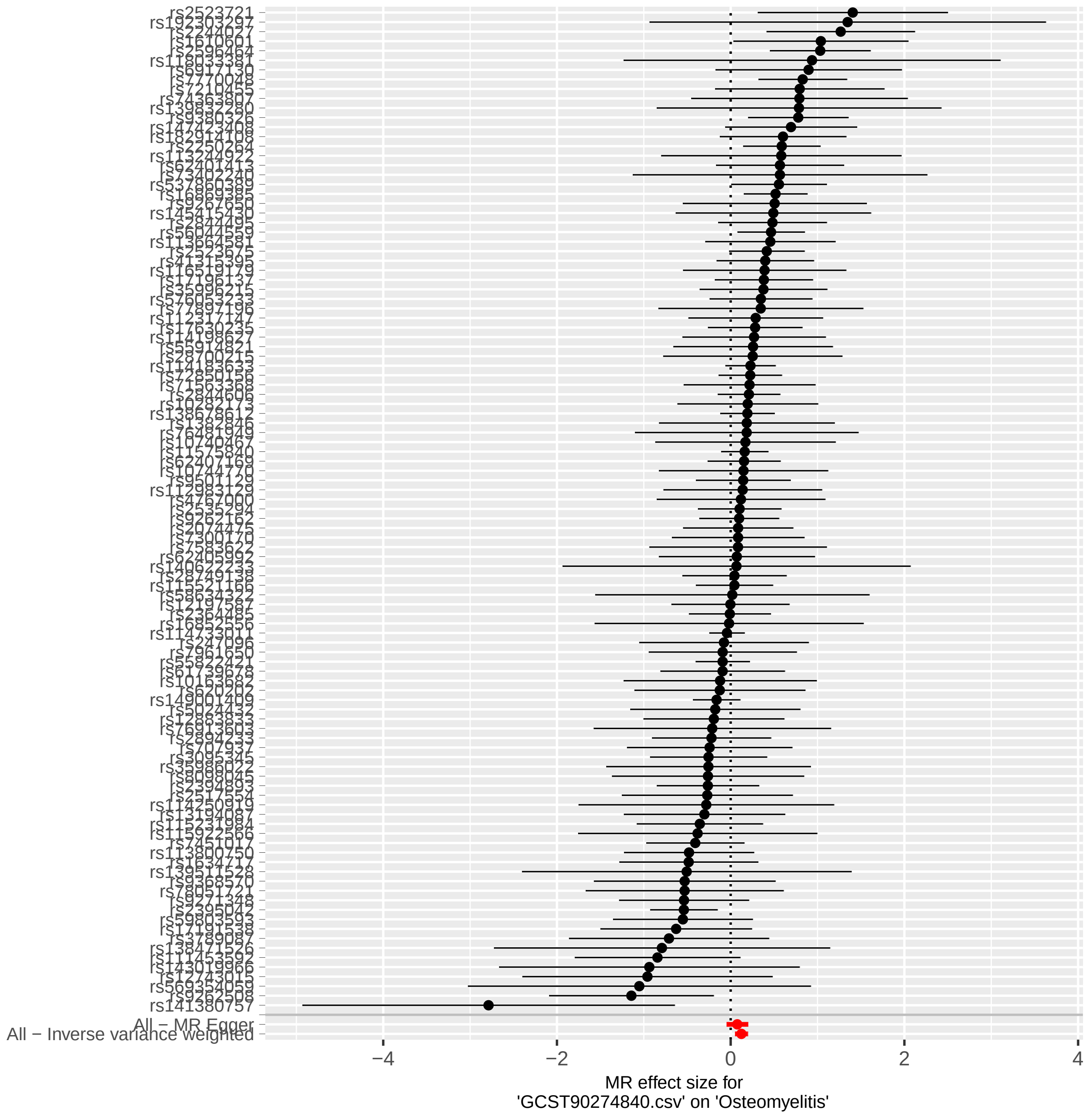

rs2523721
rs192303297
rs141380757
All − MR Egger
All − Inverse variance weighted
−4
−2
0
2
4
MR effect size for
'GCST90274840.csv' on 'Osteomyelitis'

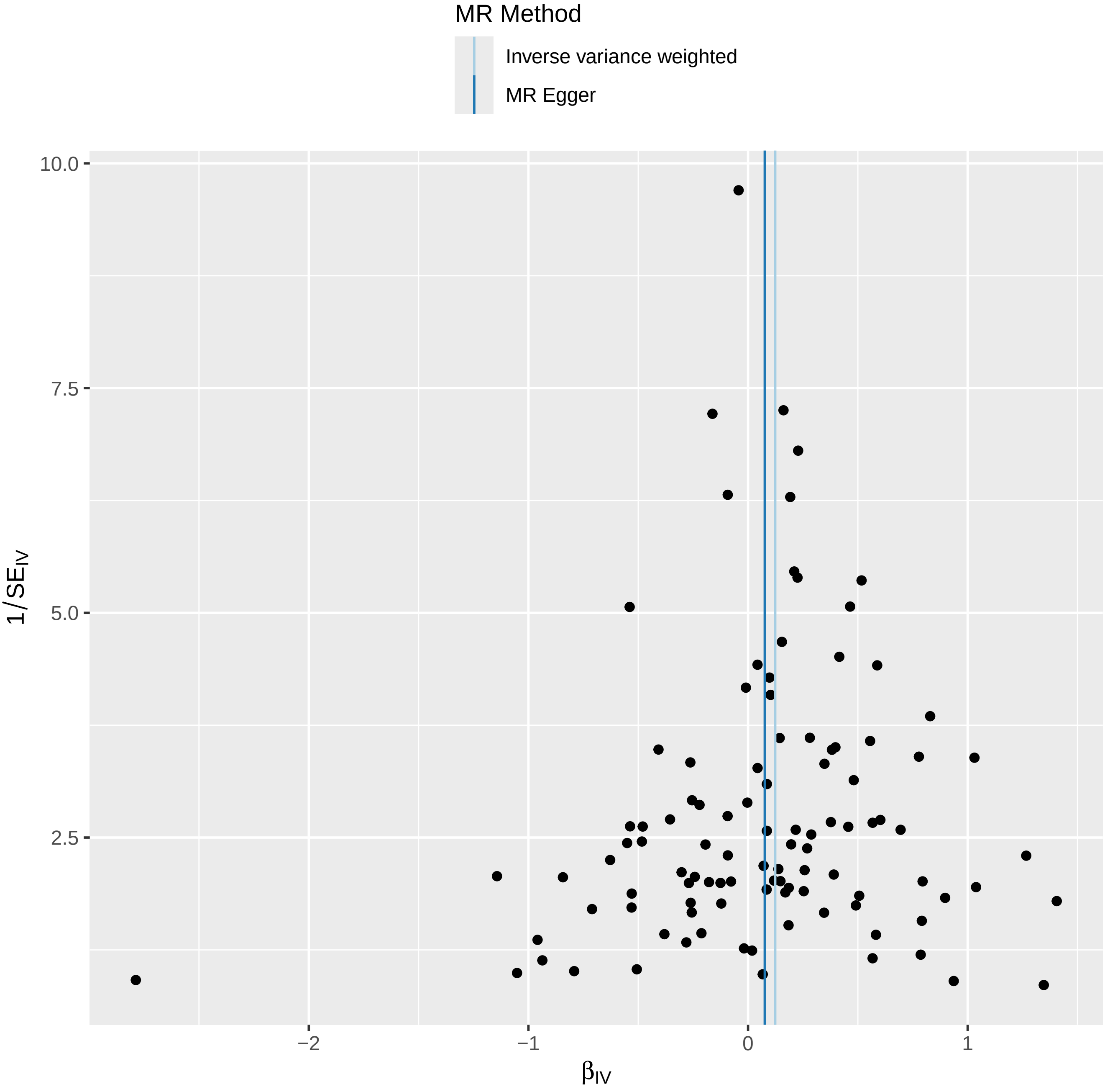
MR Method
Inverse variance weighted
MR Egger
10.0
7.5
5.0
2.5
1/SE_IV
−2
−1
0
1
β_IV

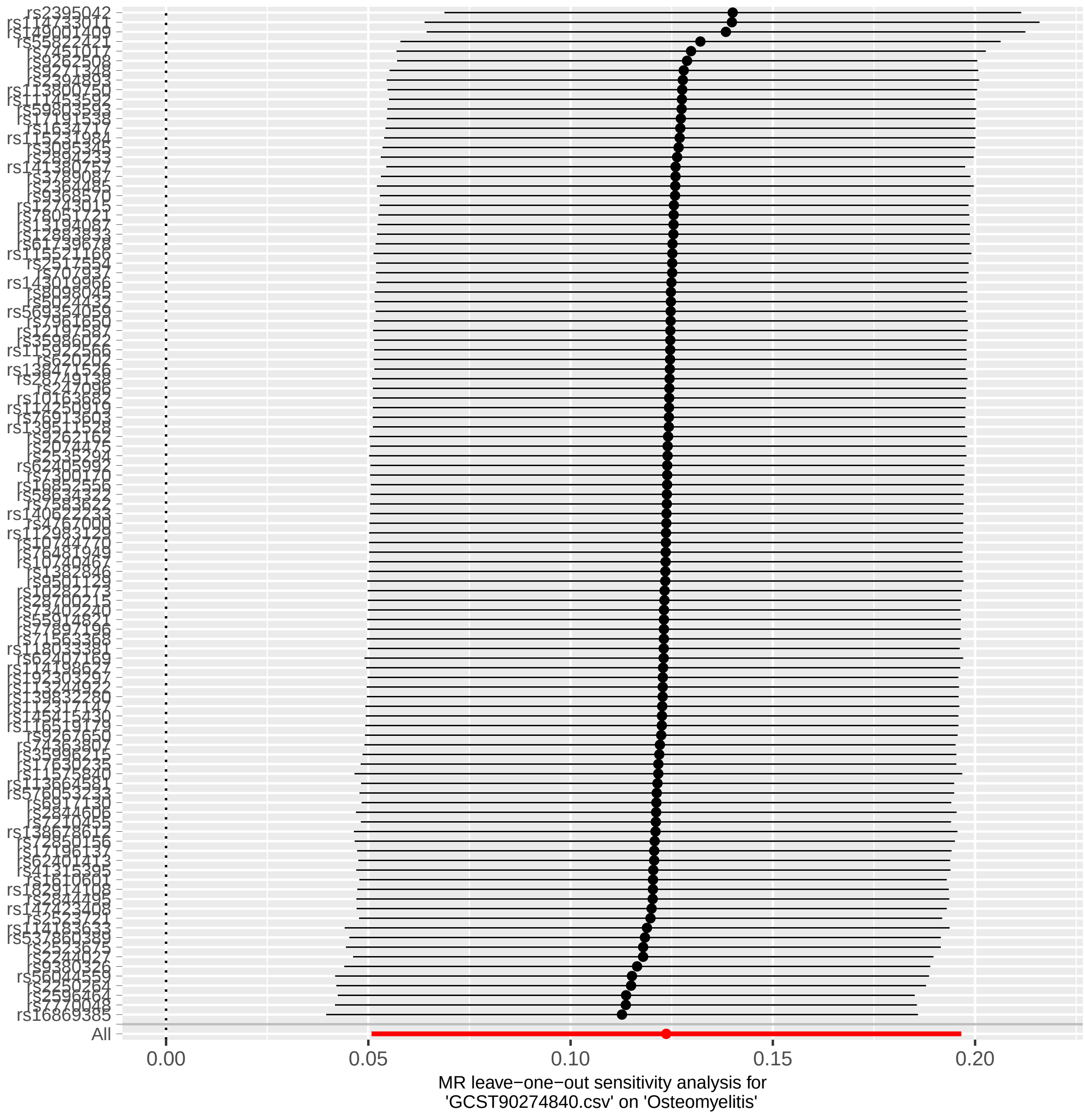
rs2395042
rs16869385
All
0.00
0.05
0.10
0.15
0.20
MR leave−one−out sensitivity analysis for
'GCST90274840.csv' on 'Osteomyelitis'

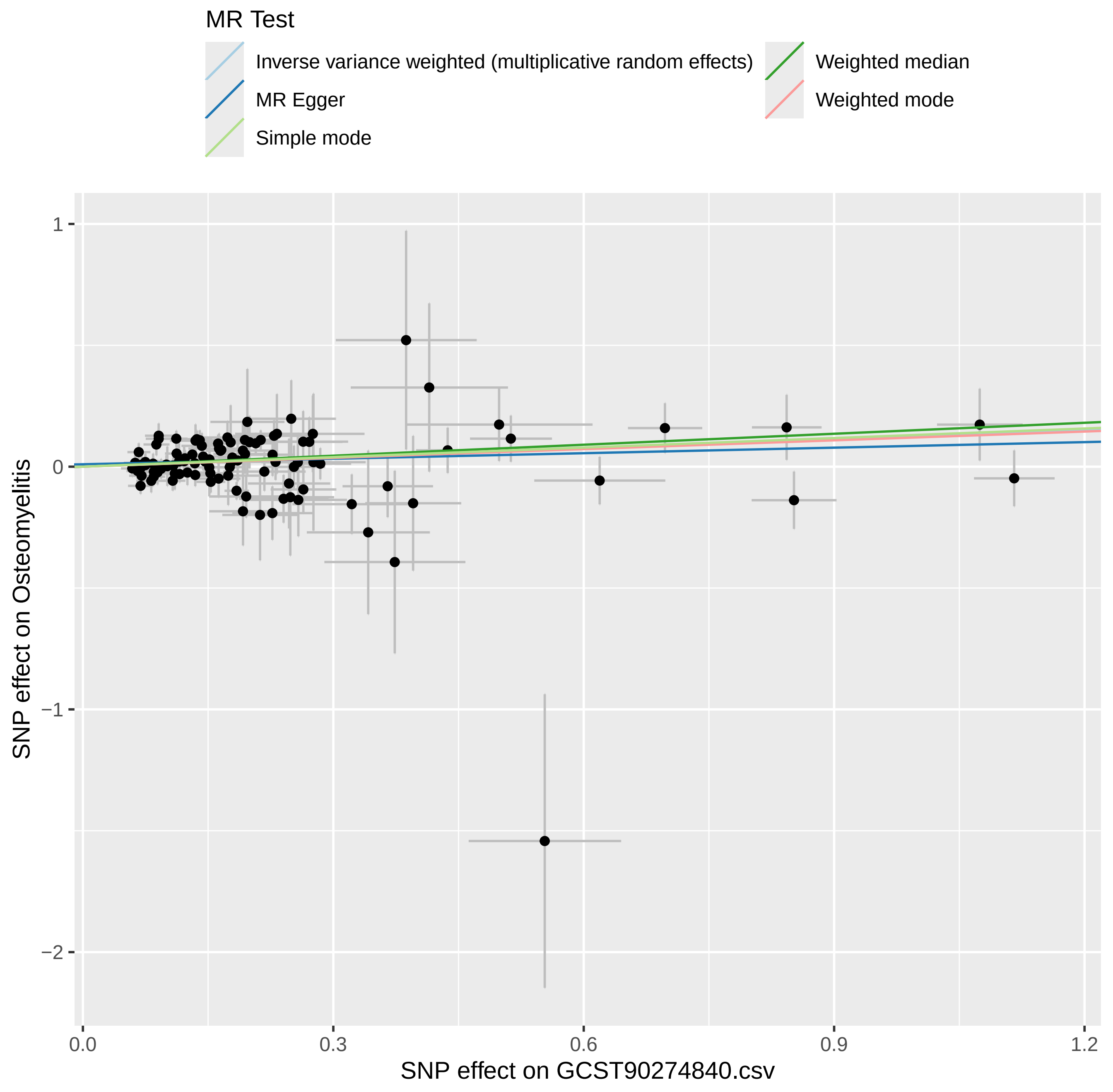

MR Test
Inverse variance weighted (multiplicative random effects)
MR Egger
Simple mode
Weighted median
Weighted mode
1
0
−1
−2
SNP effect on Osteomyelitis
0.0
0.3
0.6
0.9
1.2
SNP effect on GCST90274840.csv

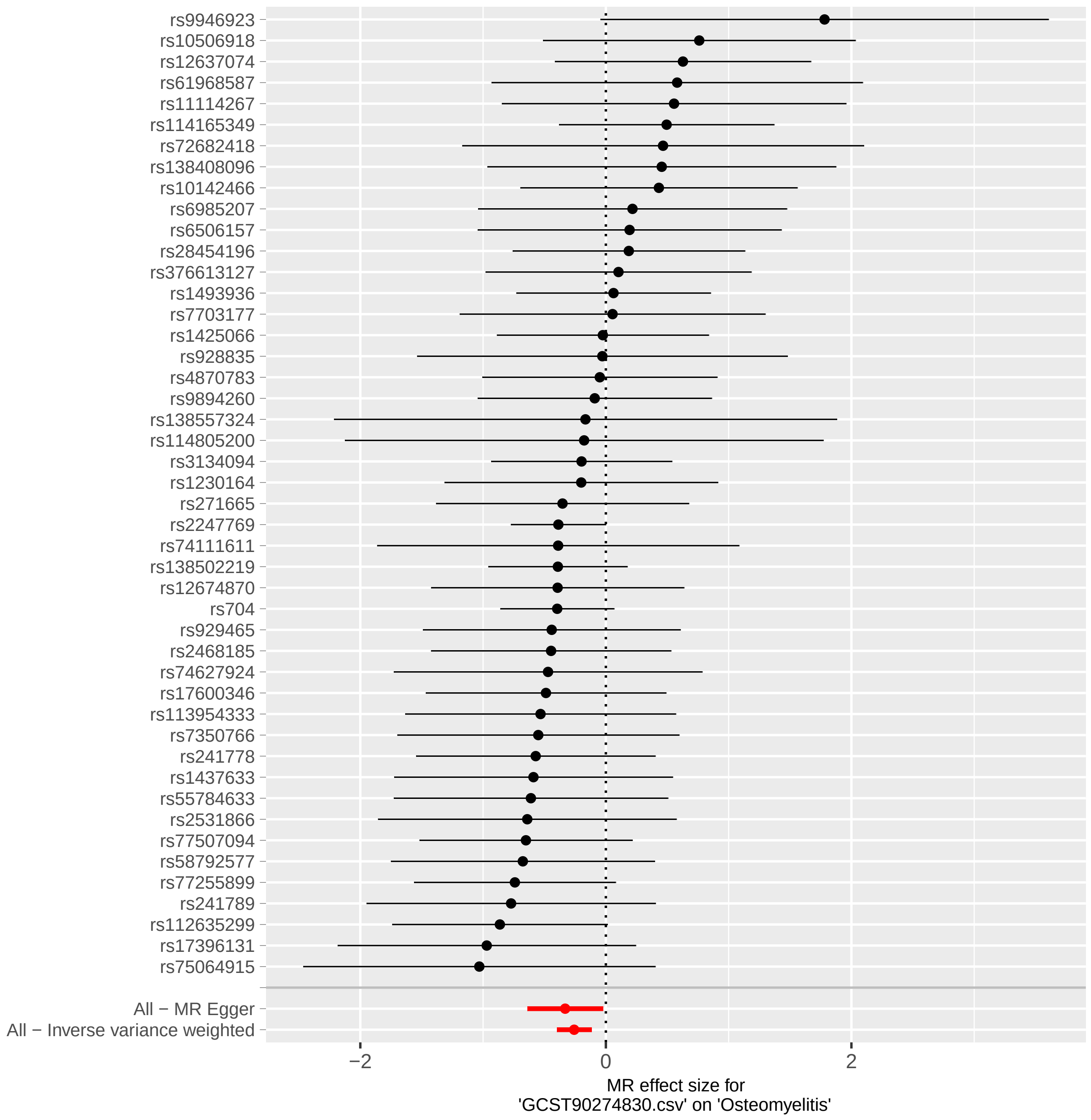
rs9946923
rs10506918
rs12637074
rs61968587
rs11114267
rs114165349
rs72682418
rs138408096
rs10142466
rs6985207
rs6506157
rs28454196
rs376613127
rs1493936
rs7703177
rs1425066
rs928835
rs4870783
rs9894260
rs138557324
rs114805200
rs3134094
rs1230164
rs271665
rs2247769
rs74111611
rs138502219
rs12674870
rs704
rs929465
rs2468185
rs74627924
rs17600346
rs113954333
rs7350766
rs241778
rs1437633
rs55784633
rs2531866
rs77507094
rs58792577
rs77255899
rs241789
rs112635299
rs17396131
rs75064915
All − MR Egger
All − Inverse variance weighted
−2
0
2
MR effect size for
'GCST90274830.csv' on 'Osteomyelitis'

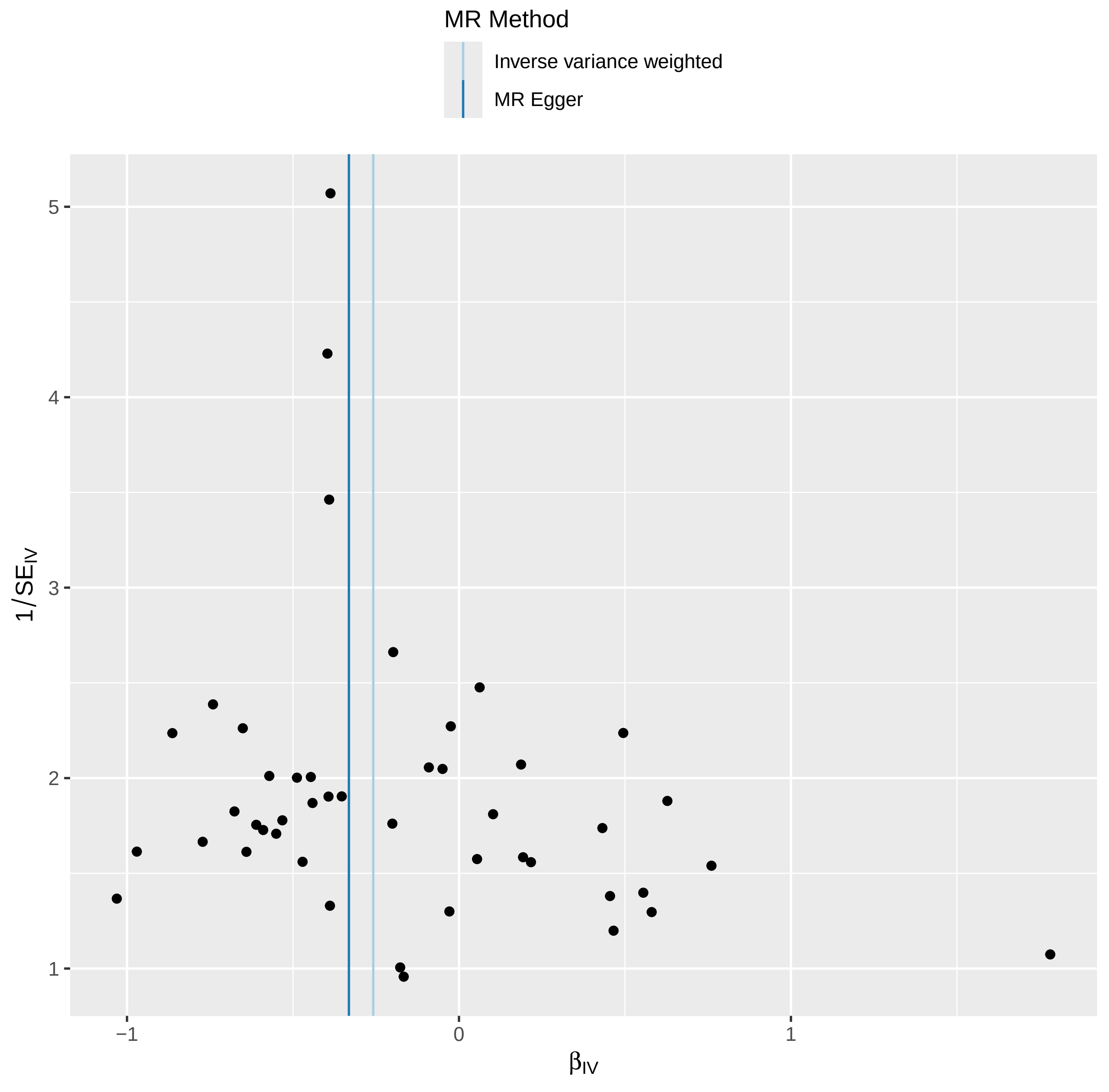
MR Method
Inverse variance weighted
MR Egger
1/SE_IV
β_IV
5
4
3
2
1
−1
0
1

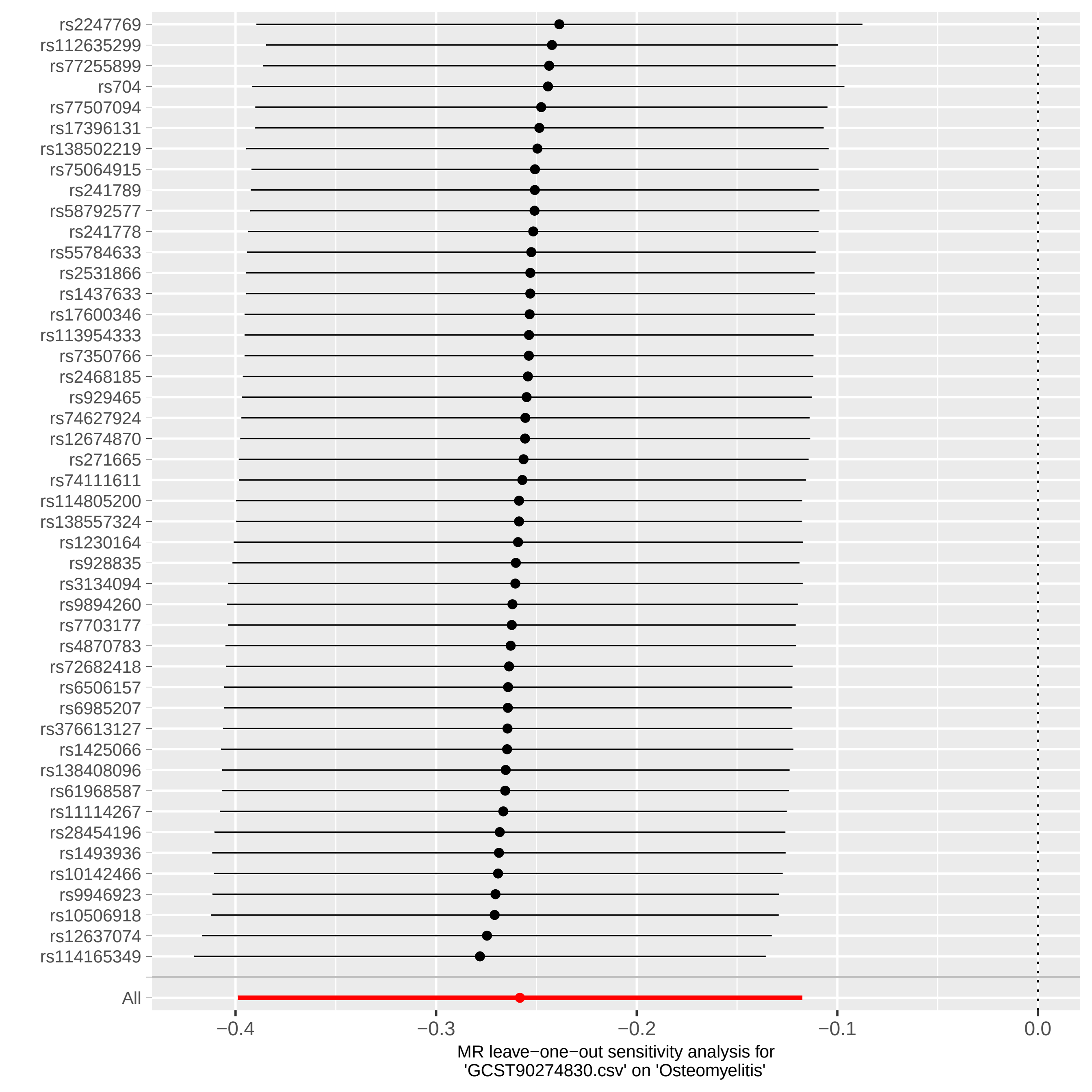
rs2247769
rs112635299
rs77255899
rs704
rs77507094
rs17396131
rs138502219
rs75064915
rs241789
rs58792577
rs241778
rs55784633
rs2531866
rs1437633
rs17600346
rs113954333
rs7350766
rs2468185
rs929465
rs74627924
rs12674870
rs271665
rs74111611
rs114805200
rs138557324
rs1230164
rs928835
rs3134094
rs9894260
rs7703177
rs4870783
rs72682418
rs6506157
rs6985207
rs376613127
rs1425066
rs138408096
rs61968587
rs11114267
rs28454196
rs1493936
rs10142466
rs9946923
rs10506918
rs12637074
rs114165349
All
−0.4
−0.3
−0.2
−0.1
0.0
MR leave−one−out sensitivity analysis for
'GCST90274830.csv' on 'Osteomyelitis'

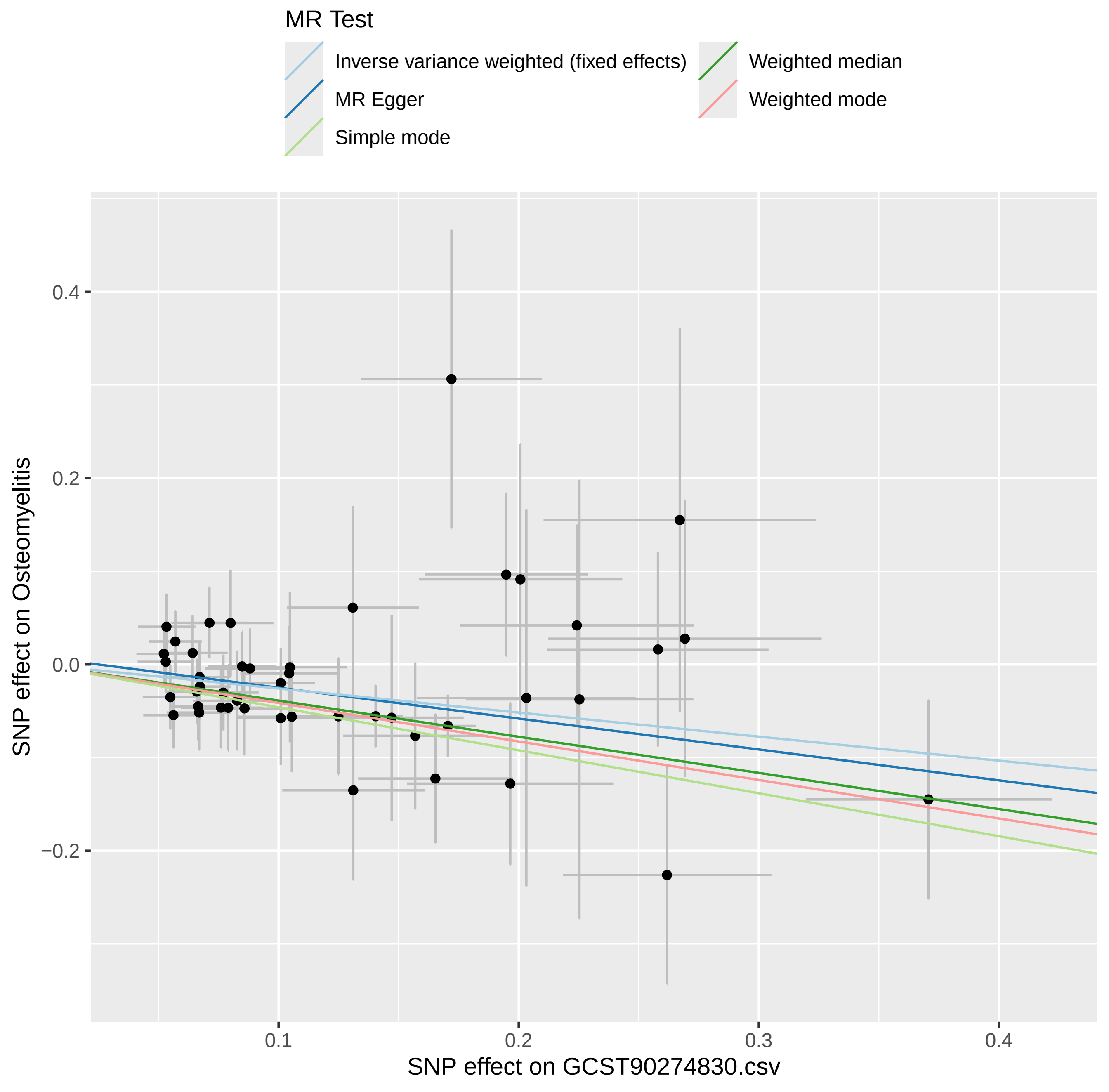
MR Test
Inverse variance weighted (fixed effects)
MR Egger
Simple mode
Weighted median
Weighted mode
SNP effect on Osteomyelitis
0.4
0.2
0.0
−0.2
0.1
0.2
0.3
0.4
SNP effect on GCST90274830.csv

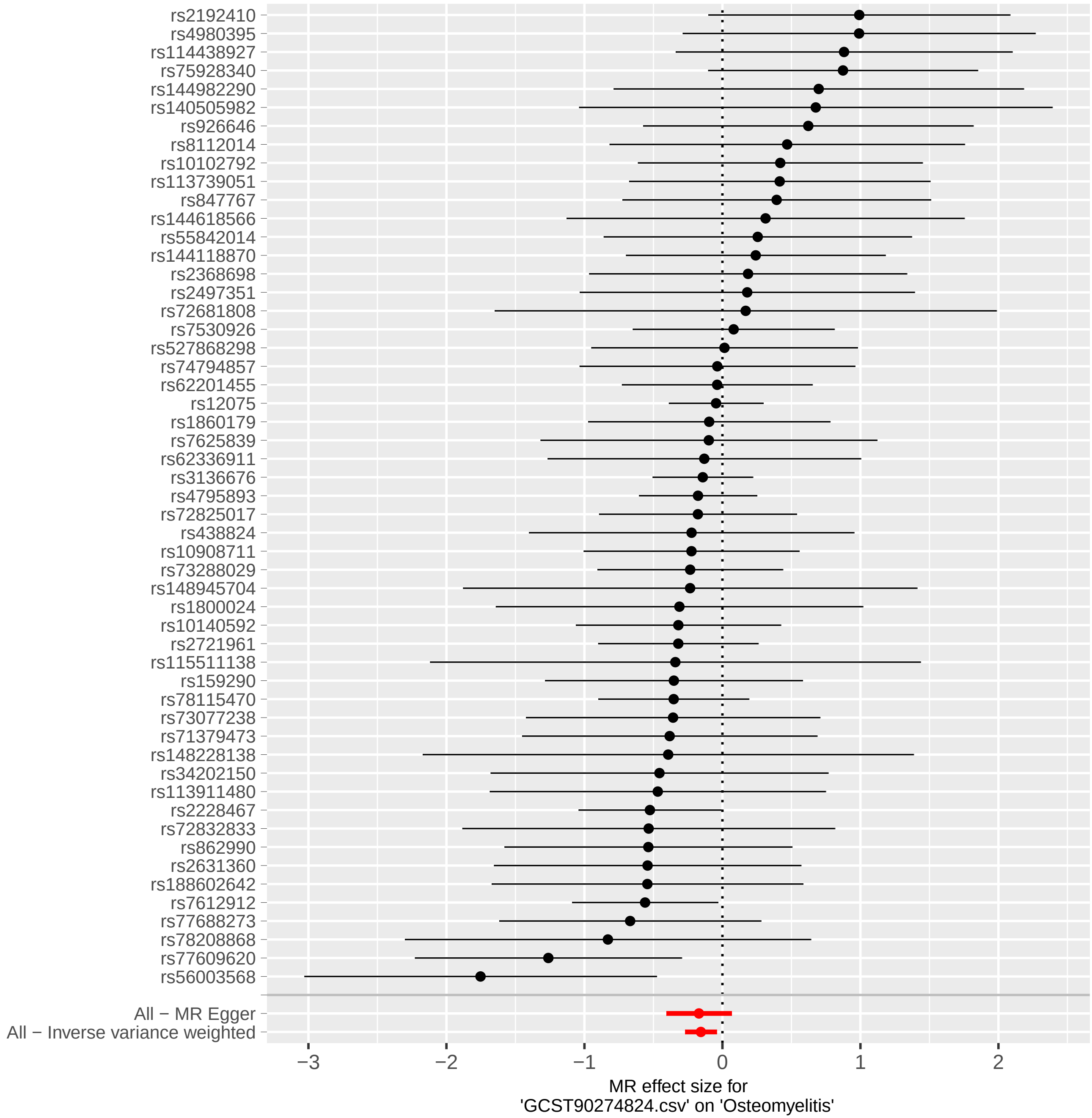
rs2192410
rs4980395
rs114438927
rs75928340
rs144982290
rs140505982
rs926646
rs8112014
rs10102792
rs113739051
rs847767
rs144618566
rs55842014
rs144118870
rs2368698
rs2497351
rs72681808
rs7530926
rs527868298
rs74794857
rs62201455
rs12075
rs1860179
rs7625839
rs62336911
rs3136676
rs4795893
rs72825017
rs438824
rs10908711
rs73288029
rs148945704
rs1800024
rs10140592
rs2721961
rs115511138
rs159290
rs78115470
rs73077238
rs71379473
rs148228138
rs34202150
rs113911480
rs2228467
rs72832833
rs862990
rs2631360
rs188602642
rs7612912
rs77688273
rs78208868
rs77609620
rs56003568
All − MR Egger
All − Inverse variance weighted
−3
−2
−1
0
1
2
MR effect size for
'GCST90274824.csv' on 'Osteomyelitis'

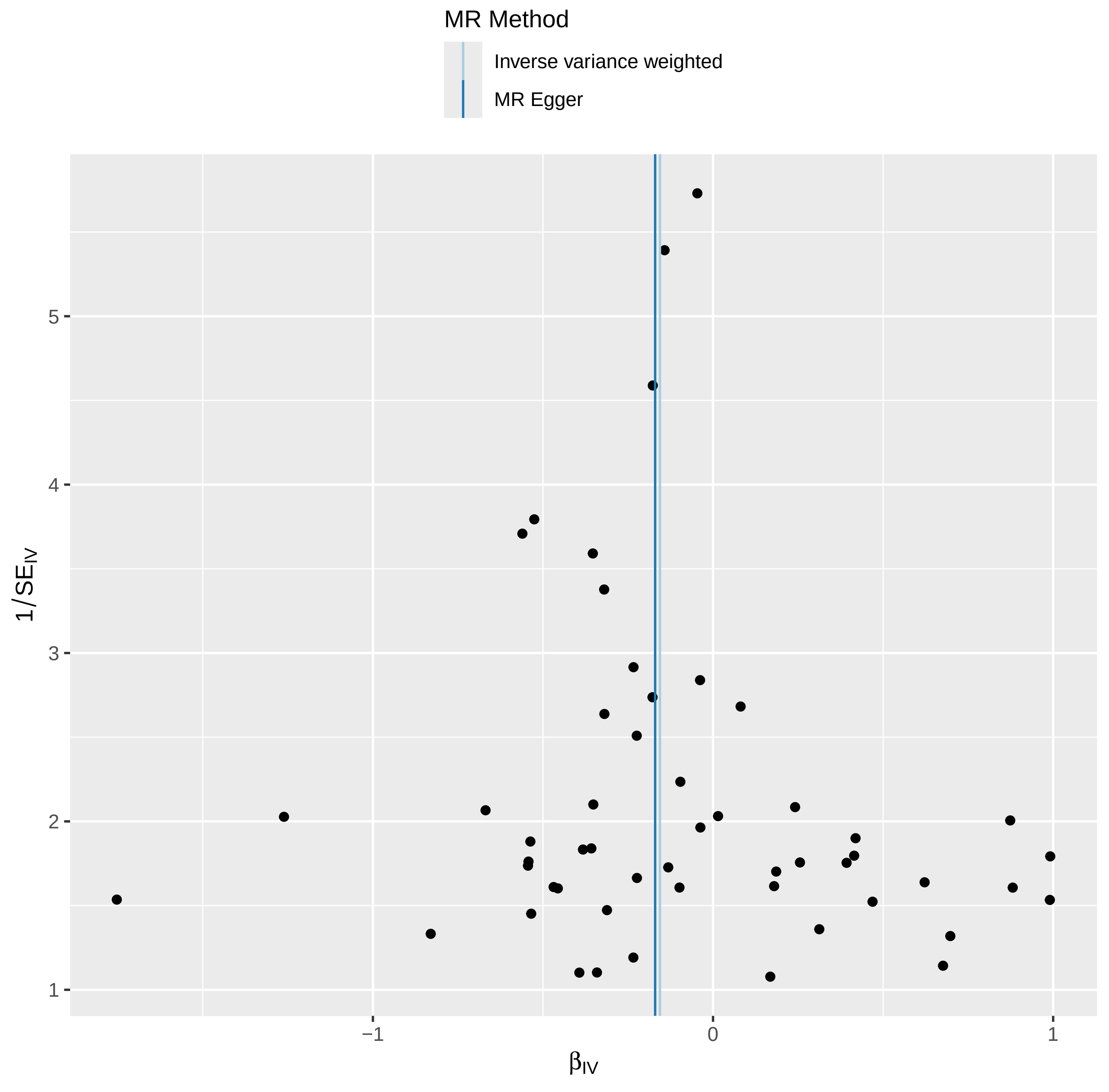

MR Method
Inverse variance weighted
MR Egger
1/SE_IV
5
4
3
2
1
−1
0
1
β_IV

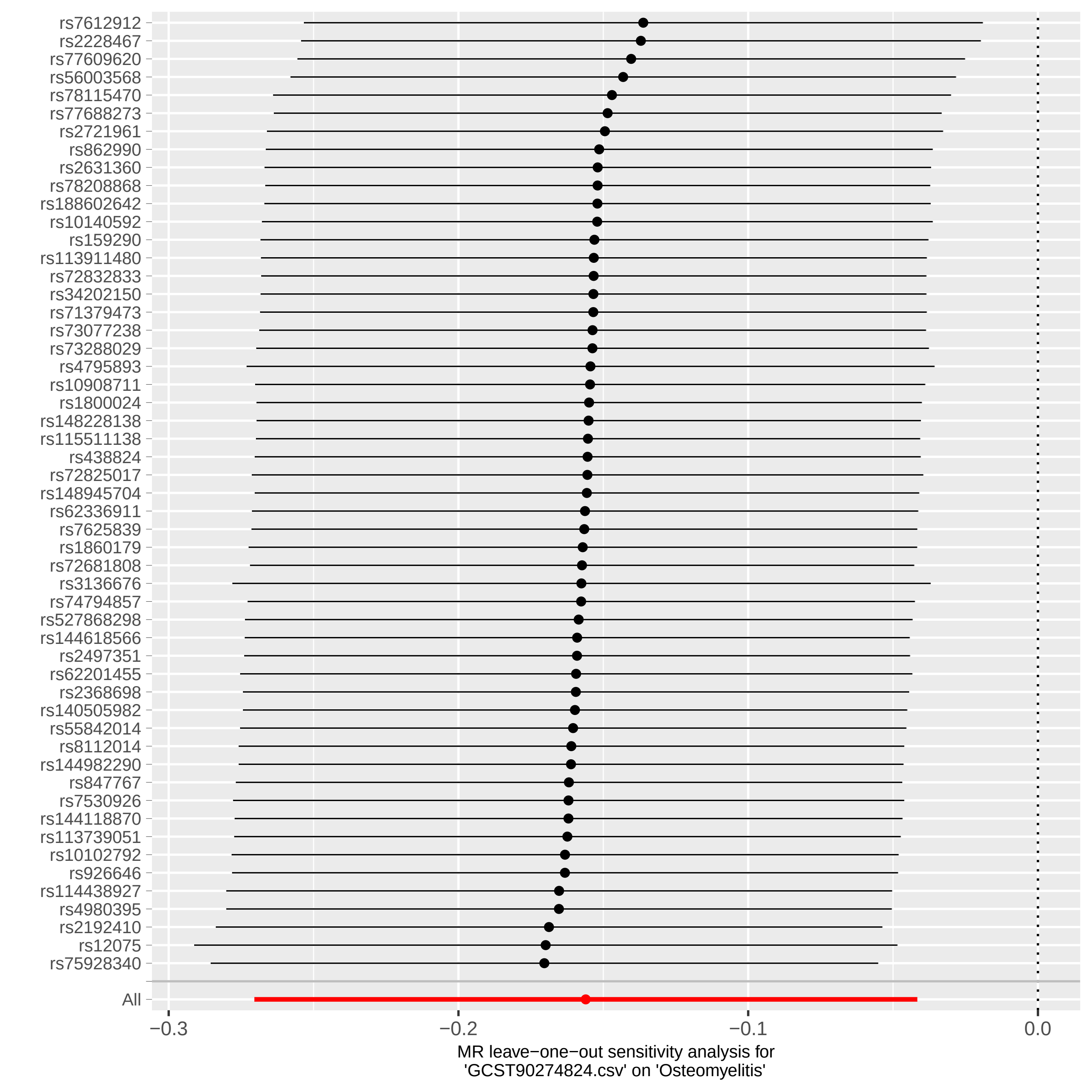

rs7612912
rs2228467
rs77609620
rs56003568
rs78115470
rs77688273
rs2721961
rs862990
rs2631360
rs78208868
rs188602642
rs10140592
rs159290
rs113911480
rs72832833
rs34202150
rs71379473
rs73077238
rs73288029
rs4795893
rs10908711
rs1800024
rs148228138
rs115511138
rs438824
rs72825017
rs148945704
rs62336911
rs7625839
rs1860179
rs72681808
rs3136676
rs74794857
rs527868298
rs144618566
rs2497351
rs62201455
rs2368698
rs140505982
rs55842014
rs8112014
rs144982290
rs847767
rs7530926
rs144118870
rs113739051
rs10102792
rs926646
rs114438927
rs4980395
rs2192410
rs12075
rs75928340
All
−0.3
−0.2
−0.1
0.0
MR leave−one−out sensitivity analysis for
'GCST90274824.csv' on 'Osteomyelitis'

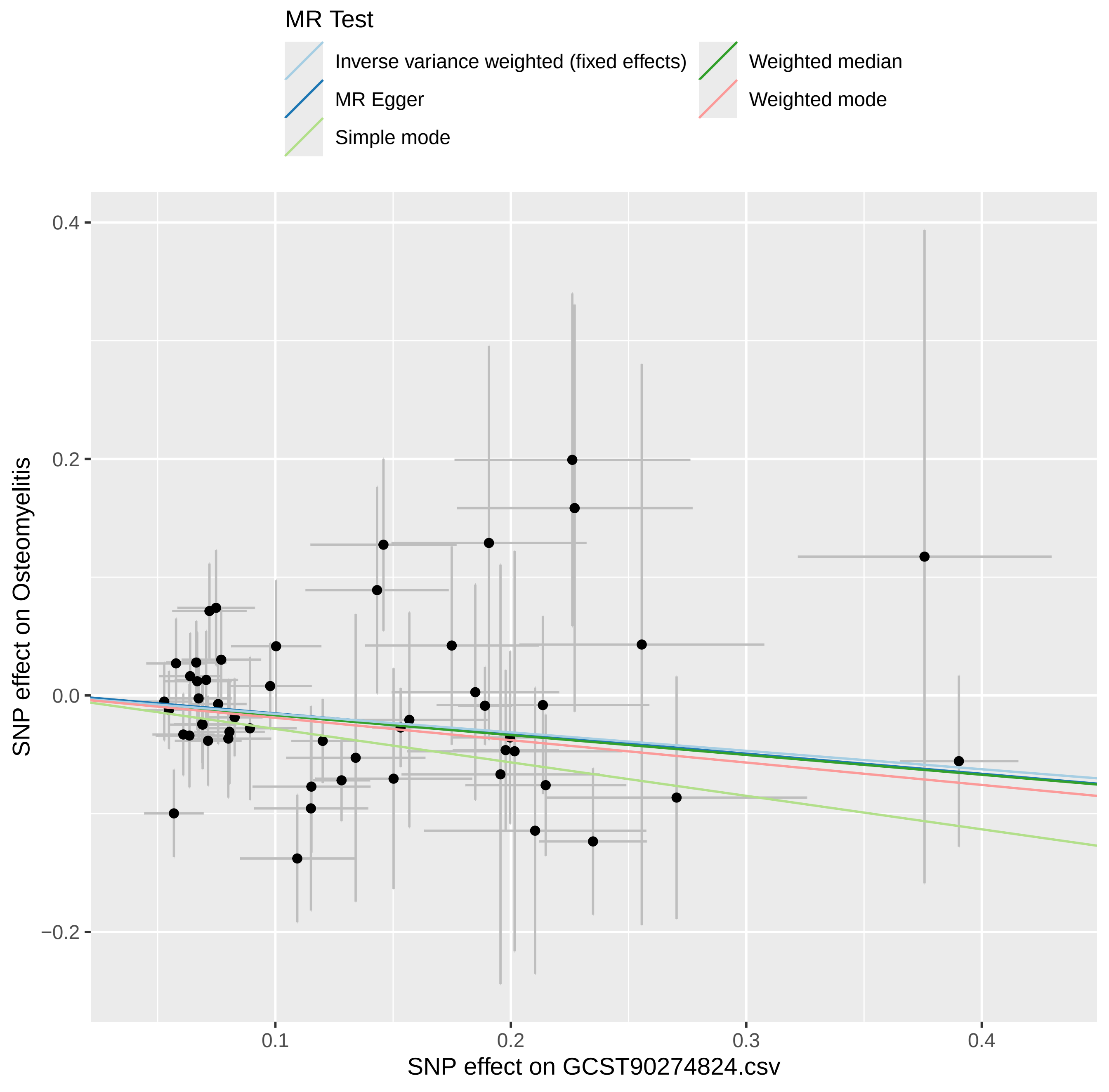
MR Test
Inverse variance weighted (fixed effects)
MR Egger
Simple mode
Weighted median
Weighted mode
0.4
0.2
0.0
−0.2
0.1
0.2
0.3
0.4
SNP effect on Osteomyelitis
SNP effect on GCST90274824.csv

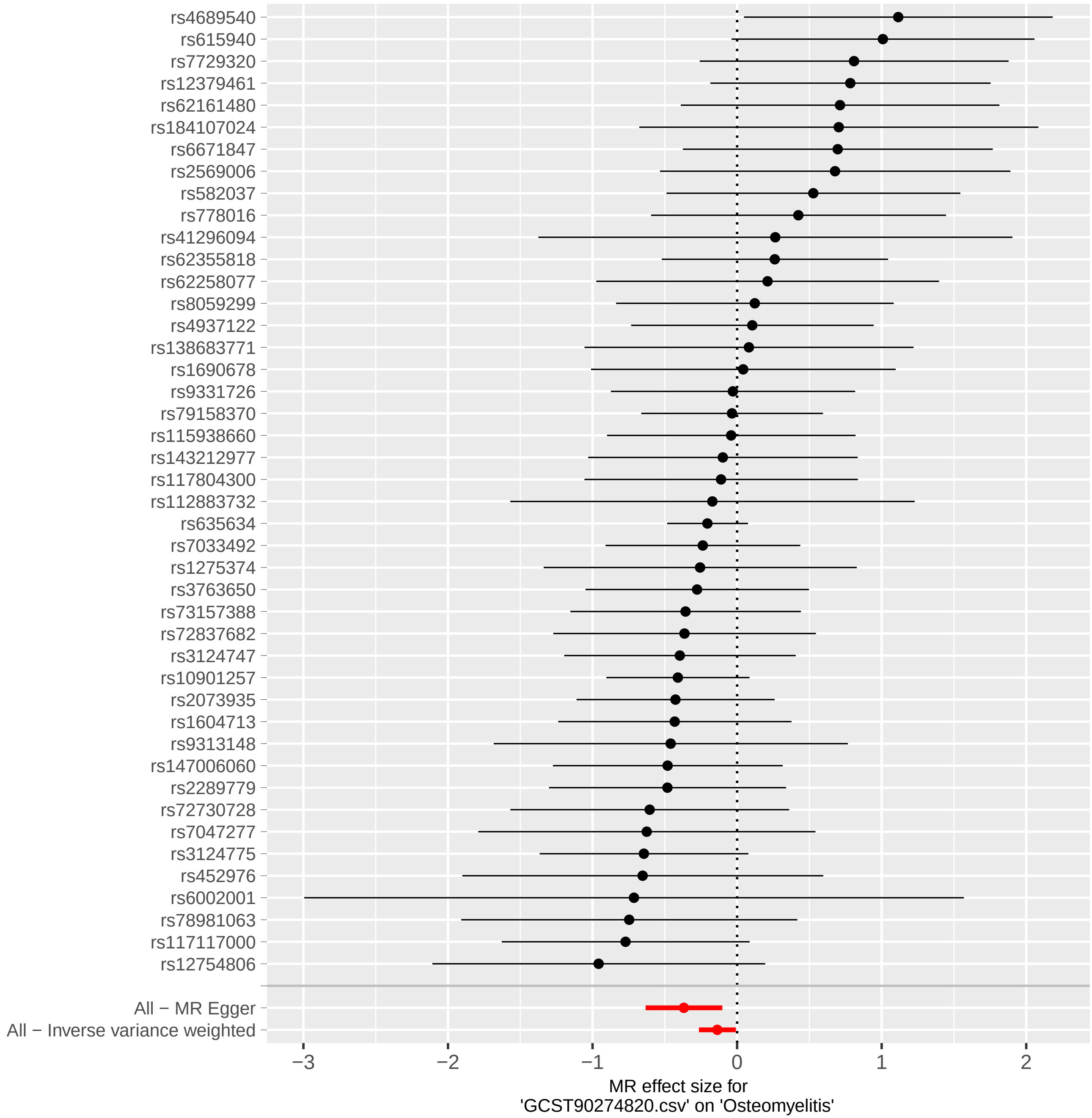

rs4689540
rs615940
rs7729320
rs12379461
rs62161480
rs184107024
rs6671847
rs2569006
rs582037
rs778016
rs41296094
rs62355818
rs62258077
rs8059299
rs4937122
rs138683771
rs1690678
rs9331726
rs79158370
rs115938660
rs143212977
rs117804300
rs112883732
rs635634
rs7033492
rs1275374
rs3763650
rs73157388
rs72837682
rs3124747
rs10901257
rs2073935
rs1604713
rs9313148
rs147006060
rs2289779
rs72730728
rs7047277
rs3124775
rs452976
rs6002001
rs78981063
rs117117000
rs12754806
All − MR Egger
All − Inverse variance weighted
−3
−2
−1
0
1
2
MR effect size for
'GCST90274820.csv' on 'Osteomyelitis'

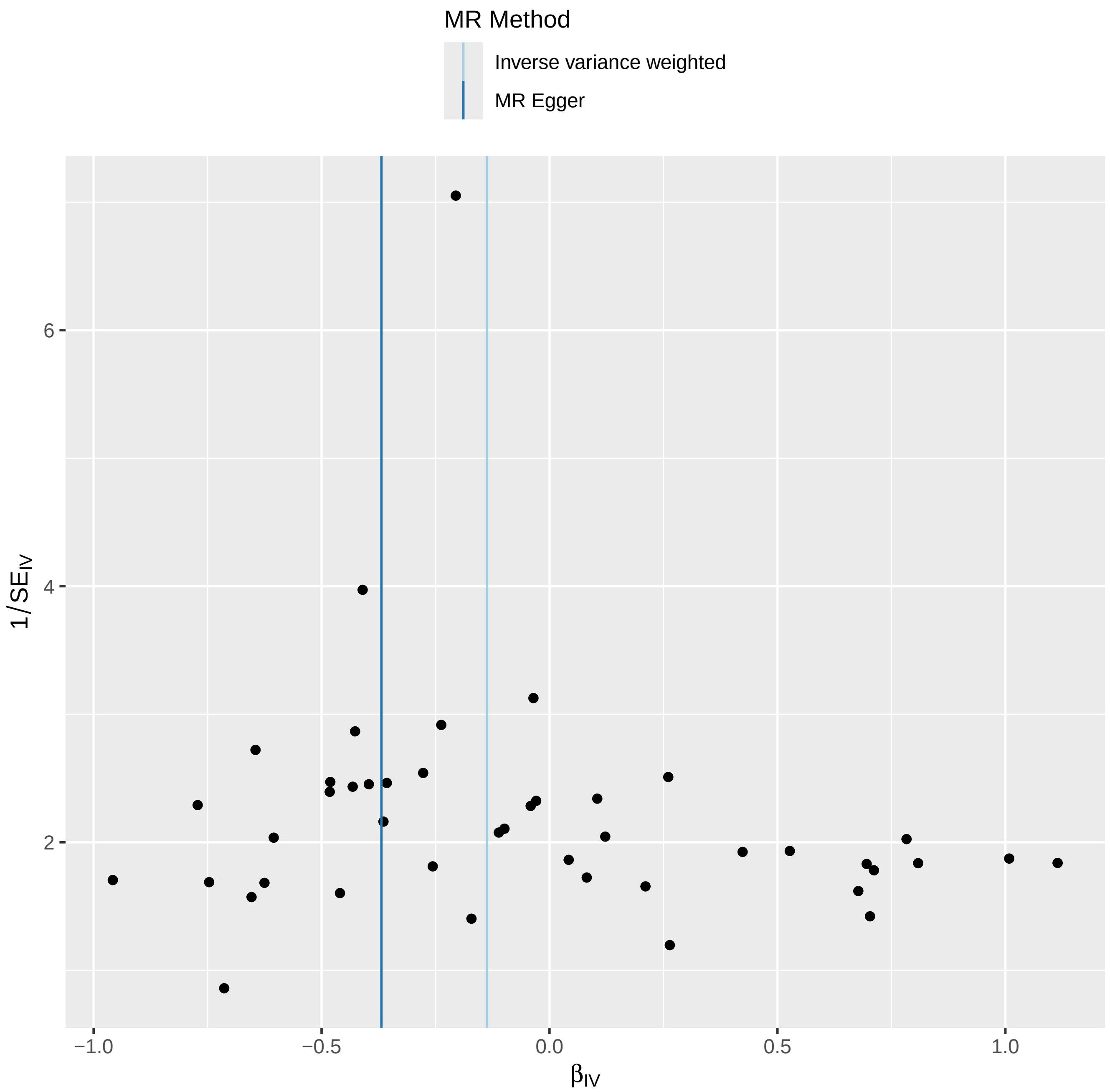

MR Method
Inverse variance weighted
MR Egger
6
4
2
1/SE_IV
−1.0
−0.5
0.0
0.5
1.0
β_IV

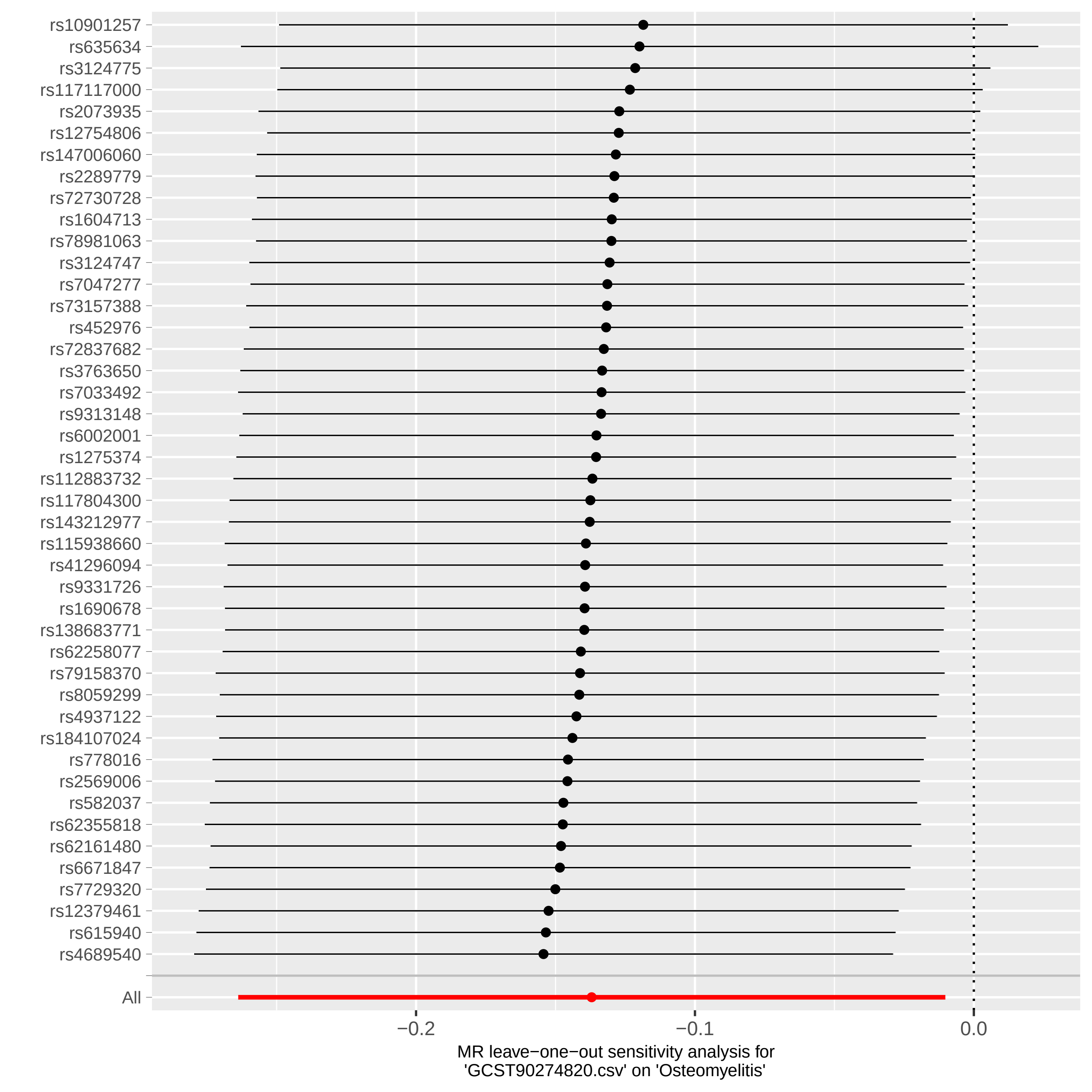
rs10901257
rs635634
rs3124775
rs117117000
rs2073935
rs12754806
rs147006060
rs2289779
rs72730728
rs1604713
rs78981063
rs3124747
rs7047277
rs73157388
rs452976
rs72837682
rs3763650
rs7033492
rs9313148
rs6002001
rs1275374
rs112883732
rs117804300
rs143212977
rs115938660
rs41296094
rs9331726
rs1690678
rs138683771
rs62258077
rs79158370
rs8059299
rs4937122
rs184107024
rs778016
rs2569006
rs582037
rs62355818
rs62161480
rs6671847
rs7729320
rs12379461
rs615940
rs4689540
All
−0.2
−0.1
0.0
MR leave−one−out sensitivity analysis for
'GCST90274820.csv' on 'Osteomyelitis'

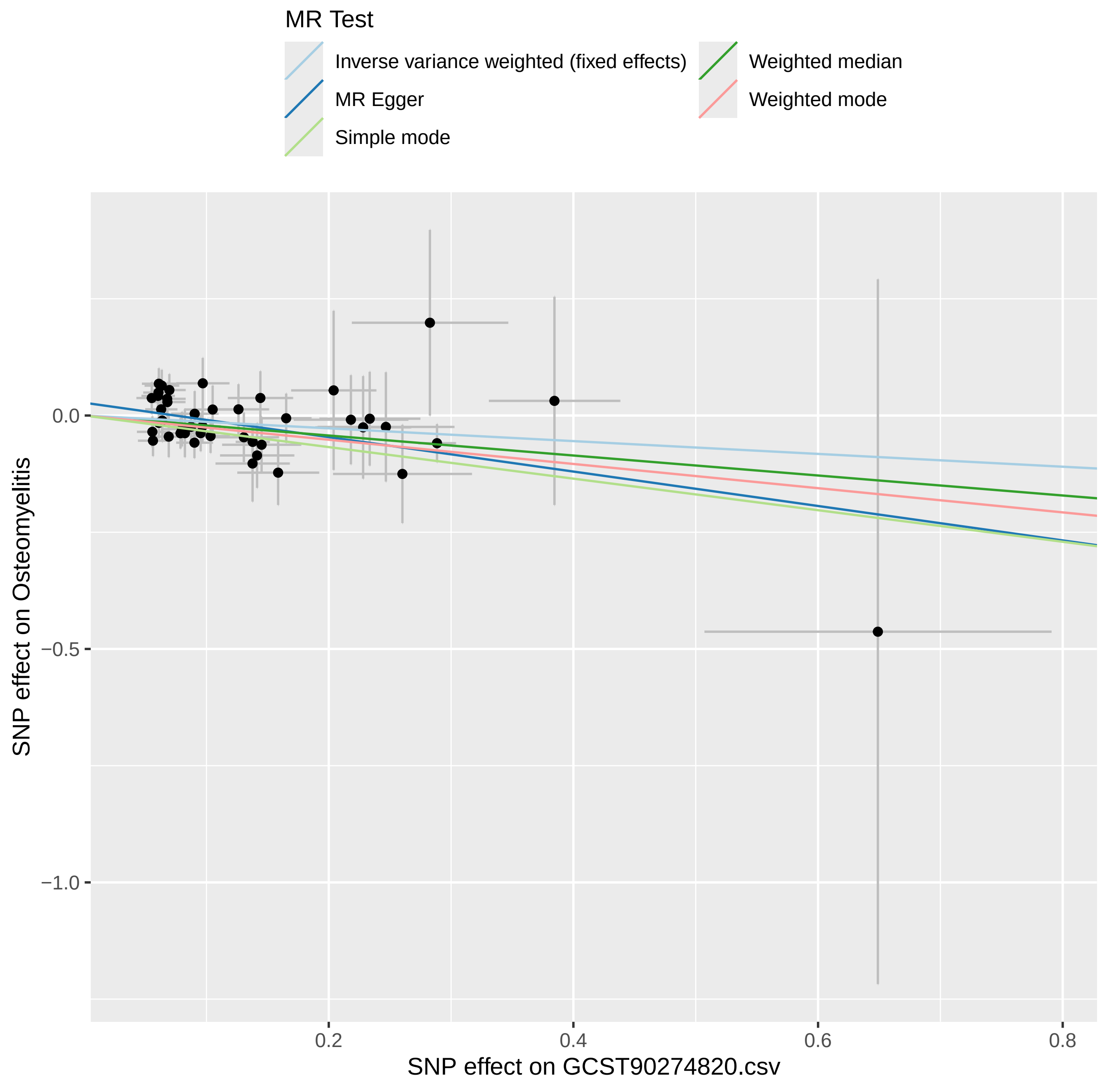
MR Test
Inverse variance weighted (fixed effects)
MR Egger
Simple mode
Weighted median
Weighted mode
0.0
−0.5
−1.0
0.2
0.4
0.6
0.8
SNP effect on Osteomyelitis
SNP effect on GCST90274820.csv

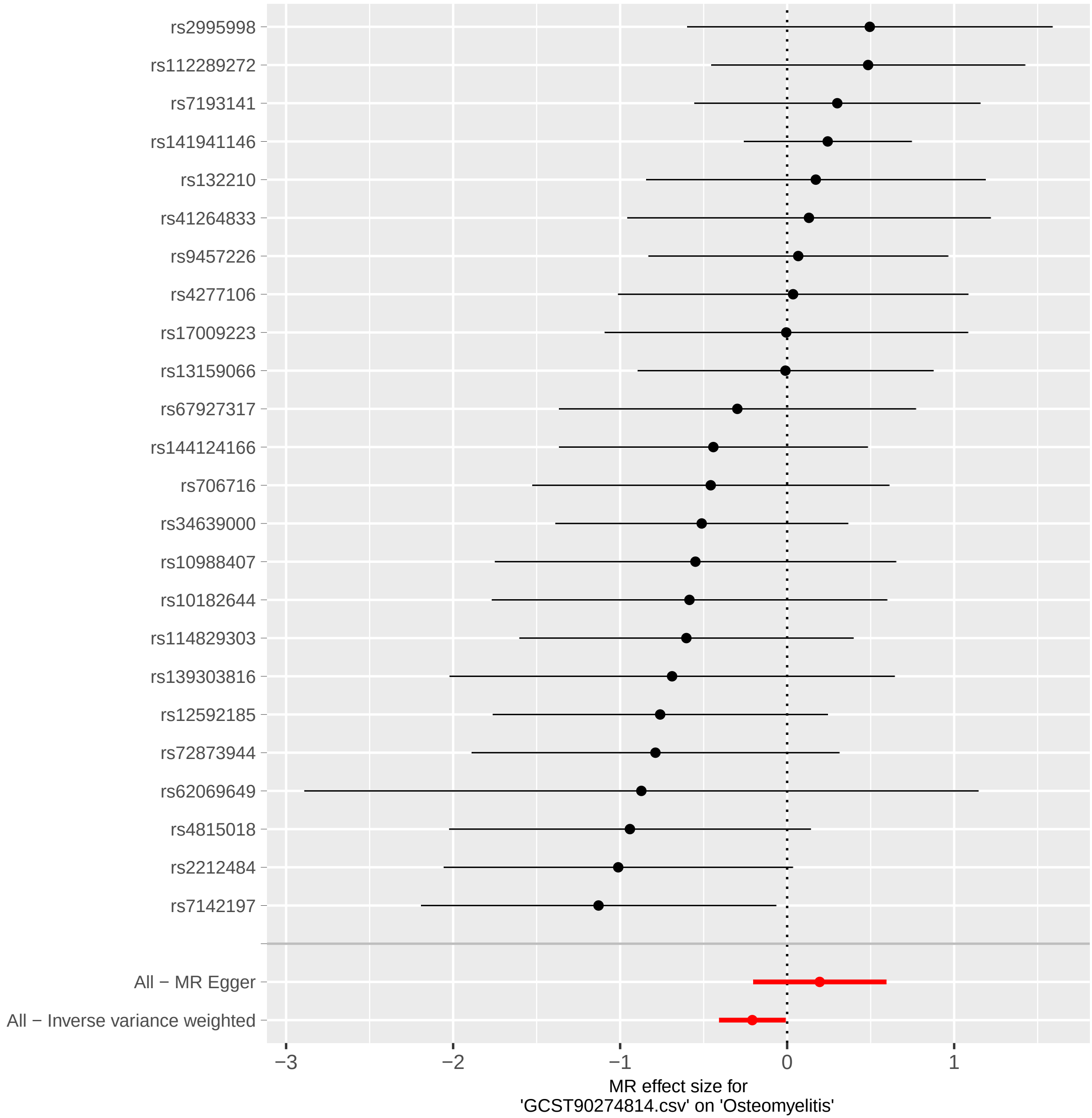

rs2995998
rs112289272
rs7193141
rs141941146
rs132210
rs41264833
rs9457226
rs4277106
rs17009223
rs13159066
rs67927317
rs144124166
rs706716
rs34639000
rs10988407
rs10182644
rs114829303
rs139303816
rs12592185
rs72873944
rs62069649
rs4815018
rs2212484
rs7142197
All − MR Egger
All − Inverse variance weighted
−3
−2
−1
0
1
MR effect size for
'GCST90274814.csv' on 'Osteomyelitis'

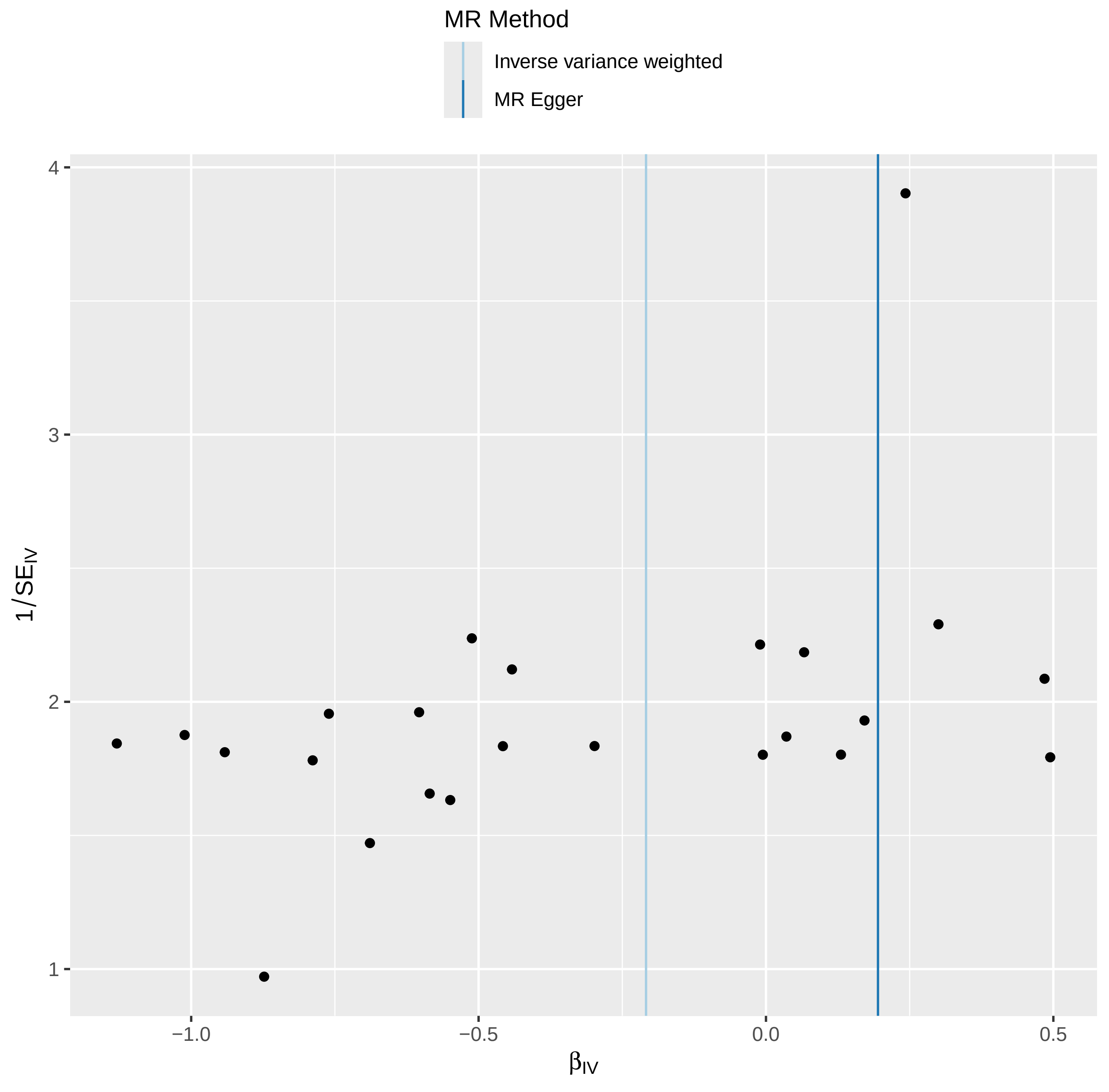

MR Method
Inverse variance weighted
MR Egger
1/SE_{IV}
β_{IV}
4
3
2
1
−1.0
−0.5
0.0
0.5

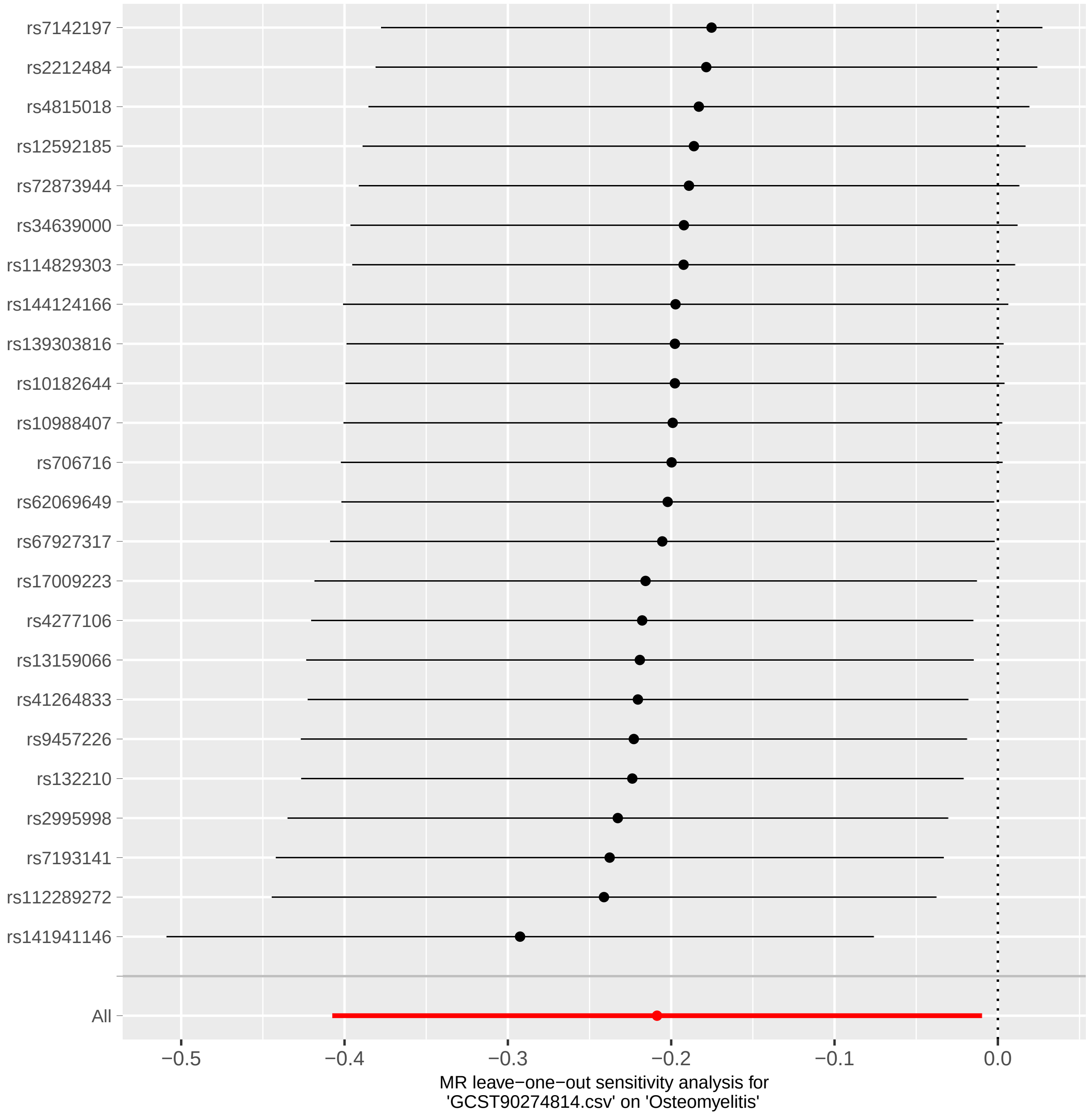

rs7142197
rs2212484
rs4815018
rs12592185
rs72873944
rs34639000
rs114829303
rs144124166
rs139303816
rs10182644
rs10988407
rs706716
rs62069649
rs67927317
rs17009223
rs4277106
rs13159066
rs41264833
rs9457226
rs132210
rs2995998
rs7193141
rs112289272
rs141941146
All
−0.5
−0.4
−0.3
−0.2
−0.1
0.0
MR leave−one−out sensitivity analysis for
'GCST90274814.csv' on 'Osteomyelitis'

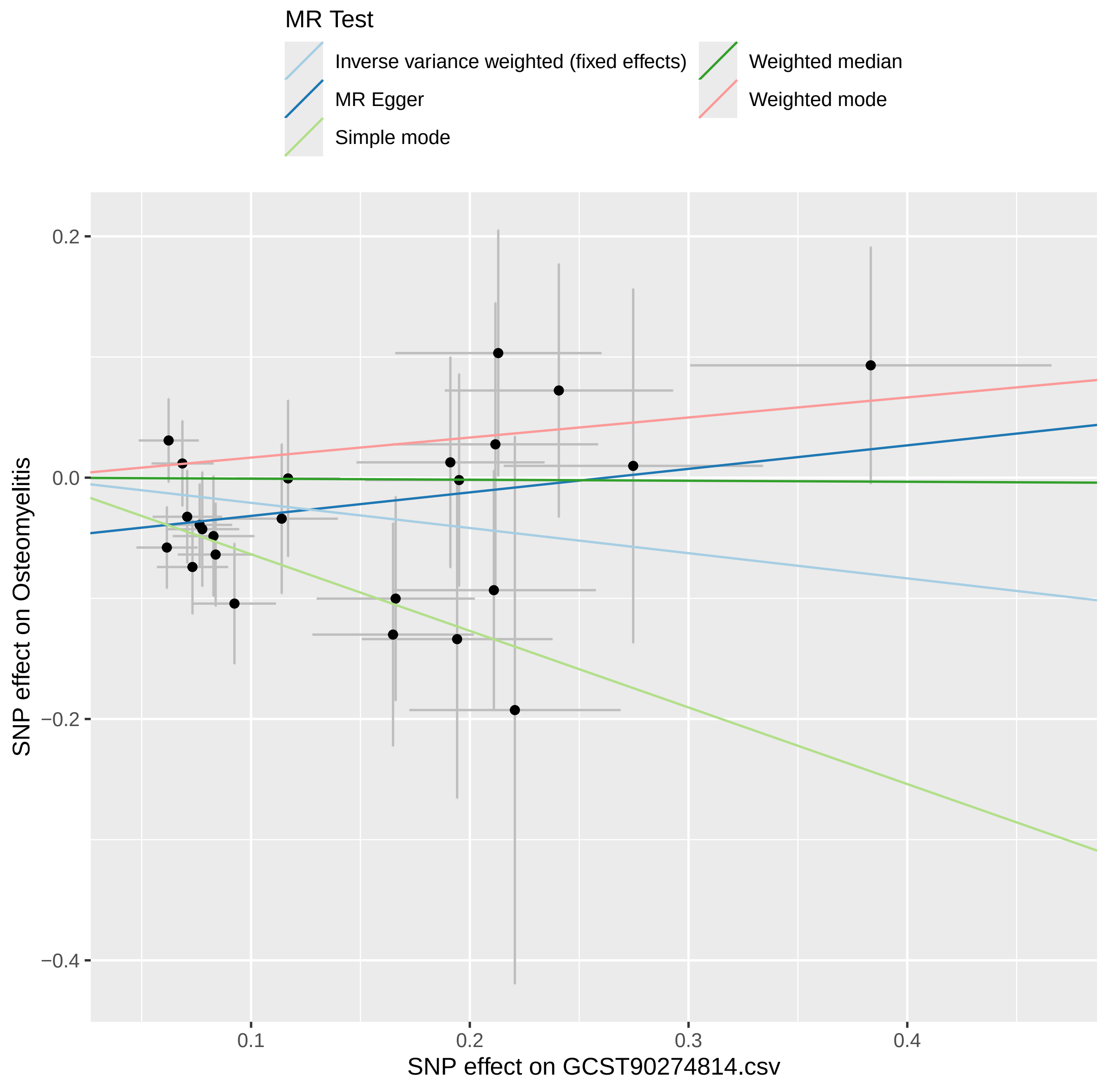
MR Test
Inverse variance weighted (fixed effects)
MR Egger
Simple mode
Weighted median
Weighted mode
0.2
0.0
−0.2
−0.4
0.1
0.2
0.3
0.4
SNP effect on Osteomyelitis
SNP effect on GCST90274814.csv

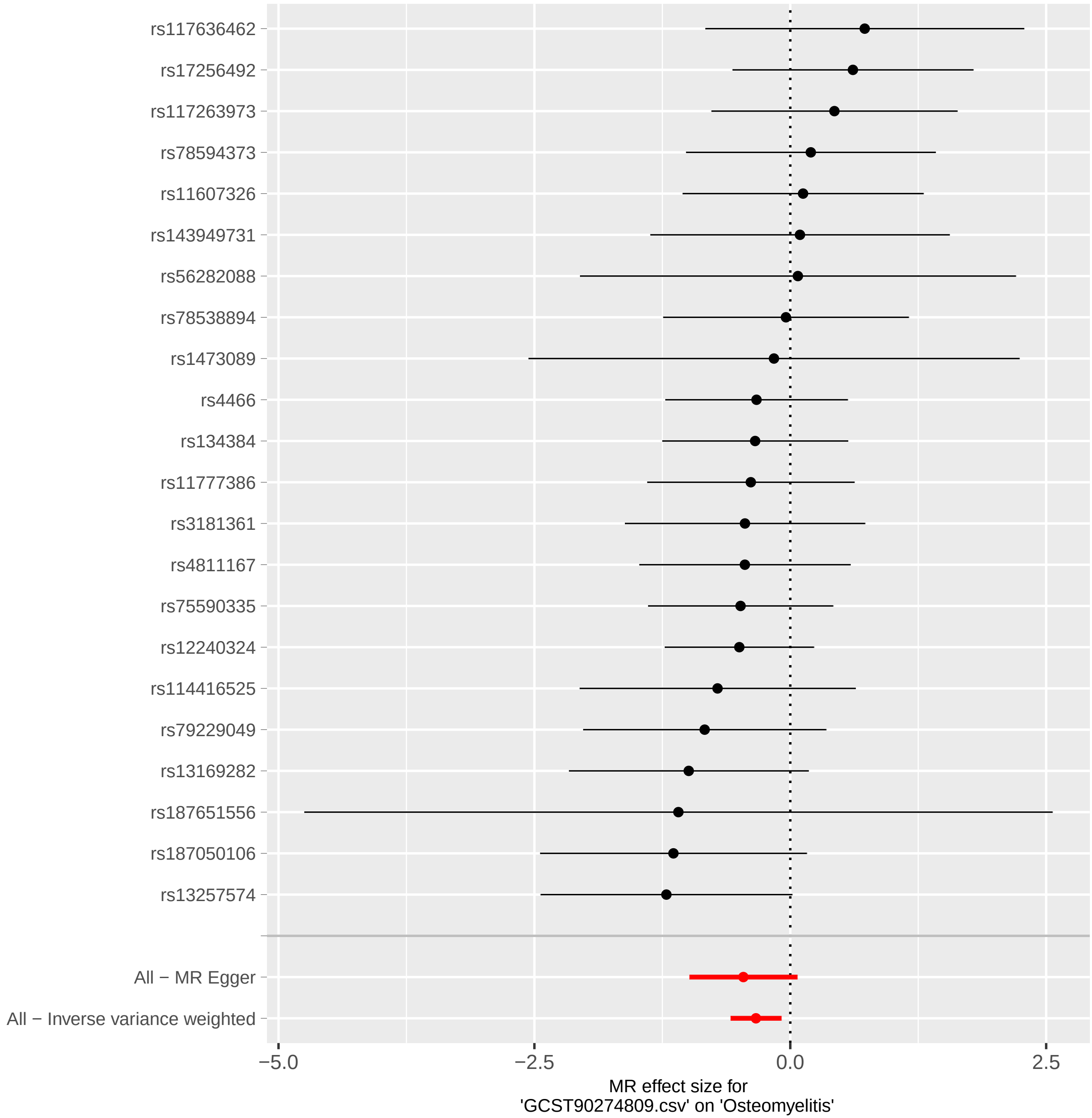
rs117636462
rs17256492
rs117263973
rs78594373
rs11607326
rs143949731
rs56282088
rs78538894
rs1473089
rs4466
rs134384
rs11777386
rs3181361
rs4811167
rs75590335
rs12240324
rs114416525
rs79229049
rs13169282
rs187651556
rs187050106
rs13257574
All − MR Egger
All − Inverse variance weighted
−5.0
−2.5
0.0
2.5
MR effect size for
'GCST90274809.csv' on 'Osteomyelitis'

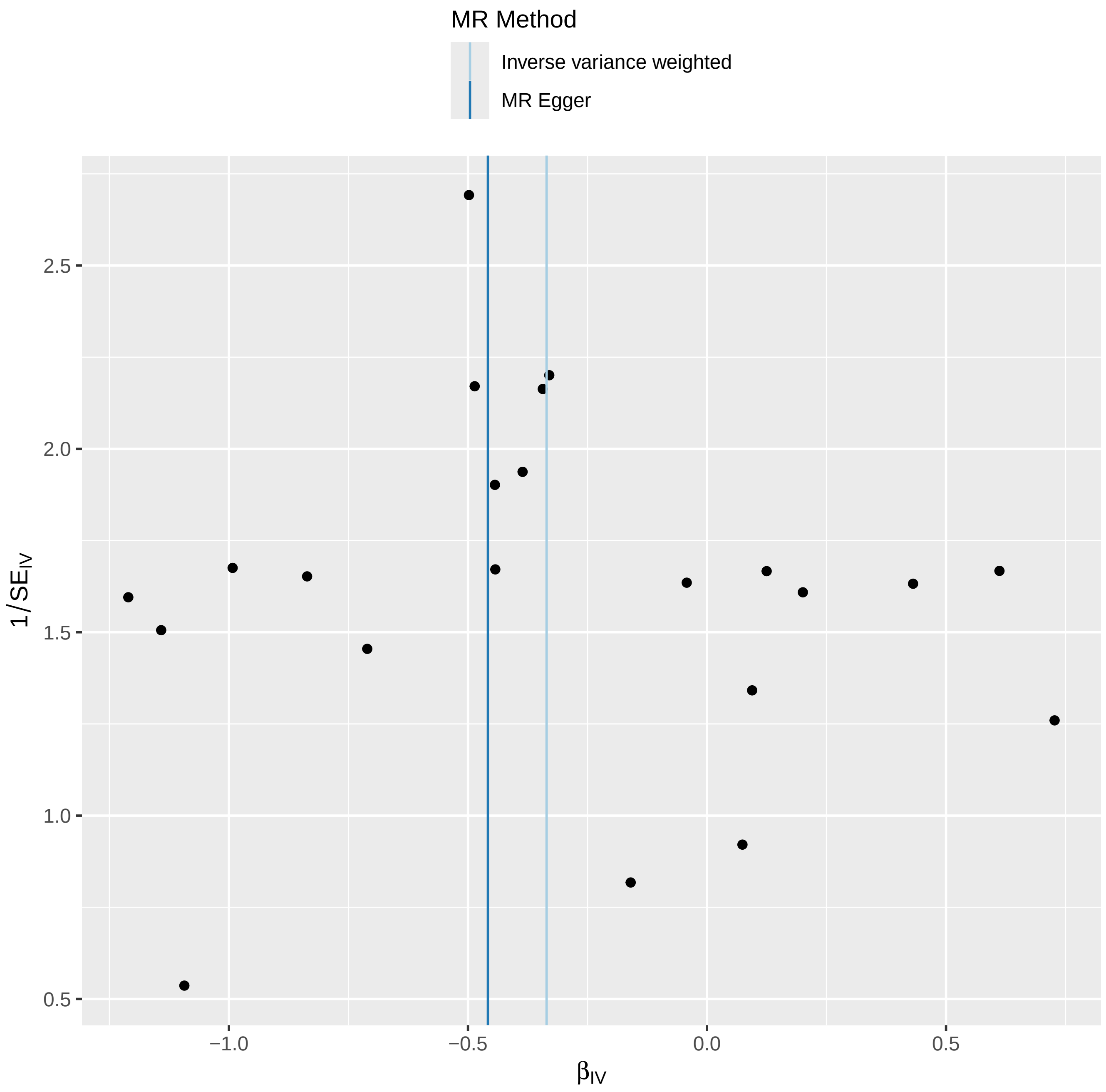
MR Method
Inverse variance weighted
MR Egger
1/SE_{IV}
2.5
2.0
1.5
1.0
0.5
−1.0
−0.5
0.0
0.5
β_{IV}

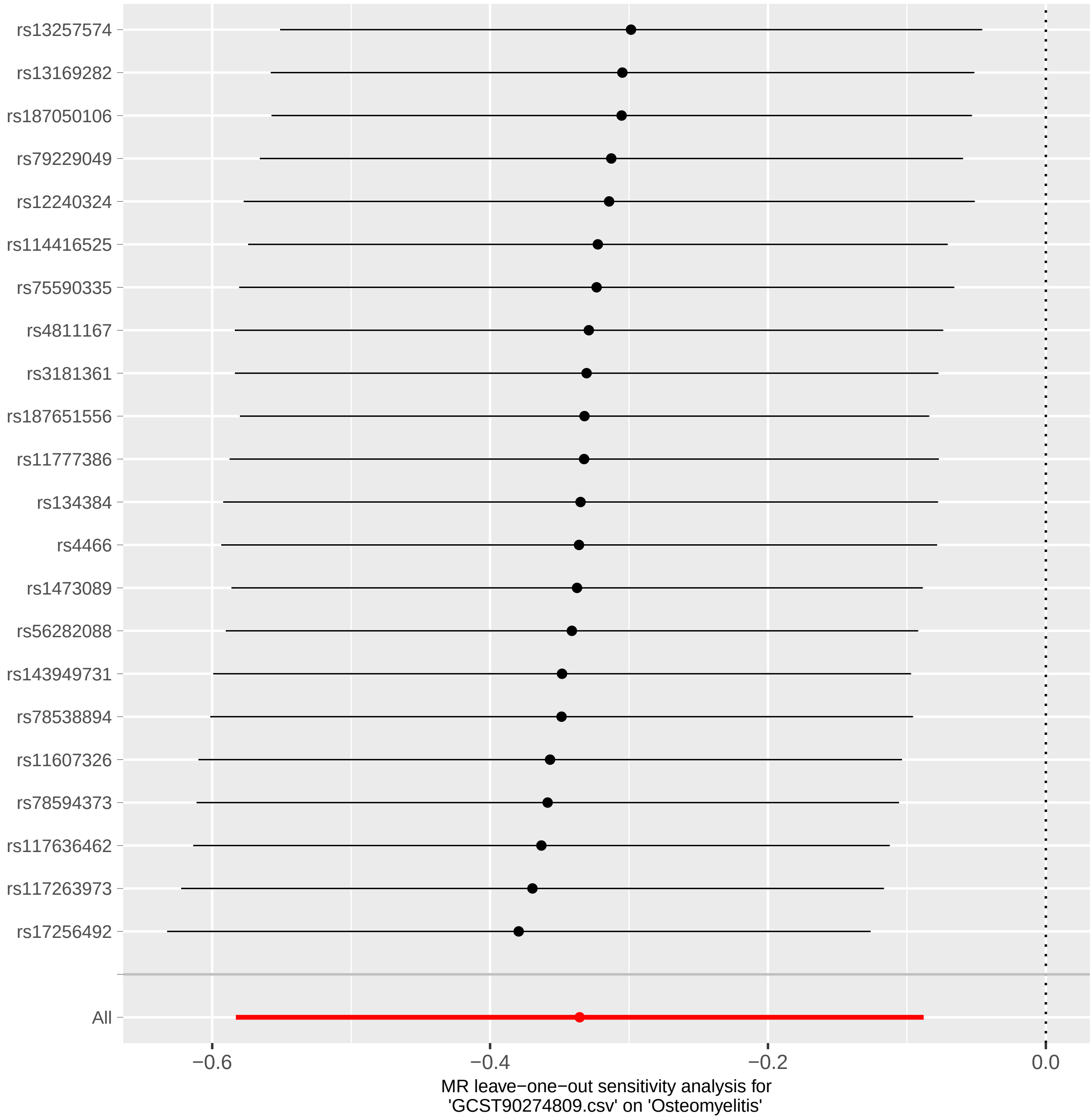
rs13257574
rs13169282
rs187050106
rs79229049
rs12240324
rs114416525
rs75590335
rs4811167
rs3181361
rs187651556
rs11777386
rs134384
rs4466
rs1473089
rs56282088
rs143949731
rs78538894
rs11607326
rs78594373
rs117636462
rs117263973
rs17256492
All
−0.6
−0.4
−0.2
0.0
MR leave−one−out sensitivity analysis for
'GCST90274809.csv' on 'Osteomyelitis'

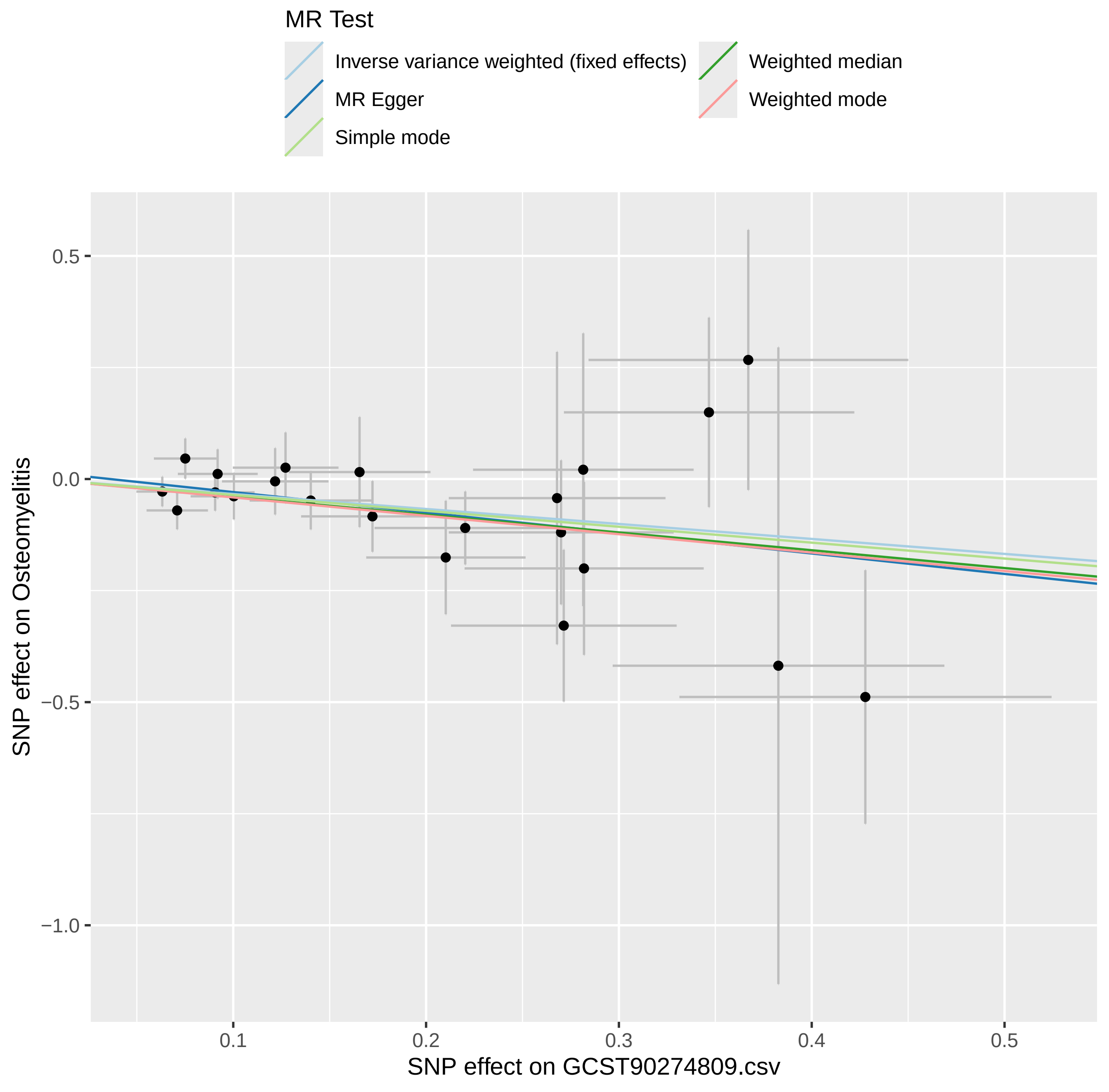
MR Test
Inverse variance weighted (fixed effects)
MR Egger
Simple mode
Weighted median
Weighted mode
0.5
0.0
−0.5
−1.0
0.1
0.2
0.3
0.4
0.5
SNP effect on Osteomyelitis
SNP effect on GCST90274809.csv

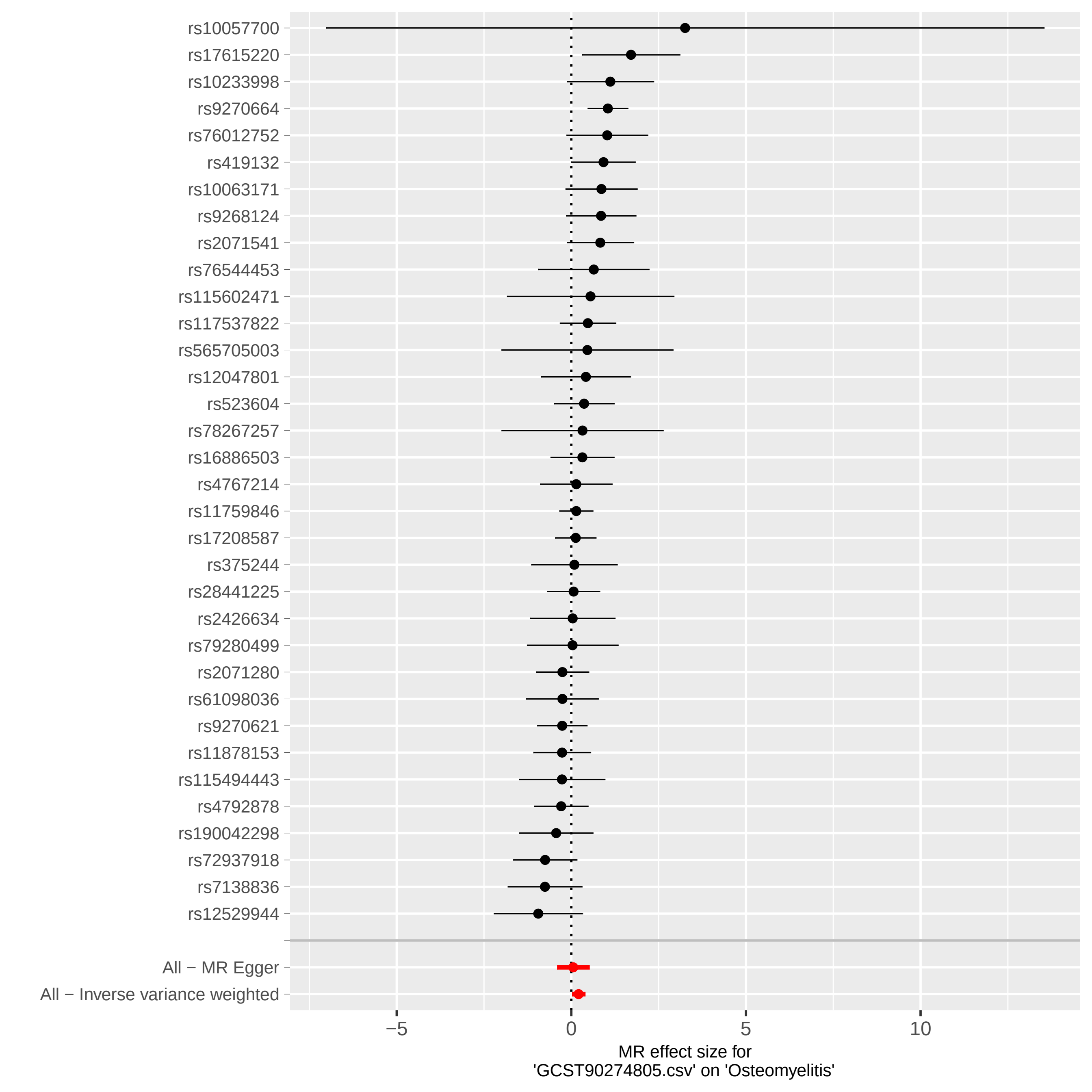
rs10057700
rs17615220
rs10233998
rs9270664
rs76012752
rs419132
rs10063171
rs9268124
rs2071541
rs76544453
rs115602471
rs117537822
rs565705003
rs12047801
rs523604
rs78267257
rs16886503
rs4767214
rs11759846
rs17208587
rs375244
rs28441225
rs2426634
rs79280499
rs2071280
rs61098036
rs9270621
rs11878153
rs115494443
rs4792878
rs190042298
rs72937918
rs7138836
rs12529944
All − MR Egger
All − Inverse variance weighted
−5
0
5
10
MR effect size for
'GCST90274805.csv' on 'Osteomyelitis'

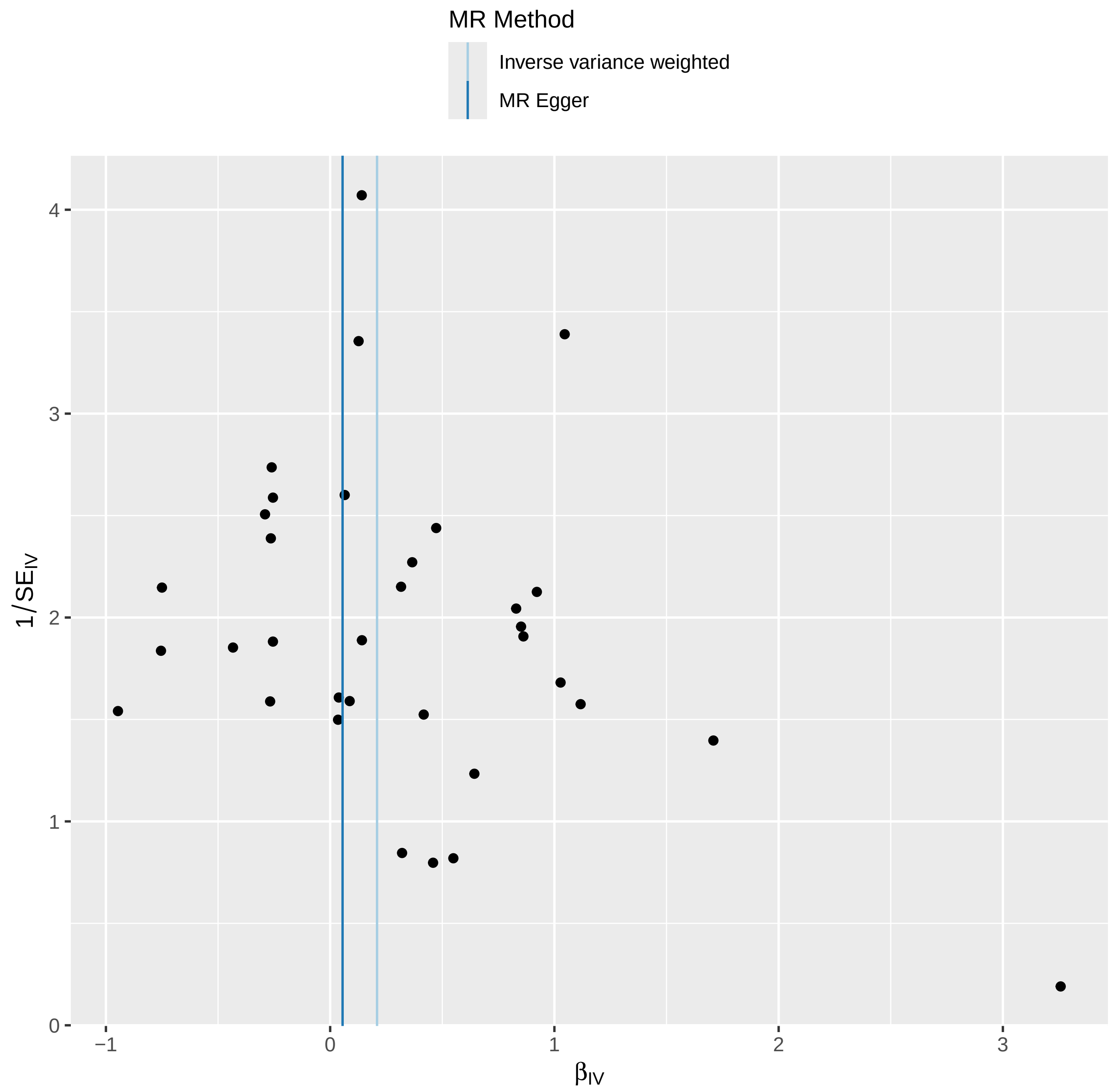

MR Method
Inverse variance weighted
MR Egger
4
3
2
1
0
1/SE_IV
−1
0
1
2
3
β_IV

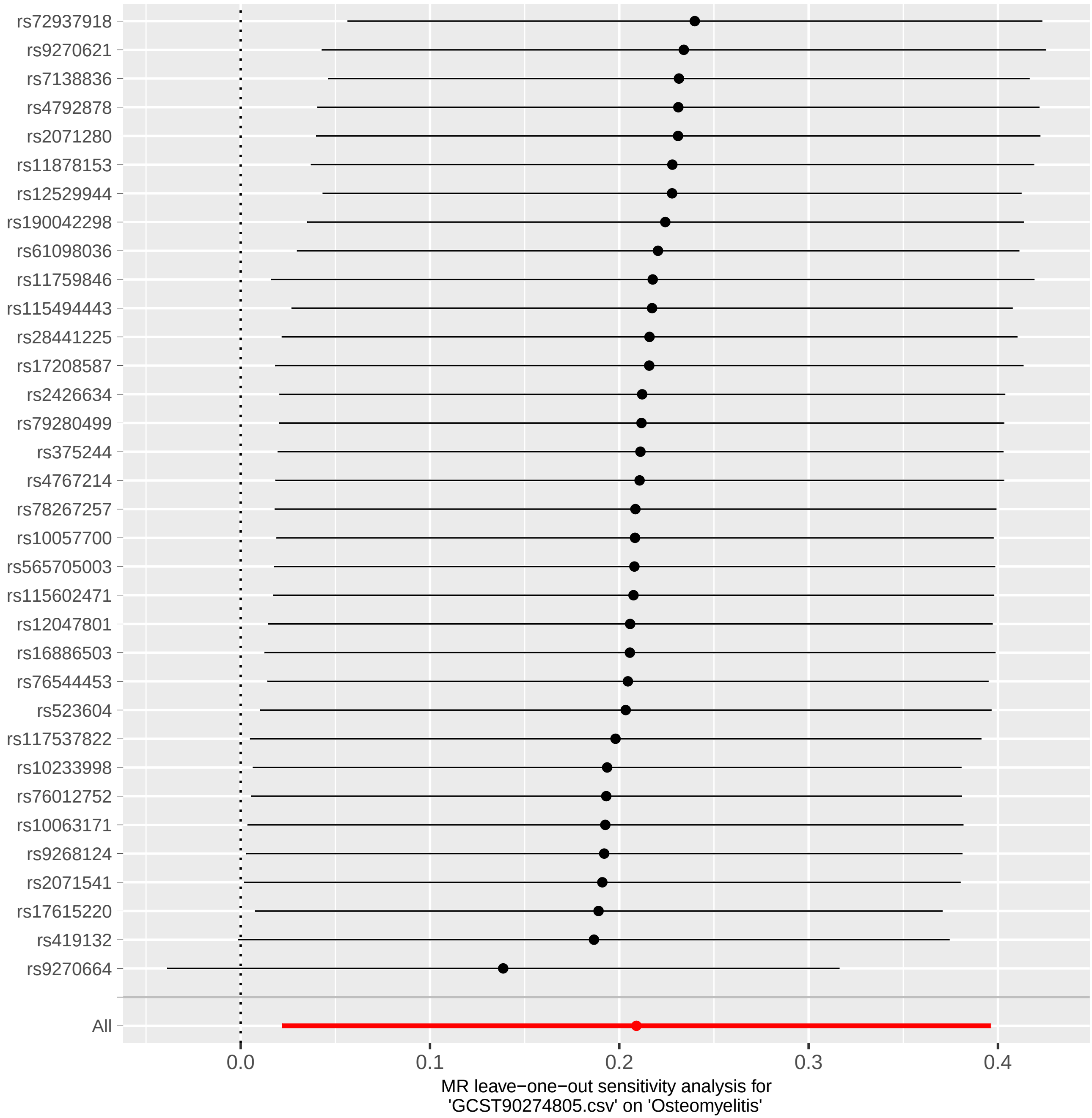
rs72937918
rs9270621
rs7138836
rs4792878
rs2071280
rs11878153
rs12529944
rs190042298
rs61098036
rs11759846
rs115494443
rs28441225
rs17208587
rs2426634
rs79280499
rs375244
rs4767214
rs78267257
rs10057700
rs565705003
rs115602471
rs12047801
rs16886503
rs76544453
rs523604
rs117537822
rs10233998
rs76012752
rs10063171
rs9268124
rs2071541
rs17615220
rs419132
rs9270664
All
0.0
0.1
0.2
0.3
0.4
MR leave−one−out sensitivity analysis for
'GCST90274805.csv' on 'Osteomyelitis'

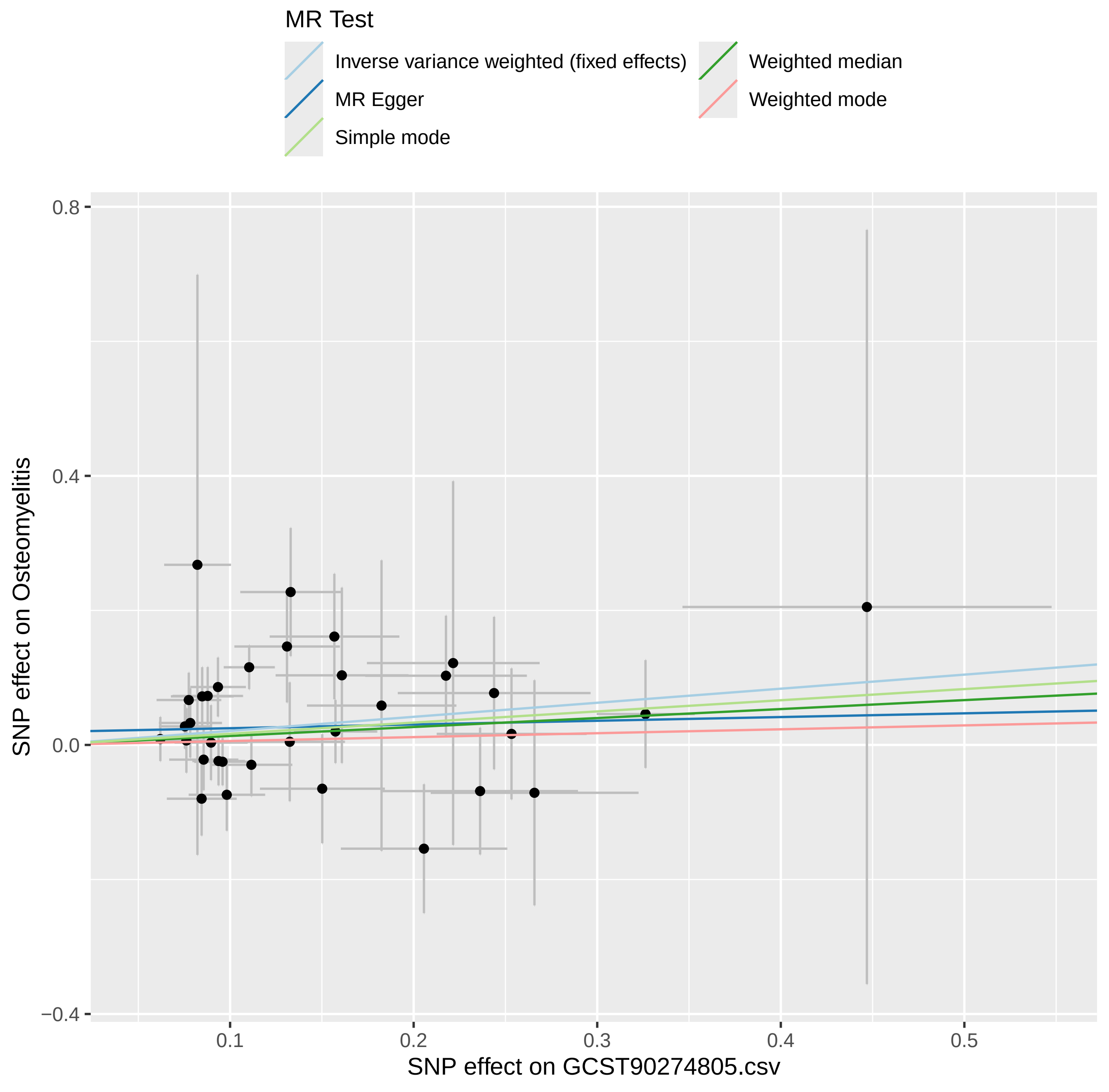
MR Test
Inverse variance weighted (fixed effects)
MR Egger
Simple mode
Weighted median
Weighted mode
0.8
0.4
0.0
−0.4
0.1
0.2
0.3
0.4
0.5
SNP effect on Osteomyelitis
SNP effect on GCST90274805.csv
