# Supplementary material for: Circulating inflammatory proteins and osteomyelitis: A bidirectional Mendelian randomization and colocalization analysis
Source: Medicine (Baltimore). 2025 Oct 17;104(42):e44916. doi: 10.1097/MD.0000000000044916 (PMC12537171; doi:10.1097/MD.0000000000044916)
Supplement: Supplementary file 4 [file medi-104-e44916-s004.pdf]

Supplementary Figures S12–S25. Diagnostic and sensitivity plots for reverse Mendelian randomization (MR) analyses, including scatter plots, forest plots, funnel plots, and leave-one-out analyses for each significant osteomyelitis–protein association.

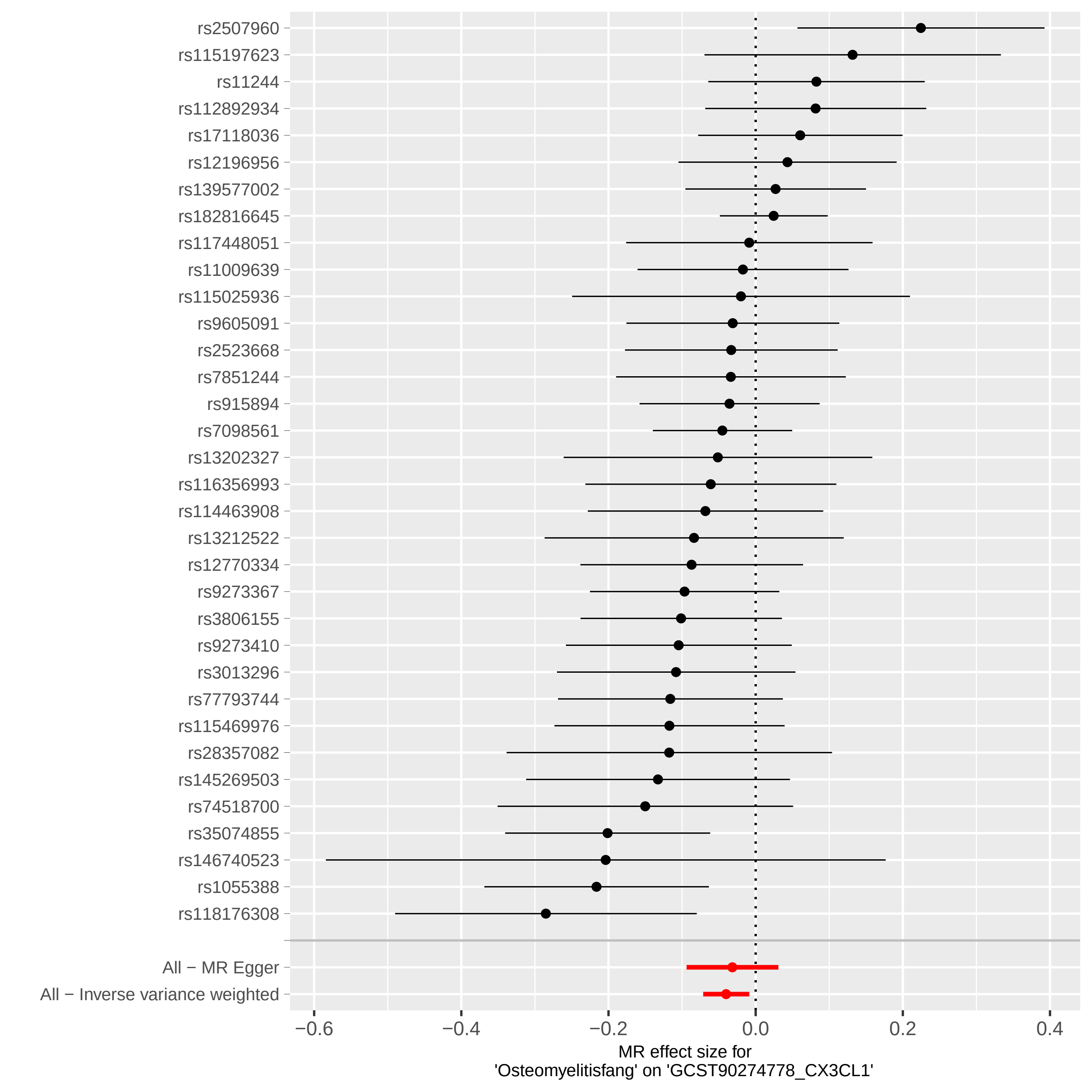

rs2507960
rs115197623
rs11244
rs112892934
rs17118036
rs12196956
rs139577002
rs182816645
rs117448051
rs11009639
rs115025936
rs9605091
rs2523668
rs7851244
rs915894
rs7098561
rs13202327
rs116356993
rs114463908
rs13212522
rs12770334
rs9273367
rs3806155
rs9273410
rs3013296
rs77793744
rs115469976
rs28357082
rs145269503
rs74518700
rs35074855
rs146740523
rs1055388
rs118176308
All − MR Egger
All − Inverse variance weighted
−0.6
−0.4
−0.2
0.0
0.2
0.4
MR effect size for
'Osteomyelitisfang' on 'GCST90274778_CX3CL1'

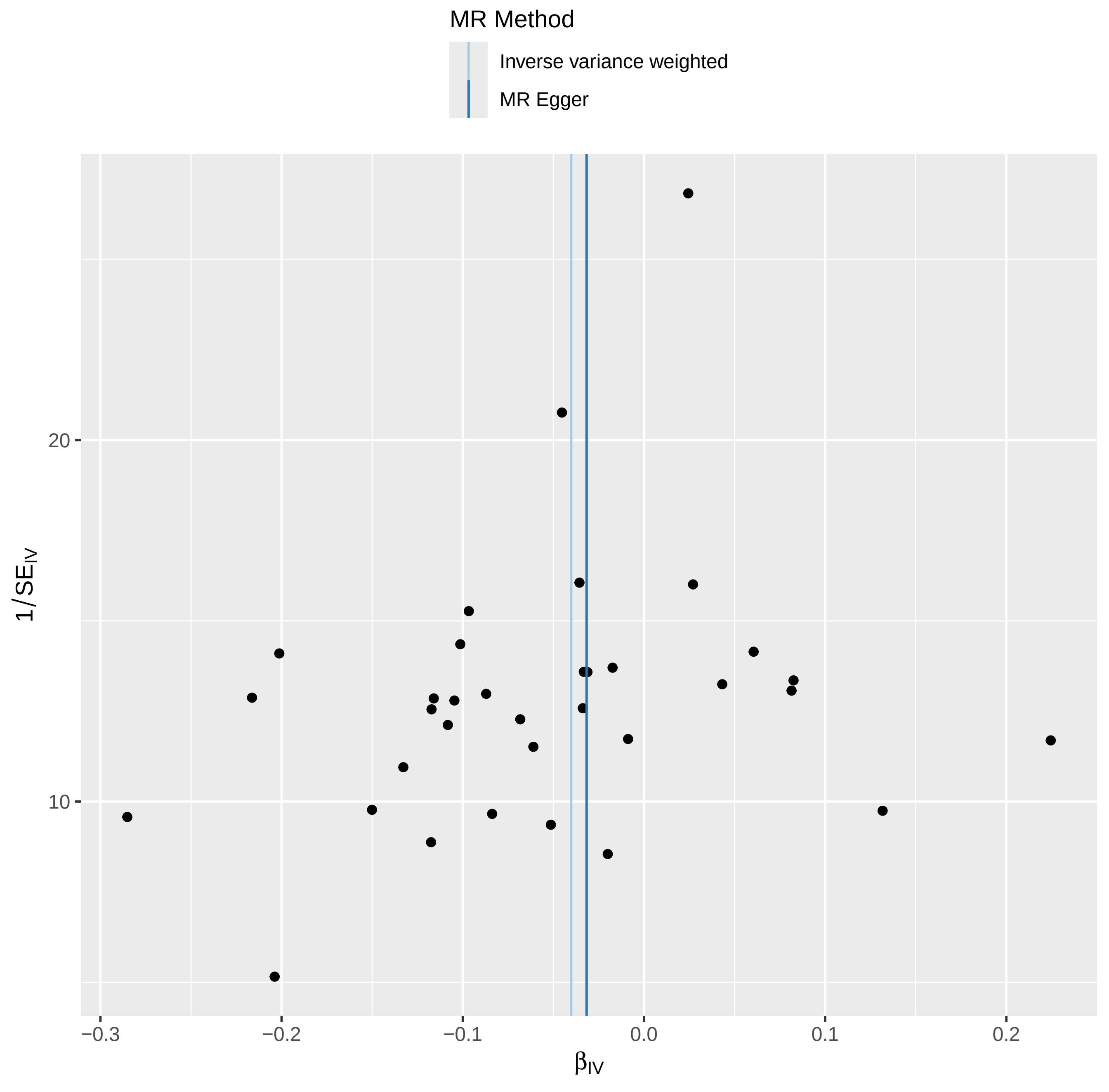

MR Method
Inverse variance weighted
MR Egger
20
10
1/SE$_{IV}$
−0.3
−0.2
−0.1
0.0
0.1
0.2
β$_{IV}$

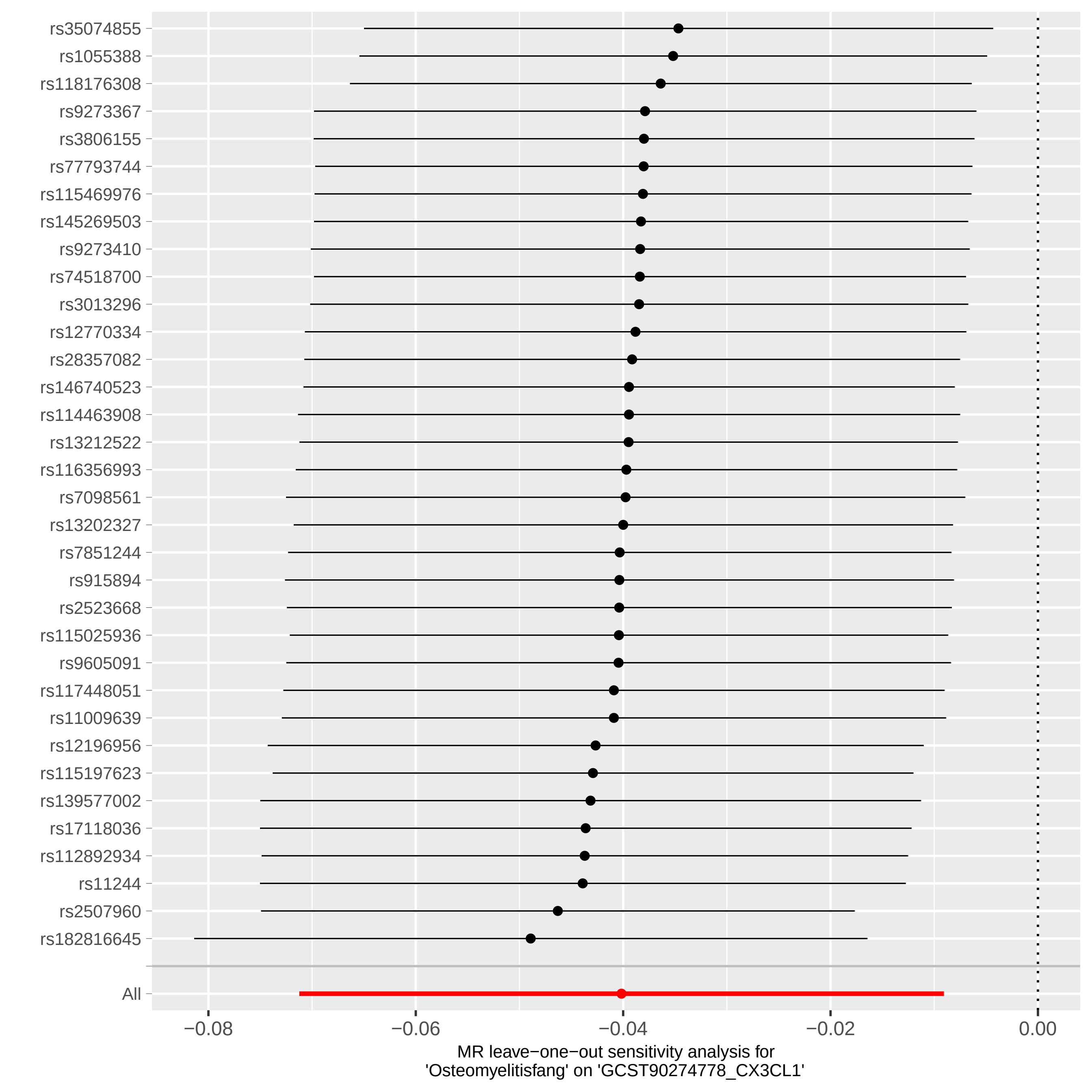
rs35074855
rs1055388
rs118176308
rs9273367
rs3806155
rs77793744
rs115469976
rs145269503
rs9273410
rs74518700
rs3013296
rs12770334
rs28357082
rs146740523
rs114463908
rs13212522
rs116356993
rs7098561
rs13202327
rs7851244
rs915894
rs2523668
rs115025936
rs9605091
rs117448051
rs11009639
rs12196956
rs115197623
rs139577002
rs17118036
rs112892934
rs11244
rs2507960
rs182816645
All
−0.08
−0.06
−0.04
−0.02
0.00
MR leave−one−out sensitivity analysis for
'Osteomyelitisfang' on 'GCST90274778_CX3CL1'

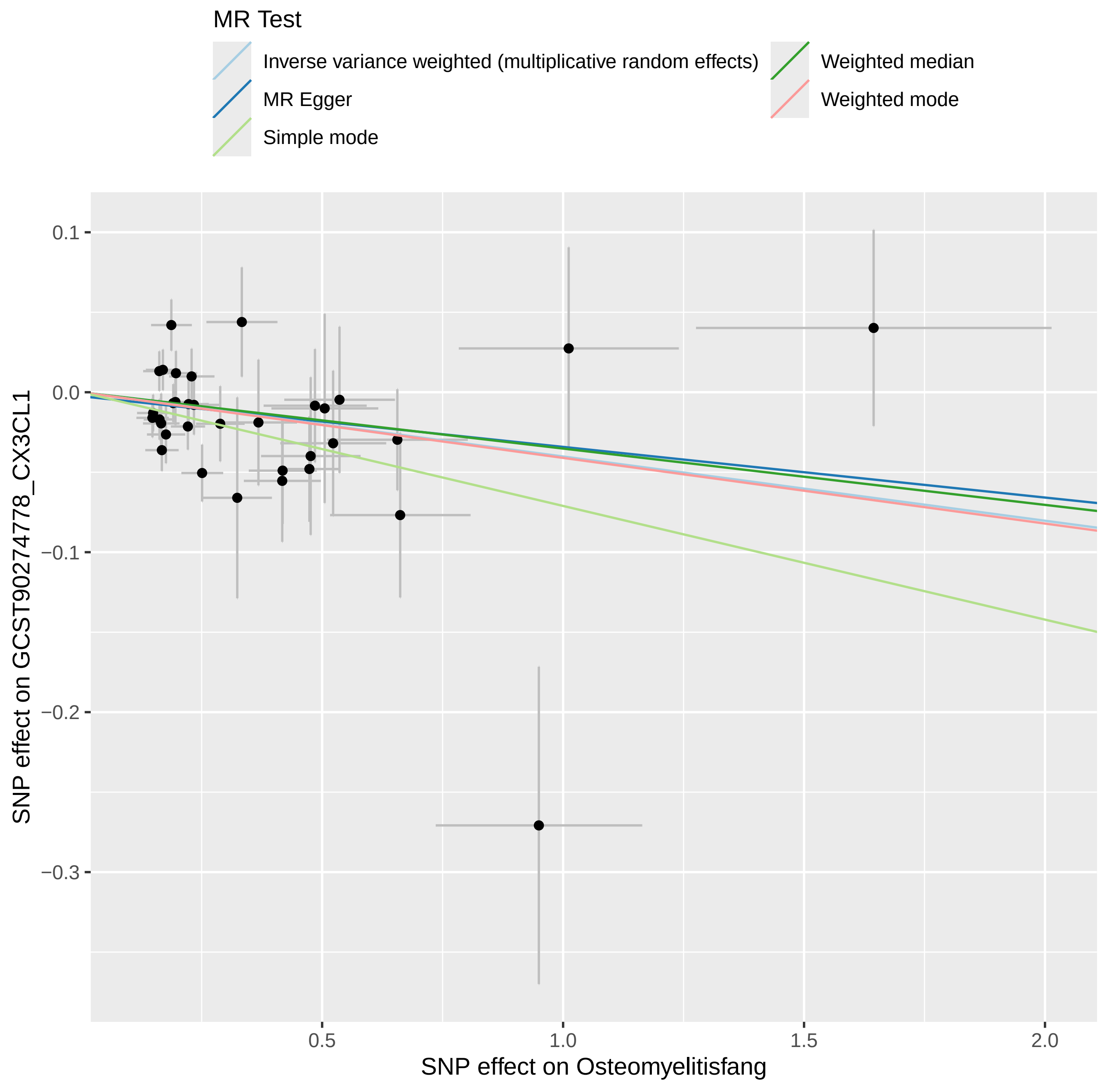
MR Test
Inverse variance weighted (multiplicative random effects)
MR Egger
Simple mode
Weighted median
Weighted mode
0.1
0.0
−0.1
−0.2
−0.3
0.5
1.0
1.5
2.0
SNP effect on GCST90274778_CX3CL1
SNP effect on Osteomyelitisfang

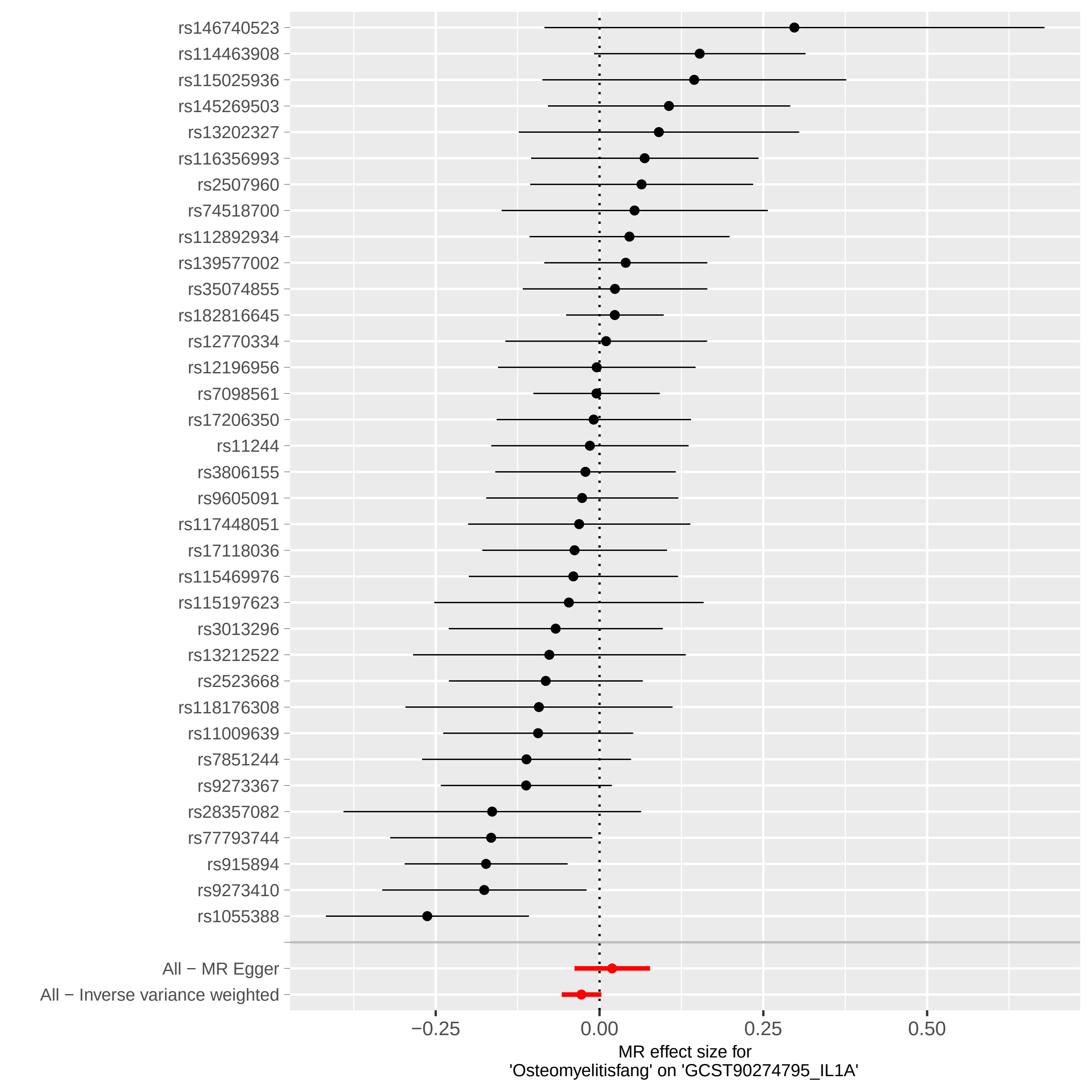

rs146740523
rs114463908
rs115025936
rs145269503
rs13202327
rs116356993
rs2507960
rs74518700
rs112892934
rs139577002
rs35074855
rs182816645
rs12770334
rs12196956
rs7098561
rs17206350
rs11244
rs3806155
rs9605091
rs117448051
rs17118036
rs115469976
rs115197623
rs3013296
rs13212522
rs2523668
rs118176308
rs11009639
rs7851244
rs9273367
rs28357082
rs77793744
rs915894
rs9273410
rs1055388
All − MR Egger
All − Inverse variance weighted
−0.25
0.00
0.25
0.50
MR effect size for
'Osteomyelitisfang' on 'GCST90274795_IL1A'

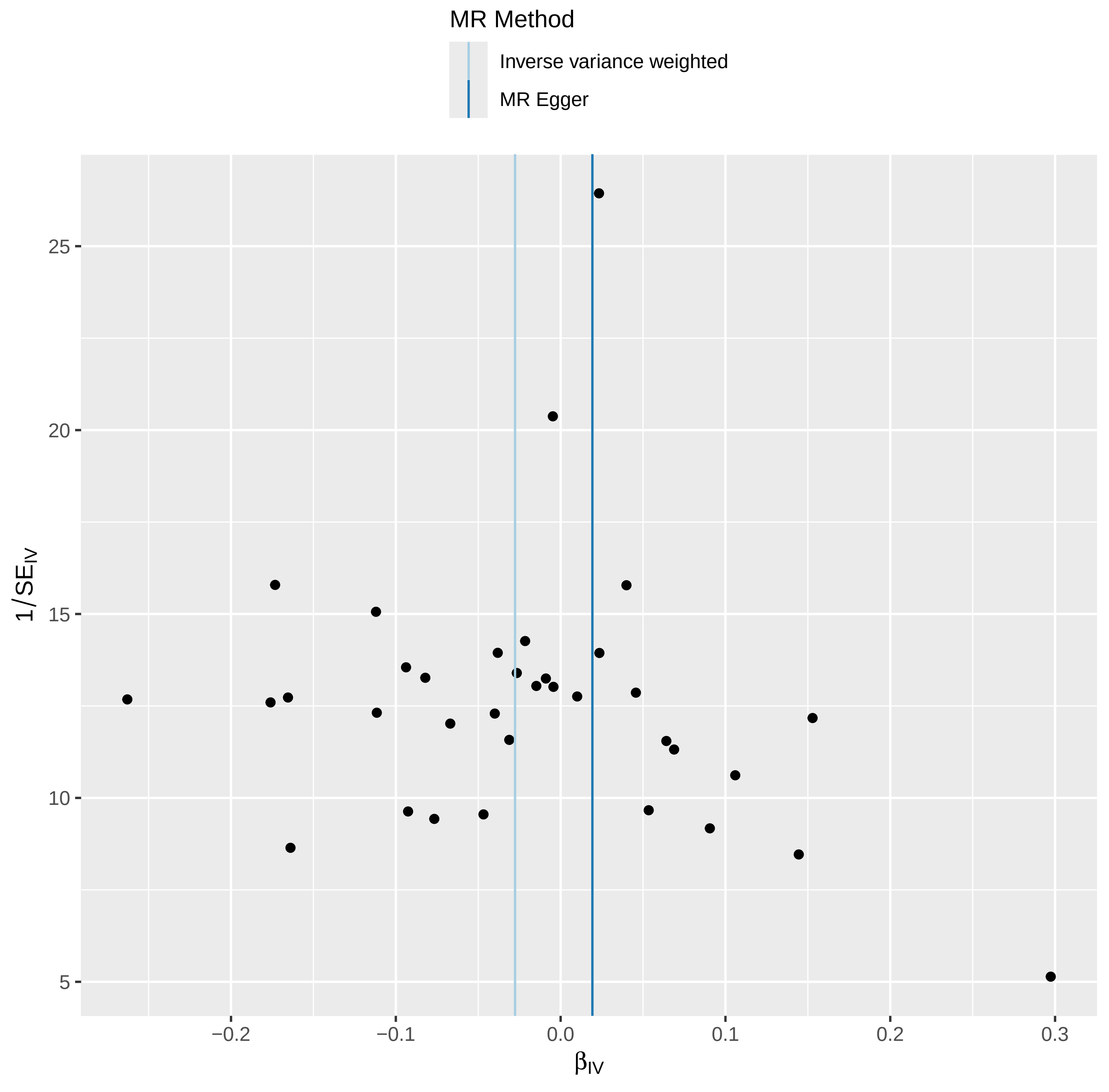

MR Method
Inverse variance weighted
MR Egger
25
20
15
10
5
1/SE_IV
−0.2
−0.1
0.0
0.1
0.2
0.3
β_IV

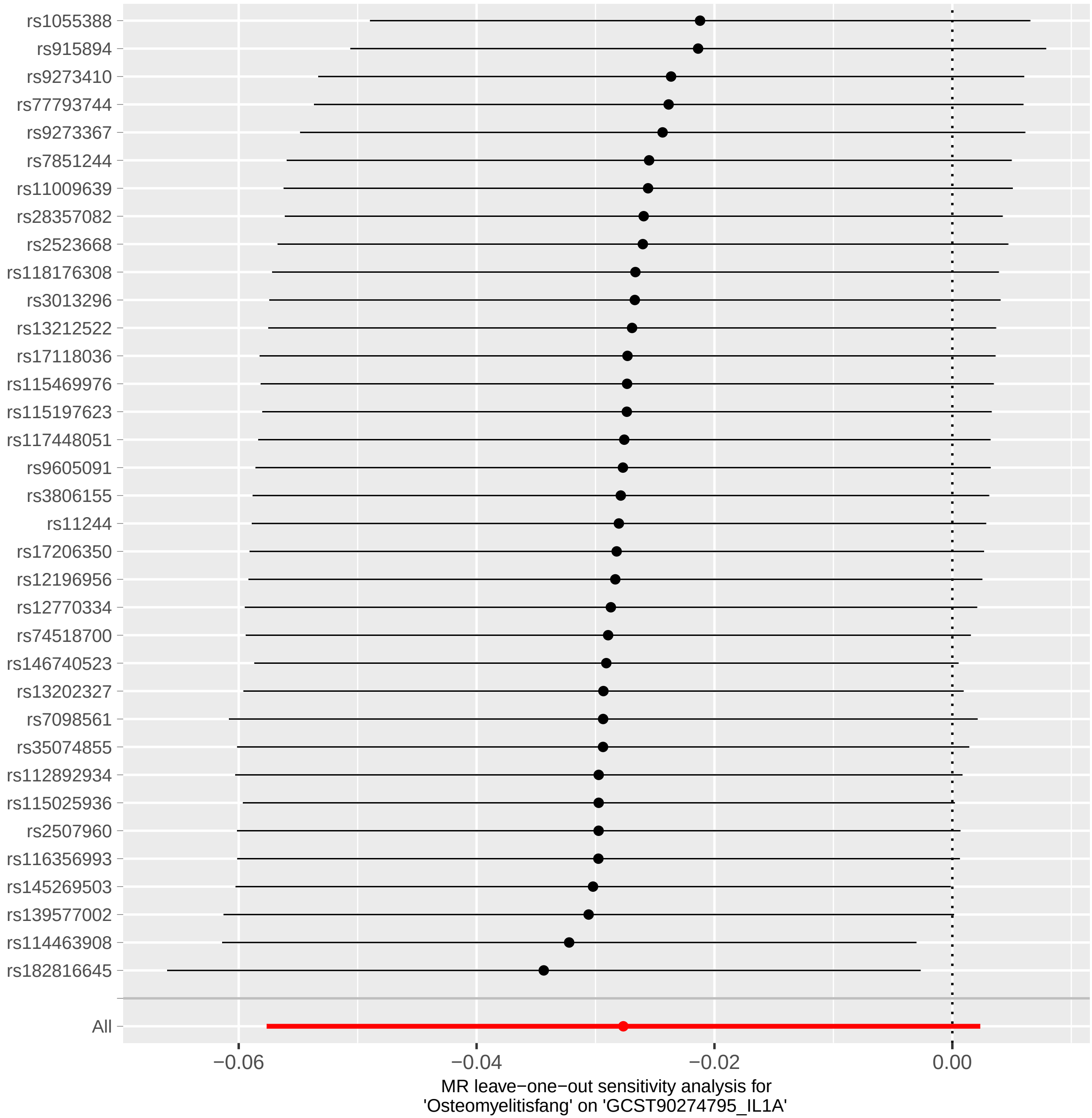

rs1055388
rs915894
rs9273410
rs77793744
rs9273367
rs7851244
rs11009639
rs28357082
rs2523668
rs118176308
rs3013296
rs13212522
rs17118036
rs115469976
rs115197623
rs117448051
rs9605091
rs3806155
rs11244
rs17206350
rs12196956
rs12770334
rs74518700
rs146740523
rs13202327
rs7098561
rs35074855
rs112892934
rs115025936
rs2507960
rs116356993
rs145269503
rs139577002
rs114463908
rs182816645
All
−0.06
−0.04
−0.02
0.00
MR leave−one−out sensitivity analysis for
'Osteomyelitisfang' on 'GCST90274795_IL1A'

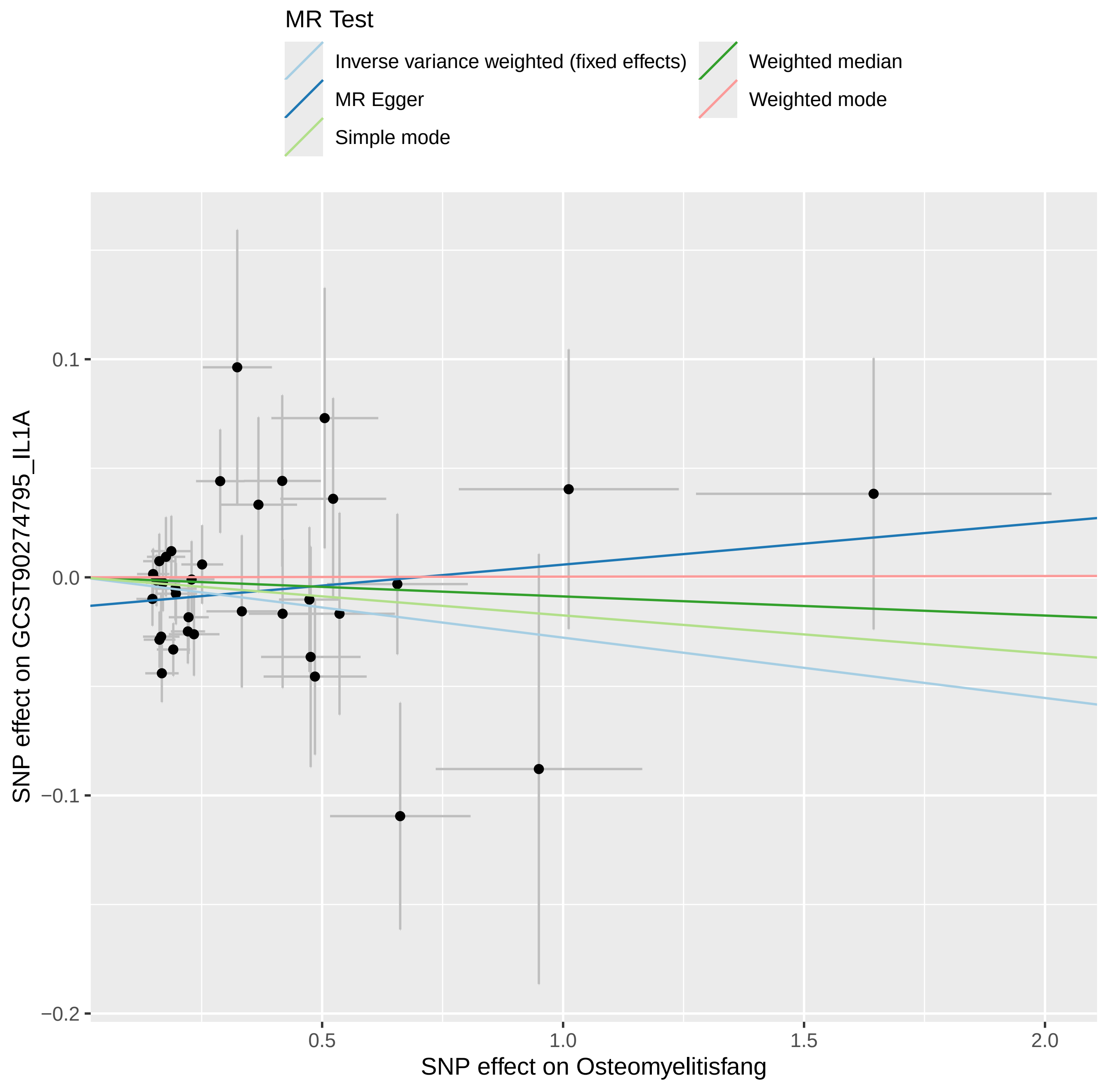

MR Test
Inverse variance weighted (fixed effects)
MR Egger
Simple mode
Weighted median
Weighted mode
SNP effect on GCST90274795_IL1A
0.1
0.0
−0.1
−0.2
0.5
1.0
1.5
2.0
SNP effect on Osteomyelitisfang

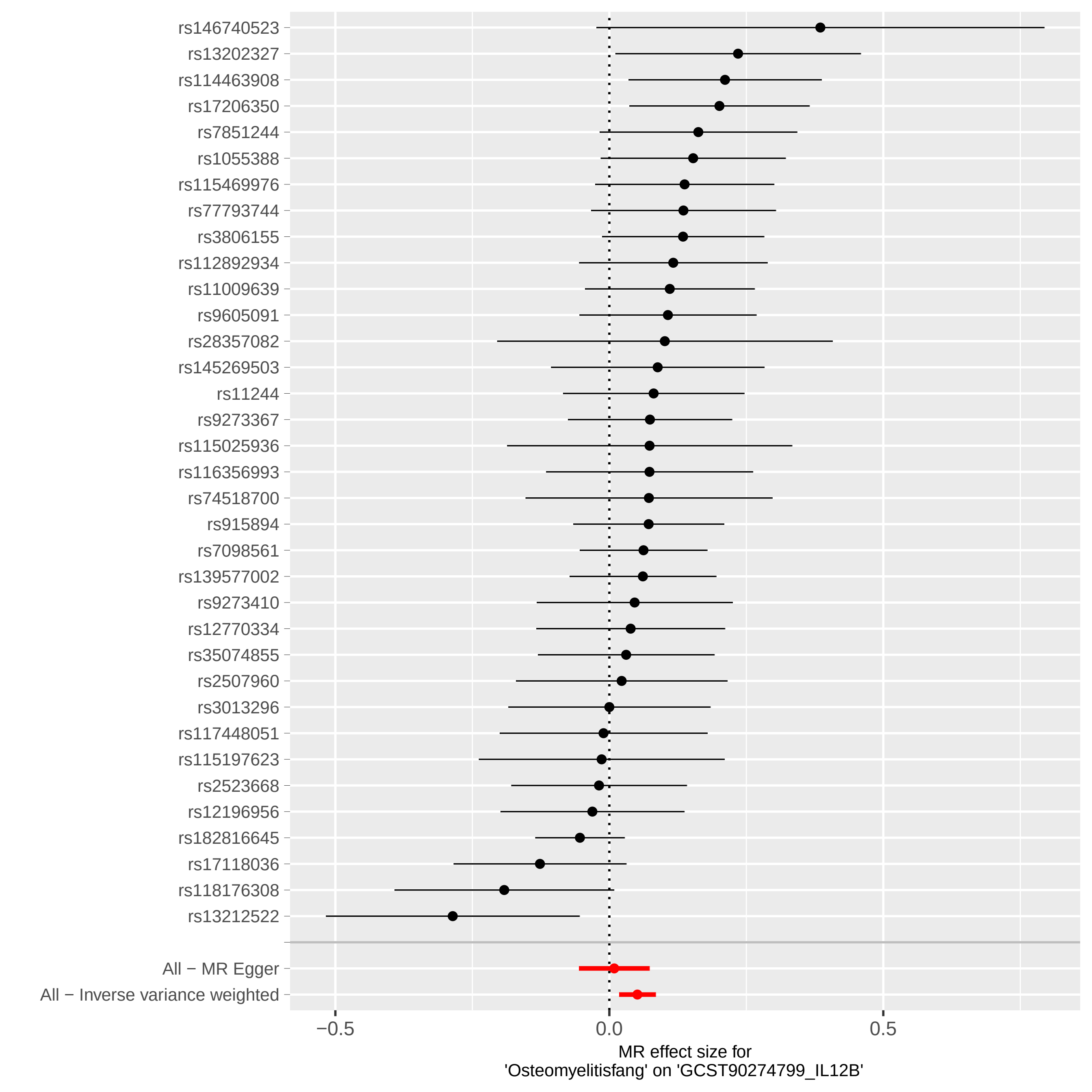
rs146740523
rs13202327
rs114463908
rs17206350
rs7851244
rs1055388
rs115469976
rs77793744
rs3806155
rs112892934
rs11009639
rs9605091
rs28357082
rs145269503
rs11244
rs9273367
rs115025936
rs116356993
rs74518700
rs915894
rs7098561
rs139577002
rs9273410
rs12770334
rs35074855
rs2507960
rs3013296
rs117448051
rs115197623
rs2523668
rs12196956
rs182816645
rs17118036
rs118176308
rs13212522
All − MR Egger
All − Inverse variance weighted
−0.5
0.0
0.5
MR effect size for
'Osteomyelitisfang' on 'GCST90274799_IL12B'

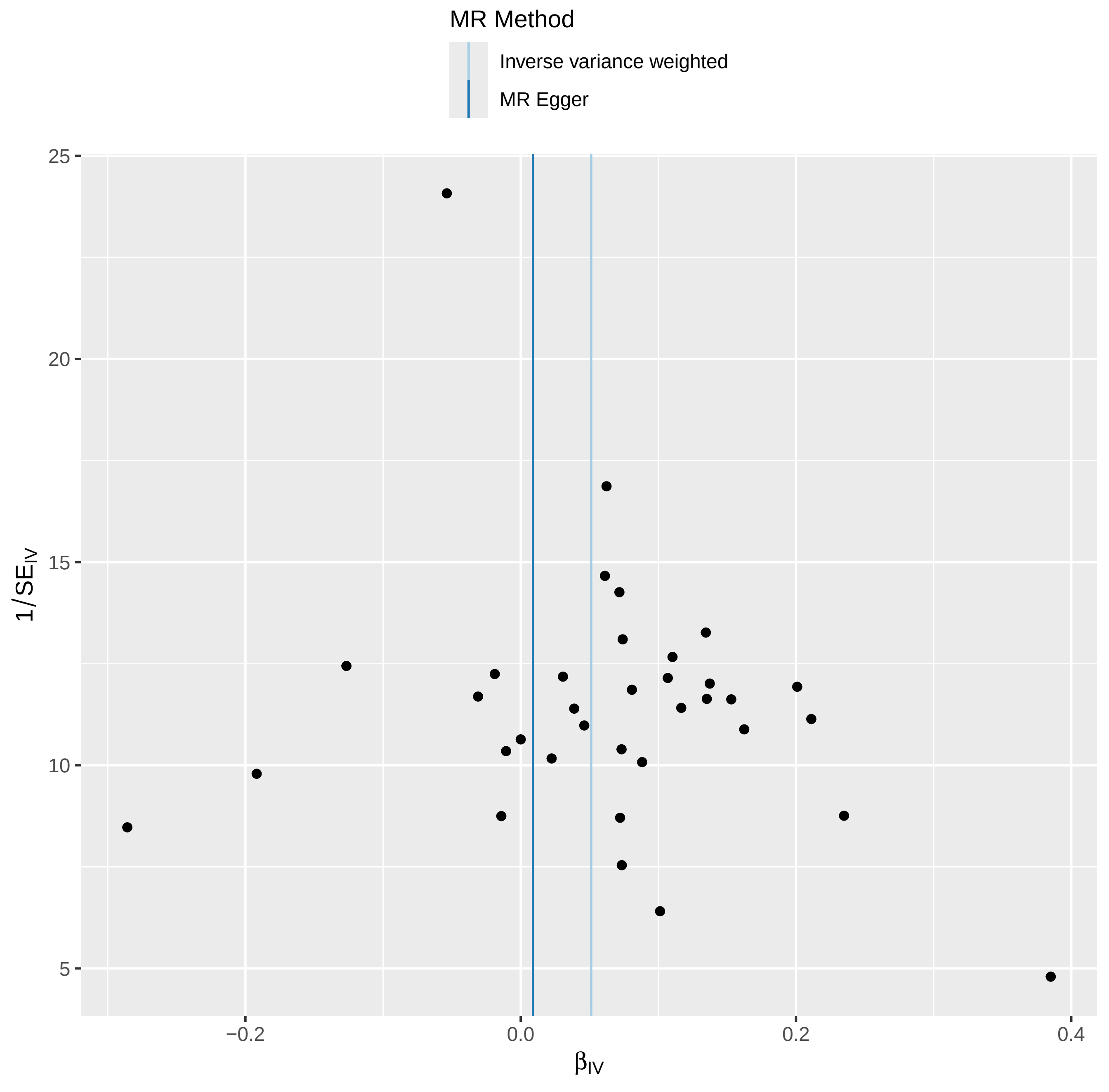
MR Method
Inverse variance weighted
MR Egger
25
20
15
10
5
$1/SE_{IV}$
−0.2
0.0
0.2
0.4
β_{IV}

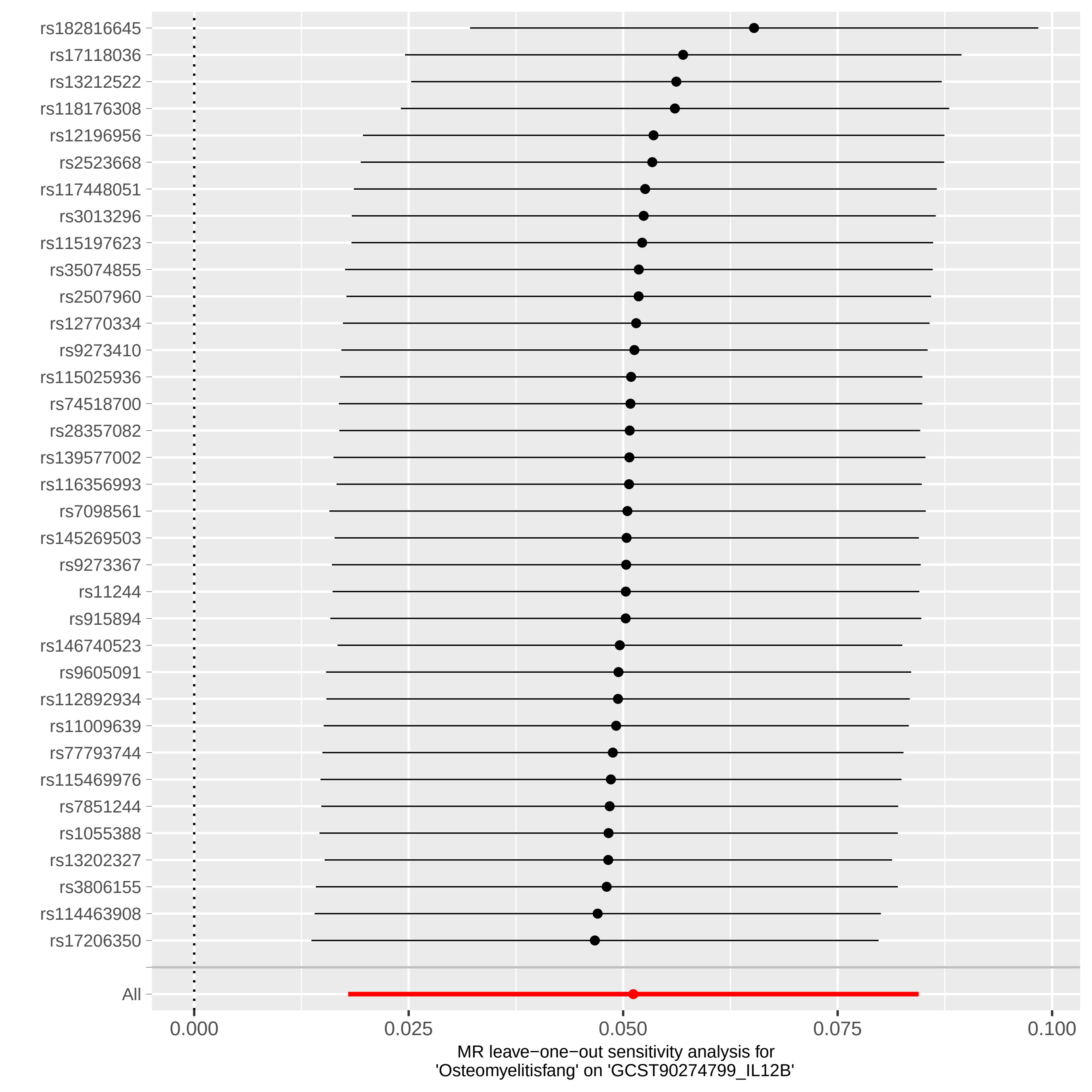
rs182816645
rs17118036
rs13212522
rs118176308
rs12196956
rs2523668
rs117448051
rs3013296
rs115197623
rs35074855
rs2507960
rs12770334
rs9273410
rs115025936
rs74518700
rs28357082
rs139577002
rs116356993
rs7098561
rs145269503
rs9273367
rs11244
rs915894
rs146740523
rs9605091
rs112892934
rs11009639
rs77793744
rs115469976
rs7851244
rs1055388
rs13202327
rs3806155
rs114463908
rs17206350
All
0.000
0.025
0.050
0.075
0.100
MR leave−one−out sensitivity analysis for
'Osteomyelitisfang' on 'GCST90274799_IL12B'

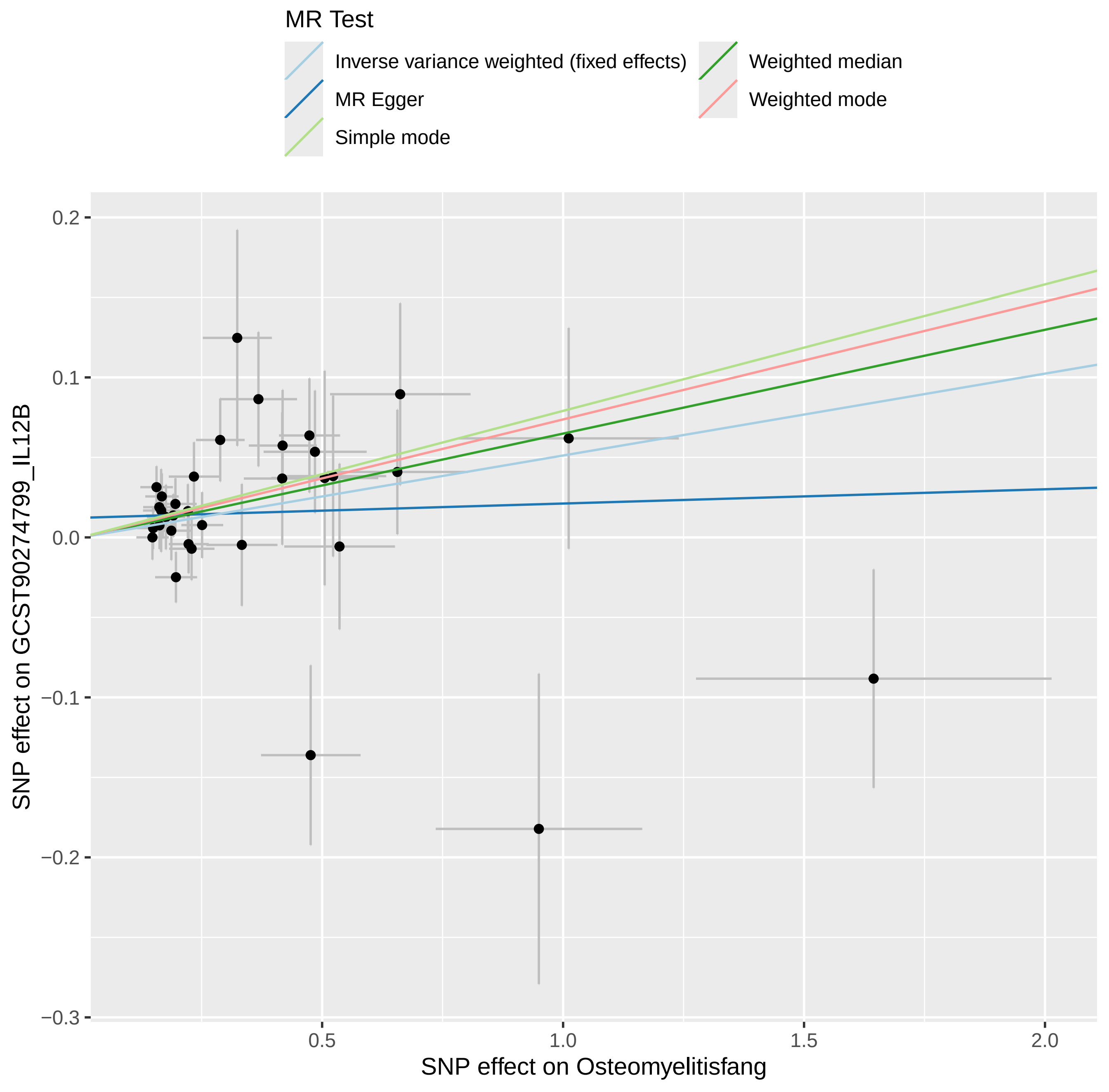

MR Test
Inverse variance weighted (fixed effects)
MR Egger
Simple mode
Weighted median
Weighted mode
SNP effect on GCST90274799_IL12B
0.2
0.1
0.0
−0.1
−0.2
−0.3
0.5
1.0
1.5
2.0
SNP effect on Osteomyelitisfang

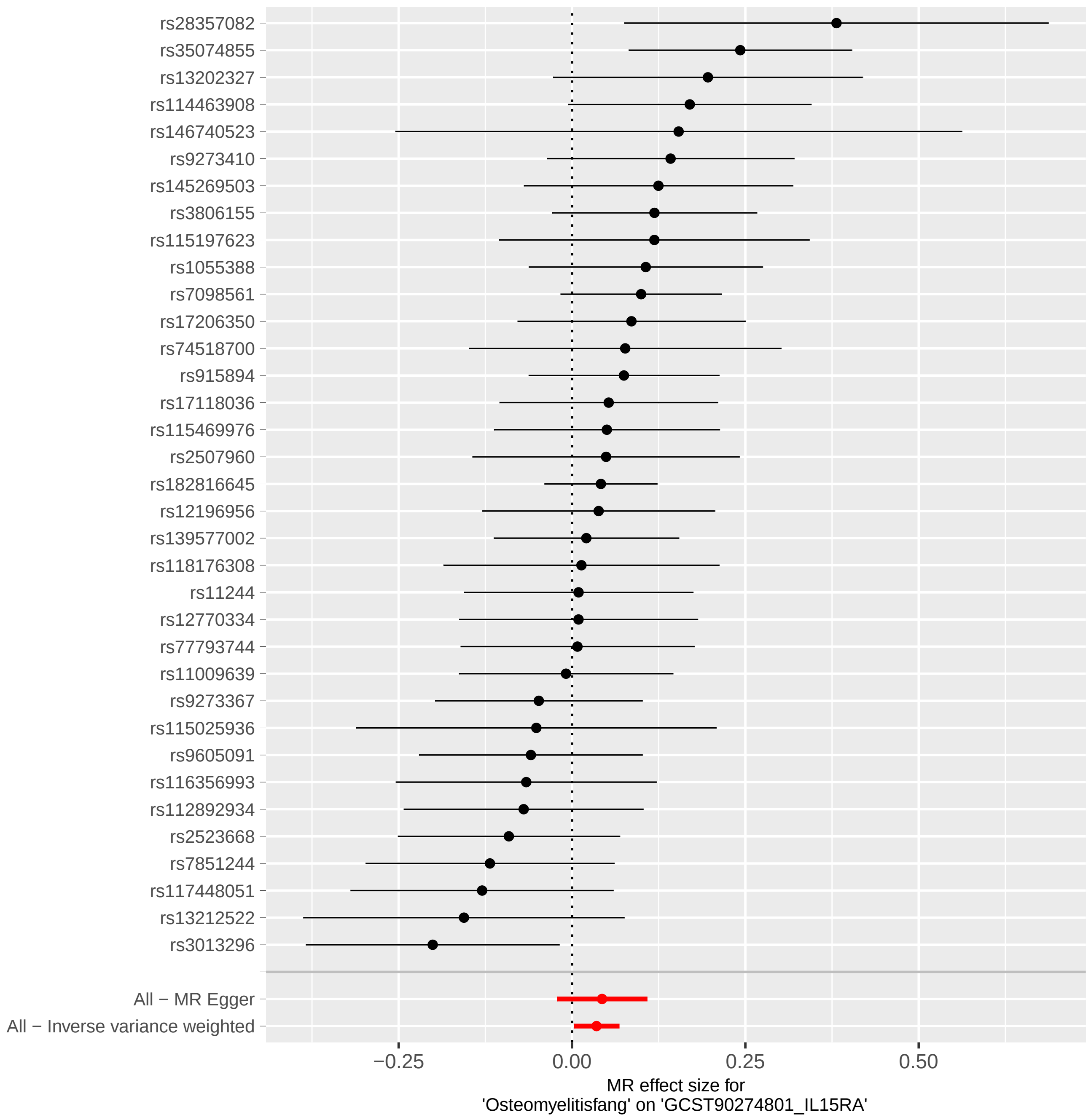

rs28357082
rs35074855
rs13202327
rs114463908
rs146740523
rs9273410
rs145269503
rs3806155
rs115197623
rs1055388
rs7098561
rs17206350
rs74518700
rs915894
rs17118036
rs115469976
rs2507960
rs182816645
rs12196956
rs139577002
rs118176308
rs11244
rs12770334
rs77793744
rs11009639
rs9273367
rs115025936
rs9605091
rs116356993
rs112892934
rs2523668
rs7851244
rs117448051
rs13212522
rs3013296
All − MR Egger
All − Inverse variance weighted
−0.25
0.00
0.25
0.50
MR effect size for
'Osteomyelitisfang' on 'GCST90274801_IL15RA'

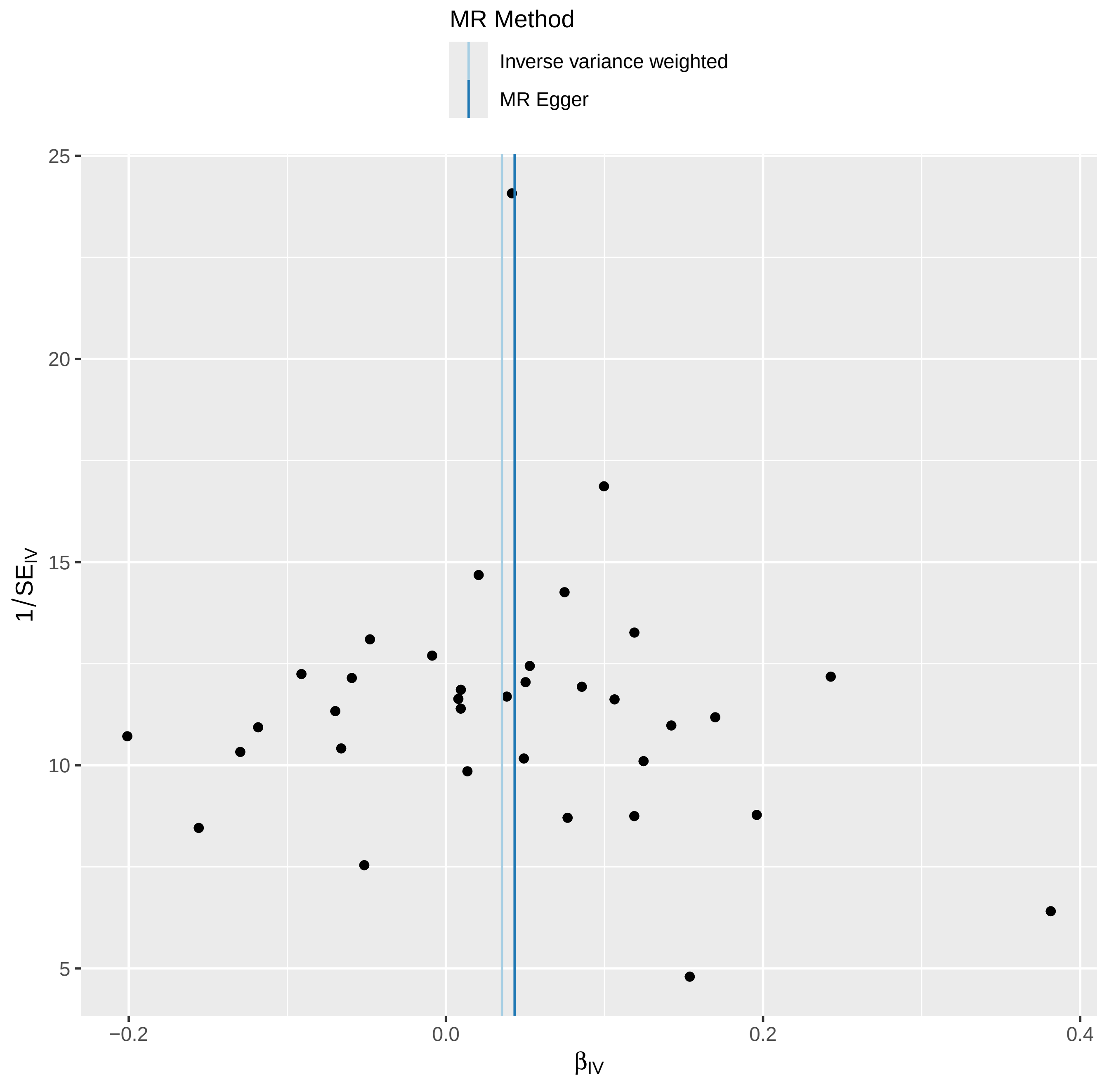

MR Method
Inverse variance weighted
MR Egger
25
20
15
10
5
1/SE$_{IV}$
−0.2
0.0
0.2
0.4
β$_{IV}$

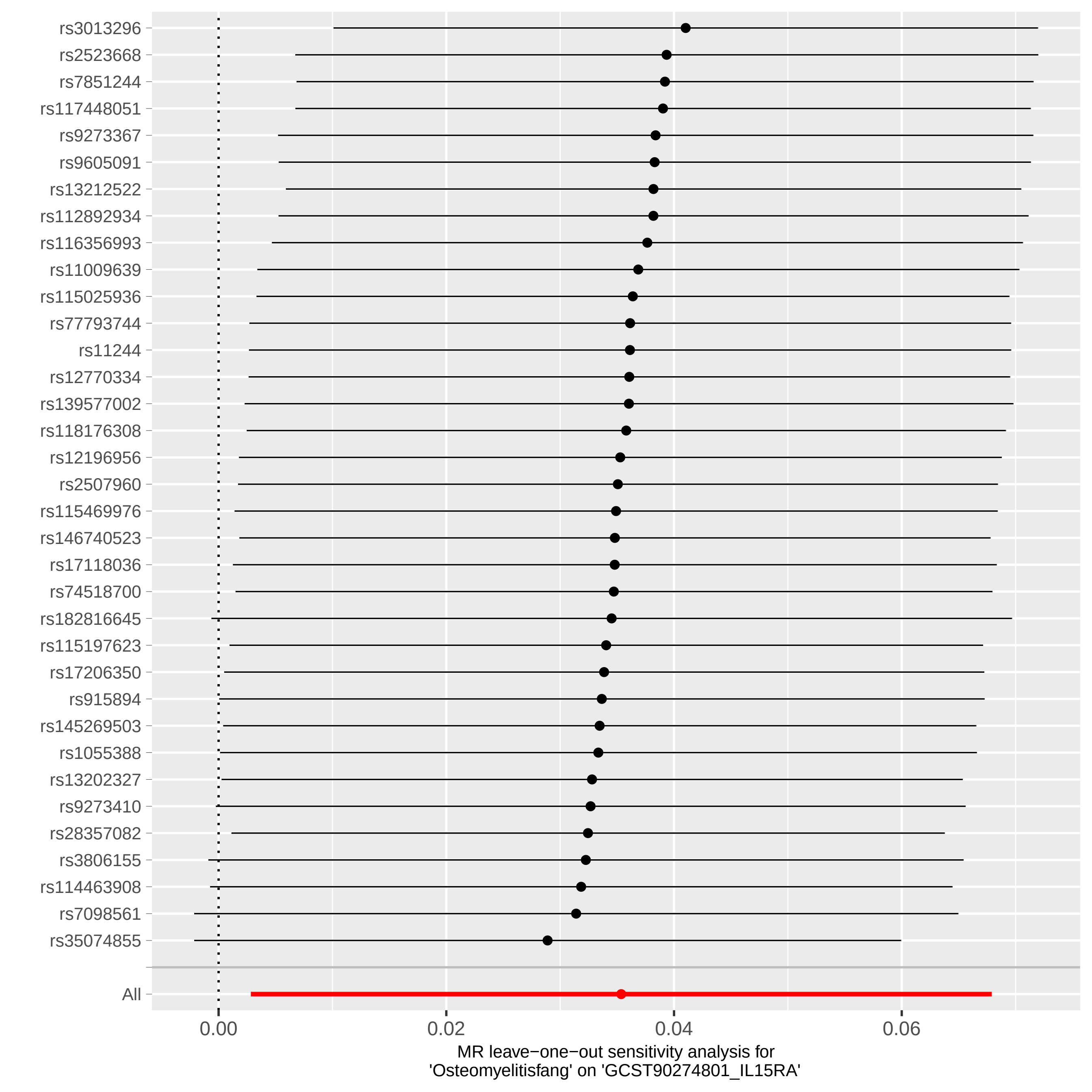
rs3013296
rs2523668
rs7851244
rs117448051
rs9273367
rs9605091
rs13212522
rs112892934
rs116356993
rs11009639
rs115025936
rs77793744
rs11244
rs12770334
rs139577002
rs118176308
rs12196956
rs2507960
rs115469976
rs146740523
rs17118036
rs74518700
rs182816645
rs115197623
rs17206350
rs915894
rs145269503
rs1055388
rs13202327
rs9273410
rs28357082
rs3806155
rs114463908
rs7098561
rs35074855
All
0.00
0.02
0.04
0.06
MR leave−one−out sensitivity analysis for
'Osteomyelitisfang' on 'GCST90274801_IL15RA'

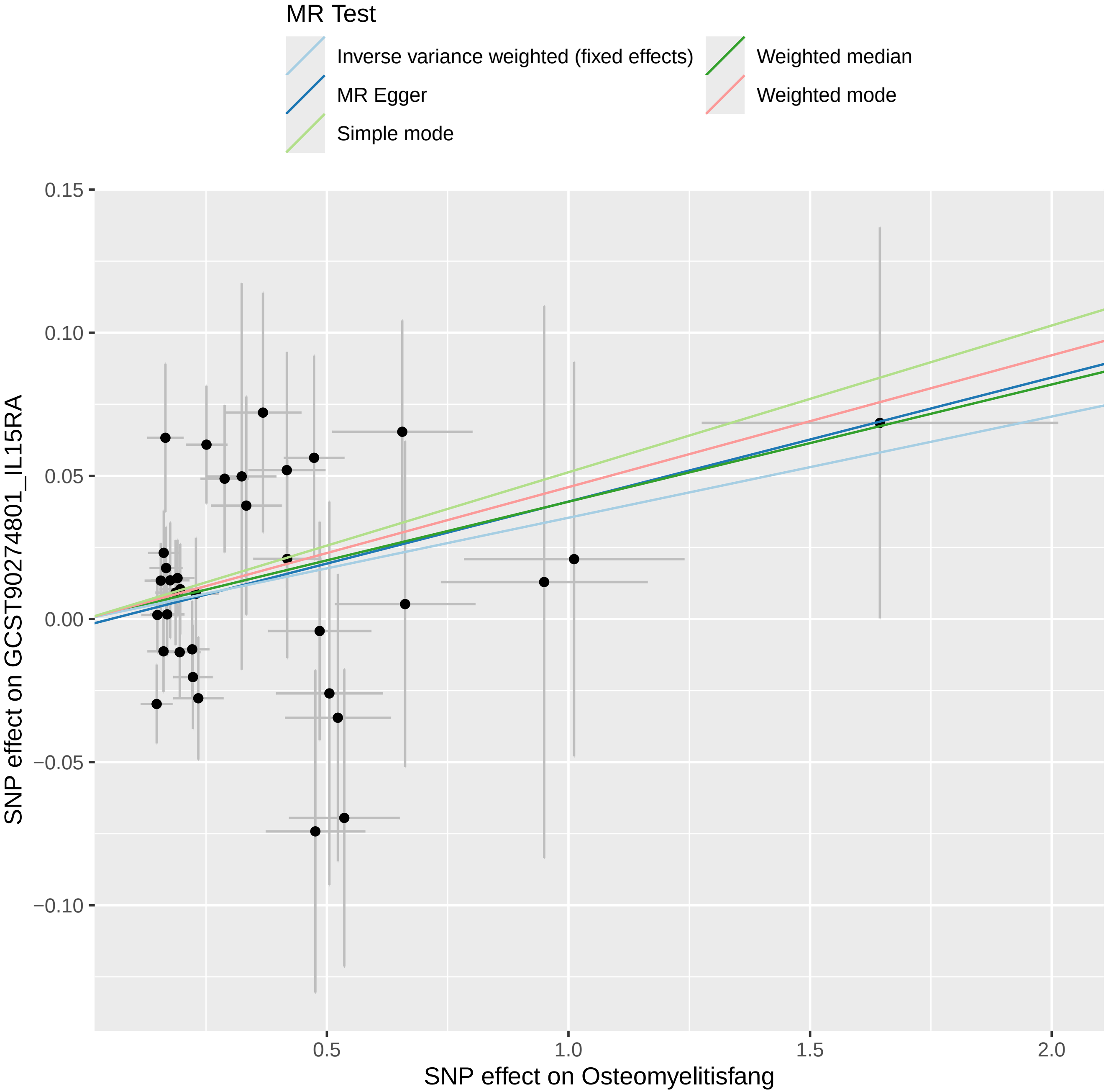
MR Test
Inverse variance weighted (fixed effects)
MR Egger
Simple mode
Weighted median
Weighted mode
0.15
0.10
0.05
0.00
−0.05
−0.10
SNP effect on GCST90274801_IL15RA
0.5
1.0
1.5
2.0
SNP effect on Osteomyelitisfang

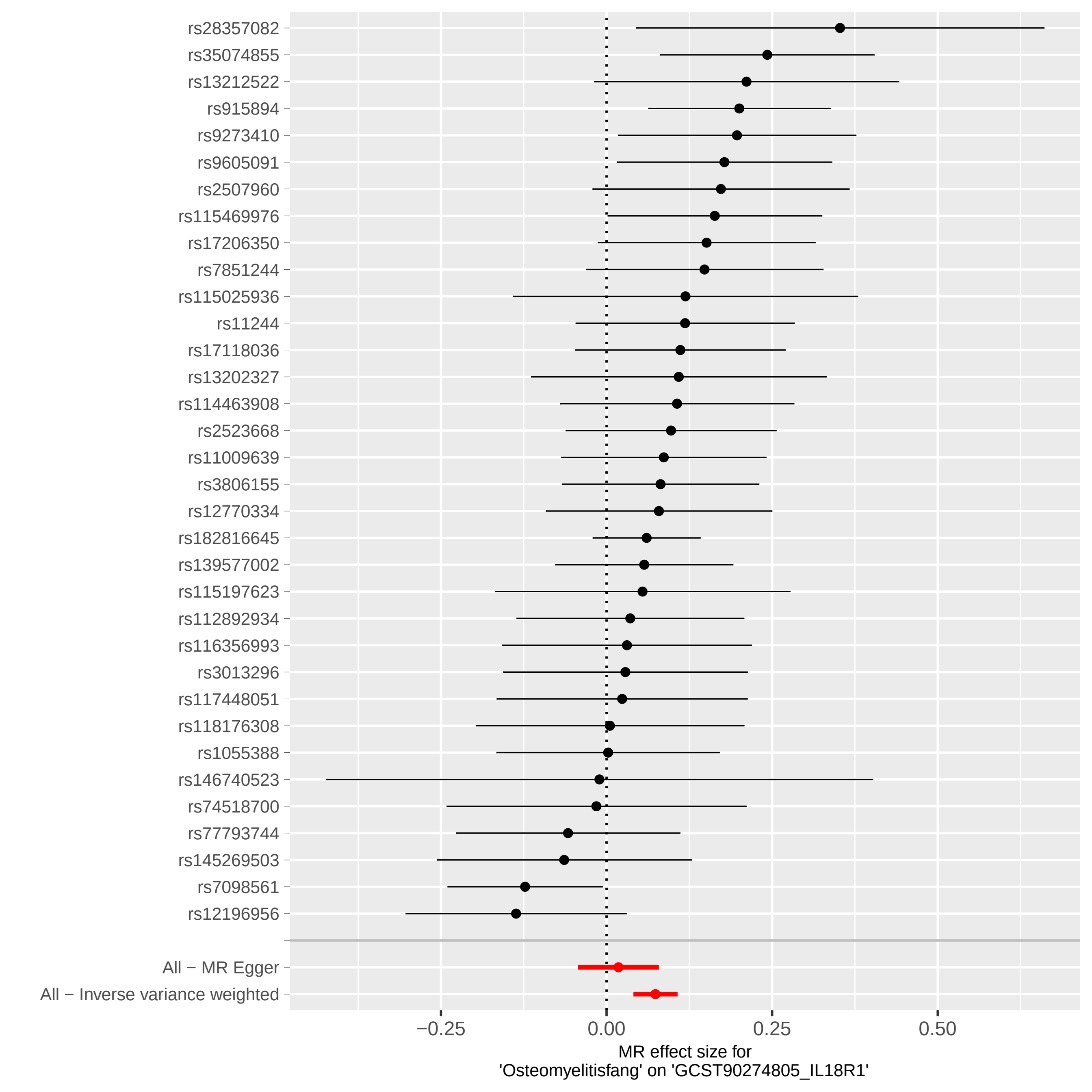

rs28357082
rs35074855
rs13212522
rs915894
rs9273410
rs9605091
rs2507960
rs115469976
rs17206350
rs7851244
rs115025936
rs11244
rs17118036
rs13202327
rs114463908
rs2523668
rs11009639
rs3806155
rs12770334
rs182816645
rs139577002
rs115197623
rs112892934
rs116356993
rs3013296
rs117448051
rs118176308
rs1055388
rs146740523
rs74518700
rs77793744
rs145269503
rs7098561
rs12196956
All − MR Egger
All − Inverse variance weighted
−0.25
0.00
0.25
0.50
MR effect size for
'Osteomyelitisfang' on 'GCST90274805_IL18R1'

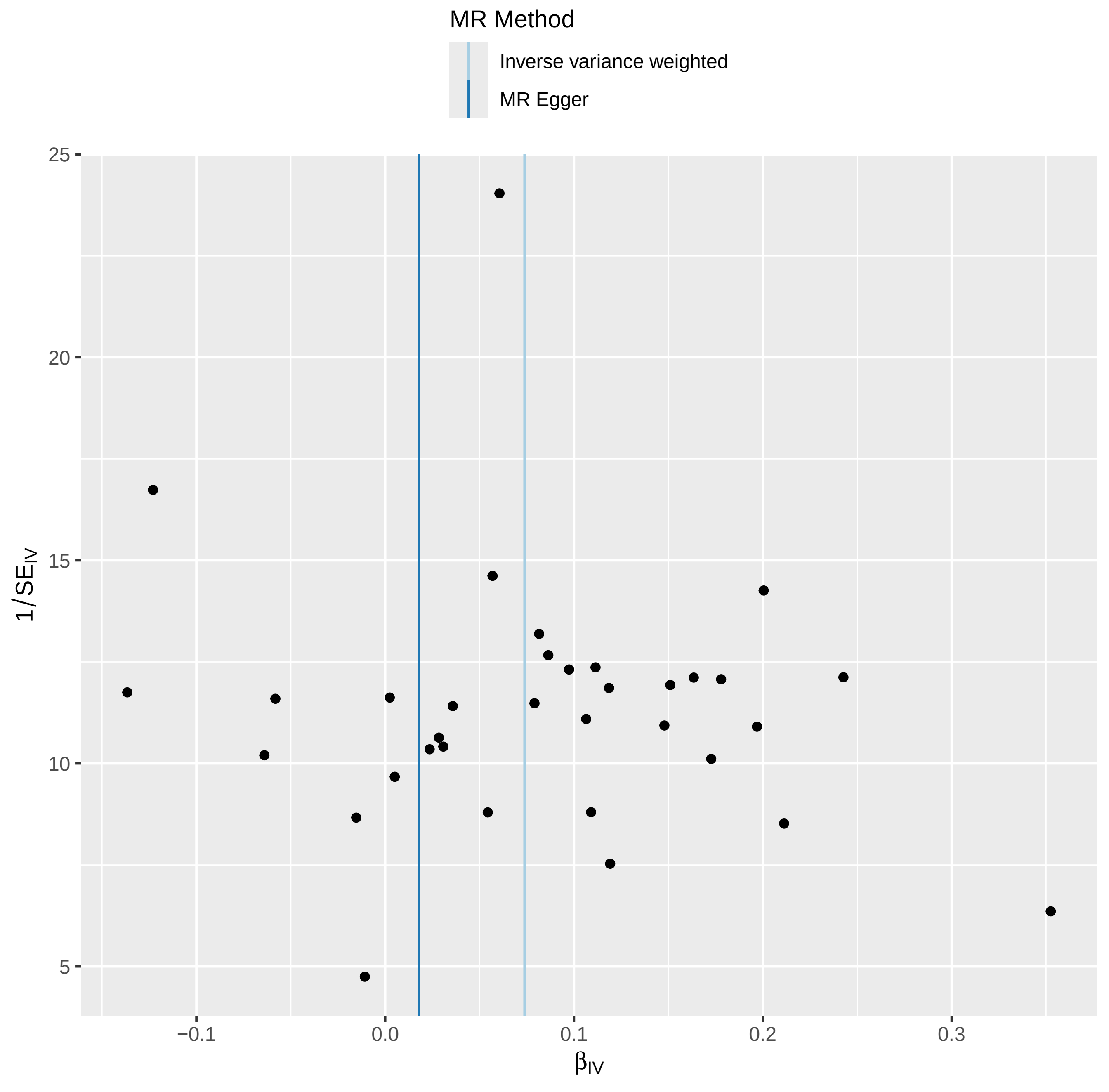

MR Method
Inverse variance weighted
MR Egger
25
20
15
10
5
1/SE_IV
−0.1
0.0
0.1
0.2
0.3
β_IV

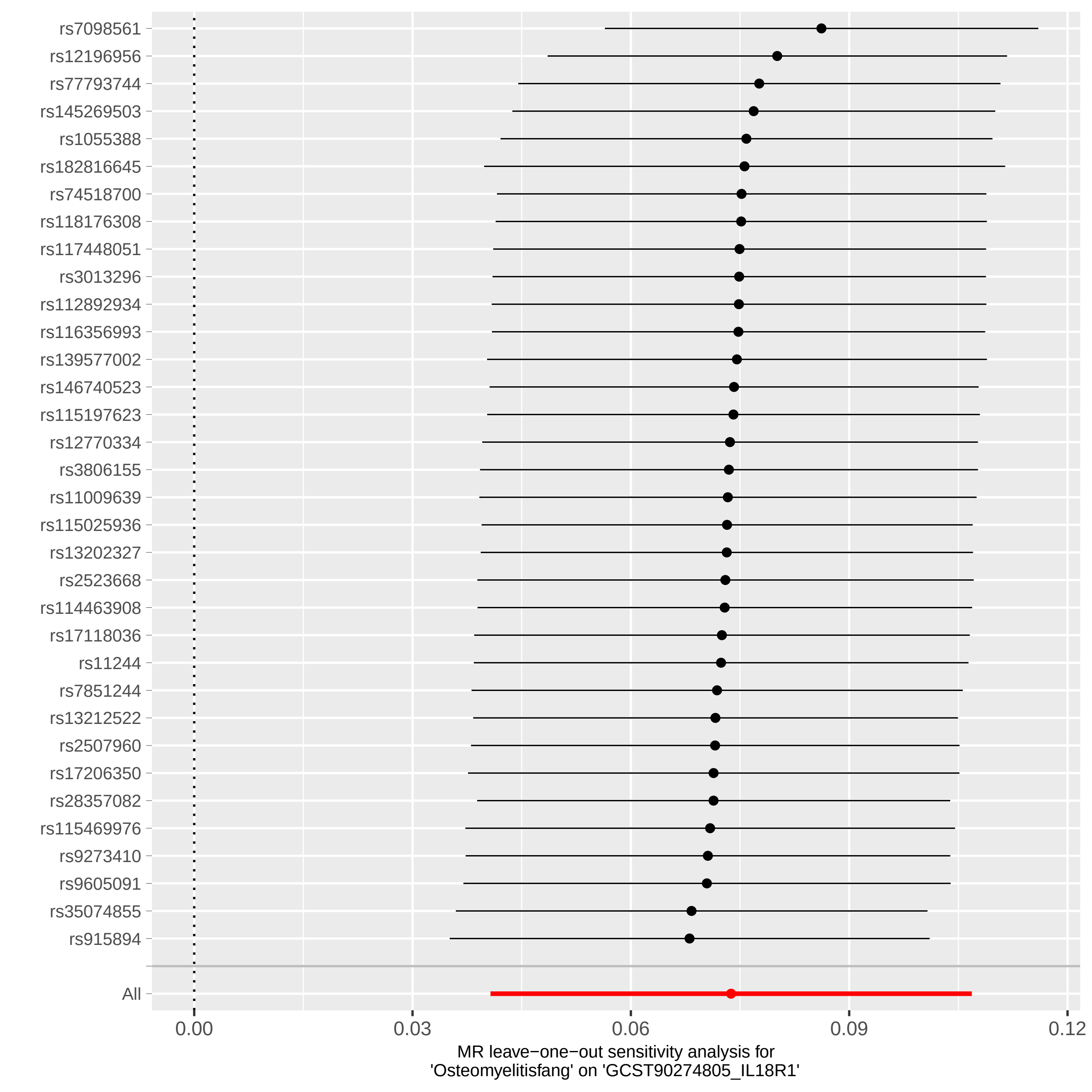

rs7098561
rs12196956
rs77793744
rs145269503
rs1055388
rs182816645
rs74518700
rs118176308
rs117448051
rs3013296
rs112892934
rs116356993
rs139577002
rs146740523
rs115197623
rs12770334
rs3806155
rs11009639
rs115025936
rs13202327
rs2523668
rs114463908
rs17118036
rs11244
rs7851244
rs13212522
rs2507960
rs17206350
rs28357082
rs115469976
rs9273410
rs9605091
rs35074855
rs915894
All
0.00
0.03
0.06
0.09
0.12
MR leave−one−out sensitivity analysis for
'Osteomyelitisfang' on 'GCST90274805_IL18R1'

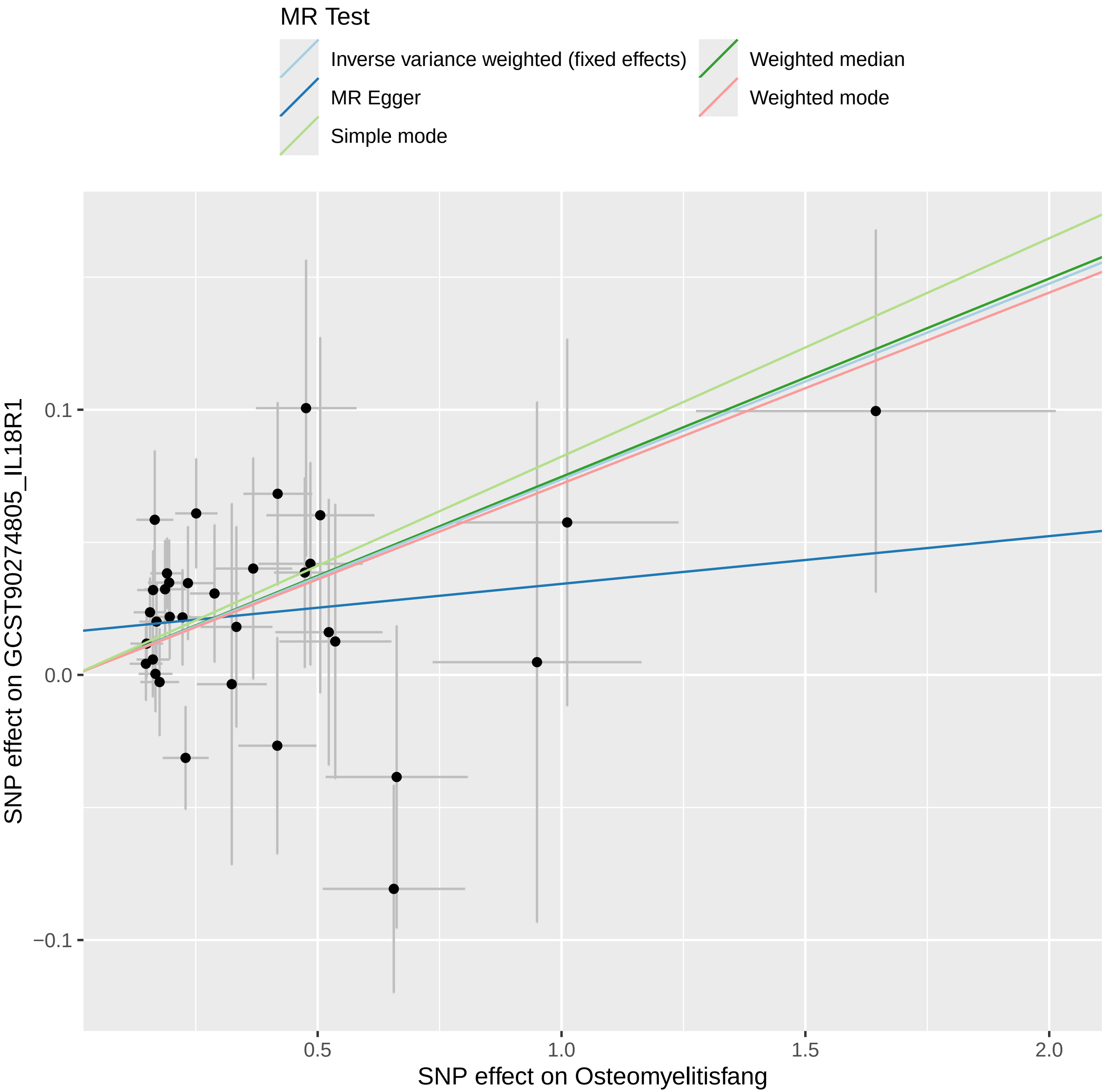

MR Test
Inverse variance weighted (fixed effects)
MR Egger
Simple mode
Weighted median
Weighted mode
SNP effect on GCST90274805_IL18R1
0.1
0.0
−0.1
0.5
1.0
1.5
2.0
SNP effect on Osteomyelitisfang

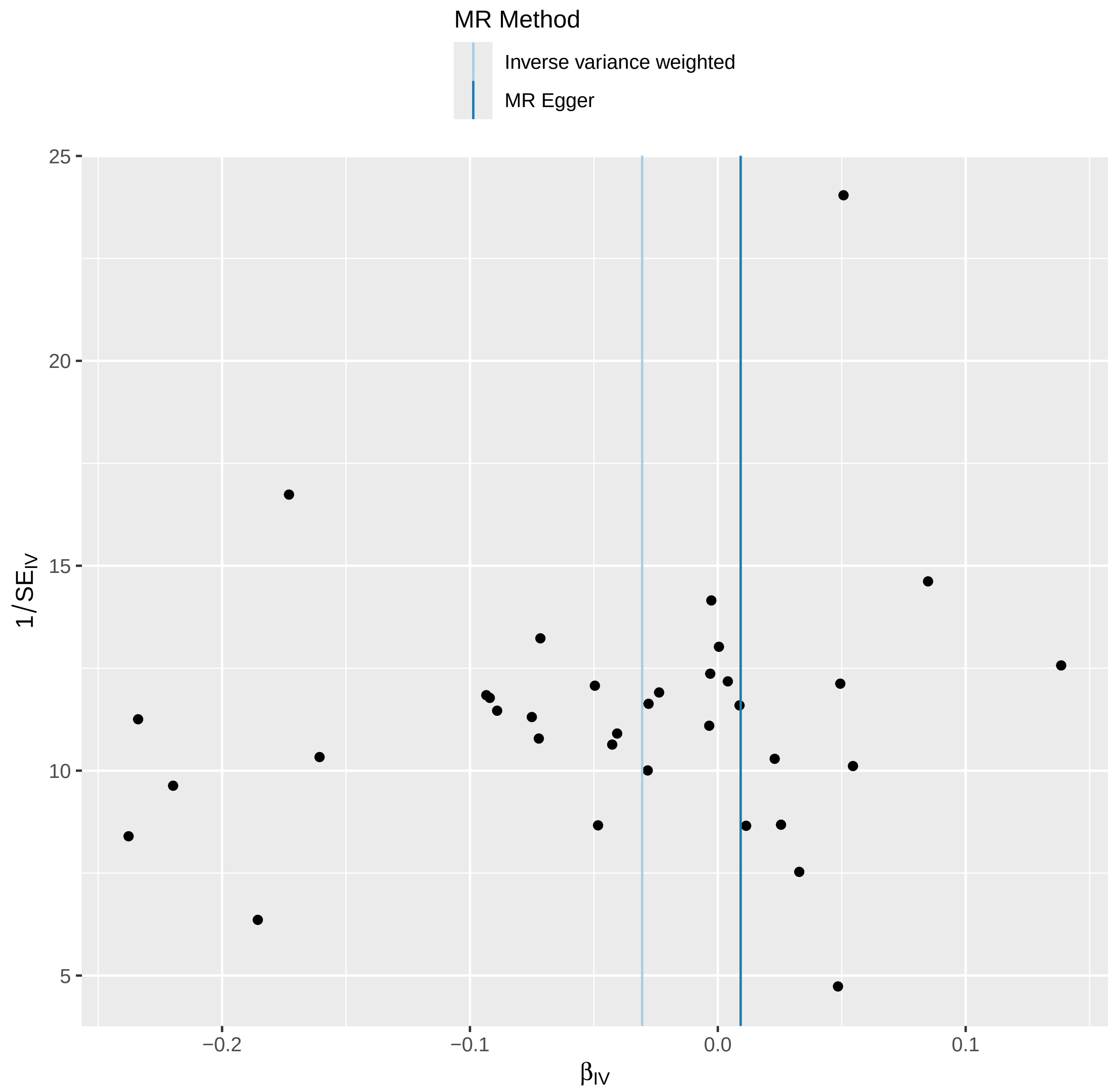

MR Method
Inverse variance weighted
MR Egger
25
20
15
10
5
$1/SE_{IV}$
−0.2
−0.1
0.0
0.1
β_{IV}

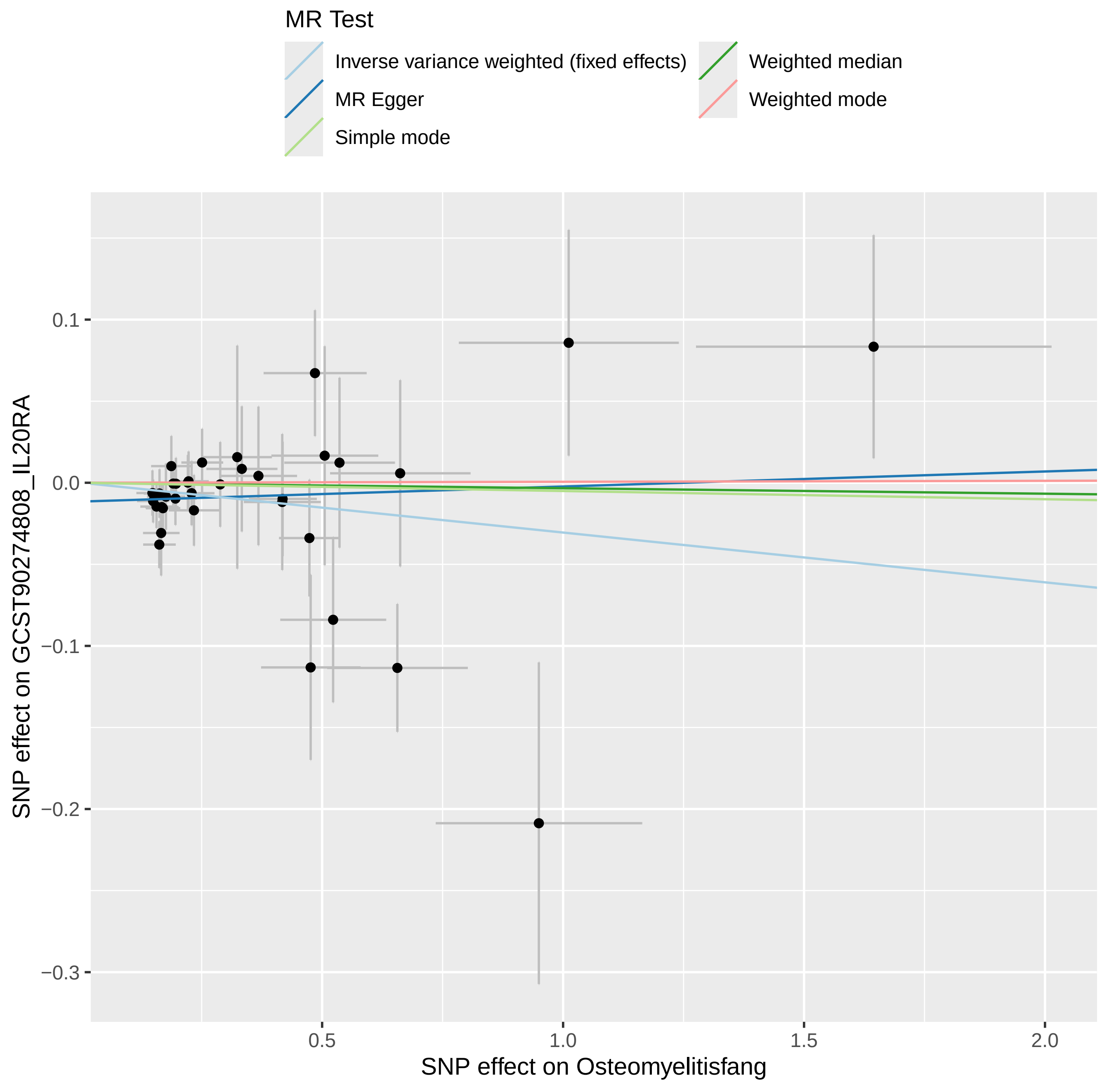
MR Test
Inverse variance weighted (fixed effects)
MR Egger
Simple mode
Weighted median
Weighted mode
0.1
0.0
−0.1
−0.2
−0.3
0.5
1.0
1.5
2.0
SNP effect on GCST90274808_IL20RA
SNP effect on Osteomyelitisfang

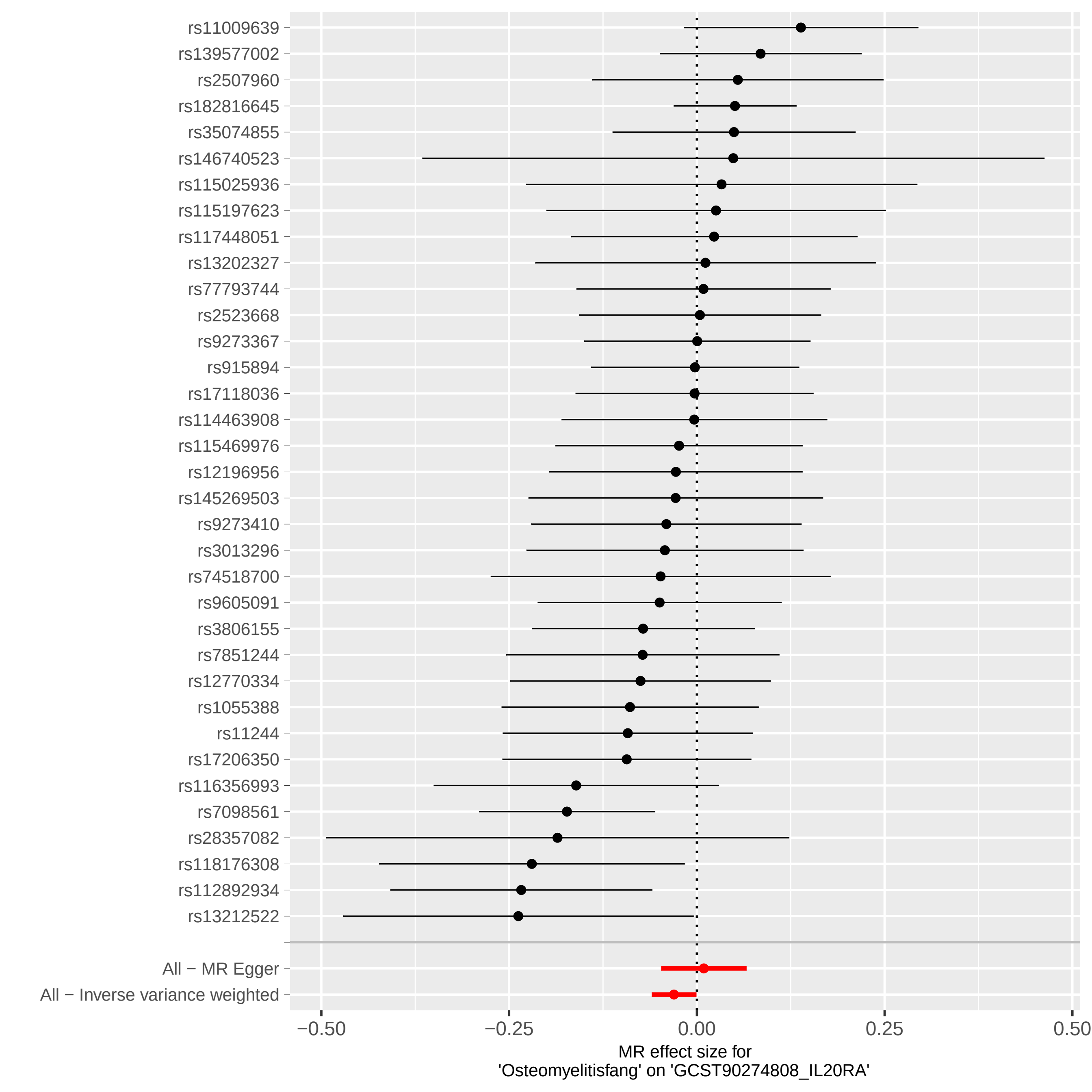

rs11009639
rs139577002
rs2507960
rs182816645
rs35074855
rs146740523
rs115025936
rs115197623
rs117448051
rs13202327
rs77793744
rs2523668
rs9273367
rs915894
rs17118036
rs114463908
rs115469976
rs12196956
rs145269503
rs9273410
rs3013296
rs74518700
rs9605091
rs3806155
rs7851244
rs12770334
rs1055388
rs11244
rs17206350
rs116356993
rs7098561
rs28357082
rs118176308
rs112892934
rs13212522
All − MR Egger
All − Inverse variance weighted
−0.50
−0.25
0.00
0.25
0.50
MR effect size for
'Osteomyelitisfang' on 'GCST90274808_IL20RA'

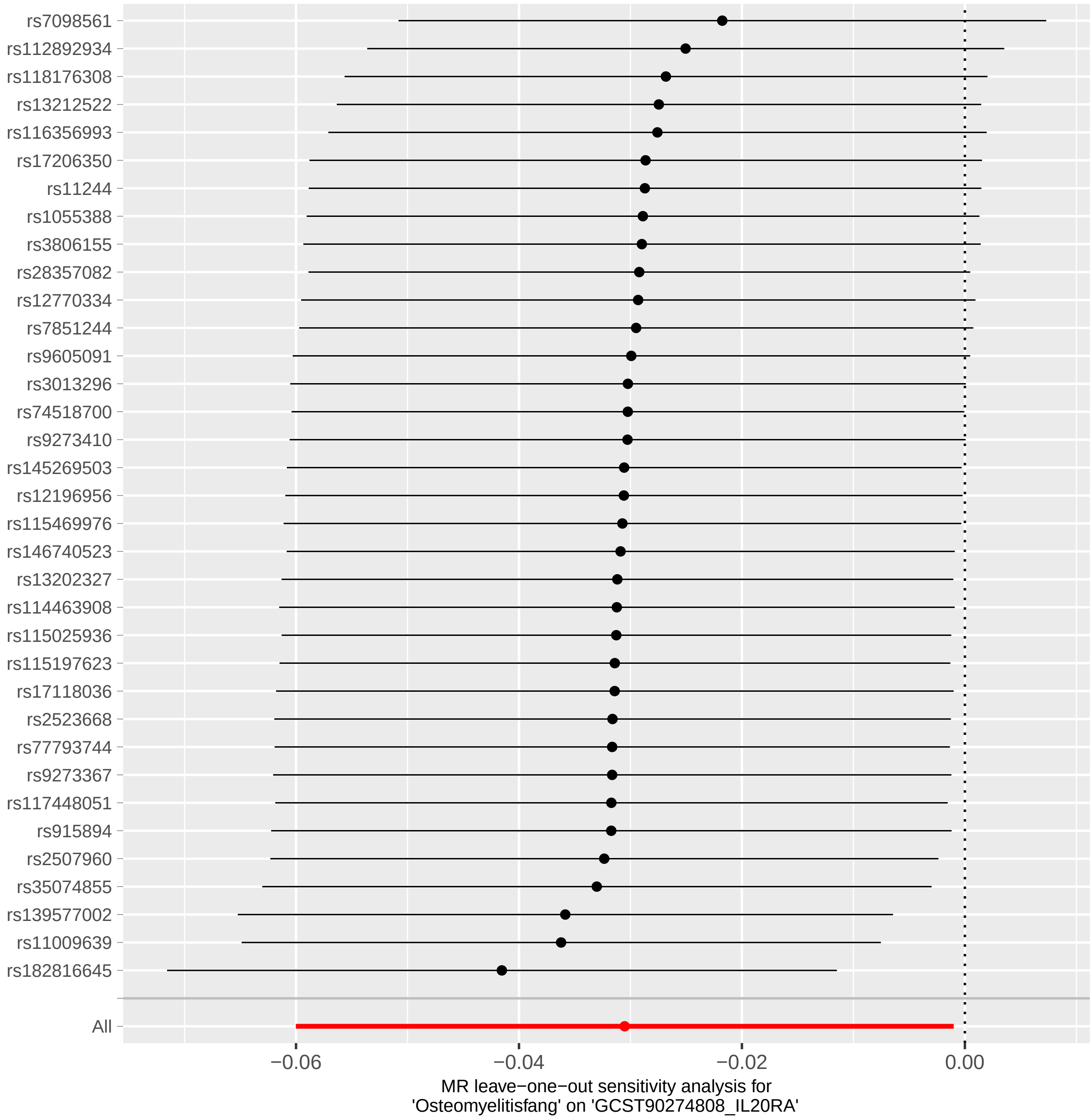

rs7098561
rs112892934
rs118176308
rs13212522
rs116356993
rs17206350
rs11244
rs1055388
rs3806155
rs28357082
rs12770334
rs7851244
rs9605091
rs3013296
rs74518700
rs9273410
rs145269503
rs12196956
rs115469976
rs146740523
rs13202327
rs114463908
rs115025936
rs115197623
rs17118036
rs2523668
rs77793744
rs9273367
rs117448051
rs915894
rs2507960
rs35074855
rs139577002
rs11009639
rs182816645
All
−0.06
−0.04
−0.02
0.00
MR leave−one−out sensitivity analysis for
'Osteomyelitisfang' on 'GCST90274808_IL20RA'

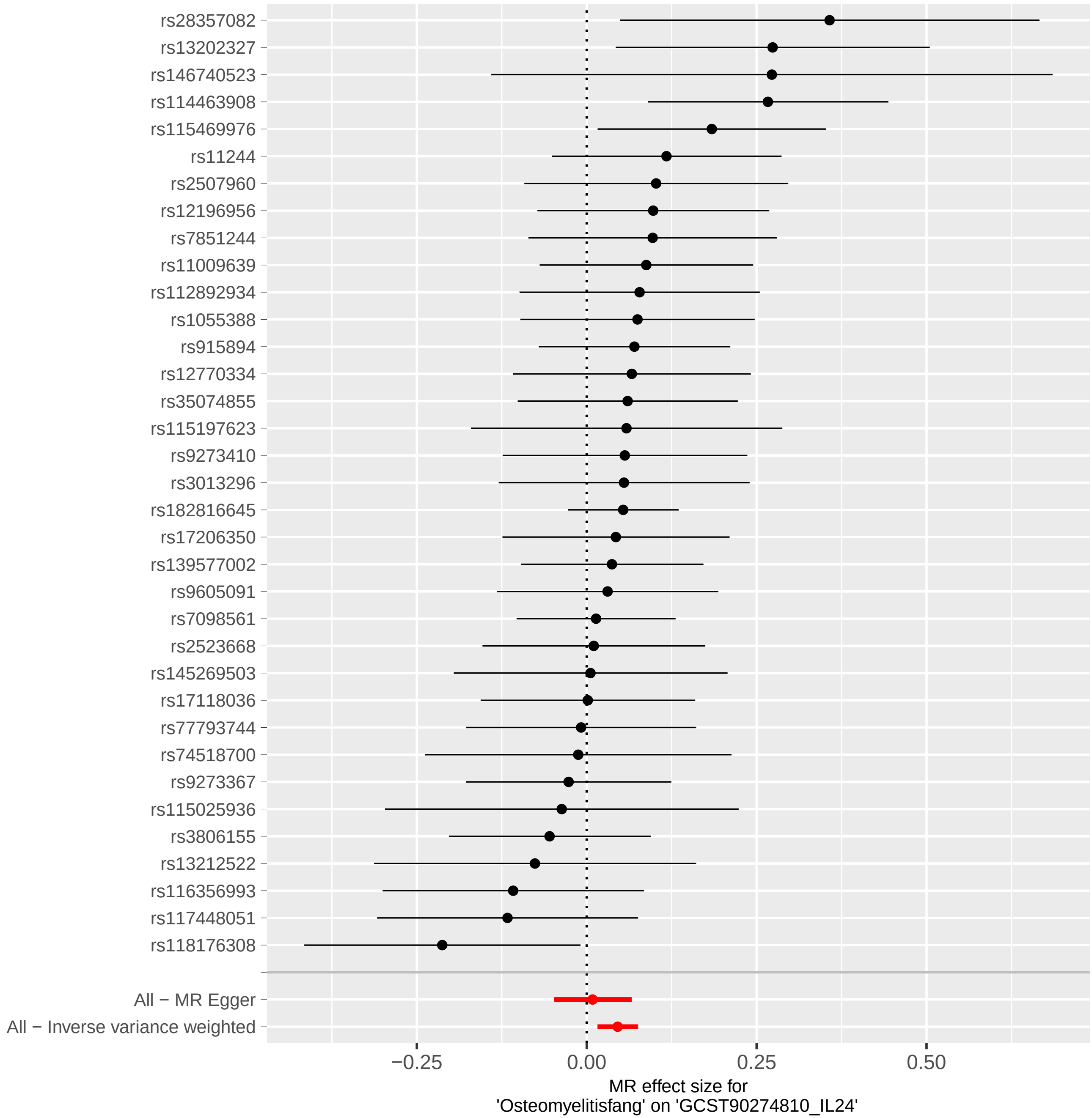

rs28357082
rs13202327
rs146740523
rs114463908
rs115469976
rs11244
rs2507960
rs12196956
rs7851244
rs11009639
rs112892934
rs1055388
rs915894
rs12770334
rs35074855
rs115197623
rs9273410
rs3013296
rs182816645
rs17206350
rs139577002
rs9605091
rs7098561
rs2523668
rs145269503
rs17118036
rs77793744
rs74518700
rs9273367
rs115025936
rs3806155
rs13212522
rs116356993
rs117448051
rs118176308
All − MR Egger
All − Inverse variance weighted
−0.25
0.00
0.25
0.50
MR effect size for
'Osteomyelitisfang' on 'GCST90274810_IL24'

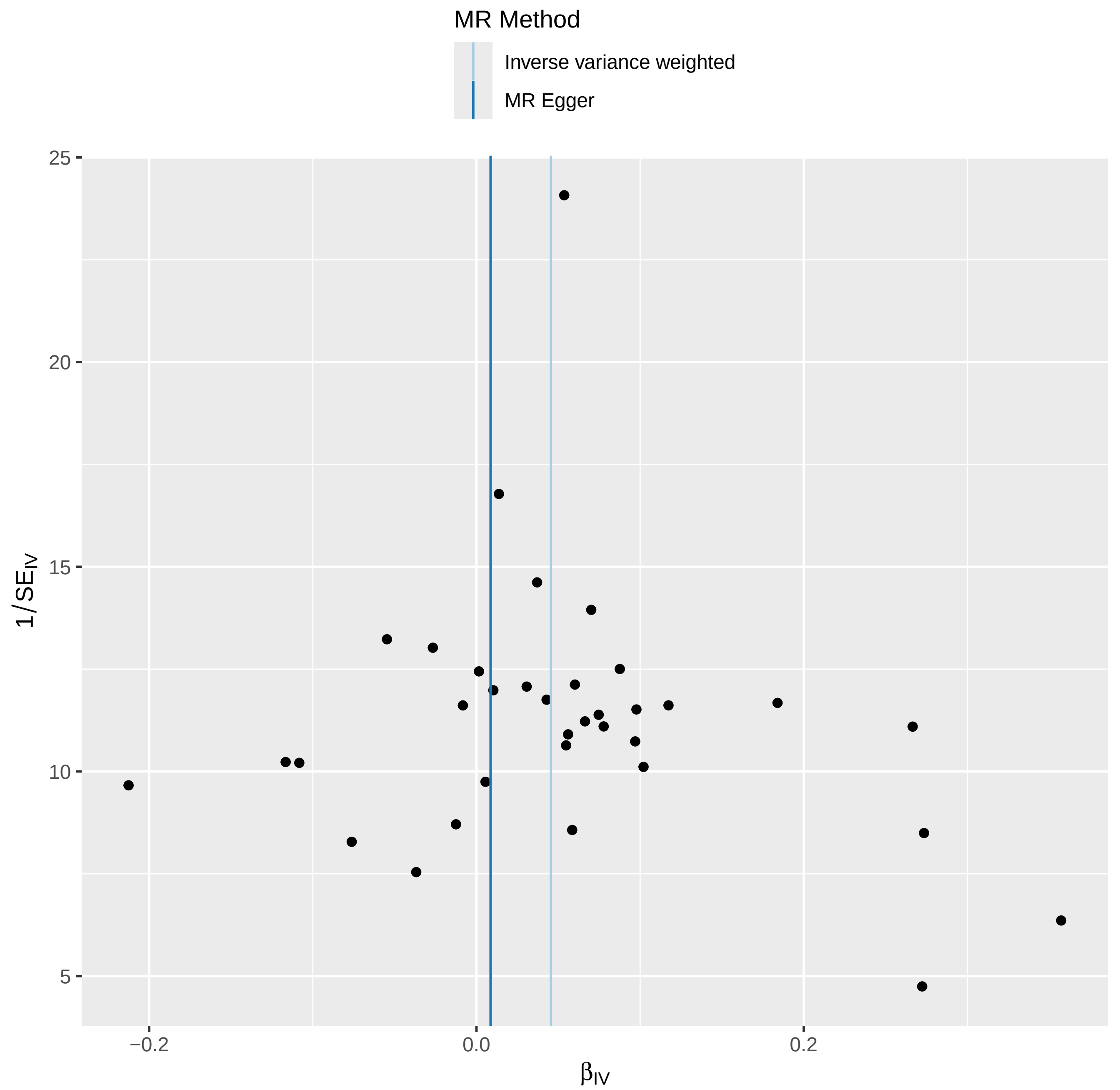

MR Method
Inverse variance weighted
MR Egger
25
20
15
10
5
$1/SE_{IV}$
−0.2
0.0
0.2
β_{IV}

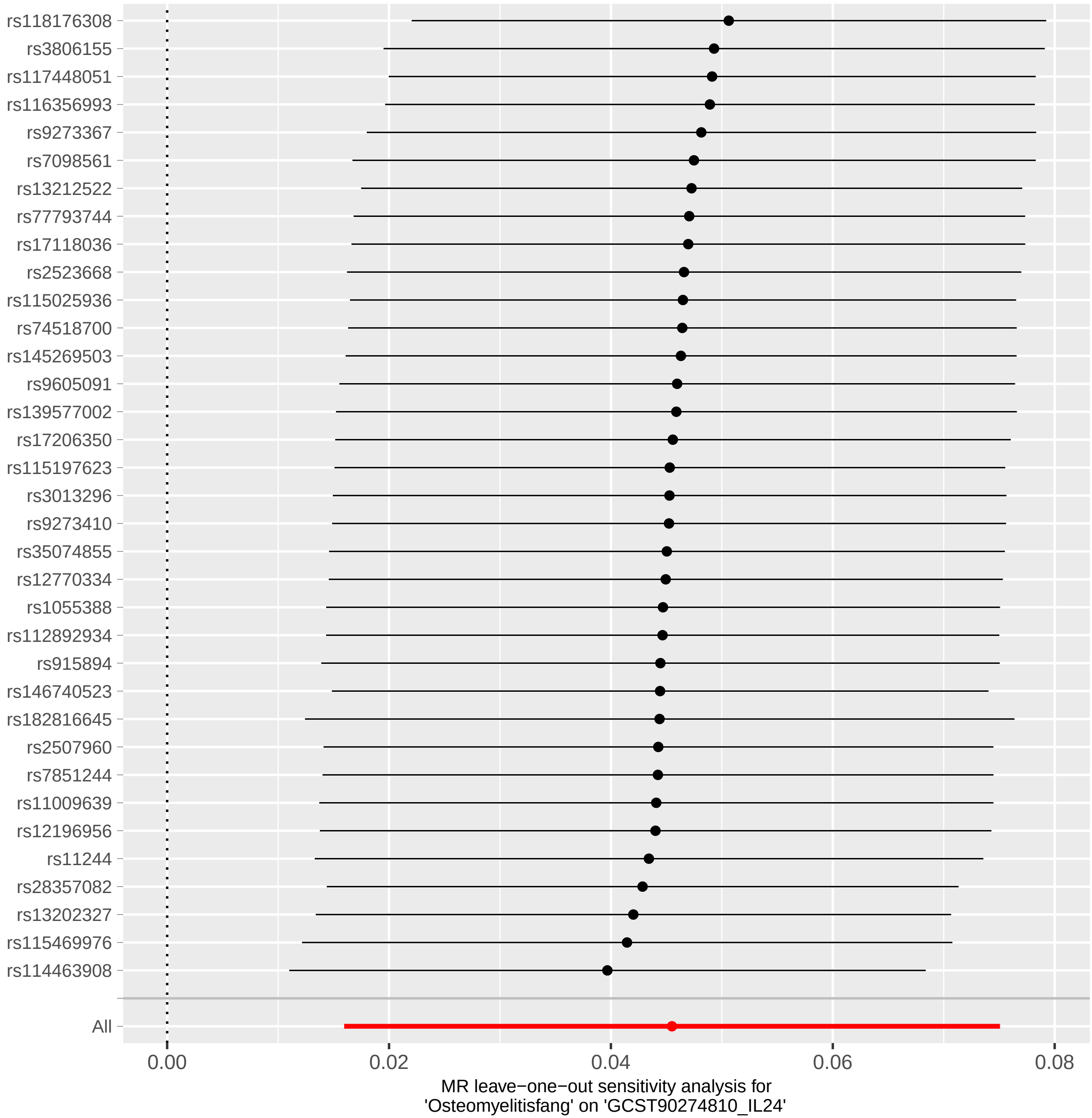

rs118176308
rs3806155
rs117448051
rs116356993
rs9273367
rs7098561
rs13212522
rs77793744
rs17118036
rs2523668
rs115025936
rs74518700
rs145269503
rs9605091
rs139577002
rs17206350
rs115197623
rs3013296
rs9273410
rs35074855
rs12770334
rs1055388
rs112892934
rs915894
rs146740523
rs182816645
rs2507960
rs7851244
rs11009639
rs12196956
rs11244
rs28357082
rs13202327
rs115469976
rs114463908
All
0.00
0.02
0.04
0.06
0.08
MR leave−one−out sensitivity analysis for
'Osteomyelitisfang' on 'GCST90274810_IL24'

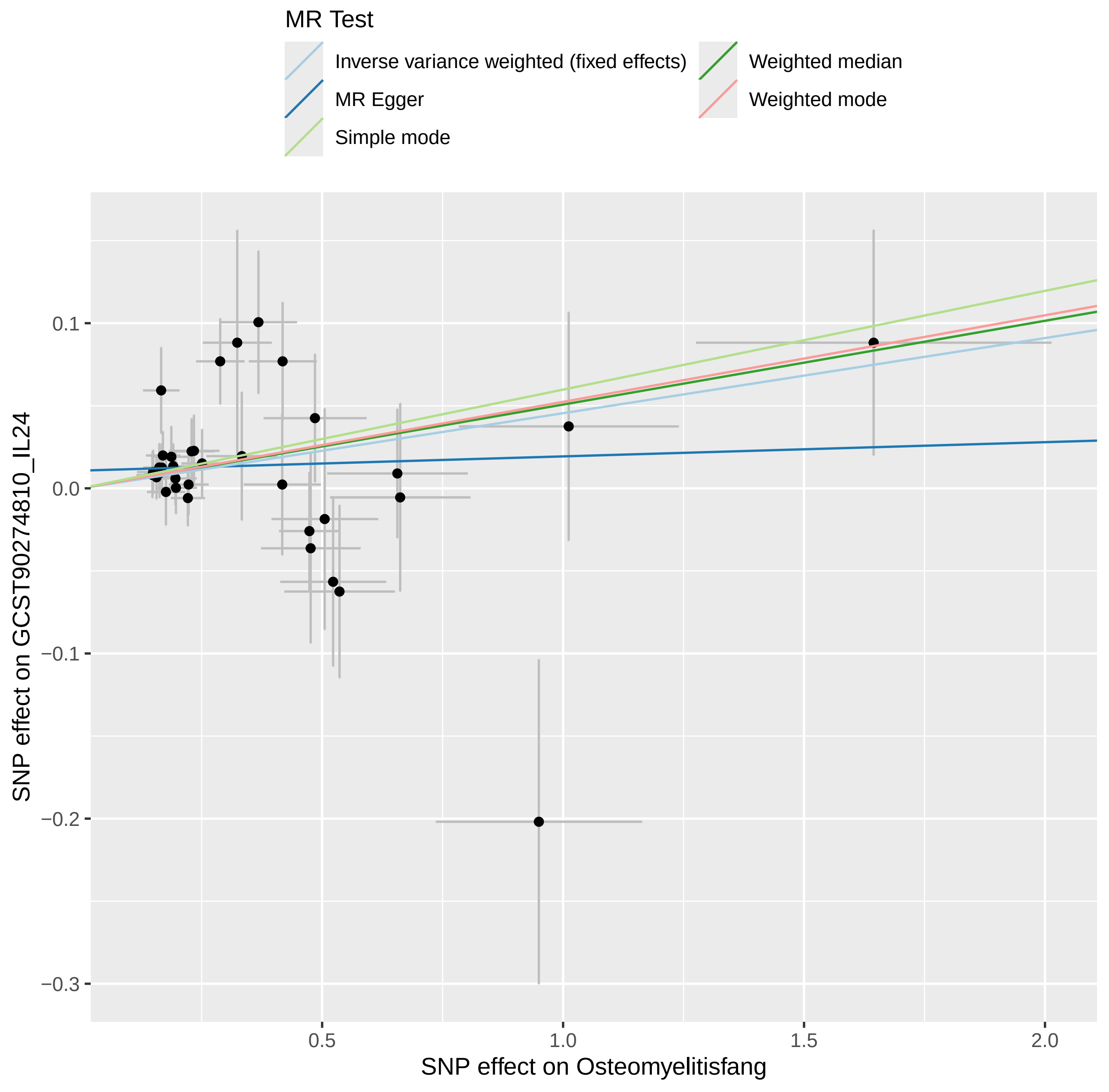
MR Test
Inverse variance weighted (fixed effects)
MR Egger
Simple mode
Weighted median
Weighted mode
SNP effect on GCST90274810_IL24
0.1
0.0
−0.1
−0.2
−0.3
0.5
1.0
1.5
2.0
SNP effect on Osteomyelitisfang

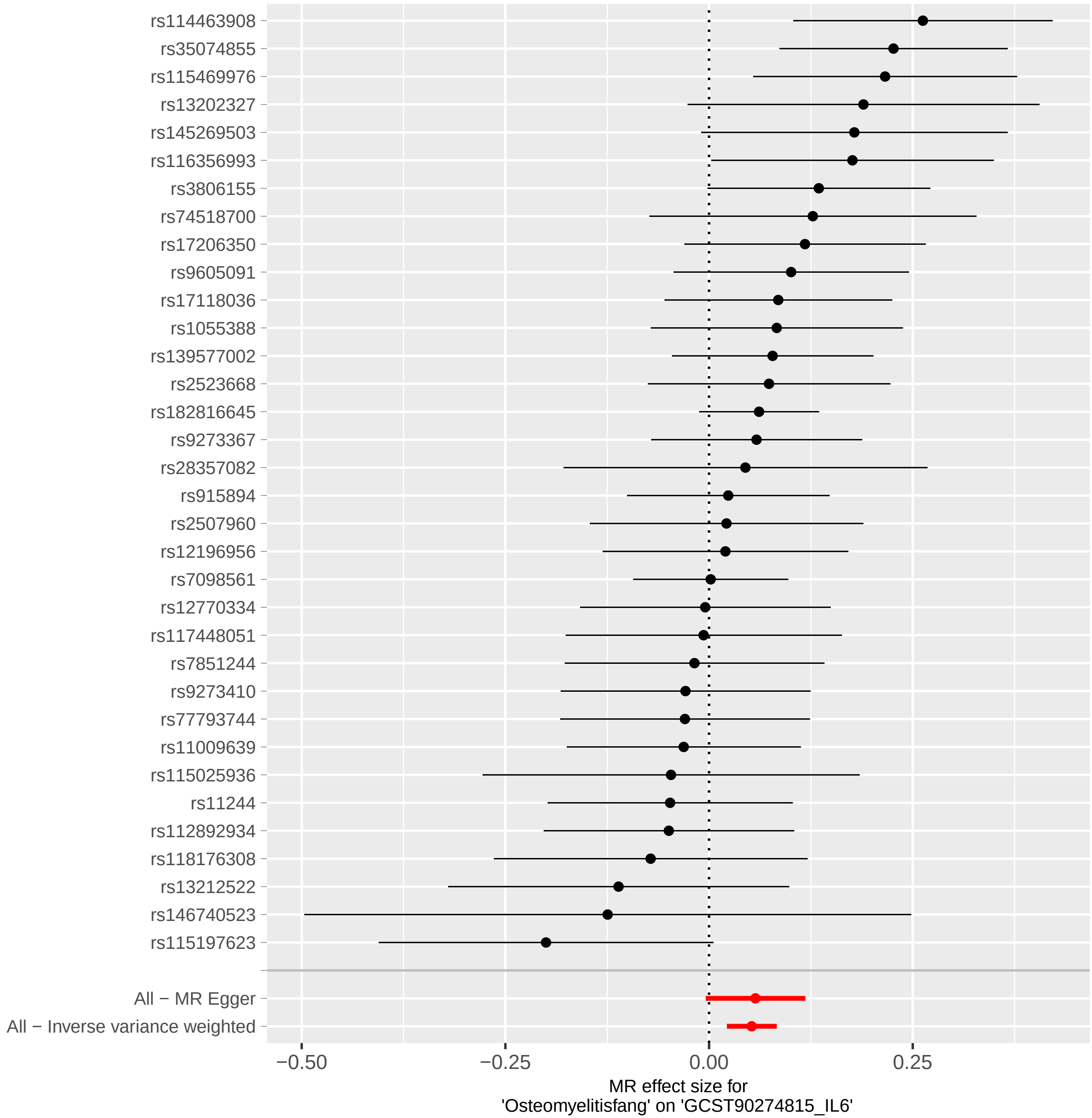

rs114463908
rs35074855
rs115469976
rs13202327
rs145269503
rs116356993
rs3806155
rs74518700
rs17206350
rs9605091
rs17118036
rs1055388
rs139577002
rs2523668
rs182816645
rs9273367
rs28357082
rs915894
rs2507960
rs12196956
rs7098561
rs12770334
rs117448051
rs7851244
rs9273410
rs77793744
rs11009639
rs115025936
rs11244
rs112892934
rs118176308
rs13212522
rs146740523
rs115197623
All − MR Egger
All − Inverse variance weighted
−0.50
−0.25
0.00
0.25
MR effect size for
'Osteomyelitisfang' on 'GCST90274815_IL6'

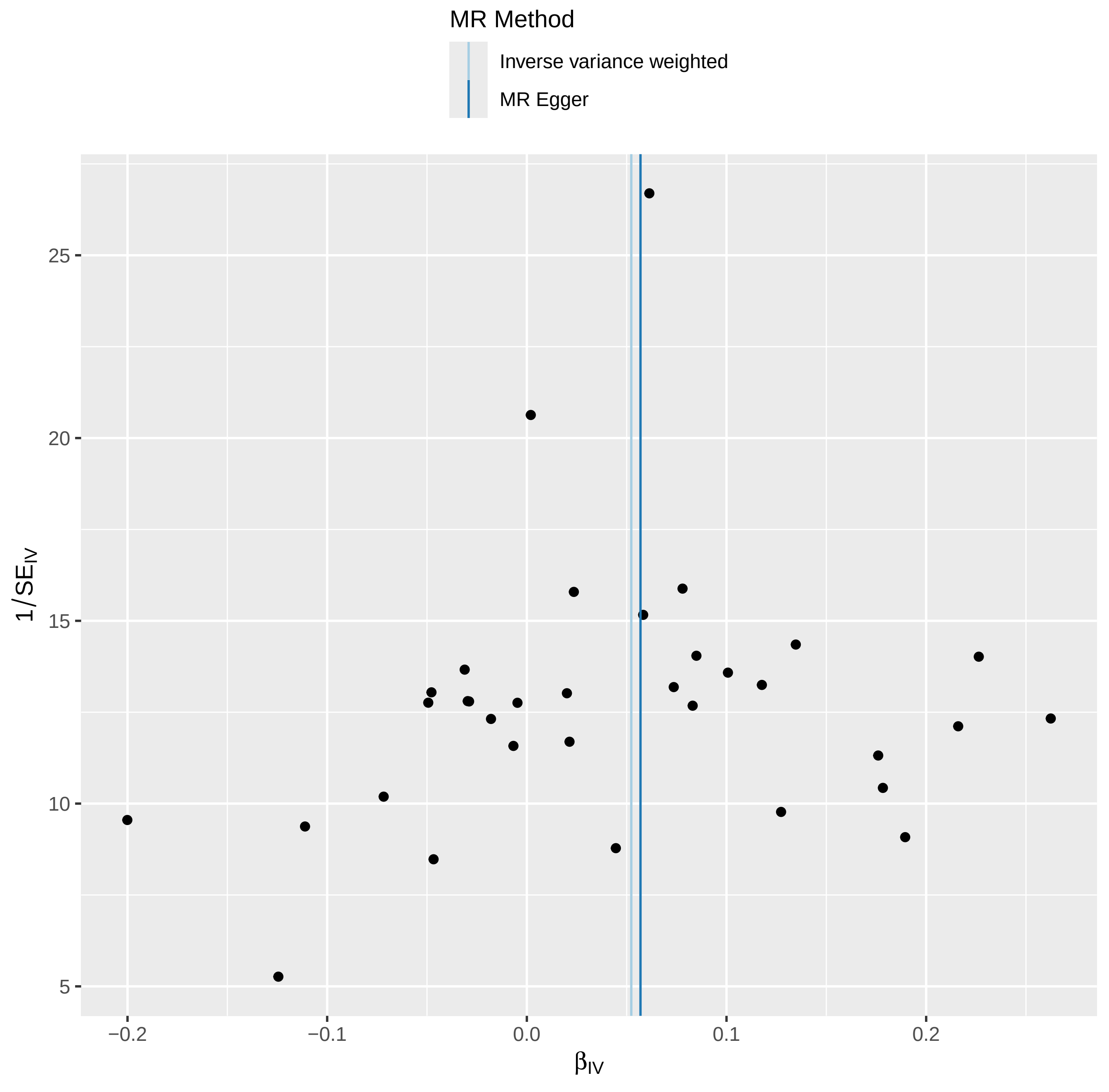
MR Method
Inverse variance weighted
MR Egger
25
20
15
10
5
1/SE_IV
−0.2
−0.1
0.0
0.1
0.2
β_IV

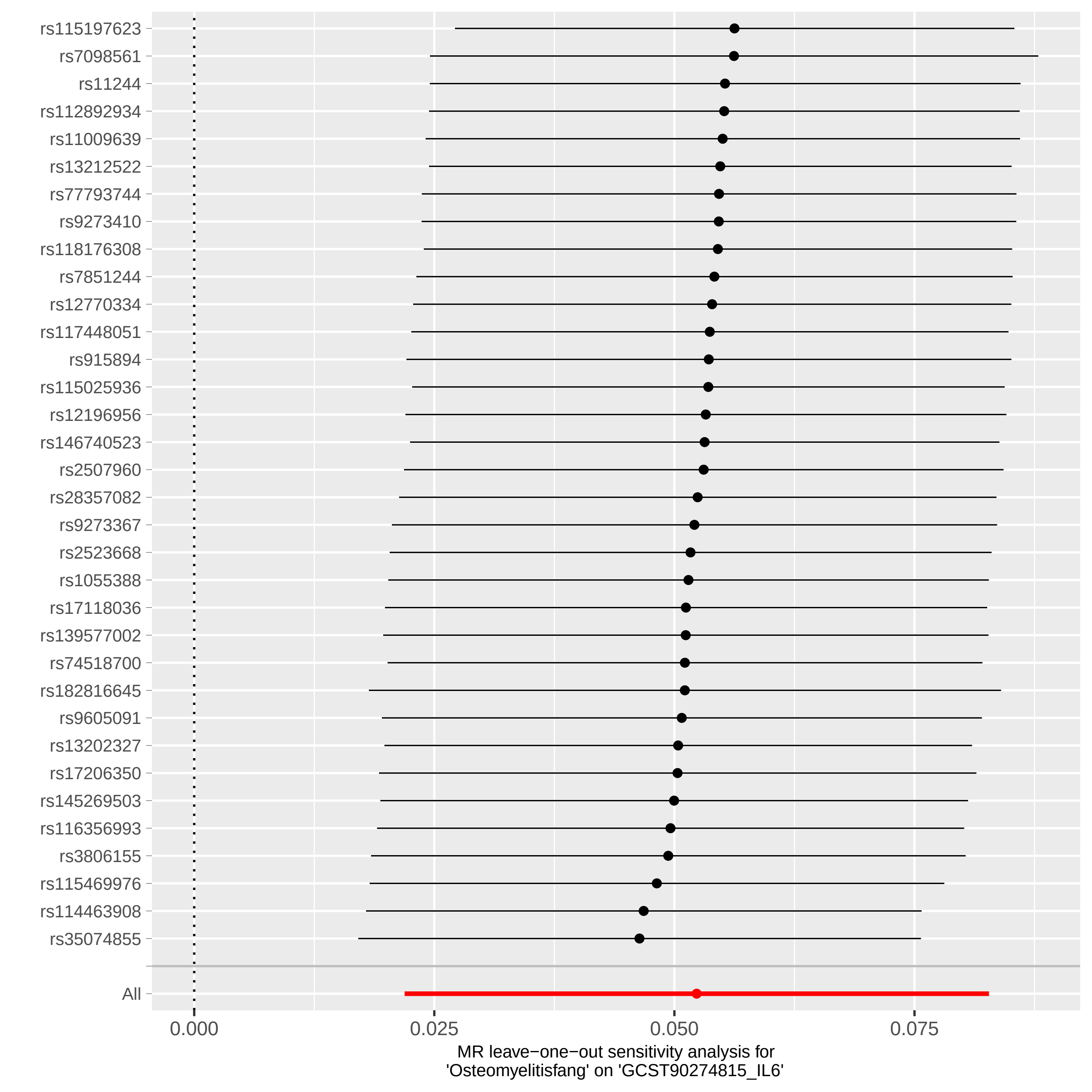
rs115197623
rs7098561
rs11244
rs112892934
rs11009639
rs13212522
rs77793744
rs9273410
rs118176308
rs7851244
rs12770334
rs117448051
rs915894
rs115025936
rs12196956
rs146740523
rs2507960
rs28357082
rs9273367
rs2523668
rs1055388
rs17118036
rs139577002
rs74518700
rs182816645
rs9605091
rs13202327
rs17206350
rs145269503
rs116356993
rs3806155
rs115469976
rs114463908
rs35074855
All
0.000
0.025
0.050
0.075
MR leave−one−out sensitivity analysis for
'Osteomyelitisfang' on 'GCST90274815_IL6'

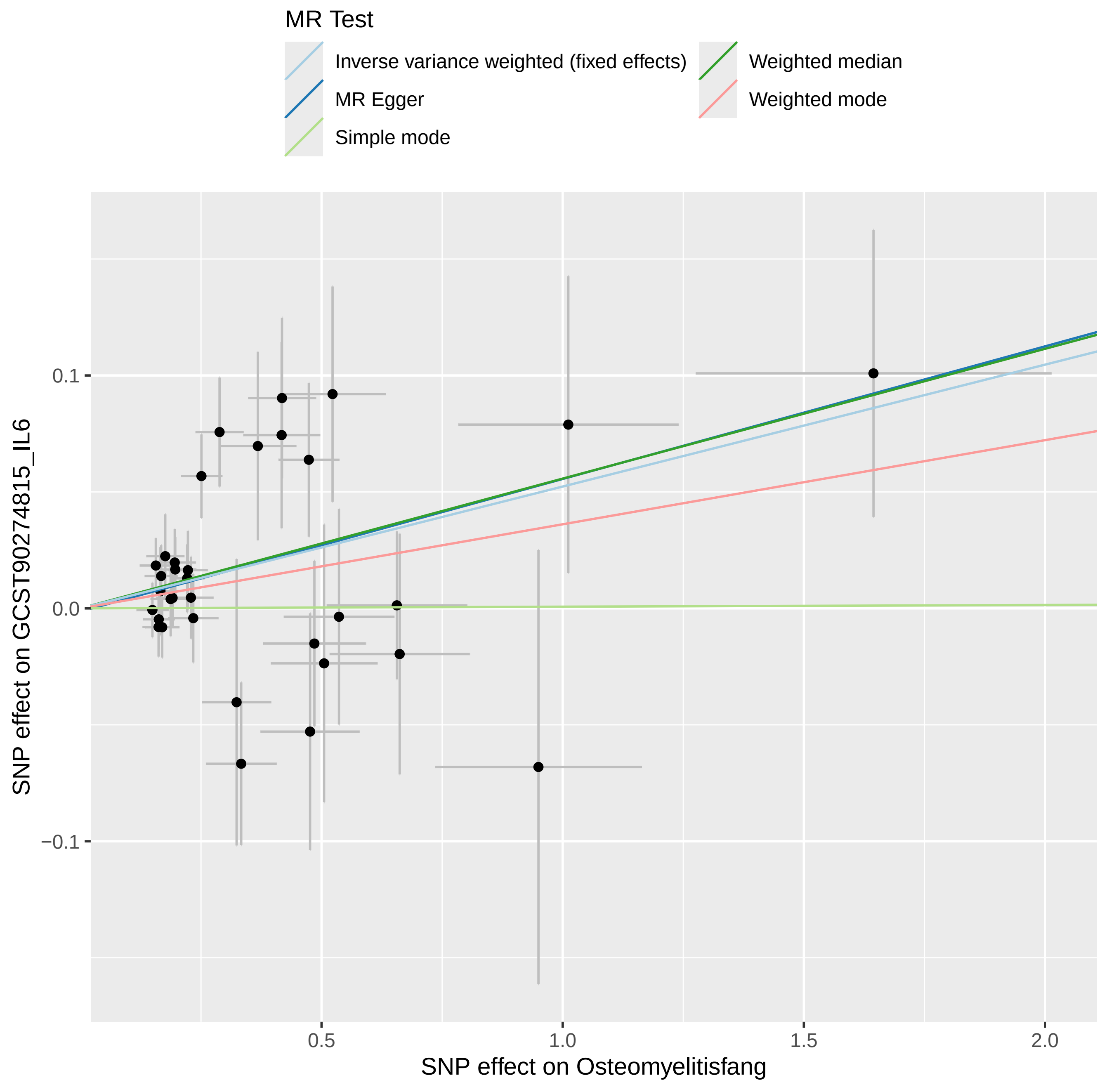

MR Test
Inverse variance weighted (fixed effects)
MR Egger
Simple mode
Weighted median
Weighted mode
SNP effect on GCST90274815_IL6
0.1
0.0
−0.1
0.5
1.0
1.5
2.0
SNP effect on Osteomyelitisfang

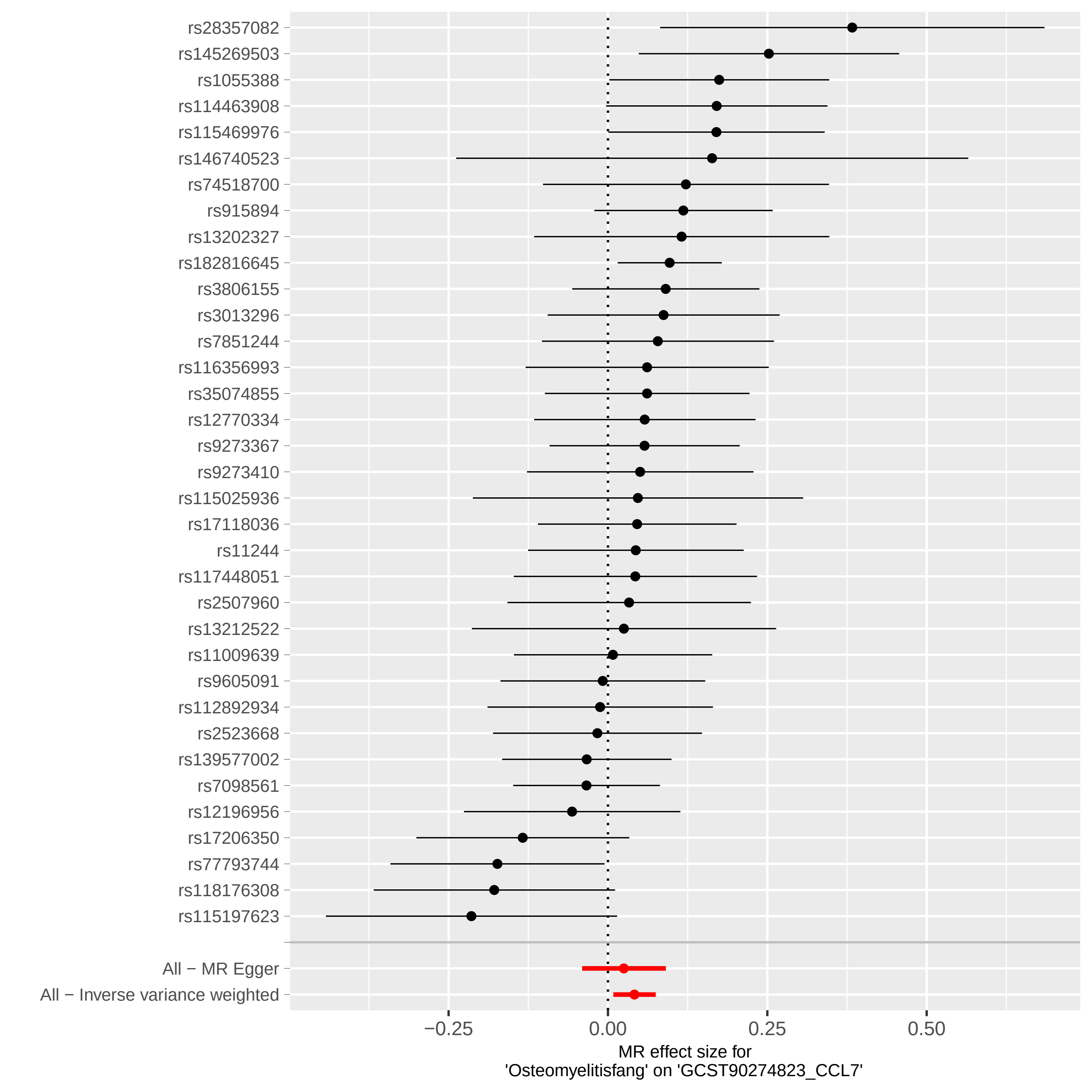
rs28357082
rs145269503
rs1055388
rs114463908
rs115469976
rs146740523
rs74518700
rs915894
rs13202327
rs182816645
rs3806155
rs3013296
rs7851244
rs116356993
rs35074855
rs12770334
rs9273367
rs9273410
rs115025936
rs17118036
rs11244
rs117448051
rs2507960
rs13212522
rs11009639
rs9605091
rs112892934
rs2523668
rs139577002
rs7098561
rs12196956
rs17206350
rs77793744
rs118176308
rs115197623
All − MR Egger
All − Inverse variance weighted
−0.25
0.00
0.25
0.50
MR effect size for
'Osteomyelitisfang' on 'GCST90274823_CCL7'

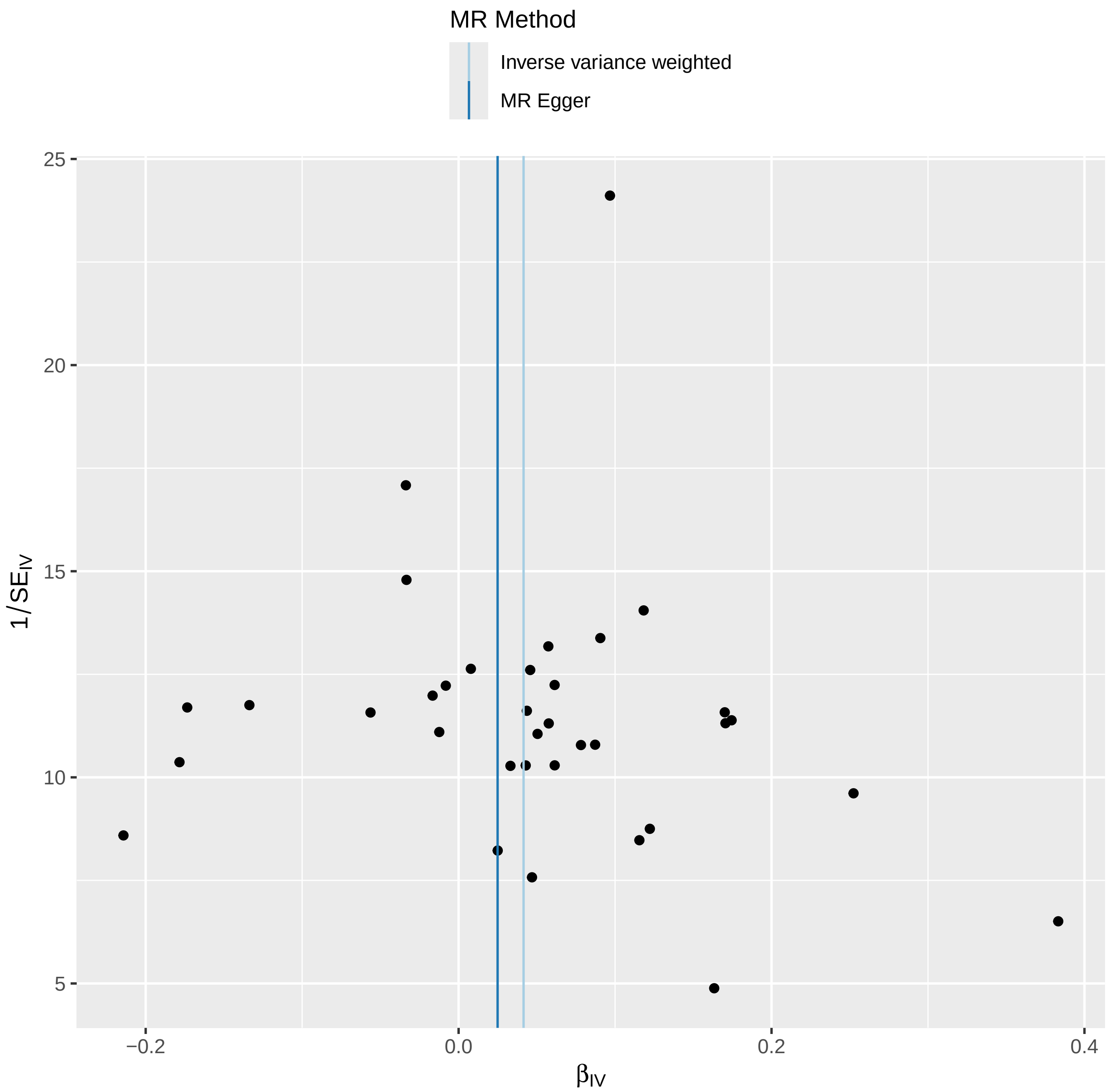
MR Method
Inverse variance weighted
MR Egger
25
20
15
10
5
$1/SE_{IV}$
−0.2
0.0
0.2
0.4
β_{IV}

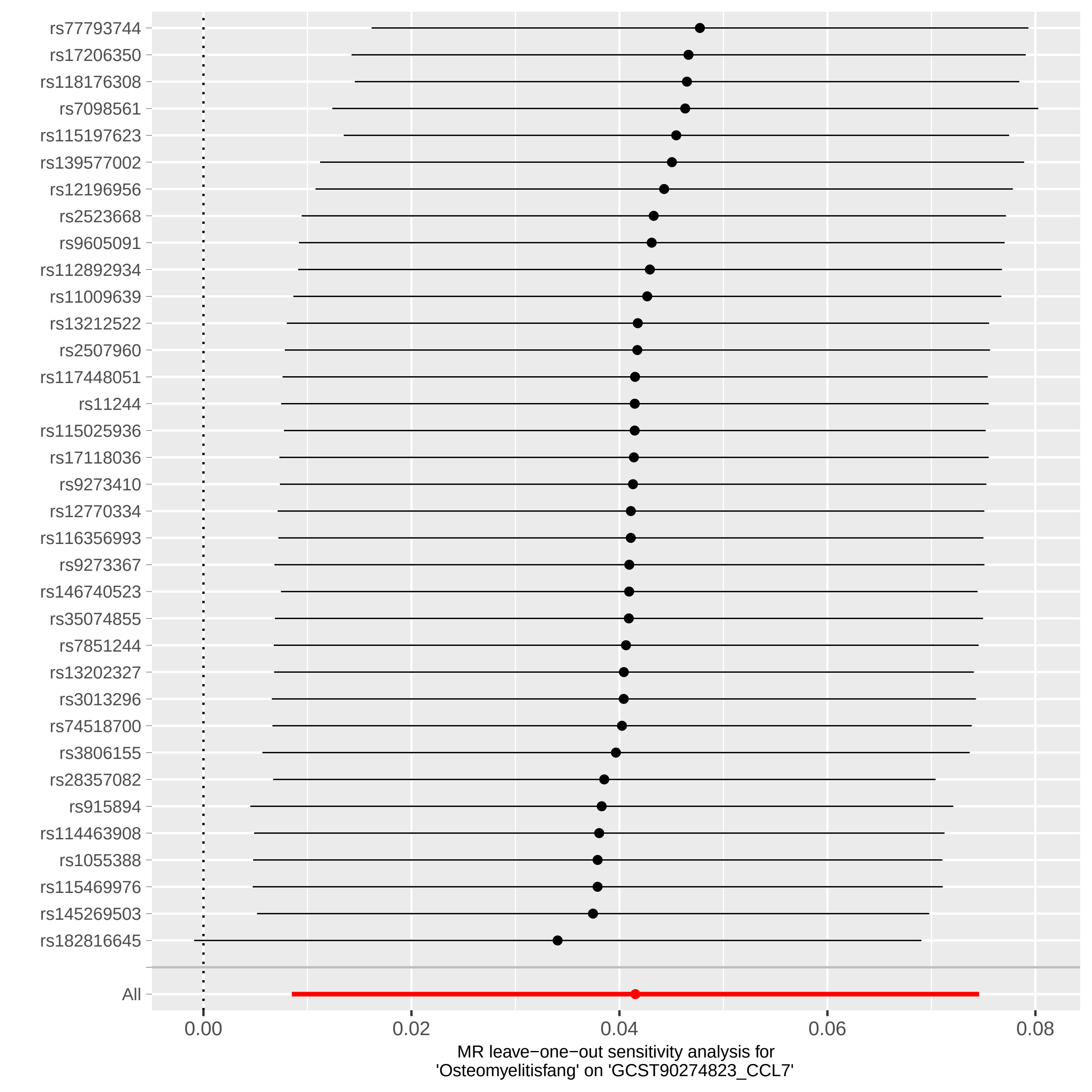

rs77793744
rs17206350
rs118176308
rs7098561
rs115197623
rs139577002
rs12196956
rs2523668
rs9605091
rs112892934
rs11009639
rs13212522
rs2507960
rs117448051
rs11244
rs115025936
rs17118036
rs9273410
rs12770334
rs116356993
rs9273367
rs146740523
rs35074855
rs7851244
rs13202327
rs3013296
rs74518700
rs3806155
rs28357082
rs915894
rs114463908
rs1055388
rs115469976
rs145269503
rs182816645
All
0.00
0.02
0.04
0.06
0.08
MR leave−one−out sensitivity analysis for
'Osteomyelitisfang' on 'GCST90274823_CCL7'

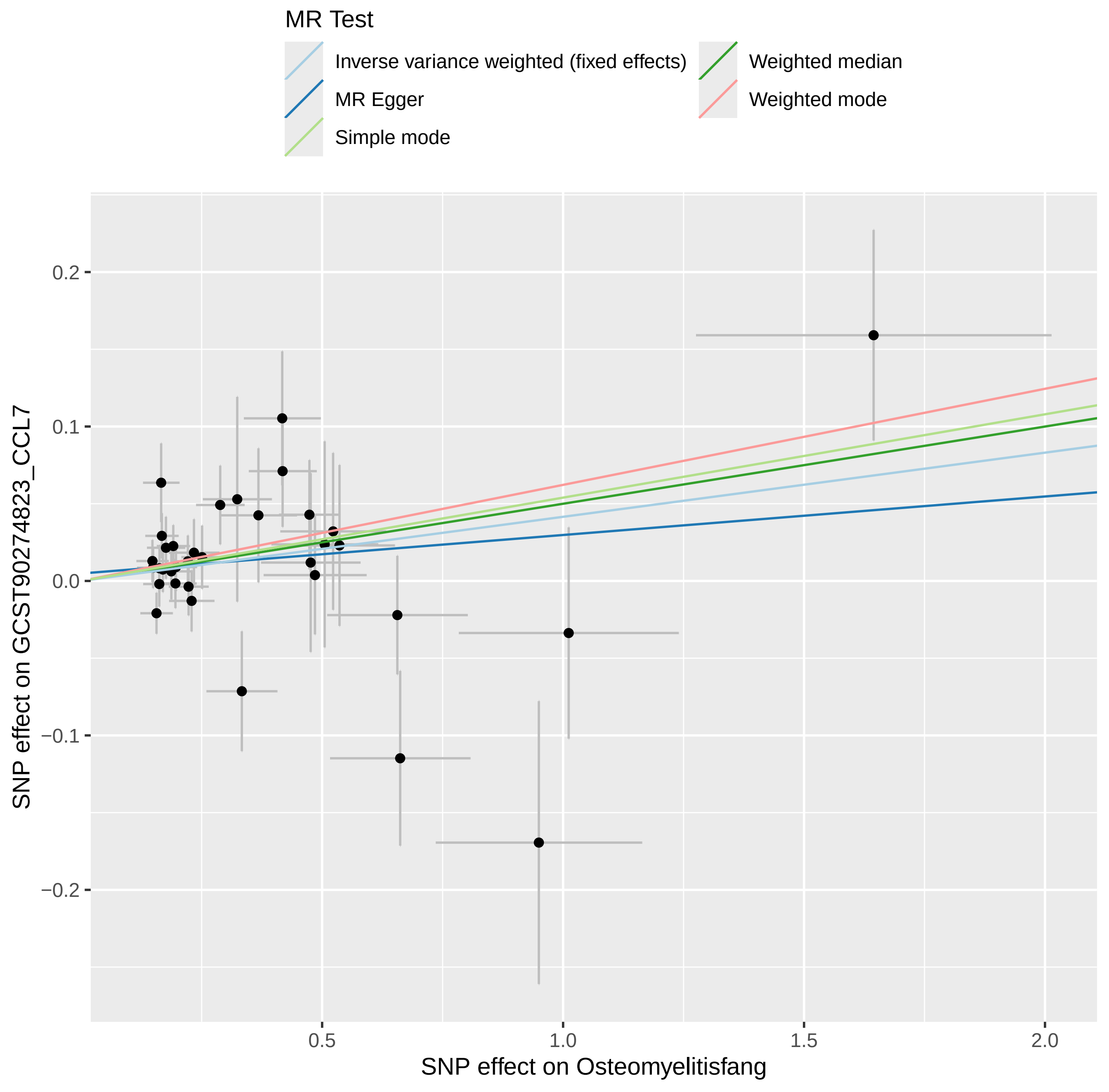

MR Test
Inverse variance weighted (fixed effects)
MR Egger
Simple mode
Weighted median
Weighted mode
SNP effect on GCST90274823_CCL7
SNP effect on Osteomyelitisfang
0.2
0.1
0.0
−0.1
−0.2
0.5
1.0
1.5
2.0

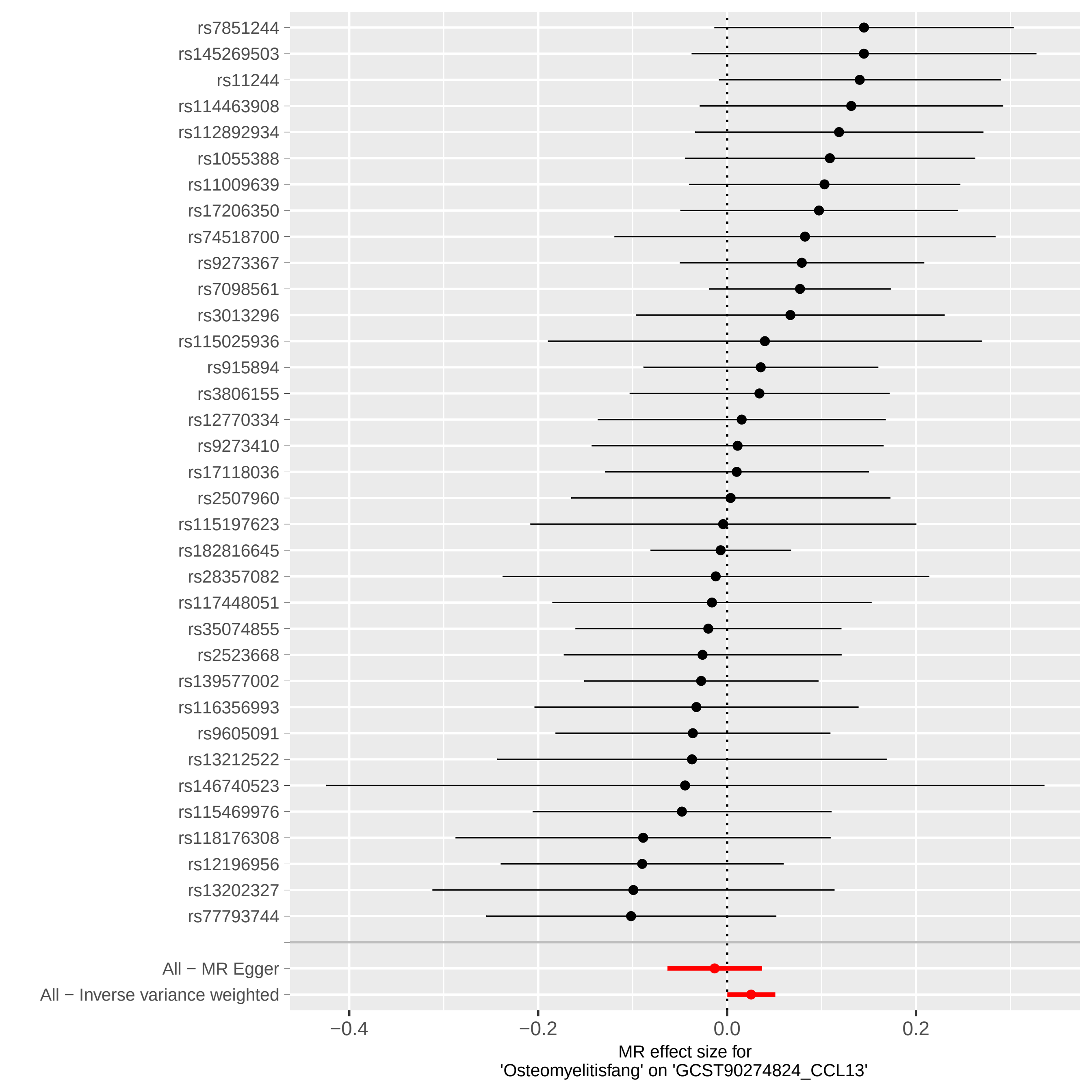
rs7851244
rs145269503
rs11244
rs114463908
rs112892934
rs1055388
rs11009639
rs17206350
rs74518700
rs9273367
rs7098561
rs3013296
rs115025936
rs915894
rs3806155
rs12770334
rs9273410
rs17118036
rs2507960
rs115197623
rs182816645
rs28357082
rs117448051
rs35074855
rs2523668
rs139577002
rs116356993
rs9605091
rs13212522
rs146740523
rs115469976
rs118176308
rs12196956
rs13202327
rs77793744
All − MR Egger
All − Inverse variance weighted
−0.4
−0.2
0.0
0.2
MR effect size for
'Osteomyelitisfang' on 'GCST90274824_CCL13'

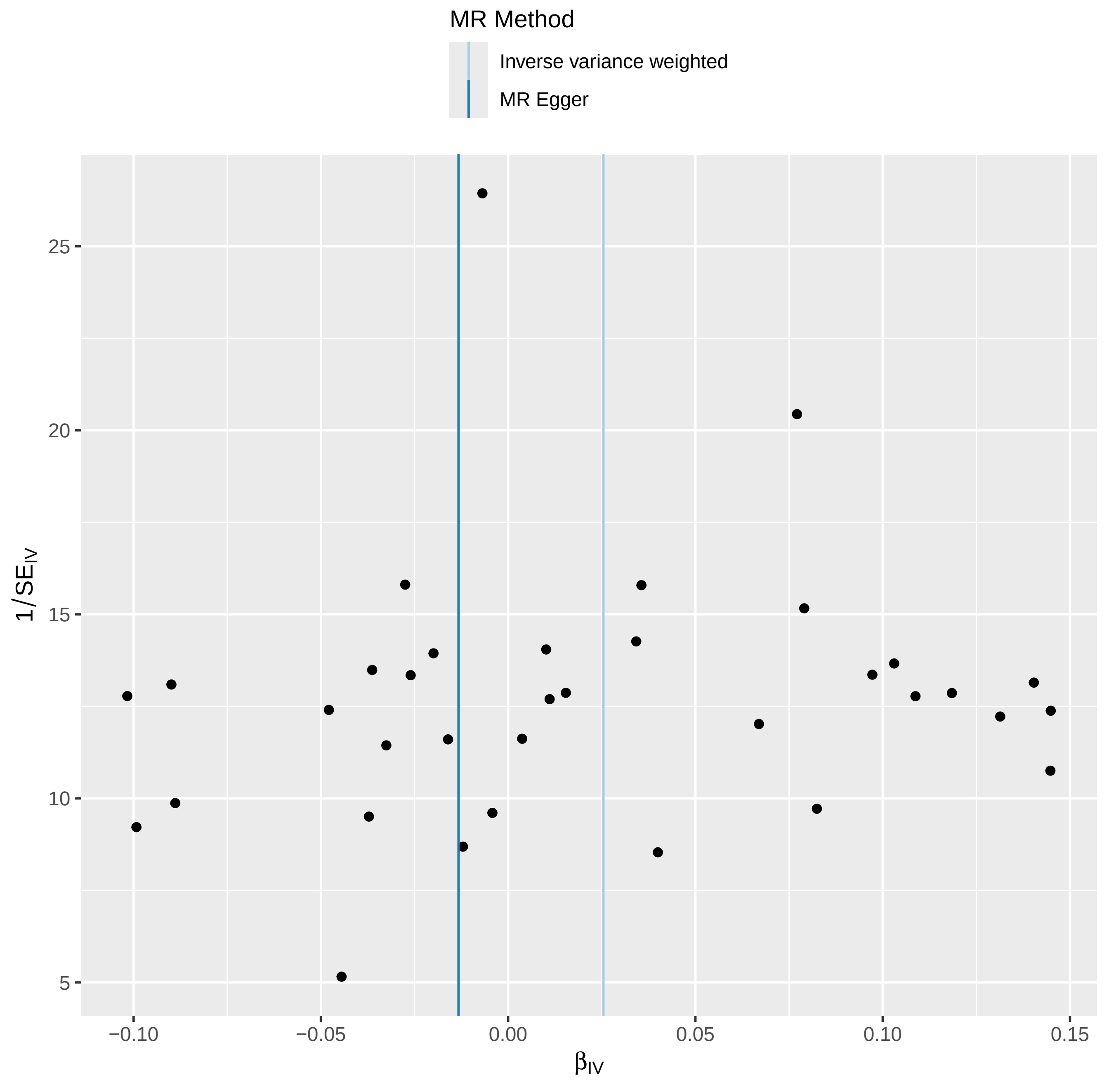
MR Method
Inverse variance weighted
MR Egger
25
20
15
10
5
1/SE_IV
−0.10
−0.05
0.00
0.05
0.10
0.15
β_IV

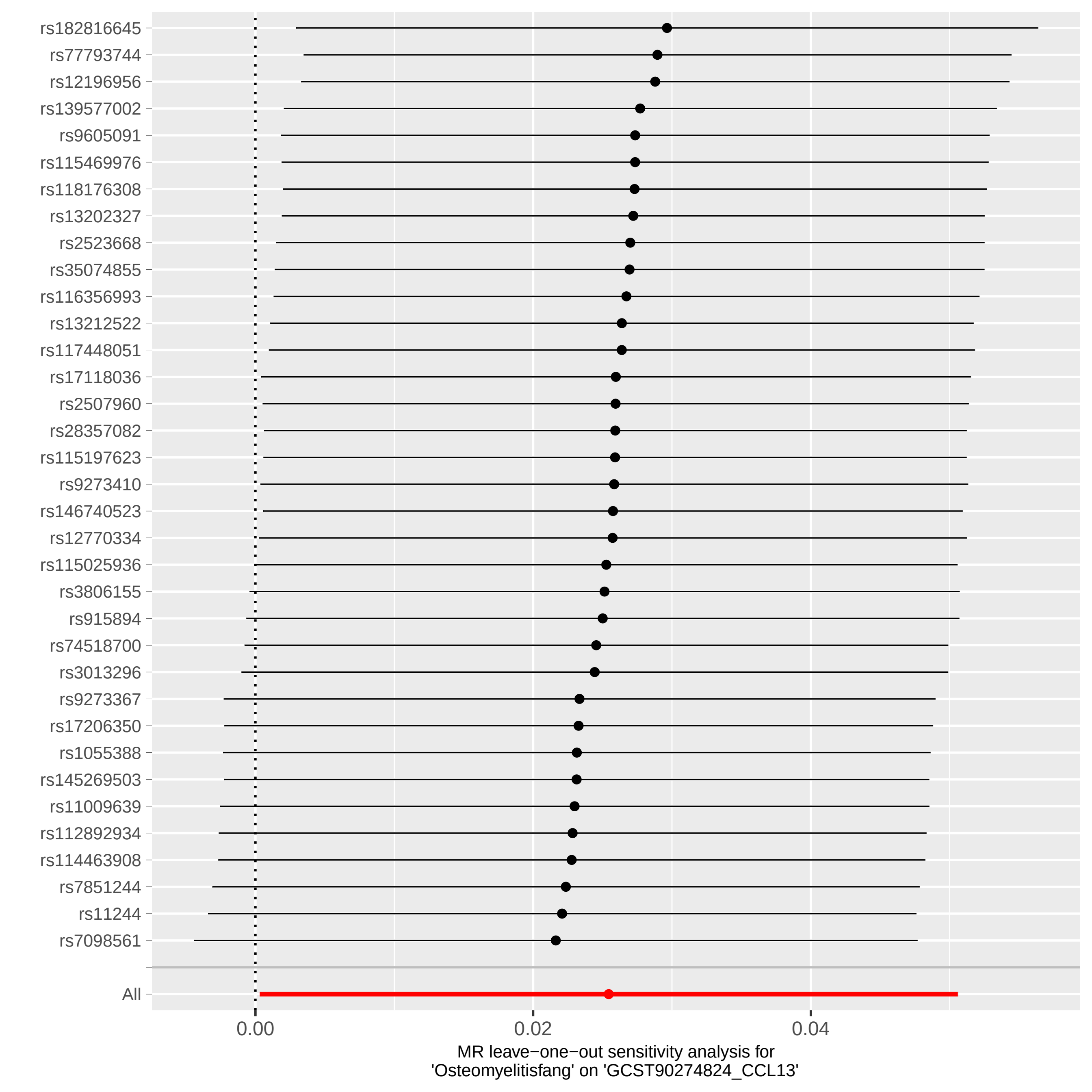
rs182816645
rs77793744
rs12196956
rs139577002
rs9605091
rs115469976
rs118176308
rs13202327
rs2523668
rs35074855
rs116356993
rs13212522
rs117448051
rs17118036
rs2507960
rs28357082
rs115197623
rs9273410
rs146740523
rs12770334
rs115025936
rs3806155
rs915894
rs74518700
rs3013296
rs9273367
rs17206350
rs1055388
rs145269503
rs11009639
rs112892934
rs114463908
rs7851244
rs11244
rs7098561
All
0.00
0.02
0.04
MR leave−one−out sensitivity analysis for
'Osteomyelitisfang' on 'GCST90274824_CCL13'

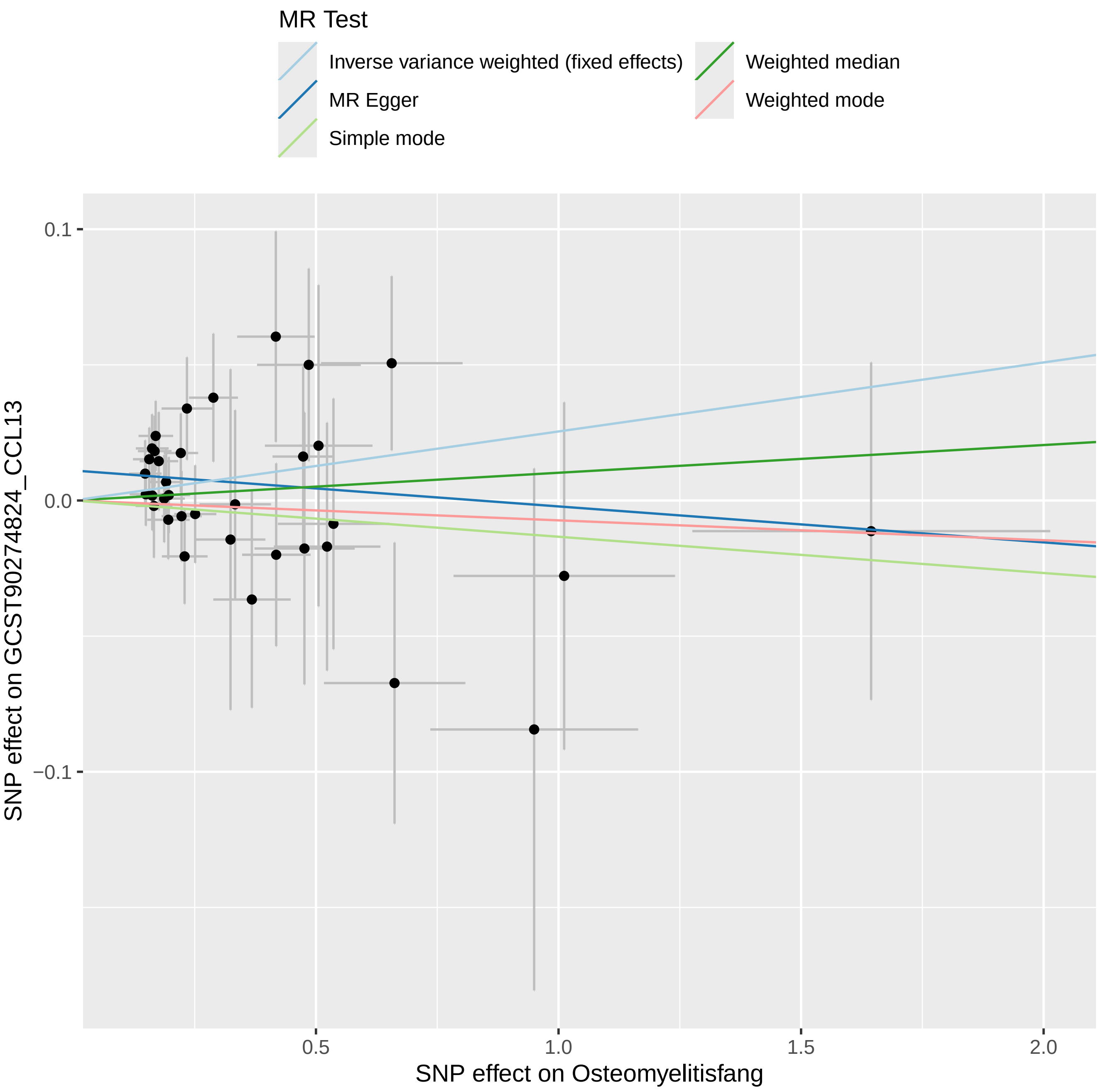

MR Test
Inverse variance weighted (fixed effects)
MR Egger
Simple mode
Weighted median
Weighted mode
0.1
0.0
−0.1
0.5
1.0
1.5
2.0
SNP effect on GCST90274824_CCL13
SNP effect on Osteomyelitisfang

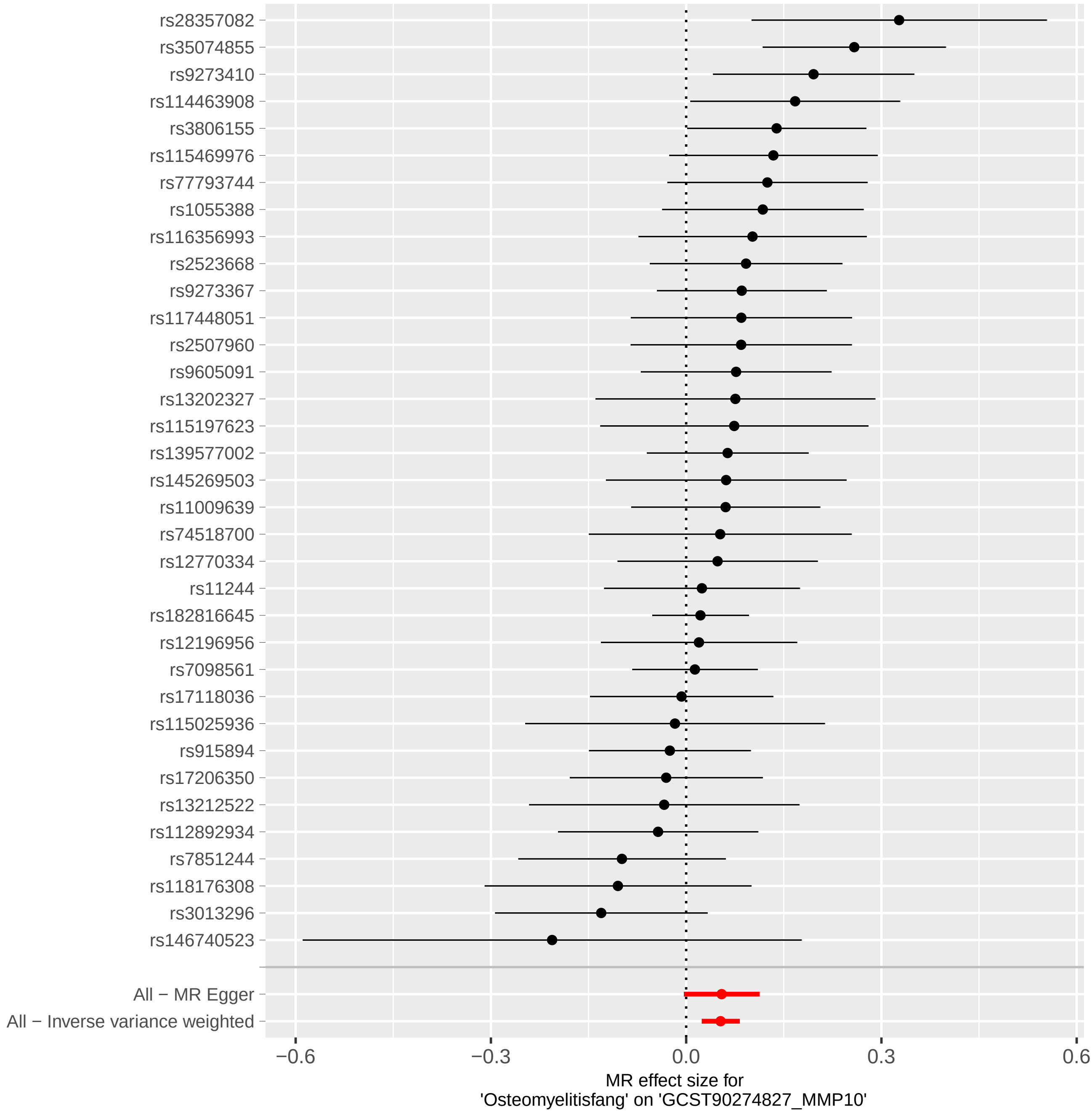
rs28357082
rs35074855
rs9273410
rs114463908
rs3806155
rs115469976
rs77793744
rs1055388
rs116356993
rs2523668
rs9273367
rs117448051
rs2507960
rs9605091
rs13202327
rs115197623
rs139577002
rs145269503
rs11009639
rs74518700
rs12770334
rs11244
rs182816645
rs12196956
rs7098561
rs17118036
rs115025936
rs915894
rs17206350
rs13212522
rs112892934
rs7851244
rs118176308
rs3013296
rs146740523
All − MR Egger
All − Inverse variance weighted
−0.6
−0.3
0.0
0.3
0.6
MR effect size for
'Osteomyelitisfang' on 'GCST90274827_MMP10'

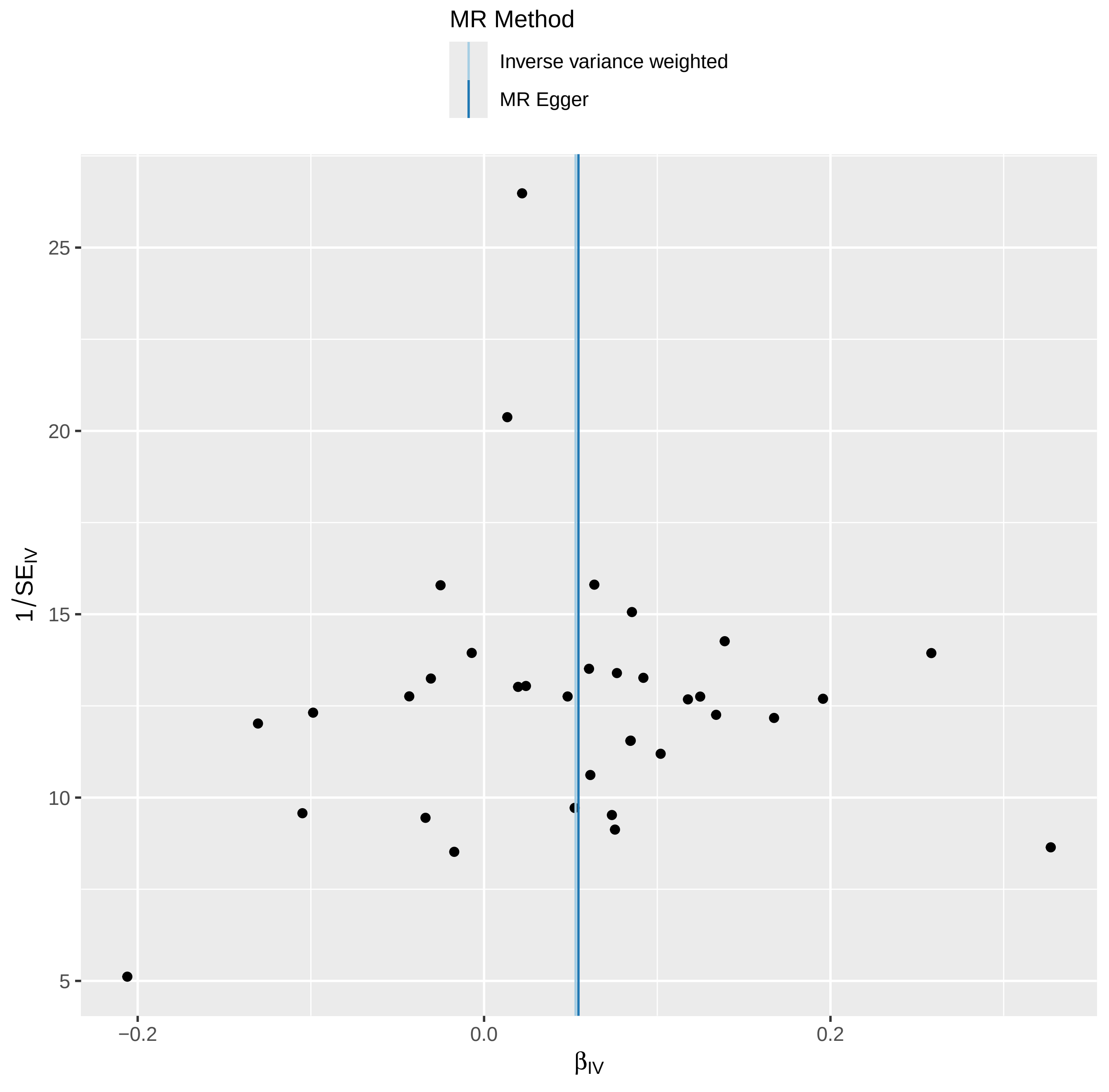
MR Method
Inverse variance weighted
MR Egger
25
20
15
10
5
1/SE_IV
−0.2
0.0
0.2
β_IV

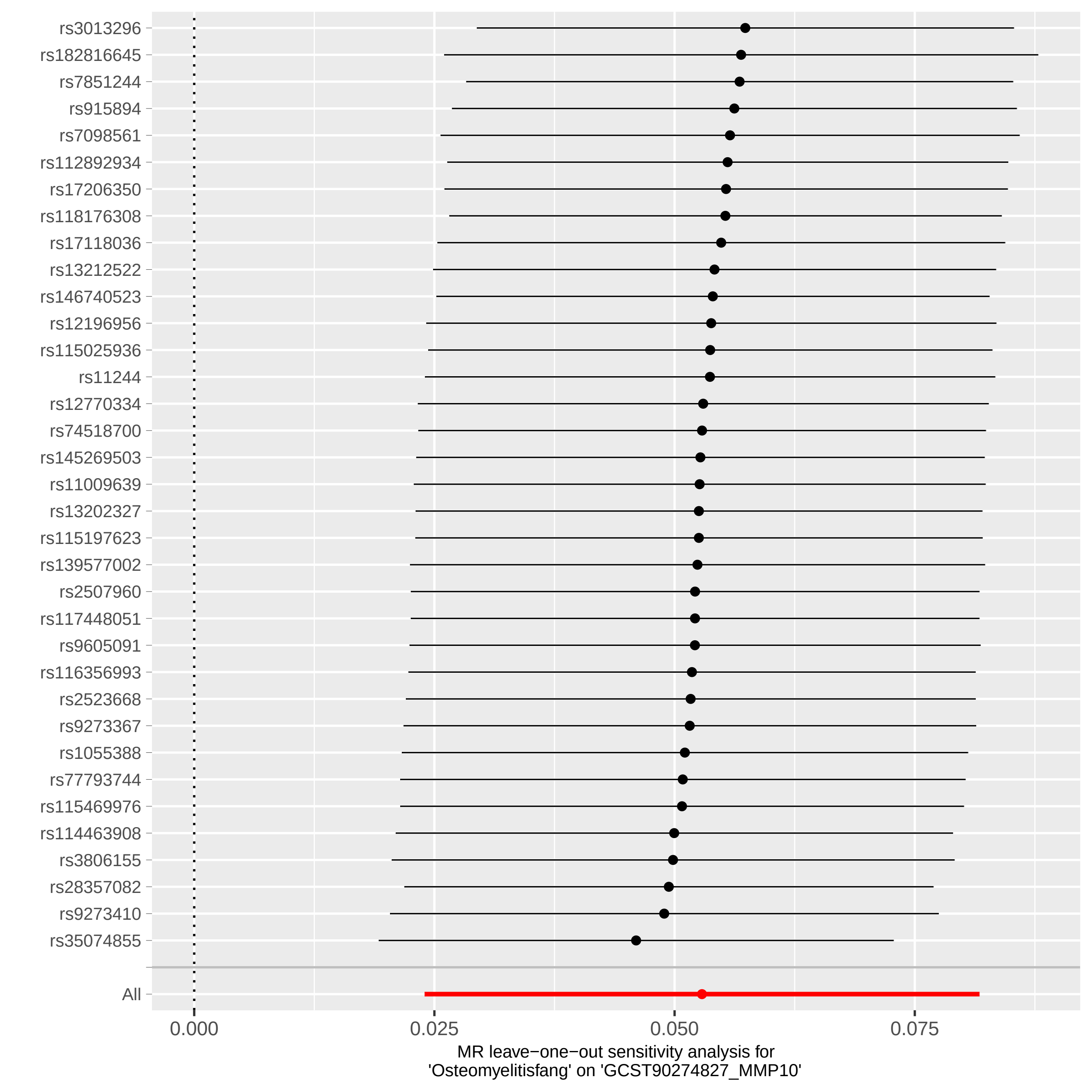
rs3013296
rs182816645
rs7851244
rs915894
rs7098561
rs112892934
rs17206350
rs118176308
rs17118036
rs13212522
rs146740523
rs12196956
rs115025936
rs11244
rs12770334
rs74518700
rs145269503
rs11009639
rs13202327
rs115197623
rs139577002
rs2507960
rs117448051
rs9605091
rs116356993
rs2523668
rs9273367
rs1055388
rs77793744
rs115469976
rs114463908
rs3806155
rs28357082
rs9273410
rs35074855
All
0.000
0.025
0.050
0.075
MR leave−one−out sensitivity analysis for
'Osteomyelitisfang' on 'GCST90274827_MMP10'

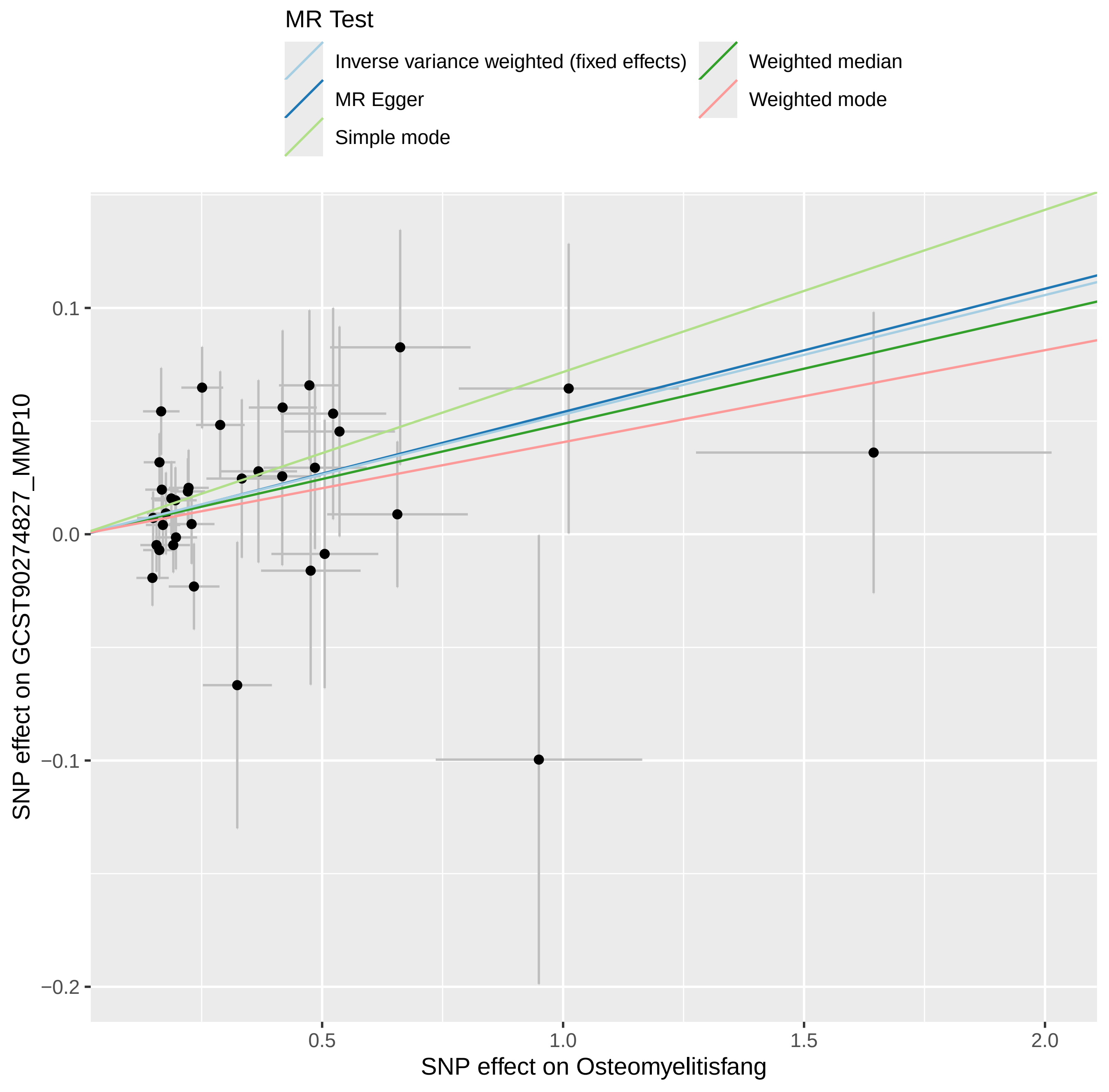

MR Test
Inverse variance weighted (fixed effects)
MR Egger
Simple mode
Weighted median
Weighted mode
SNP effect on GCST90274827_MMP10
0.1
0.0
−0.1
−0.2
0.5
1.0
1.5
2.0
SNP effect on Osteomyelitisfang

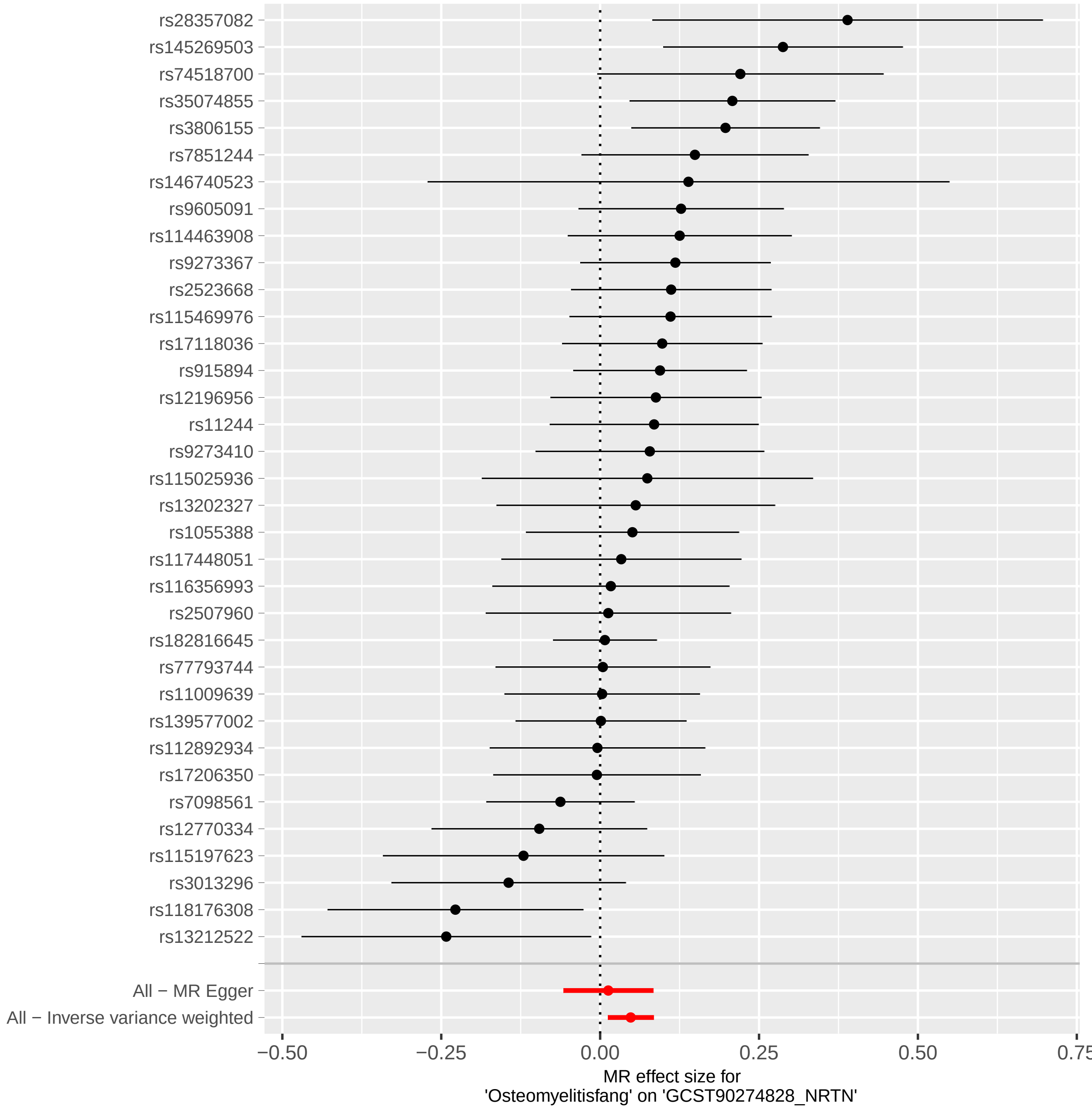

rs28357082
rs145269503
rs74518700
rs35074855
rs3806155
rs7851244
rs146740523
rs9605091
rs114463908
rs9273367
rs2523668
rs115469976
rs17118036
rs915894
rs12196956
rs11244
rs9273410
rs115025936
rs13202327
rs1055388
rs117448051
rs116356993
rs2507960
rs182816645
rs77793744
rs11009639
rs139577002
rs112892934
rs17206350
rs7098561
rs12770334
rs115197623
rs3013296
rs118176308
rs13212522
All − MR Egger
All − Inverse variance weighted
−0.50
−0.25
0.00
0.25
0.50
0.75
MR effect size for
'Osteomyelitisfang' on 'GCST90274828_NRTN'

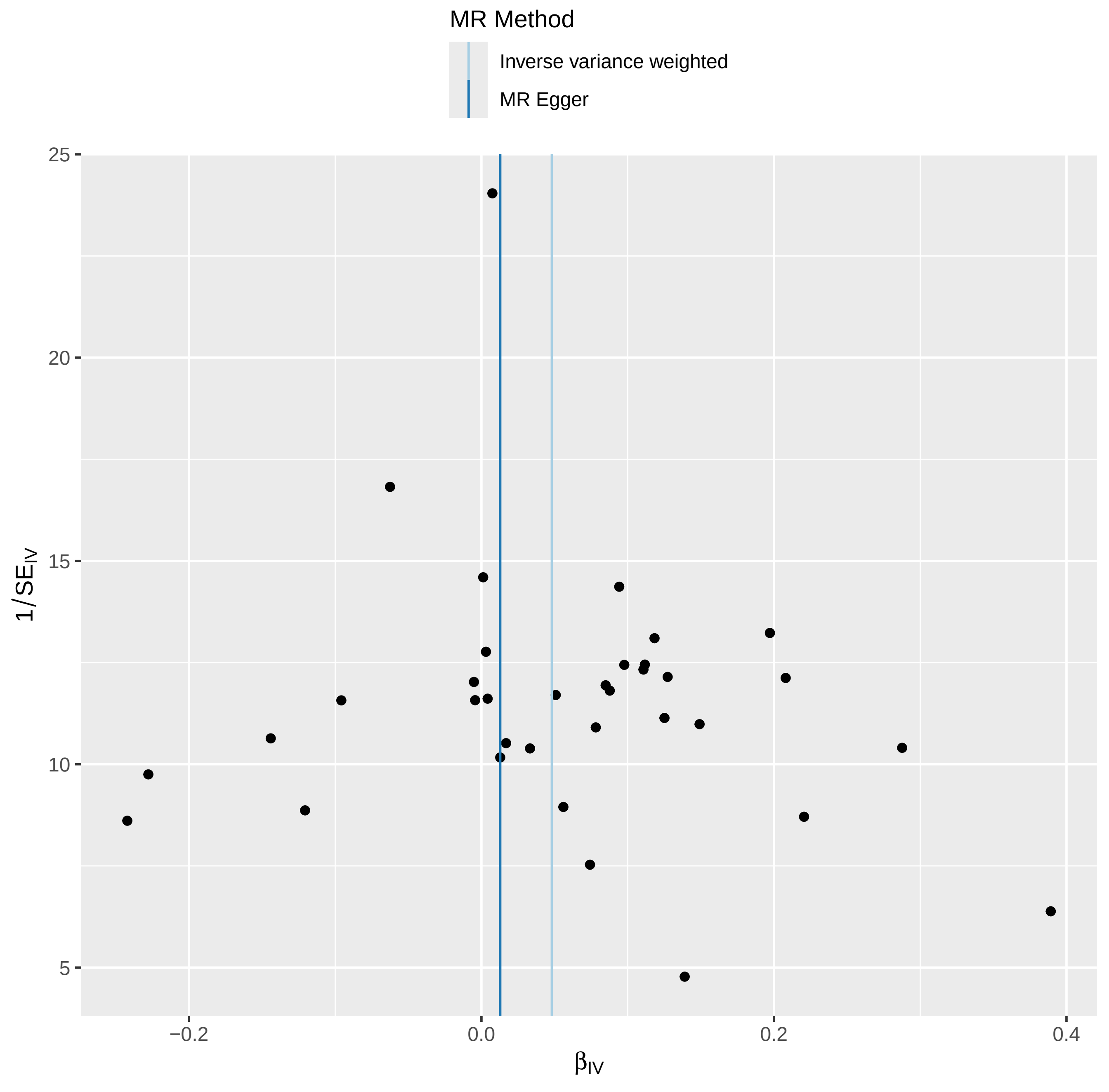
MR Method
Inverse variance weighted
MR Egger
25
20
15
10
5
1/SE$_{IV}$
−0.2
0.0
0.2
0.4
β$_{IV}$

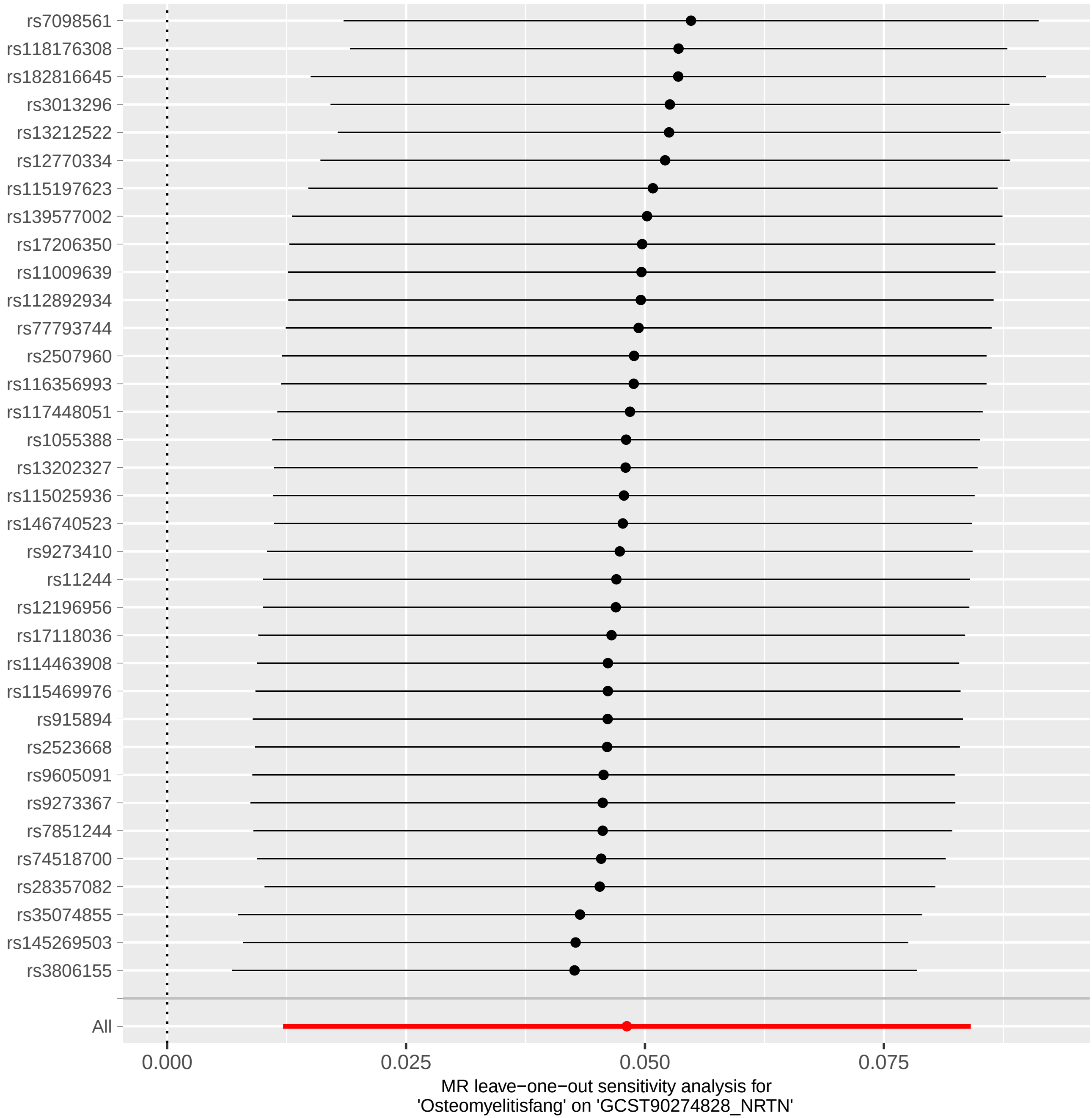

rs7098561
rs118176308
rs182816645
rs3013296
rs13212522
rs12770334
rs115197623
rs139577002
rs17206350
rs11009639
rs112892934
rs77793744
rs2507960
rs116356993
rs117448051
rs1055388
rs13202327
rs115025936
rs146740523
rs9273410
rs11244
rs12196956
rs17118036
rs114463908
rs115469976
rs915894
rs2523668
rs9605091
rs9273367
rs7851244
rs74518700
rs28357082
rs35074855
rs145269503
rs3806155
All
0.000
0.025
0.050
0.075
MR leave−one−out sensitivity analysis for
'Osteomyelitisfang' on 'GCST90274828_NRTN'

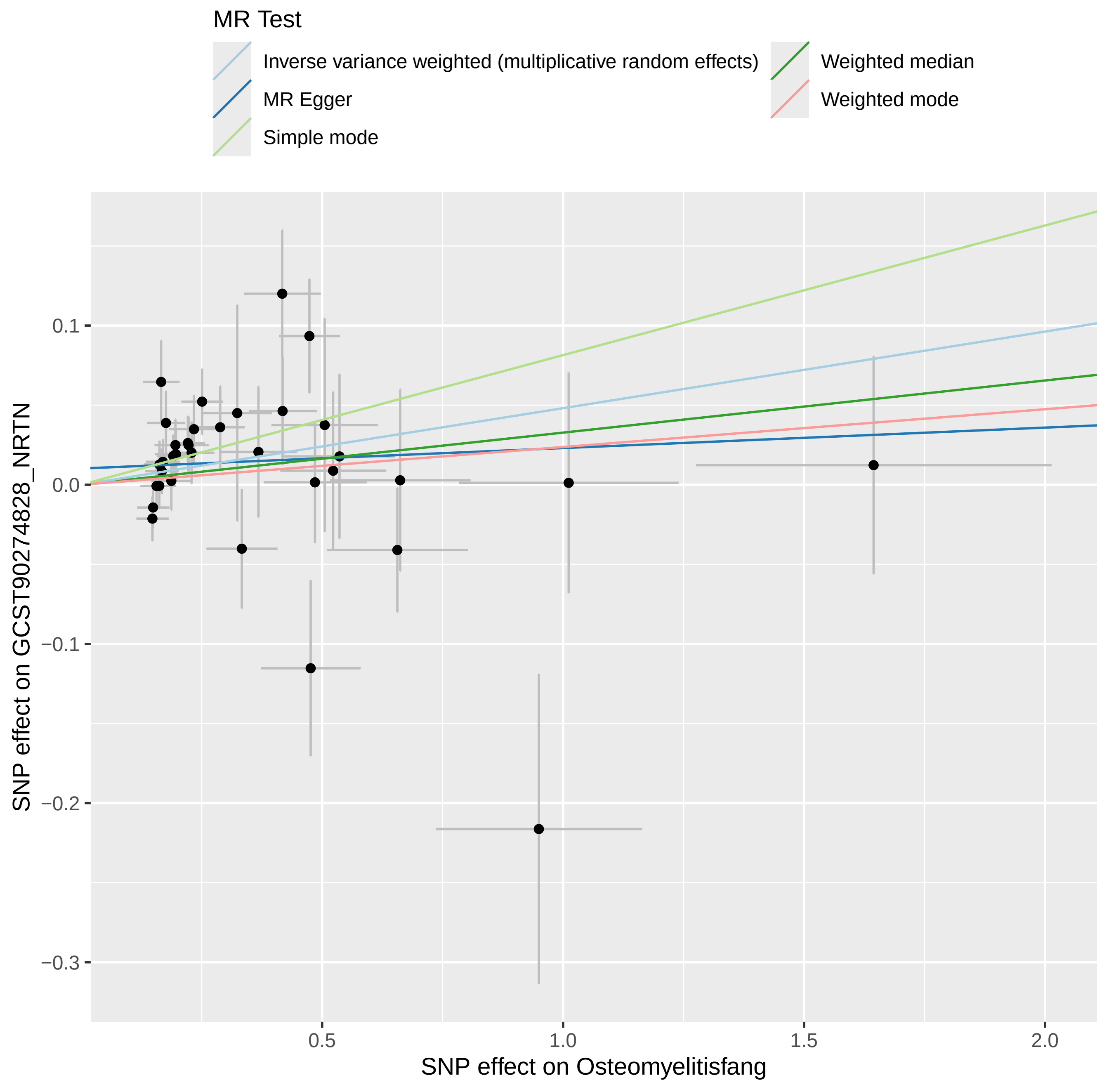

MR Test
Inverse variance weighted (multiplicative random effects)
MR Egger
Simple mode
Weighted median
Weighted mode
SNP effect on GCST90274828_NRTN
0.1
0.0
−0.1
−0.2
−0.3
0.5
1.0
1.5
2.0
SNP effect on Osteomyelitisfang

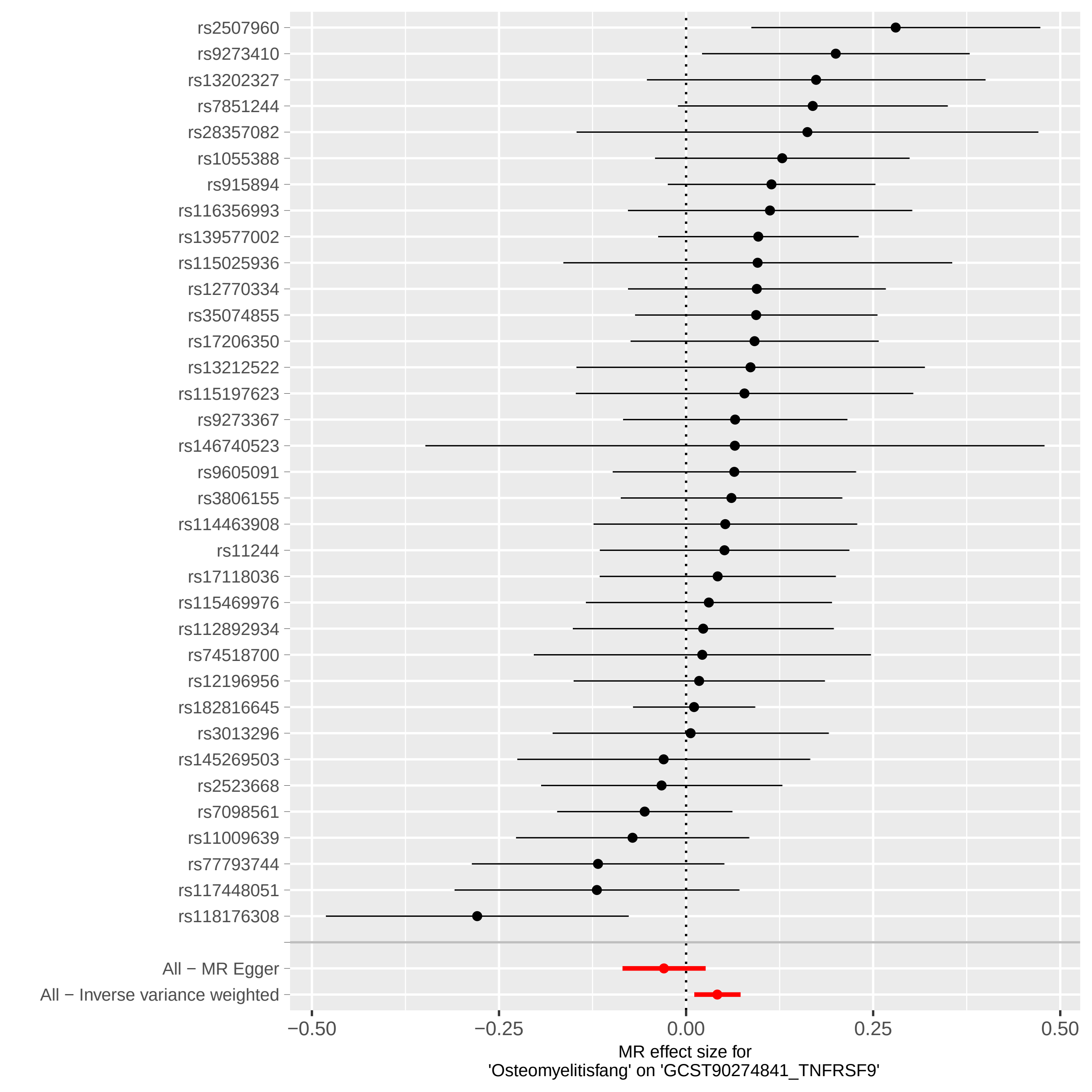
rs2507960
rs9273410
rs13202327
rs7851244
rs28357082
rs1055388
rs915894
rs116356993
rs139577002
rs115025936
rs12770334
rs35074855
rs17206350
rs13212522
rs115197623
rs9273367
rs146740523
rs9605091
rs3806155
rs114463908
rs11244
rs17118036
rs115469976
rs112892934
rs74518700
rs12196956
rs182816645
rs3013296
rs145269503
rs2523668
rs7098561
rs11009639
rs77793744
rs117448051
rs118176308
All − MR Egger
All − Inverse variance weighted
−0.50
−0.25
0.00
0.25
0.50
MR effect size for
'Osteomyelitisfang' on 'GCST90274841_TNFRSF9'

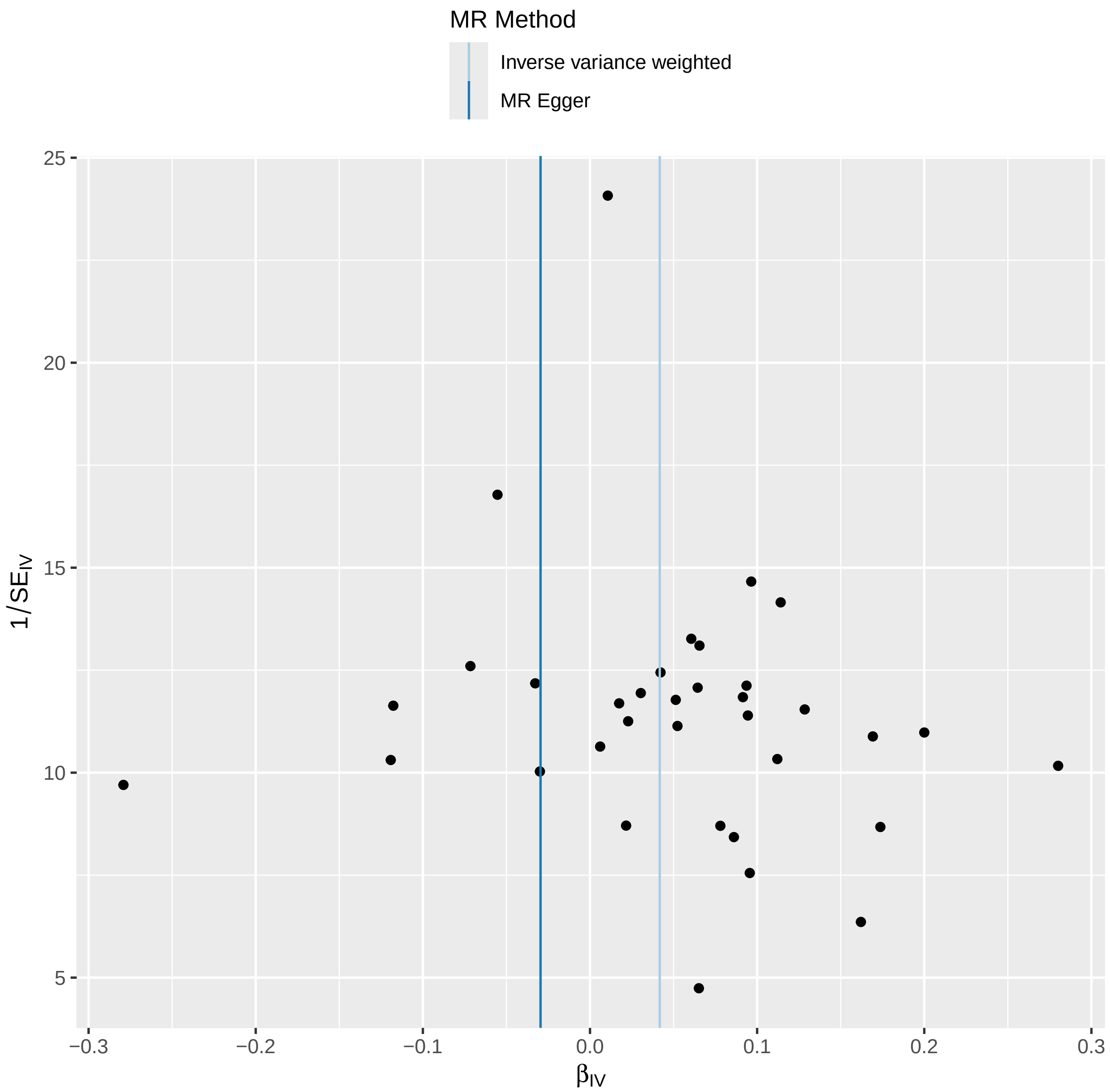

MR Method
Inverse variance weighted
MR Egger
25
20
15
10
5
1/SE$_{IV}$
−0.3
−0.2
−0.1
0.0
0.1
0.2
0.3
β$_{IV}$

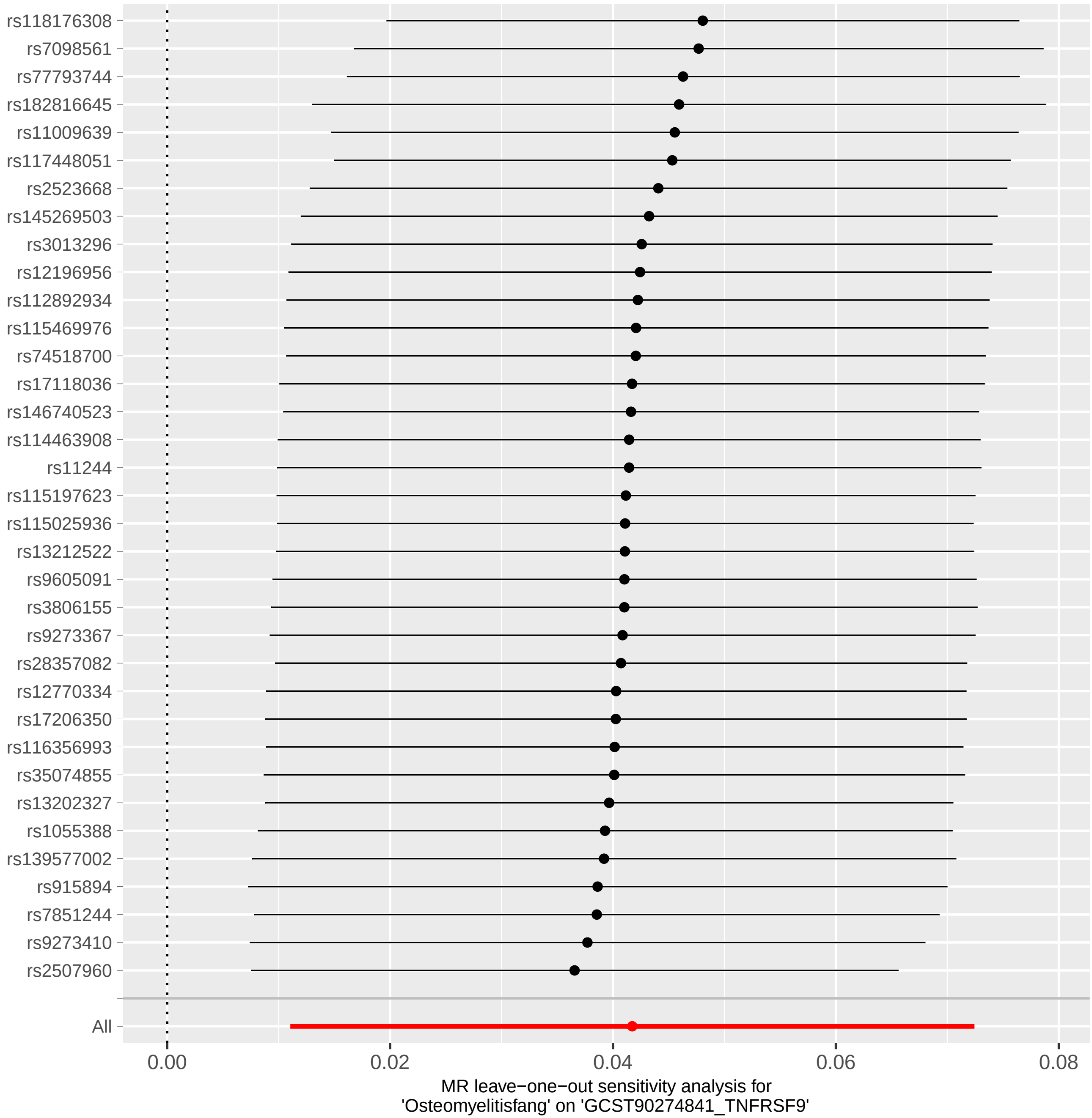
rs118176308
rs7098561
rs77793744
rs182816645
rs11009639
rs117448051
rs2523668
rs145269503
rs3013296
rs12196956
rs112892934
rs115469976
rs74518700
rs17118036
rs146740523
rs114463908
rs11244
rs115197623
rs115025936
rs13212522
rs9605091
rs3806155
rs9273367
rs28357082
rs12770334
rs17206350
rs116356993
rs35074855
rs13202327
rs1055388
rs139577002
rs915894
rs7851244
rs9273410
rs2507960
All
0.00
0.02
0.04
0.06
0.08
MR leave−one−out sensitivity analysis for
'Osteomyelitisfang' on 'GCST90274841_TNFRSF9'

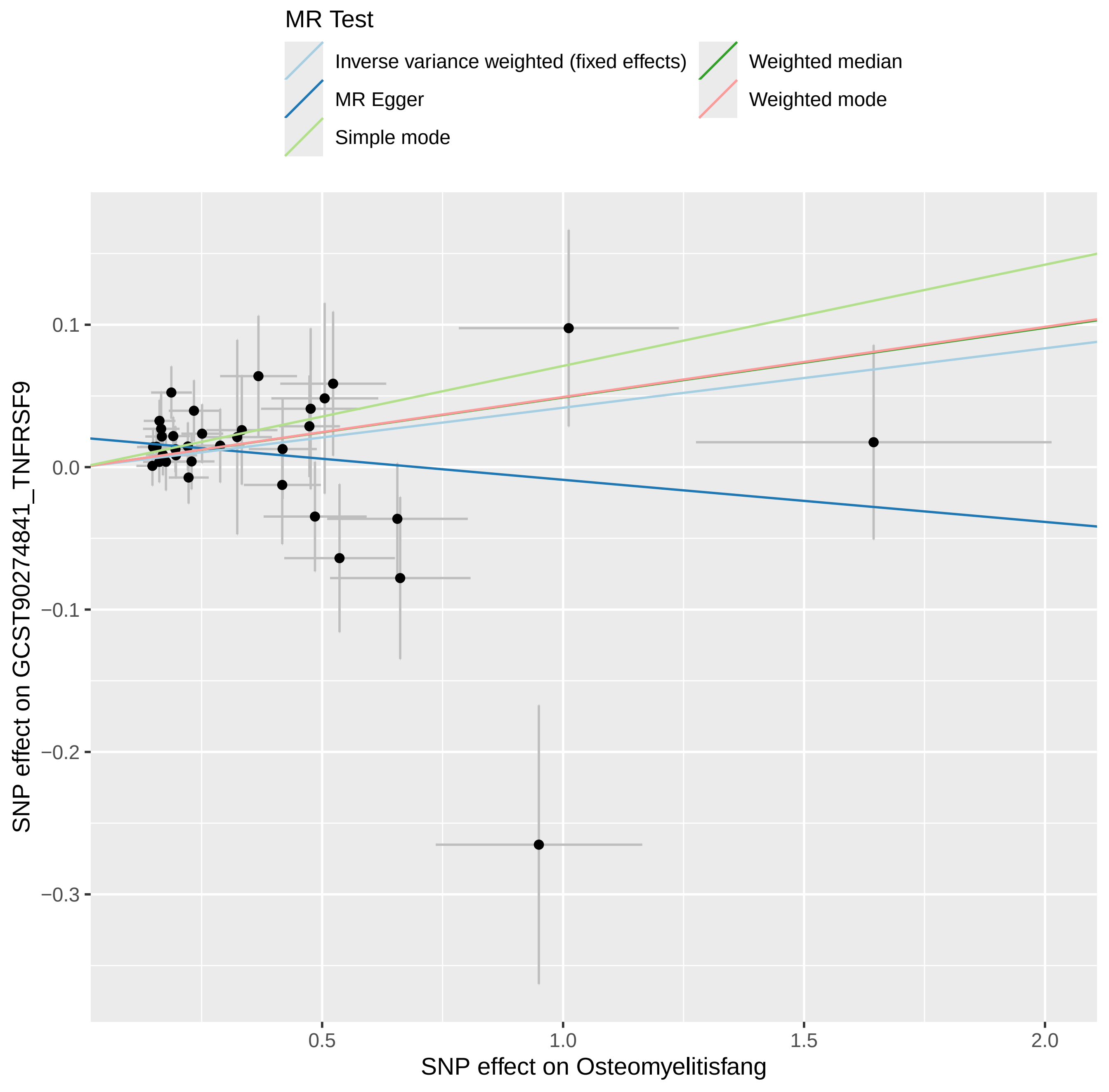
MR Test
Inverse variance weighted (fixed effects)
MR Egger
Simple mode
Weighted median
Weighted mode
SNP effect on GCST90274841_TNFRSF9
0.1
0.0
−0.1
−0.2
−0.3
0.5
1.0
1.5
2.0
SNP effect on Osteomyelitisfang

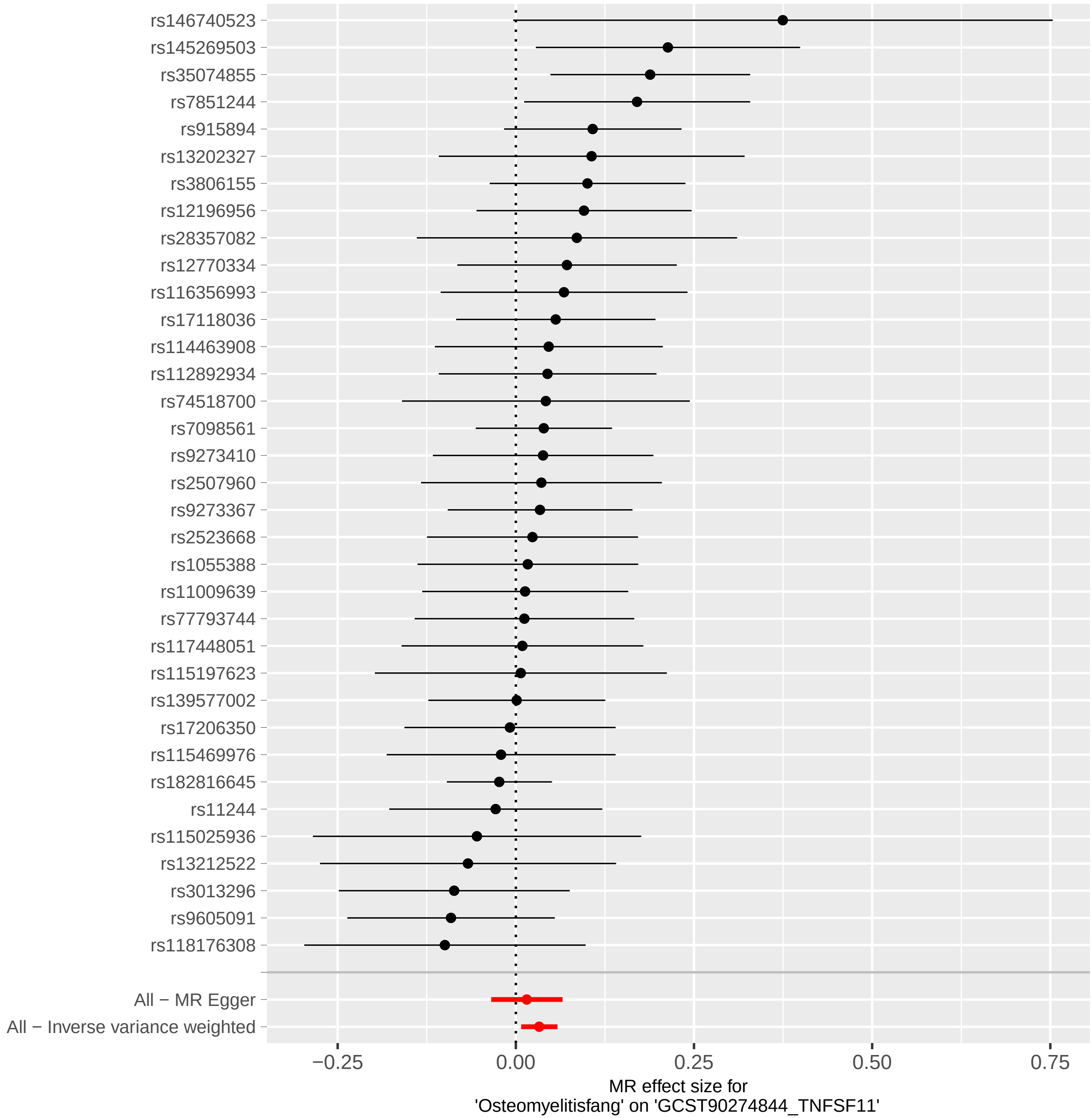
rs146740523
rs145269503
rs35074855
rs7851244
rs915894
rs13202327
rs3806155
rs12196956
rs28357082
rs12770334
rs116356993
rs17118036
rs114463908
rs112892934
rs74518700
rs7098561
rs9273410
rs2507960
rs9273367
rs2523668
rs1055388
rs11009639
rs77793744
rs117448051
rs115197623
rs139577002
rs17206350
rs115469976
rs182816645
rs11244
rs115025936
rs13212522
rs3013296
rs9605091
rs118176308
All − MR Egger
All − Inverse variance weighted
−0.25
0.00
0.25
0.50
0.75
MR effect size for
'Osteomyelitisfang' on 'GCST90274844_TNFSF11'

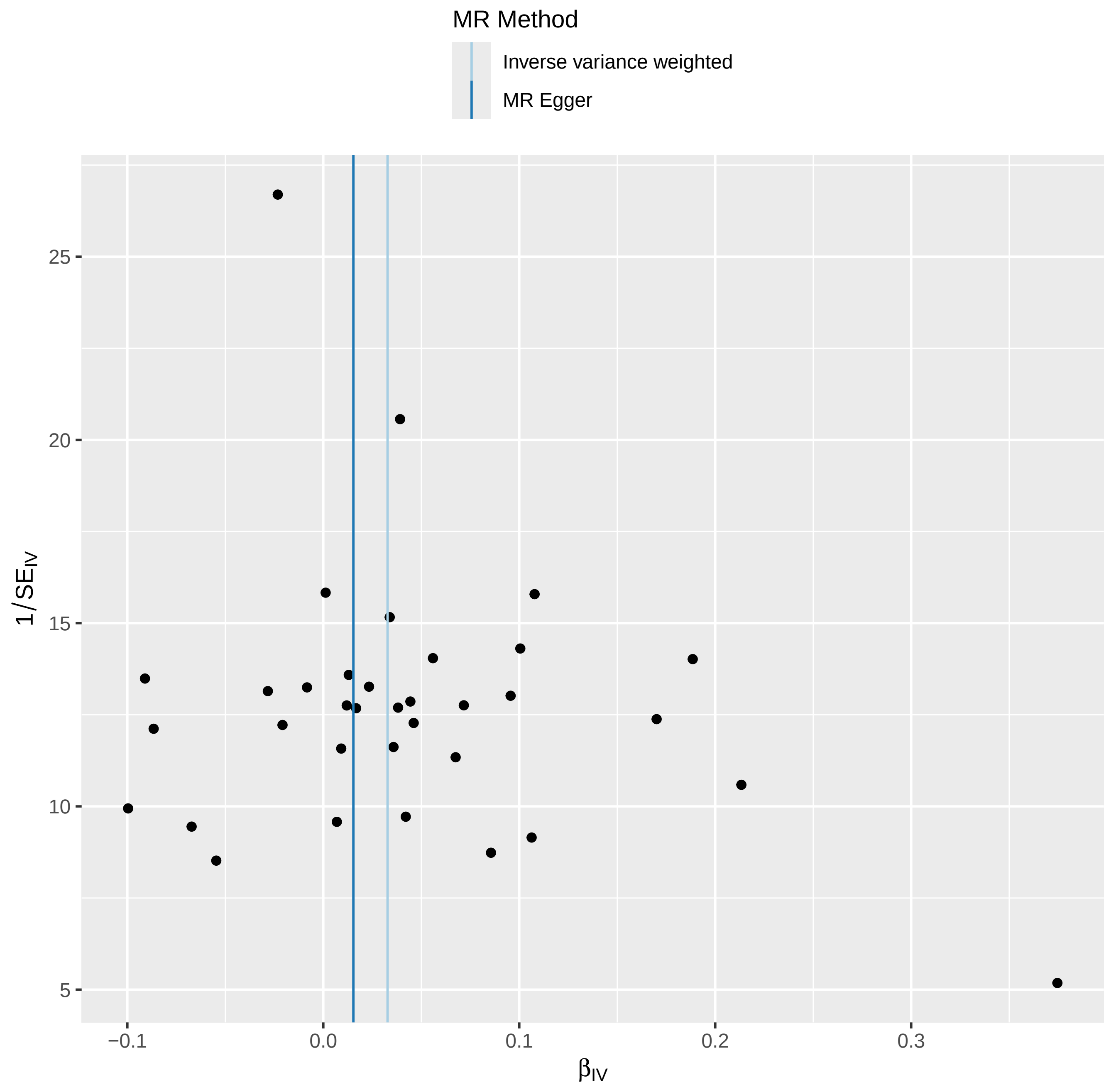

MR Method
Inverse variance weighted
MR Egger
1/SE_IV
25
20
15
10
5
−0.1
0.0
0.1
0.2
0.3
β_IV

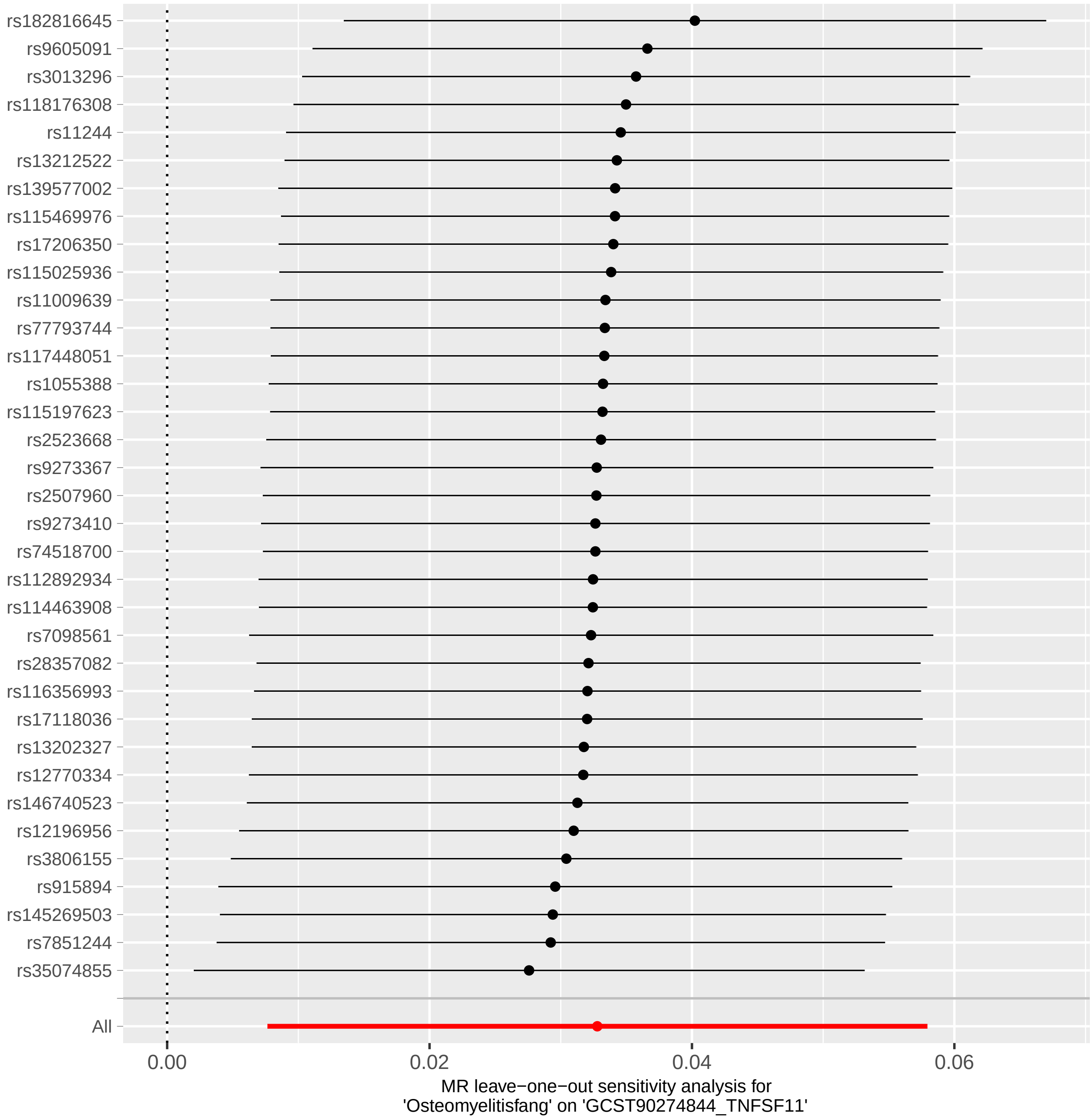

rs182816645
rs9605091
rs3013296
rs118176308
rs11244
rs13212522
rs139577002
rs115469976
rs17206350
rs115025936
rs11009639
rs77793744
rs117448051
rs1055388
rs115197623
rs2523668
rs9273367
rs2507960
rs9273410
rs74518700
rs112892934
rs114463908
rs7098561
rs28357082
rs116356993
rs17118036
rs13202327
rs12770334
rs146740523
rs12196956
rs3806155
rs915894
rs145269503
rs7851244
rs35074855
All
0.00
0.02
0.04
0.06
MR leave−one−out sensitivity analysis for
'Osteomyelitisfang' on 'GCST90274844_TNFSF11'

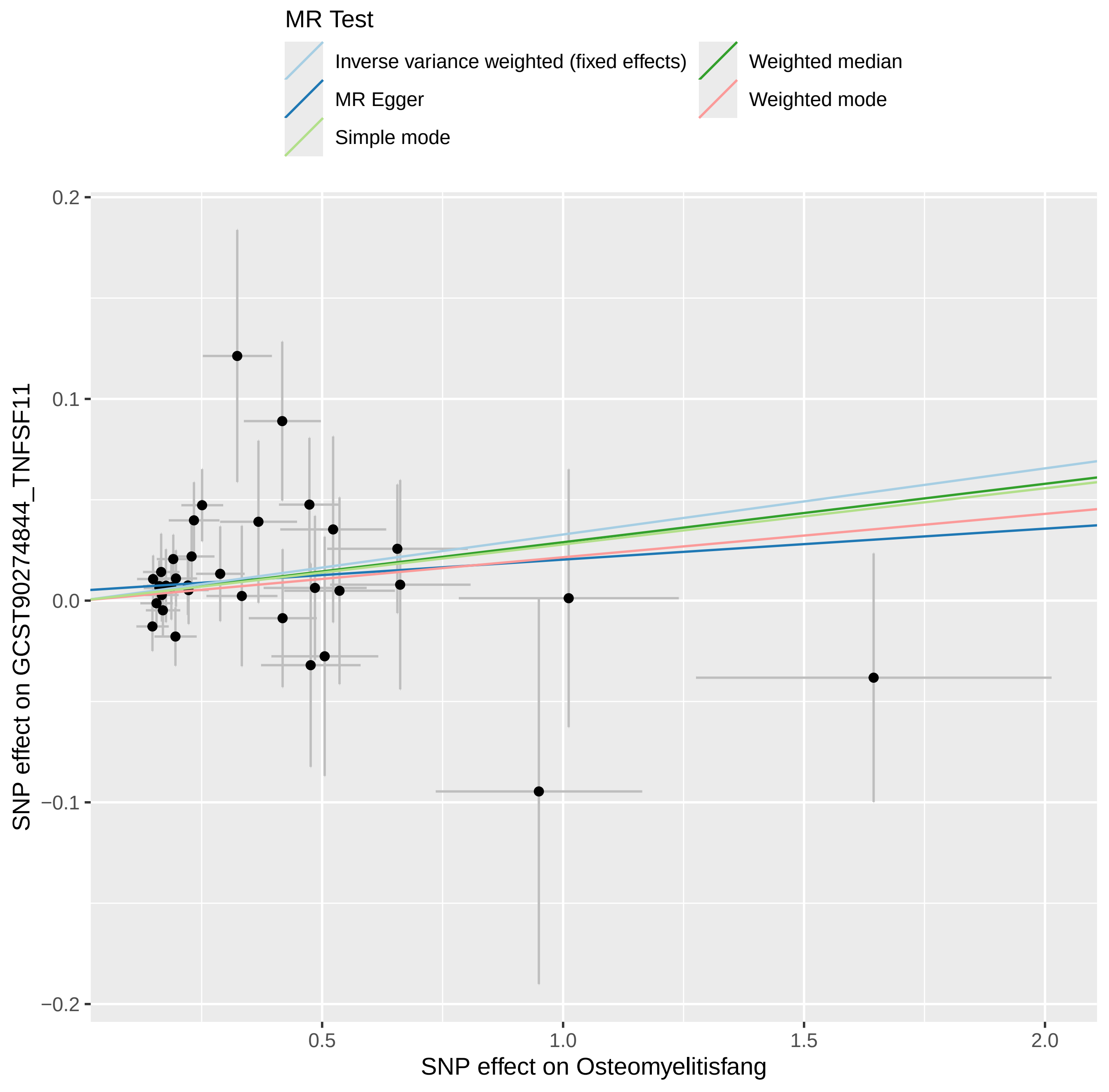
MR Test
Inverse variance weighted (fixed effects)
MR Egger
Simple mode
Weighted median
Weighted mode
SNP effect on GCST90274844_TNFSF11
SNP effect on Osteomyelitisfang
0.2
0.1
0.0
−0.1
−0.2
0.5
1.0
1.5
2.0
